# Merging Copper Catalysis with Nitro Allyl and Allyl Sulfone Derivatives: Practical, Straightforward, and Scalable Synthesis of Diversely Functionalized Allyl Boranes

**DOI:** 10.1021/jacsau.4c00809

**Published:** 2025-01-08

**Authors:** Nicolas Fincias, Louis Clavier, Cora Escande de Messières, Marianne Guillard, Mansour Dole Kerim, Nicolas Casaretto, Julian Garrec, Stellios Arseniyadis, Laurent El Kaïm

**Affiliations:** § Laboratoire de Synthèse Organique (LSO-UMR 7652) CNRS, Ecole Polytechnique, ENSTA-Paris, Institut Polytechnique de Paris 828 Bd des Maréchaux, 91128 Palaiseau Cedex, France; ‡ Laboratoire de Chimie Moléculaire (LCM-UMR 9168) CNRS, Ecole Polytechnique, Institut Polytechnique de Paris, 91128 Palaiseau, France; 4 Unité Chimie et Procédés (UCP) ENSTA-Paris, Institut Polytechnique de Paris, 828 Bd des Maréchaux, 91128 Palaiseau Cedex, France; † Queen Mary University of London, Department of Chemistry, Mile End Road, London E1 4NS, U.K.

**Keywords:** copper catalysis, borylation, nitroalkanes, allylsulfones, allylboronic esters, fluoroallyl boronic esters

## Abstract

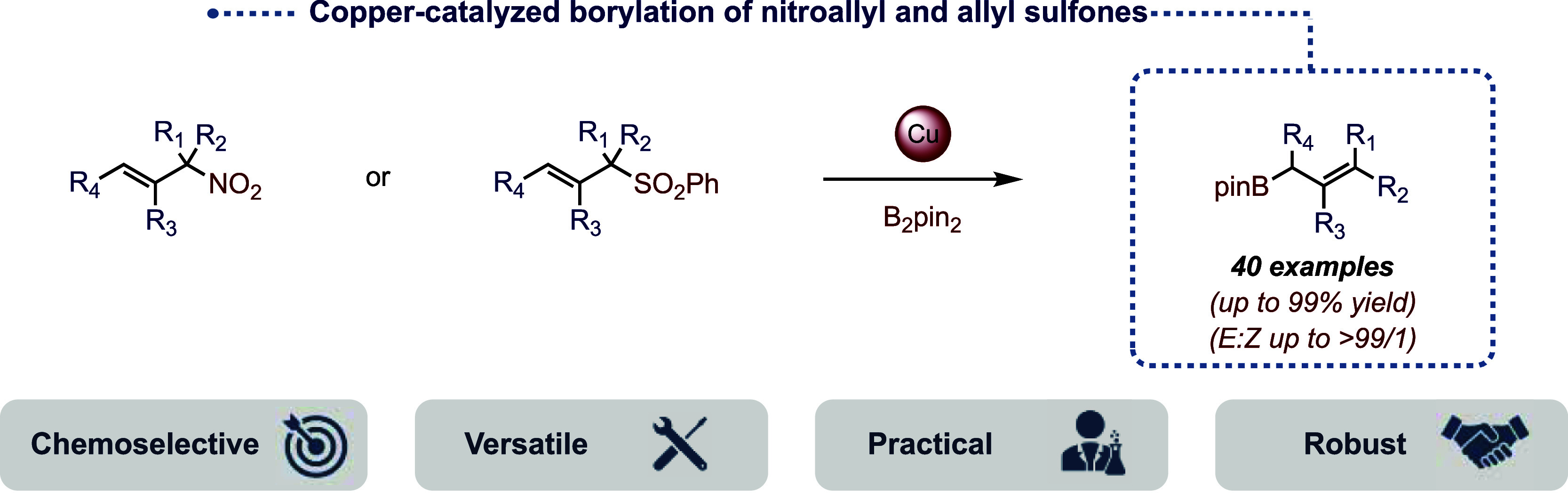

We report here the first example of a copper-catalyzed transformation involving nitro allyl derivatives. This borylation reaction, which exploits the high versatility of the aforementioned precursor, tolerates a variety of functional groups and allows practical, scalable, and highly straightforward access to diversely substituted allylboronic esters in high yields. The method was also extended to allyl sulfones, which provides a very complementary approach, offering additional structural diversity along with improved stereoselectivities. This new reactivity was further exploited to synthesize γ-fluoroallyl boronic esters as well as various synthetically useful building blocks through key post-functionalizations. Both the reaction mechanism and the chemoselectivity were rationalized experimentally and through DFT calculations.

## Introduction

**Figure 1:**
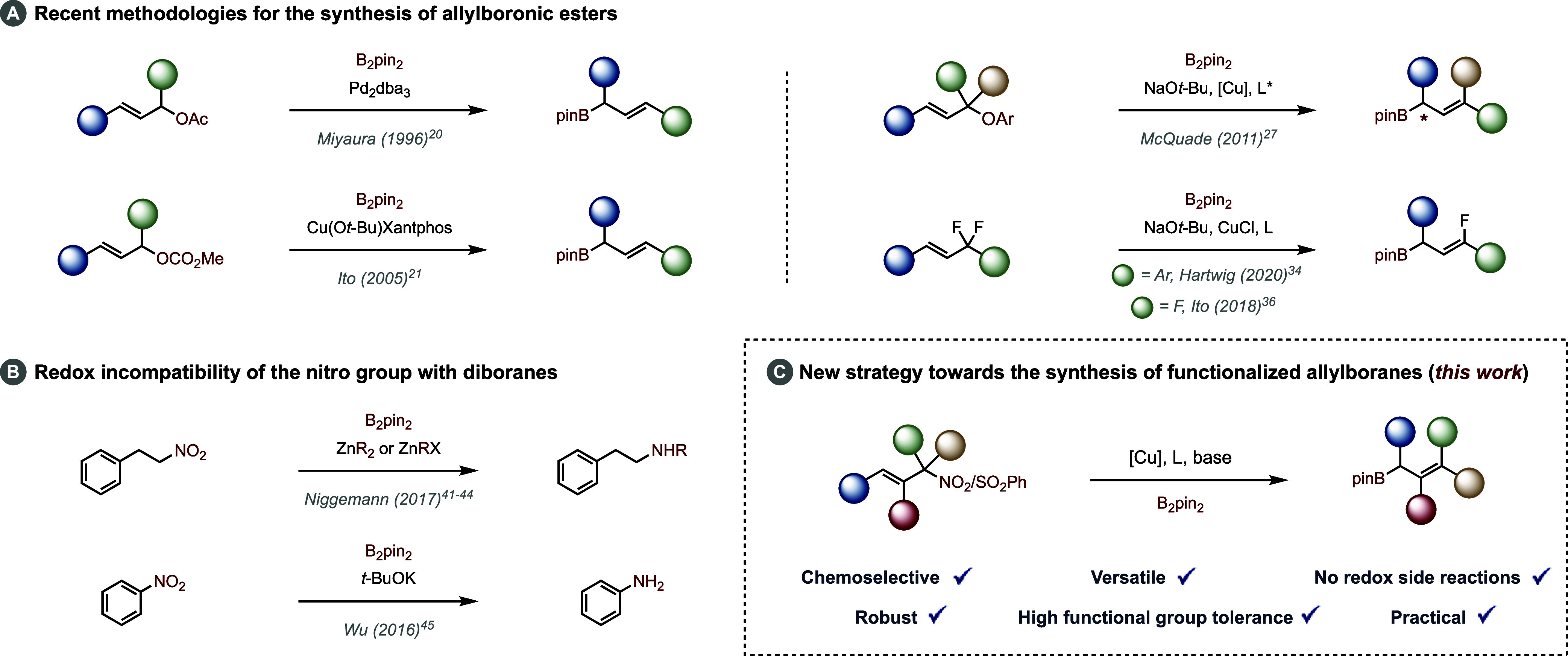
Strategies for the synthesis of allylboronic esters.

Allylboronic esters make up an important class of compounds used in a variety of transformations. Their moderate nucleophilic character offers a high functional group tolerance, making them ideal for the synthesis of structurally complex molecules.^1−8^ Allylboronic esters are also commonly used to react with aldehydes, ketones, and imines to form the corresponding homoallylic derivatives with high levels of enantio- and diastereocontrol.^9−17^ Hence, the development of new methods allowing straightforward, sustainable, and ideally inexpensive access to these compounds is of great importance. Several strategies have been reported over the years.^18^ The first one involves the addition of highly reactive organometallic species such as Grignard reagents on trialkoxyboranes and subsequent addition of a diol;^19^ however, although effective, this approach suffers from an easy 1,3-metallotropic shift of the organometallic species, which leads to a mixture of the linear and the branched allylboranes, thus limiting this method to the simplest allylboronic esters. Since the mid-90s and the pioneering work of Miyaura^20^ and Ito^21^ on the palladium- and copper-catalyzed borylation of allylic acetates (Figure 1A), the development of transition-metal-catalyzed borylation of allylic derivatives has tremendously facilitated the synthesis of these compounds. As a matter of fact, these two seminal works have prompted the development of several other borylation reactions using either different transition metal catalysts such as Fe, Co, Pt, and Ni or other leaving groups such as a phosphates, ketals, and fluorides.^22−36^ These methods are however usually limited in scope due to the syntheses of the allylic precursors, which often require the use of strong nucleophiles or reducing agents, ultimately restricting the functional group tolerance. The development of a borylation reaction involving allylic nitroalkanes appeared to us as a promising alternative, as it would circumvent all these issues and provide direct access to highly functionalized allylboranes. Indeed, the acidity of the protons α to the nitro group allows an easy functionalization of the allylic position using reactions that are mostly compatible with a large variety of functional groups such as the Henry and the Mannich reactions, the Knoevenagel condensation, or the Michael addition.^37−40^ This approach appeared slightly counterintuitive at first as there was evidence of redox incompatibility between the nitro group and diborane species leading to the formation of reduced compounds as shown by Niggemann^41−44^ and Wu^45^ (Figure 1B). Nonetheless, by taking advantage of the fast kinetics of the borylation reaction, we believed we could tame the reducing ability of the boronate species and favor the borylation process over reduction of the nitro group.

## Results and Discussion

Our journey began with an evaluation of the redox compatibility of allyl nitroalkanes with B_2_Pin_2_ in the presence of various copper salts. We chose allyl nitroalkane **1a** as our model substrate. The latter was prepared in four steps and a 28% overall yield starting from nitromethane (see Supporting Information for more details). Our first borylation reaction run in THF at rt using 10 mol % of CuI and 2 equiv of *t*-BuOLi led to the desired allylboronic ester **2a**, albeit in only 18% yield (Table 1, entry 1). Interestingly, changing the solvent to methanol considerably increased to 72% (Table 1, entry 3). A similar result was also observed when replacing *t*-BuOLi with *t*-BuOK, MeOLi, or Cs_2_CO_3_ (Table 1, entries 4–6). In sharp contrast, the use of a weaker base such as NaOAc did not lead to any product formation (Table 1, entry 7). The nature of the copper salt was also examined. Hence, replacing CuI by Cu(OAc)_2_ (Table 1, entry 8) appeared to be detrimental, while Cu(I) salts bearing a hard counteranion demonstrated a better catalytic activity as showcased by the use of CuCN, which led to full conversion and 89% isolated yield (Table 1, entry 10). The reaction could also be run using only 2 mol % of CuCN; however, longer reaction times were required (Table 1, entry 11). Finally, CuClXantphos (Table 1, entry 12), which was also employed by Ito and co-workers in the first borylation of allylic carbonates, demonstrated a similar efficacy but was discarded for the cost and practicality.

**Table 1:**
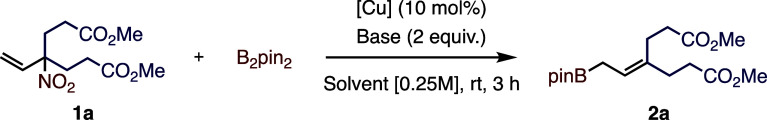
Systematic Study^a^

entry	[Cu]	base	solvent	conversion (%)^b^	yield (%)^b^
1	CuI	t-BuOLi	THF	100	18
2	CuI	t-BuOLi	DMF	100	55
3	CuI	t-BuOLi	MeOH	85	72
4	CuI	t-BuOLi	MeOH	85	63
5	CuI	MeOLi	MeOH	85	72
6	CuI	Cs_2_CO_3_	MeOH	85	72
7	CuI	NaOAc	MeOH	0	0
8	Cu(OAc)_2_	MeOLi	MeOH	80	55
9	CuBr Me_2_SH	MeOLi	MeOH	85	72
10	CuCN	MeOLi	MeOH	100	91(89^c^)
11	CuCN	MeOLi	MeOH	100	85^d^
12	CuClXantphos	MeOLi	MeOH	95	85
13		MeOLi	MeOH	0	0
14	CuCN		MeOH	0	0

a Reaction conditions: **1a** (0.2 mmol, 1 equiv), B_2_pin_2_ (0.4 mmol, 2 equiv), base (0.4 mmol, 2 equiv), solvent (0.8 mL), rt, 3 h.

b Determined by NMR using 1,3,5-trimethoxybenzene as an internal standard.

c Isolated yield.

d Reaction run using 2 mol % of [Cu].

**Figure 2:**
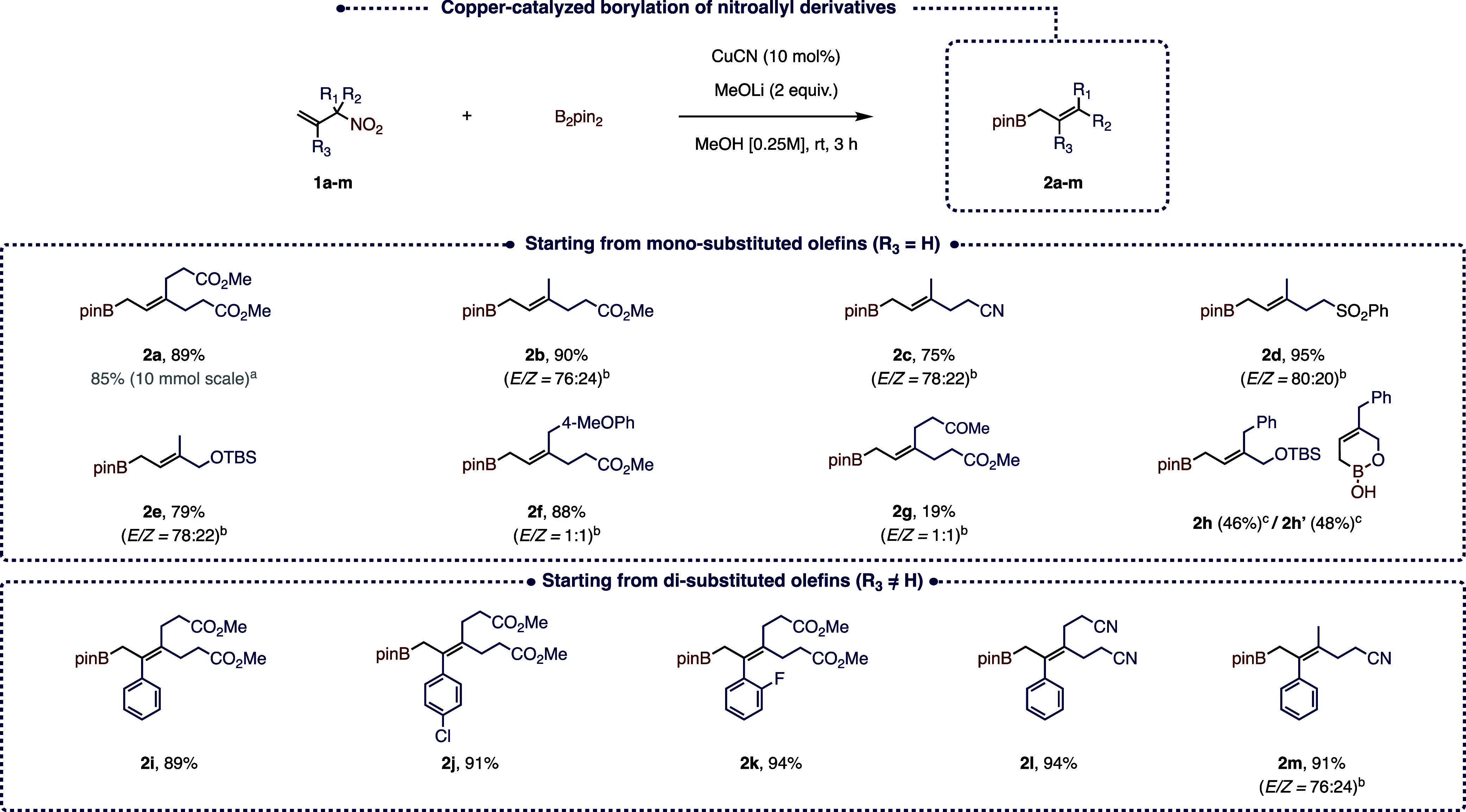
Substrate scope. (a) Reaction run using 2 mol % of CuCN. (b) Determined by 1H NMR on the crude reaction mixture. (c) Reaction run over a period of 24 h.

With these first results in hand, we next explored the scope of the reaction by subjecting a variety of allylic nitroalkanes to our optimized borylation conditions [CuCN (10 mol %), MeOLi (2 equiv), B_2_pin_2_ (2 equiv), MeOH, and rt]. As shown in Figure 2, all the resulting allylboranes **2a**–**g** were obtained in high yields independently of the nature of the starting terminal olefin **1a**–**g**, which also showcases the high functional group tolerance of the reaction (CO_2_Me, CN, SO_2_Ph, OTBS, and 4-MeOPh), although the reaction with the ketone-containing substrate proved to be low-yielding. In the case of the nonsymmetrical α-disubstituted nitro allyl derivatives, the two resulting diastereoisomers were generally obtained in a roughly 4:1 ratio, although in the case of compound **1f**, the two stereoisomers were obtained in equal amounts, thus suggesting the importance of the size of the substituents on the selectivity outcome. Interestingly, in the case of compound **1h** bearing a silyl-protected allylic alcohol, the two stereoisomers were obtained in a roughly 1:1 ratio, but the *Z* isomer further reacted to afford the corresponding dihydro-oxaborinin product **1h′**. The reaction also proved equally effective when applied to gem-disubstituted terminal olefins, such as **1i**–**m**. Indeed, the corresponding allylboranes **2i**–**m** were obtained in high yields ranging from 89% to 94% with once again a roughly 3:1 ratio of the two diasteroisomers in the case of the nonsymmetrical α-disubstituted precursors.

Finally, the reaction proved to be easily scalable, as the borylation of nitroalkane **1a** run on a 2.6 g scale (10 mmol) afforded the corresponding borylated product **2a** in 85% isolated yield.

Overall and as anticipated, the use of nitro allyl precursors allowed us to easily introduce functional diversity around the allyl moiety with groups such as esters, cyanides, sulfones, and silyl-protected alcohols. But most importantly, our borylation method proved particularly effective as all the allylic nitroalkanes tested readily underwent borylation independently of the substitution pattern on the allyl moiety, thus offering a straightforward access to tri- and tetrasubstituted allylboranes in usually excellent yields. This appeared to be all the more appealing that this allylic substitution approach offers a new entry to functionalized tetrasubstituted allylboronates.^2^

### Reaction Mechanism Study

In contrast to other leaving groups employed in previous borylation studies,^21,46−48^ we can reasonably hypothesize that the NO_2_ moiety could interact with the metal at different stages of the mechanism and potentially favor one pathway over another. To address this question, we modeled the entire reaction mechanism by means of DFT calculations using 3-methyl-3-nitrobutene as a model substrate, CuXantphos(Bpin) as the active organometallic species, and the ωB97X-D/6-311+G(2d,2p)//ωB97X-D/6-31G(d) level of theory.^49−51^ Although the reaction scope was run using CuCN, we decided to compute the reaction profile using CuXantphos as the speciation of the latter is well reported, and the results are relatively similar. The 3D molecular structures of the reactants and products of our model reaction are shown in Figure S1 in the Supporting Information. The solvent (methanol) was represented by mean of a polarizable continuum model.^52^ All calculations were performed using the Gaussian 16 software (see the Supporting Information for more details).^53^

**Figure 3:**
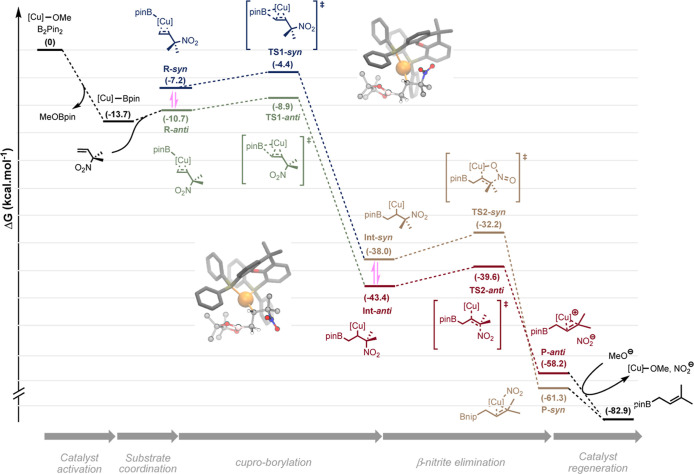
Free energy profile of the cupro-borylation of 3-methyl-3-nitrobutene followed by the β-nitrite elimination leading to the corresponding allylborane. Both *anti* and *syn* pathways are represented for each chemical step. Vertical arrows in magenta indicate a conformational equilibrium. The 3D molecular structures of **Int-***anti* and **Int-***syn* are shown as insets (for the sake of clarity, all H atoms are hidden except those close to the reaction center). The structures of the end points of the reaction (in black here) are shown in Figure S1 in the Supporting Information. The remaining molecular structures for the cupro-borylation and β-nitrite elimination steps are shown in Figure S2 in the Supporting Information.

As shown in the free energy profile represented in Figure 3, the reaction starts with activation of the catalyst, followed by its coordination with the substrate. This leads to the formation of the reactive complex, which exhibits two conformers, noted **R-***syn* and **R-***anti*, that differ by the relative positioning of the NO_2_ group with respect to the Cu (the corresponding 3D molecular structures are shown in Figure S2 in the Supporting Information). The Cu···NO_2_ distance in **R-***syn* is about 4 Å, which is too high for establishing an interaction (either stabilizing or destabilizing) between the two moieties. It turns out that the **R-***anti* conformer is more stable than **R-***syn* by 3.5 kcal mol^–1^.

The following chemical step, namely, the cupro-borylation of the alkene moiety, is then initiated from either the **R-***syn* or the **R-***anti* conformer. This results in two distinct transition states (TSs), namely, **TS1-***syn* and **TS1-***anti* (the corresponding 3D molecular structures are also shown in Figure S2 in the Supporting Information). The free energy of **TS1-***syn* is significantly higher by 4.5 kcal mol^–1^ than that of **TS1-***anti*, which is in line with the relative stability of **R-***syn* versus **R-***anti* previously discussed.

This cupro-borylation leads to the formation of two conformers of the same alkyl copper intermediate noted **Int-***syn* and **Int-***anti*. The latter is again the most stable by 5.4 kcal mol^–1^ with respect to **Int-***syn*. We noticed that the energetic separation between the *syn*- and *anti*-pathways gets larger and larger along the **R** → **TS1** → **Int** progression, as the [Cu] and NO_2_ moieties get closer and closer. In the **R-***syn* state, the Cu···NO_2_ distance is about 3.2 Å, which allows a significant interaction (either stabilizing or destabilizing) between the two moieties.

To proceed further, we evaluated the strength of the intramolecular noncovalent interactions (NCI) within **Int-***anti* vs **Int-***syn* by means of a so-called NCIPlot analysis (see Supporting Information for more details).^54,55^ Our calculations revealed that **Int-***syn* exhibits a local strong Pauli repulsion between the electron cloud of the Cu atom and that of the nearby oxygen of the NO_2_ group (see Figure S3 in the Supporting Information).

The next chemical step is β-nitrite elimination, which again proceeds through either an *anti*- or a *syn*-type mechanism. The corresponding TSs, namely, **TS2-***anti* and **TS2-***syn* exhibit again a significant free energy difference (7.4 kcal mol^–1^) in favor of the *anti* mechanism. **TS2-***anti* and **TS2-***syn* lead to **P-***anti* and **P-***syn*, respectively, which ultimately decompose to form the final allylborane through a highly stabilizing process. We note that there is an inversion of the relative stability between **P-***anti* and **P-***syn* that we attribute to the nature of the Cu···NO_2_ interaction within **P-***anti* and **P-***syn*, which is ionic and covalent, respectively. However, it should be stressed that **P-***anti* can be rapidly turned into **P-***syn* by migrating the NO_2_^–^ ion onto the Cu center. Thus, the main factor governing the *anti* vs the *syn* β-nitrite elimination is the activation barrier. Overall, considering the entire reaction pathway, including the cupro-borylation and the β-nitrite elimination steps, our calculations support the *anti* mechanism.

### Extension of the Scope

**Figure 4:**
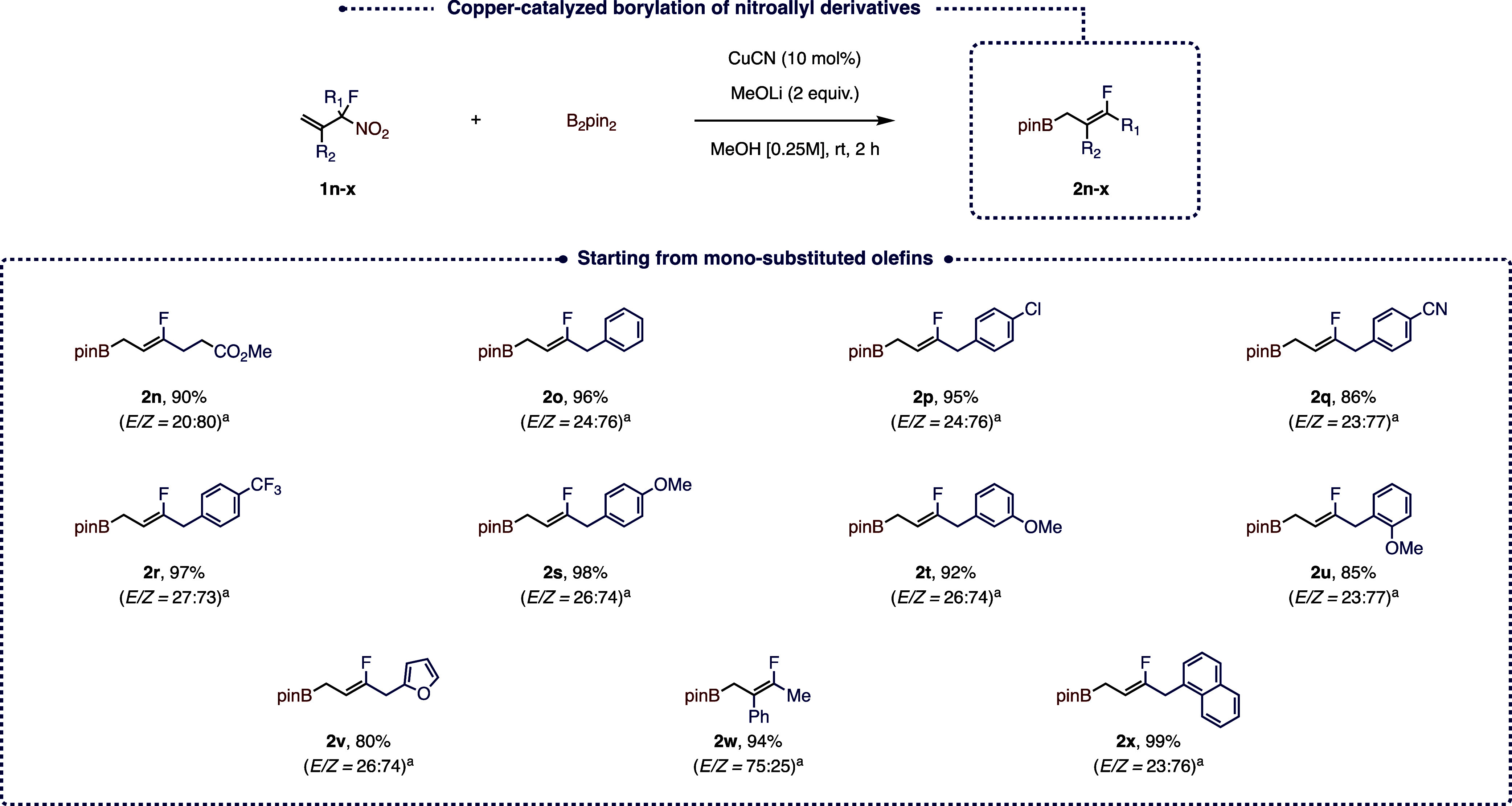
Synthesis of γ-fluoroallyl boronic esters. (a) Determined by ^1^H NMR on the crude reaction mixture.

Encouraged by these results, we decided to extend the method to fluorinated allylic nitroalkanes as this would provide a straightforward access to γ-fluoro-allylboronic esters without needing to use ozone-depleting fluorine-containing precursors.^11^ To that effect, a fluorination procedure was developed on various nitro olefin precursors using SelectFluor as an electrophilic source of fluorine.^56^ The corresponding fluorinated nitro allyl derivatives **1n**–**x** were obtained in high yields (see Supporting Information for full details). The latter were engaged in our borylation reaction under the previously optimized conditions, affording the exclusive formation of the γ-fluoro-allylboronic esters **2n**–**x** in excellent yields ranging from 85 to 99% (Figure 4). Once again, the reaction tolerated a variety of functional groups including cyanides, esters, and halogens and did not lead to any loss of fluorine during the process, which was one of the risks as fluoro-allyl derivatives are known to readily undergo borylation under Cu-catalysis.^32,47,48^

### Rationalization

**Figure 5:**
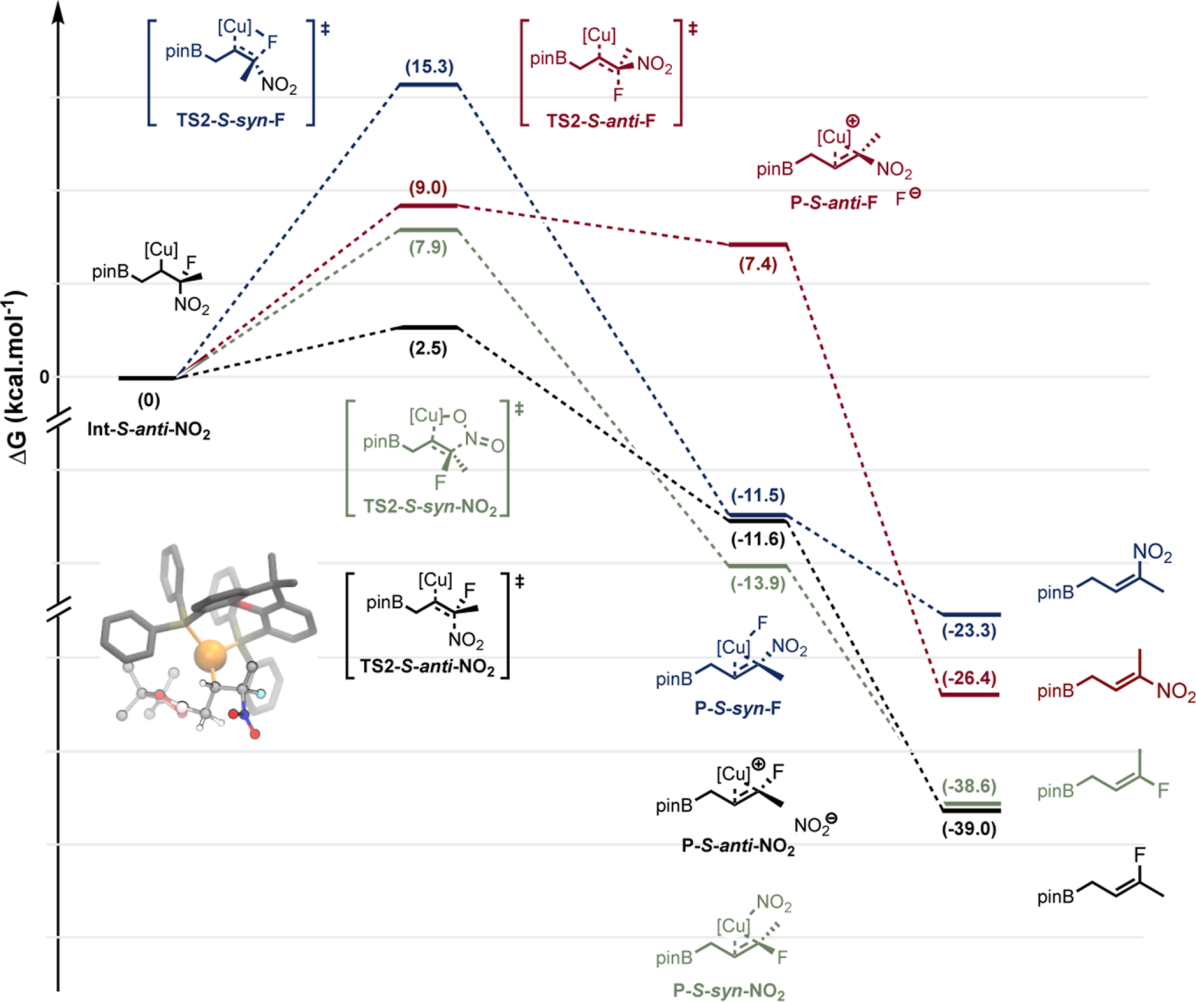
Comparison of the β-elimination pathways with the fluorine or nitro leaving groups, starting from the *S* intermediate. The 3D molecular structures of all TSs, adducts, and final products are shown in Figure S4 in the Supporting Information.

To rationalize the excellent chemoselectivity observed, we performed additional DFT calculations using a fluorinated model substrate. As we previously showed that both the *anti*- and the *syn*-cupro-borylations lead to the same intermediate, albeit exhibiting different conformers in thermal equilibrium, we decided to start from the fluorinated intermediate and focus on the subsequent elimination step. More specifically, we chose as a molecular model the intermediates that would result from the cupro-borylation of 3-fluoro-3-nitrobutene (Figures 5 and S5 in the Supporting Information). There are two such intermediates owing to the two configurations (*R* or *S*) of the carbon bearing the NO_2_ and the F leaving groups. Figure 5 shows the free energy profile starting from the *S* intermediate with the NO_2_ group in the *anti* conformation, as noted for **Int-***S*-*anti***-NO**_**2**_ hereafter. This conformer was chosen as a reference as it turns out to be the most stable, as shown in our previous discussion on the borylation of 3-methyl-3-nitrobutene.

From **Int-***S*-*anti***-NO**_**2**_, the system can proceed through four different pathways, i.e., an *anti*- or syn-elimination of either the fluorine or the nitro group. The four corresponding TSs are noted **TS2-***S*-*anti***-F**, **TS2-***S*-*syn***-F**, **TS2-***S*-*anti***-NO**_**2**_, and **TS2-***S*-*syn***-NO**_**2**_ in Figure 5, and the resulting adducts are noted **P-***S*-*anti***-F**, **P-***S*-*syn***-F**, **P-***S*-*anti***-NO**_**2**_, and **P-***S*-*syn***-NO**_**2**_, respectively, while the final decomposition products are the γ-nitro-*E*-allylboronic, γ-nitro-*Z*-allylboronic, γ-fluoro-*Z*-allylboronic, and γ-fluoro-*E*-allylboronic esters, respectively.

The elimination of the nitro group corresponds to the smallest barriers, and the resulting γ-fluoro-*Z*/*E*-allylboronic esters are much more stable (by > 12 kcal mol^–1^) than their γ-nitro counterparts, which would result from the elimination of the fluorine atom instead. Overall, our calculations provide some rationale for the observed chemoselectivity toward the fluorinated compounds. However, our model cannot capture the observed diastereoselectivity (*Z* versus *E*) of the final products, as **TS2-***S*-*anti***-NO**_**2**_ is only 5.4 kcal mol^–1^ lower in free energy than **TS2-***S*-*syn***-NO**_**2**_. We attribute this discrepancy to the simplicity of our molecular model (which corresponds to **1m**–**w** with R_1_ = Me and R_2_ = H) compared with the actual compounds considered in our scope. The same conclusions can be drawn from the calculations initiated from **Int-***R*-*anti***-NO**_**2**_, i.e., the intermediate in which the carbon bearing the leaving groups has an *R* configuration (see the free energy surface and structures in Figures S5 and S6 in the Supporting Information, respectively).

### Extension of the Method

**Figure 6:**
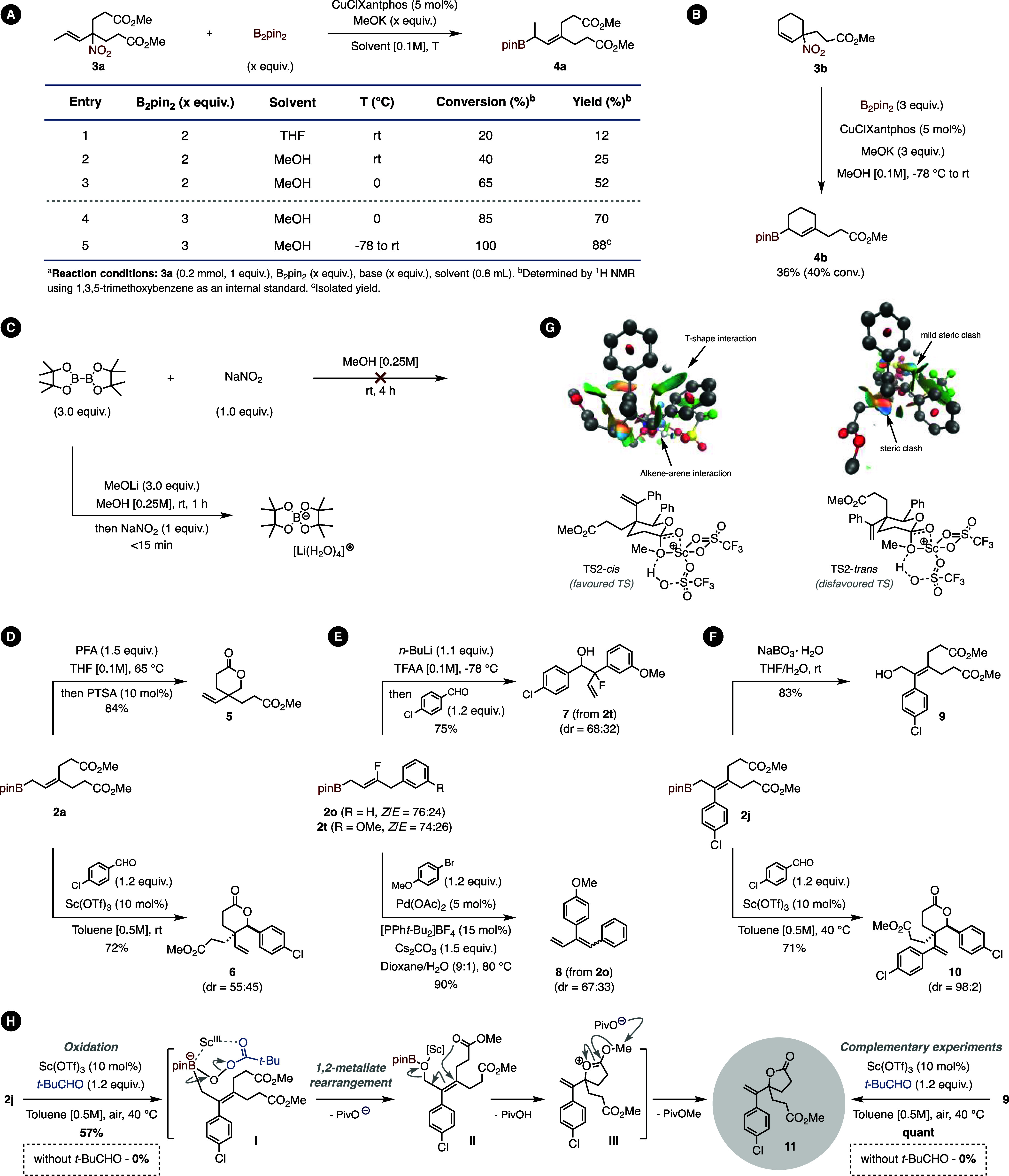
(A) Optimization of the reaction conditions for 1,2-disubstituted alkenes. (B) Extension to internal alkenes. (C) Redox compatibility test between diborane species and sodium nitrite (top). Redox compatibility test between ate complex and sodium nitrite (bottom). (D) Synthesis of δ-lactones via allylation and subsequent cyclization. (E) Derivatization of monofluoro allylboranes: allylation of *p*-chlorobenzaldehyde (top); synthesis of dienes through a Pd-catalyzed Suzuki/dehydrofluorination cascade (bottom). (F) Derivatization of tetrasubstituted allylboranes: oxidation to the corresponding allylic alcohol (top); synthesis of γ-lactones through an oxidation/lactonization cascade (center); and diastereoselective synthesis of δ-lactones via allylation and subsequent cyclization. (G) NCIplot of TSs associated with the elimination of methanol: strong stabilizing interactions are highlighted in blue, weak interactions are highlighted in green, and strong destabilizing interactions are highlighted in red. TS leading to the major compound with the two aryl rings in a *cis* conformation (left); TS leading to the formation of the minor compound with the two aryl rings in a *trans* conformation (right). (H) *t*-BuCHO/Sc(OTf)_3_-mediated formal γ-oxidation/lactonization of **2j** and **9** leading to γ-lactone **11** [PFA = paraformaldehyde].

Following these results, we decided to extend the method to 1,2-disubstituted allylic nitroalkane precursors with the idea of further extending the scope to secondary allylic boronic esters. To that effect, **3a** was prepared and subjected to our borylation conditions. Unfortunately, no conversion was observed, and new conditions needed to be found (Figure 6, **A**). The typical reaction conditions developed by Ito and co-workers [B_2_pin_2_ (2 equiv), MeOK (2 equiv), CuClXantphos (5 mol %), THF, and rt] led to only 20% conversion of the starting material and 12% yield (entry 1). Luckily, by switching the solvent to MeOH and by running the reaction at 0 °C instead of rt, we were able to boost the yield to 52% (entry 3). Increasing the amount of B_2_Pin_2_ to 3 equiv also improved the yield (70%, entry 4); however, the best result was obtained by adding the base at −78 °C and letting the temperature slowly rise to rt. This afforded the desired allylboronic ester **4a** in 88% yield (entry 5). The same conditions were eventually applied to the nitrocyclohexene derivative **3b** (Figure 6, **B**); however, the reaction was more sluggish, affording **4b** in 36% yield (90% brsm).

To identify any off-cycle reaction that could consume B_2_Pin_2_ and thus explain the low conversions observed, we also ran a series of test reactions (Figure 6, **C**). Based on previous reports describing the reduction of the nitro group by diborane species, we first envisioned that B_2_Pin_2_ could potentially interact with the allylic nitroalkane, leading to the formation of reduced products. However, none were detected, even in trace amounts. Consequently, we hypothesized that the nitrite anion released during the reaction could perturb the catalytic cycle by interacting with the copper complex and B_2_Pin_2_. To test this hypothesis, we generated the ate complex in MeOH in the presence of sodium nitrite. Interestingly, after a few minutes, a slight increase in temperature was observed, accompanied by gas evolution and the formation of a precipitate, the structure of which was confirmed by X-ray analysis (Figure 6, **C**), thus demonstrating the presence of an off-cycle reaction leading to in situ B_2_Pin_2_ degradation. As a consequence, in the case of more hindered allylic nitroalkanes, which are arguably less reactive, the nitrite released during the reaction oxidizes the ate complex generated in situ, which inhibits the reaction and explains the poor conversion observed.

### Post-functionalizations

With all of these highly substituted allylboranes in hand, we were curious to exploit their inherent reactivity in various post-transformations. In this context, allylborane **2a** was easily converted to the corresponding δ-lactone **5** in the presence of paraformaldehyde (1.5 equiv) and PTSA (10 mol %) (Figure 6, **D**). When the reaction was run with *p*-chlorobenzaldehyde, the use of Sc(OTf)_3_ was necessary to increase the rate of the reaction. The resulting δ-lactone **6** was obtained in a good 72% yield, albeit with a poor diastereoselectivity of 45:55 in favor of the trans-lactone.^57−59^

Two γ-fluoroallylboranes (**2o** and **2t**) were also engaged in post-transformations (Figure 6, **E**). Hence, the use of **2t** in the allylation of *p*-chlorobenzaldehyde under the conditions developed by Aggarwal and co-workers^60^ afforded the corresponding β-fluoro alcohol **7** in 75% yield. Compound **2o**, on the other hand, was engaged in a Pd-catalyzed Suzuki/dehydrofluorination cascade leading to the formation of diene **8**, which was obtained in an excellent 90% yield. More hindered allylboranes such as **2i** were also very reactive (Figure 6, **F**). The C–B bond, for instance, was readily oxidized with NaBO_3_·H_2_O to afford the corresponding allylic alcohol **9** in 83% yield. The allylation of *p*-chloro benzaldehyde with **2j** afforded the corresponding δ-lactone **10** in 71% yield and excellent 98:2 diastereoselectivity, which contrasts with the roughly 1:1 ratio obtained when the reaction was run on **2a**. This difference in diastereoselectivity can be explained by the presence of π-interactions between the two aryl moieties. This hypothesis was verified by modeling the two cyclization modes associated with the formation of the *trans* and the *cis* lactones (Figure 6, **G**). Analysis of noncovalent interactions using NCIPlot software (see Supporting Information for additional details) in the TSs of the methanol elimination during the lactonization step revealed the presence of a T-shaped π-stacking interaction between the aryl groups, as well as an alkene-arene π-stacking interaction during the formation of the *cis* lactone. These stabilizing interactions, which are not present in the TS leading to the formation of the *trans* lactone, offer a free energy difference of 4.5 kcal mol^–1^ in favor of the formation of the *cis* lactone. Surprisingly, when **2j** was reacted with pivaldehyde, the expected allylation product could not be detected; instead, we isolated γ-lactone **11** in 57% yield (Figure 6, **H**). To understand this unusual formal γ-oxidation/lactonization cascade, we carried out a set of experiments. The first reaction run in the absence of pivaldehyde did not produce γ-lactone **11**, but instead afforded alcohol **9** in 19% isolated yield. Interestingly, when the latter was subjected to the previous reaction conditions in the presence of pivaldehyde, γ-lactone **11** was obtained quantitatively, whereas in the absence of pivaldehyde, the reaction failed to produce the desired lactone. This suggests that pivaldehyde is crucial in both the oxidation of the allylboronate and the lactonization. With these results in hand, we propose a mechanism where pivaldehyde is first oxidized to the corresponding peracid, which then reacts with the pinacolboronic ester to form “ate” complex **I**. The latter undergoes a 1,2-metalate rearrangement to generate intermediate **II** along with a pivalate anion. In the presence of Sc(OTf)_3_, the borylated alcohol is eliminated through an S_N_2′-type reaction facilitated by one of the ester groups, thus generating oxonium **III**. Finally, the pivalate anion released in the previous step abstracts the methyl group to produce desired γ-lactone **11** along with methyl pivalate.

### Allylic Sulfones as Complementary Partners

The power of the borylation of the nitro allyl derivatives lies in the ease by which the precursors can be prepared and the effectiveness of the method to prepare the corresponding allyl boranes. Nonetheless, several limitations need to be considered. Indeed, alkylation of the nitro derivatives is often hampered by a competing *O*-alkylation. Additionally, the high acidity of the nitro derivatives bearing a hydrogen on the adjacent carbon prevents the borylation of monosubstituted derivatives, as the basic conditions required for the activation of the catalyst leads to the formation of nitronate byproducts. To address these challenges, we decided to explore the use of allyl sulfones, as they are known to be much less acidic (p*K*_a_ around 25 in DMSO) than their nitro counterparts. As a matter of fact, the ability of acidic allylic sulfones to react in copper-catalyzed allylation reactions with Grignard reagents without deprotonation was a good indication of their compatibility with our borylation conditions. Moreover, while allylic sulfones have been often used in cross-coupling processes as electrophiles to construct C–C and C–heteroatom bonds,^61−66^ they have been, to the best of our knowledge, only scarcely used in borylation reactions.^67^

**Figure 7:**
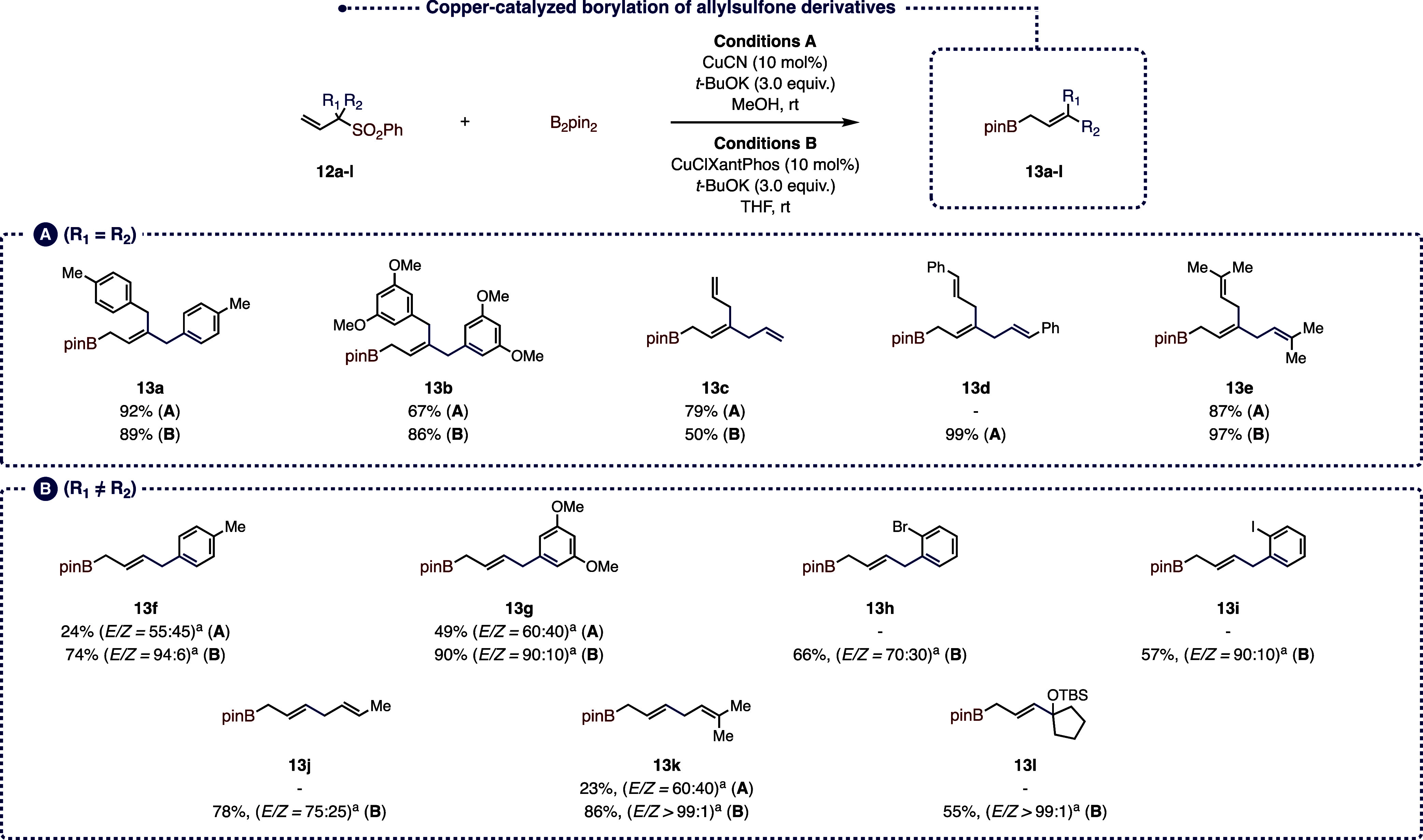
Copper-catalyzed borylation of allyl sulfone derivatives. (a) Determined by ^1^H NMR on the crude reaction mixture.

To test the reaction, a series of experiments were undertaken using allyl sulfone **12a** as a model substrate (see Supporting Information for complete optimization). Among all of the conditions tested, two sets of conditions led to the best results (Figure 7). The first one involved the use of CuCN (10 mol %) and *t*-BuOK (3.0 equiv) in MeOH at rt (Conditions A), while the second one used CuClXantPhos (10 mol %) and *t*-BuOK (3.0 equiv) in THF at the same temperature (conditions B). As a general trend, in the case of α-disubstituted allylic sulfones such as **12a**–**e** (Figure 7, **A**), we were pleased to observe the formation of the corresponding pinacolboronic esters **13a**–**e** in good to excellent yields (up to 99%) independent of the conditions used. Surprisingly, the results were significantly different when we conducted the reactions on α-monosubstituted allylic sulfones (**12f**–**l**) (Figure 7, **B**). Indeed, not only were the yields higher under the CuClXantPhos-catalyzed conditions but we also noticed a remarkable improvement in the stereoselectivity, reaching *E*/*Z* ratios >99:1 in the case of pinacolboronic esters **13k** and **13l**, clearly surpassing the stereoselectivities obtained with the nitro ally precursors. This difference in the selectivity may be attributed to the size of the aryl sulfone moiety compared to the nitro group, which favors the formation of the *E* double bond in the elimination step.

## Conclusions

In summary, we have reported a novel reactivity for allylic nitroalkanes, which can now be used as valuable precursors for the synthesis of diversely substituted allylic boronic esters through copper-catalyzed borylation. This is the first time that a metal is used to catalyze the borylation of nitro allyl derivatives. The method is chemoselective, favoring C–NO_2_ fragmentation over NO_2_ reduction, high yielding, scalable, easy to set up, affordable, highly functional group tolerant, and applicable to the synthesis of polysubstituted allylic boronic esters, which are valuable synthetic platforms as showcased in the various post-transformations. The method is also highly versatile as the allylic nitro derivatives can be easily prepared from readily available nitroalkene precursors; a strategy best pictured in our fluorination/borylation process toward fluorinated allyl boronic esters. A comprehensive density functional theory study has helped us gather some valuable insights into the reaction mechanism and the origin of the diastereoselectivity. Interestingly, we were also able to extend the method to allyl sulfones. Indeed, the latter proved to readily undergo borylation under similar conditions, affording the corresponding allylpinacol boranes in excellent yields and also excellent stereoselectivities. The use of allyl sulfones offers an even broader substrate scope as the latter can be easily prepared and functionalized via a plethora of synthetic transformations such as alkylations, fluorinations, acylations, and Michael additions just to name a few. Most importantly, we believe this work paves the way to further developments in the field of copper-catalyzed allylations, which are currently under investigation in the group.

## Data Availability

The data underlying this study are available in the published article and its Supporting Information.^68^

## Supporting Information Available

Experimental methods, characterization data, diffraction data, NMR spectra, and DFT method (PDF)
