# Supplementary material for: Merging Copper Catalysis with Nitro Allyl and Allyl Sulfone Derivatives: Practical, Straightforward, and Scalable Synthesis of Diversely Functionalized Allyl Boranes
Source: JACS Au. 2025 Jan 8;5(1):99–110. doi: 10.1021/jacsau.4c00809 (PMC11775705; doi:10.1021/jacsau.4c00809)
Supplement: Supplementary file 1 — au4c00809_si_001.pdf [file au4c00809_si_001.pdf]

Supporting Information

Merging Copper Catalysis with Nitro Allyl and Allyl Sulfone Derivatives: Practical, Straightforward and Scalable Synthesis of Diversely Functionalized Allyl Boranes†

Nicolas Fincias,[§] Louis Clavier,[§] Cora Escande de Messières,[§] Marianne Guillard,[§] Mansour Dole Kerim,[§] Nicolas Casaretto,[§] Julian Garrec,[‡] Stellios Arseniyadis,[,†] Laurent El Kaïm[*,§]*

[§]Laboratoirede Synthèse Organique (LSO-UMR76523) CNRS, Ecole Polytechnique, ENSTA-Paris, Institut Polytechnique de Paris,
828 Bd des Maréchaux, 91128 Palaiseau Cedex, France
E-mail: laurent.elkaim@ensta-paris.fr

[‡]Unité Chimie et Procédés (UCP) ENSTA-Paris, Institut Polytechnique de Paris,
828 Bd des Maréchaux, 91128 Palaiseau Cedex, France

[†]Queen Mary University of London, Department of Chemistry, Mile End Road, E1 4NS, London, UK
E-mail: s.arseniyadis@qmul.ac.uk

Table of content

1. Materials and methods ***S2***
2. Experimental and Spectral data ***S3***
3. ^{1}H and ^{13}C NMR spectra copies ***S59***
4. X-Ray crystallography of lithium(tetrahydrate) bis(pinacolato)borate ***S145***
5. DFT calculations ***S150***
5.1. Computational details for the DFT study of the borylation reactions *S150*
5.2. Optimised geometries for the borylation of 3-methyl-3-nitrobutene *S150*
*5.3. NCIPlot analysis: rationalisation of the difference in energy between **Int-anti** and **Int-syn** in the borylation reaction* *S153*
5.4. Optimised geometries and additional free energy profiles for the borylation of 3-fluoro-3-nitrobutene *S154*
*5.5. Mechanism study for the scandium-catalysed lactonization leading to the formation of compound **10**, and their corresponding 3D structures* *S157*
6. Additional experiments to improve the stereoselectivity ***S159***

1. Materials and methods

All reagent-grade chemicals and other solvents were obtained from commercial suppliers and were used as received. Anhydrous solvents were either obtained from commercial sources or distilled appropriately. Petroleum ether refers to the 40-60 °C boiling fraction. Commercially available chemicals were used as purchased, or where specified, purified by standard techniques.

NMR spectra were recorded at 298 K using a Bruker AVANCE 400 spectrometer. ^{1}H NMR spectra were recorded at 400 MHz and residual solvent peaks were used as an internal reference ($CDCl_3$ δ 7.26). Data are reported as follows: chemical shift in ppm, multiplicity (s = singlet, d = doublet, t = triplet, q = quartet, p= quintet, m = multiplet or overlap of nonequivalent resonances), coupling constants, integration. ^{13}C NMR spectra were recorded at 101 MHz and residual solvent peaks were used as an internal reference ($CDCl_3$ δ 77.16). Data are reported as follows: chemical shift in ppm, multiplicity deduced from DEPT experiments (CH_3, CH_2, CH and C_q). The assignment of ^{1}H and ^{13}C signals was assisted by COSY, HSQC and HMBC experiments where necessary. **High Resolution Mass Spectra (HRMS)** were obtained on aJEOL JMS-GCmate II spectrometer (EI+) or on a Bruker tims-TOF mass spectrometer (ESI+), and reported as m/z (relative intensity). **Analytical TLC** was performed with Merck silica gel plates, pre-coated with silica gel 60 F254 (0.2 mm). Visualisation was effected by quenching of UV fluorescence (λ_{max} = 254 nm) and by staining with potassium permanganate or vanillin TLC stain solutions, followed by heating. **Flash column chromatography** employed VWR (230-400 mesh) silica gel. Reactions were conducted under a positive pressure of dry nitrogen or argon in oven-dried or flame-dried glassware, and at ambient room temperature, unless specified otherwise.

Note: for the ^{13}C spectra of allylboranes, the carbon α to the boron could not be detected.

2. Experimental and Spectral data

General sequence 1 (GS1)

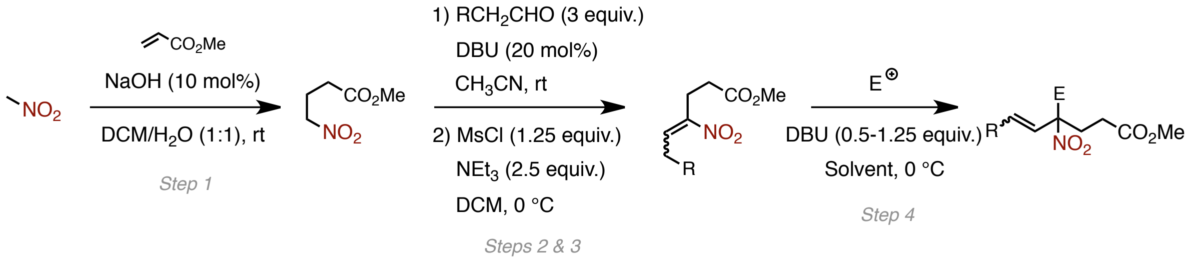

Step 1: Procedure adapted from Chasar *et al. Synthesis*, **1982**, *10*, 841-842.

A solution of NaOH (400 mg, 10 mmol) in 20 mL of water was added to a solution of methyl acrylate (9.0 mL, 100 mmol) and nitroalkane (5.4 mL, 100 mmol) in 20 mL CH_2Cl_2. The resulting biphasic mixture was agitated under strong stirring, followed by TLC until completion (approx. 10 h). The reaction mixture was quenched with 1 mL of a 1.0 M aqueous solution of HCl and transferred to a separatory funnel. The organic phase was collected and the aqueous layer was extracted with 3 x 10 mL of CH_2Cl_2. The combined organic layers were washed with a saturated aqueous solution of brine, dried over anhydrous $MgSO_4$ and concentrated under reduced pressure. The resulting dark yellow oil was diluted in Petroleum Ether/ Et_2O solution (70:30), passed through a short plug of silica to remove any polymeric side products and used in the next step without further purification (8.0 g recovered, 55% theoretical yield).

Step 2: Procedure adapted from Ono *et al. Journal of Heterocyclic Chemistry*, **1994**, *31*, 707-710.

To a solution of nitroalkane (10 mmol) and aldehyde (15 mmol) in MeCN (10 mL, 1.0 M) was added DBU (0.30 mL, 2 mmol) at 0 °C and the solution was stirred at the same temperature for 16 h until completion (reaction monitored by TLC). The reaction mixture was diluted with 50 mL of H_2O, quenched with 1 mL of a 1.0 M aqueous solution of HCl and transferred to a separatory funnel and the aqueous layer was extracted with 5 x 20 mL of Et_2O. The combined organic layers were washed with a saturated aqueous solution of brine, dried over anhydrous $MgSO_4$ and concentrated under reduced pressure. The resulting orange oil was used in the next step without further purification.

Step 3: Procedure adapted from Melton *et al. J. Org. Chem.* **1975**, *40*, 2138-2139.

To a solution of nitroalkanol in CH_2Cl_2 (20 mL, 0.5 M) was added MsCl (0.85 mL, 11 mmol) at 0 °C; the resulting solution was then stirred for 15 min before NEt_3 (3.50 mL, 25 mmol) was added dropwise over a period of 20 min at the same temperature. The solution was stirred for an additional 2 h until completion (reaction monitored by TLC). The reaction mixture was diluted with 50 mL of H_2O and 15 mL of a 1.0 M aqueous solution of HCl to quench the remaining amine. The resulting biphasic mixture was transferred in a separatory funnel. The organic layer was collected and the aqueous layer was extracted with 3 x 10 mL of CH_2Cl_2. The combined organic layers were washed with a saturated aqueous solution of brine, dried over anhydrous $MgSO_4$ and concentrated under reduced pressure.

The resulting dark orange oil was diluted in Petroleum Ether/ Et_2O solution (70:30), passed through a short plug of silica to remove any polymeric side products and used in the next step without further purification (1.2 g recovered, 69% yield theoretical).

Step 4: Michael addition and Henry reaction adapted from Ono *et al. Synthesis* **1987**, *3*, 258-260.
To a solution of nitroalkene (3.0 mmol) and the electrophile (7.5 mmol, 2.5 equiv.) in the appropriate solvent (6 mL, 0.5 M) at 0 °C was added DBU (0.20 to 1.25 equiv.). The resulting solution was stirred then for 4 h at the same temperature. The solution was eventually quenched by slow addition of 1 mL of a 1.0 M aqueous solution of HCl. The solution was transferred in a separatory funnel with 20 mL of H_2O. The organic layer was collected and the aqueous layer was extracted with 3 x 15 mL of Et_2O. The combined organic layers were washed with a saturated aqueous solution of brine, dried over anhydrous $MgSO_4$ and concentrated under reduced pressure. The crude residue was purified by flash column chromatography over silica gel using PE/EtOAc as eluent.

Step 4: Fluorination reaction adapted from Peng *et al Tetrahedron Lett.* **2005***, 46,* 4905-4909.
A solution of nitroalkene (4 mmol, 1.0 equiv.) and Selectfluor (5.0 mmol, 1.2 equiv.) in CH_2Cl_2 (20 mL, 0.2 M) was stirred at 0 °C for 15 min. DBU (1.25 equiv.) was then added dropwise at the same temperature and the reaction mixture was stirred until completion (approx. 4 h, reaction monitored by TLC). Water was then added together with 6 mL of a 1.0 M aqueous solution of HCl. The biphasic mixture was transferred to a separatory funnel. The organic layer was collected and the aqueous layer was extracted with 3 x 10 mL of CH_2Cl_2. The combined organic layers were washed with a saturated aqueous solution of brine, dried over anhydrous $MgSO_4$ and concentrated under reduced pressure. The crude residue was purified by flash column chromatography over silica gel using PE/EtOAc as eluent.

General sequence 2 (GS2)

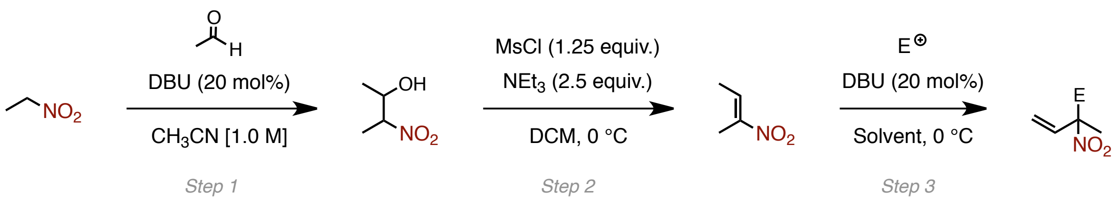

Step 1: Procedure adapted from Ono *et al. Journal of Heterocyclic Chemistry*, **1994**, *31*, 707-710.
To a solution of nitroalkane (10 mmol) and acetaldehyde (20 mmol) in MeCN (10 mL, 1.0 M) was added DBU (0.30 mL, 2 mmol) at 0 °C and the solution was stirred at the same temperature for 16 h until completion (reaction monitored by TLC). The solution was then diluted with 50 mL of H_2O, quenched with 1 mL of a 1.0 M aqueous solution of HCl, and transferred to a separatory funnel. The aqueous layer was extracted with 5 x 20 mL of Et_2O and the combined organic layers were washed

with a saturated aqueous solution of brine, dried over anhydrous $MgSO_4$ and concentrated under reduced pressure. The resulting orange oil was used in the next step without further purification.

Step 2: Procedure adapted from Melton *et al. J. Org. Chem.* **1975**, *40*, 2138-2139.
To a solution of nitroalkanol in CH_2Cl_2 (20 mL, 0.5 M) was added MsCl (0.85 mL, 11 mmol) at 0 °C. The resulting solution was stirred for 15 min at the same temperature before NEt_3 (3.50 mL, 25 mmol) was added dropwise over a period of 20 min. The reaction mixture was then stirred for 2 h until completion (reaction monitored by TLC). The solution was then diluted with 50 mL of H_2O and 15 mL of a 1.0 M aqueous solution of HCl to quench the remaining amine. The resulting biphasic solution was transferred in a separatory funnel. The organic layer was collected and the aqueous layer was extracted with 3 x 10 mL of CH_2Cl_2. The combined organic layers were washed with a saturated aqueous solution of brine, dried over anhydrous $MgSO_4$ and concentrated under reduced pressure. The resulting dark orange oil was diluted in Petroleum Ether/ Et_2O solution (70:30), passed through a short plug of silica to remove any side products and was used without further purification in the next step (1.2 g recovered, 69% yield theoretical).

Step 3: Procedure adapted from Ono *et al. Synthesis* **1987**, *3*, 258-260
To a solution of nitroalkene (3.0 mmol) and the electrophile (7.5 mmol, 2.5 equiv.) in the appropriate solvent (6 mL, 0.5 M) at 0 °C was added DBU (0.20 to 1.25 equiv.). The resulting solution was then stirred for 4 h at the same temperature. The reaction mixture was eventually quenched by a slow addition of 1 mL of a 1.0 M aqueous solution of HCl. The solution was transferred in a separatory funnel with 20 mL of H_2O. The organic layer was collected and the aqueous layer was extracted with 3 x 15 mL of Et_2O. The combined organic layers were washed with a saturated aqueous solution of brine, dried over anhydrous $MgSO_4$ and concentrated under reduced pressure. The crude residue was eventually purified by flash column chromatography over silica gel using PE/EtOAc as eluent.

General sequence 3 (GS3)

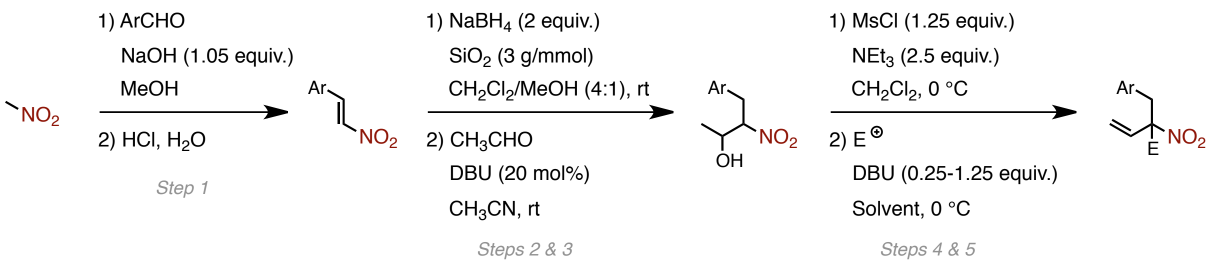

Step 1: Procedure adapted from *Org. Synth.* **1929**, *9*, 66.
In a 250 mL round bottom flask was added nitromethane (10.8 mL, 200 mmol, 1.01 equiv.), aldehyde (200 mmol, 1.0 equiv.) and 40 mL of MeOH. The reaction mixture was cooled at –10 °C before a solution of sodium hydroxide, prepared by dissolving NaOH (8.4 g, 210 mmol, 1.05 equiv.) in 20 mL of ice-cold water, was added dropwise for a period of 30 min. A white precipitate forms rapidly during the addition of the alkali. The mixture gets so thick that stirring becomes difficult and it may be advisable to

add 10 mL MeOH. After 15 min, the pasty mass is converted to a clear solution by the addition of 100-150 mL of water containing crushed ice. The resulting clear solution was then slowly added onto an aqueous solution of HCl made by diluting 40 mL of conc. HCl with 60 mL of H_2O. The addition is made at such a rate that the stream just fails to break into drops. A pale-yellow crystalline mass separates almost immediately as the alkaline solution comes in contact with the acid. The resulting solution is then filtered and the pale-yellow solid is dried *in vacuo*. The resulting nitrostyrenes were used in the next step without further purification.

Step 2: Procedure adapted from Mahesh *et al. Angew. Chem. Int. Ed.* **2020**, *59*, 2793-2801.
To a vigorously stirred mixture of nitrostyrene (50 mmol) in CH_2Cl_2 and MeOH (4:1, 50 mL) was added silica gel (3g/mmol) at rt followed by $NaBH_4$ (3.78 g, 100 mmol, 2.0 eq.) portion wise. The reaction mixture was stirred at rt until completion (approx. 2 h, reaction monitored by TLC). The reaction mixture was then quenched with a 1M aqueous solution of HCl and filtered. The silica was washed with CH_2Cl_2 (4 x 50 mL). The aqueous layer was extracted with 3 x 15 mL CH_2Cl_2. The combined organic layers were washed with a saturated aqueous solution of brine, dried over anhydrous $MgSO_4$ and concentrated under reduced pressure. The resulting yellow oil was used in the next step without further purification.

Step 3: Procedure adapted from Ono *et al. J. Heterocyclic Chem.* **1994**, *31*, 707-710.
To a solution of nitroalkane (10 mmol) and acetaldehyde (20 mmol) in MeCN (10 mL, 1.0 M) was added DBU (0.30 mL, 2 mmol) at 0 °C and the solution was stirred at 0 °C for 16 h until completion. The solution was diluted with 50 mL of H_2O and quenched with 1 mL of 1.0 M aqueous solution of HCl and transferred to a separatory funnel. The aqueous layer was extracted with 5 x 20 mL of Et_2O, the combined organic layers were washed with a saturated aqueous solution of brine, dried over anhydrous $MgSO_4$ and solvent was removed under vacuo. The resulting orange oil was used in next step without further purification.

Step 4: Procedure adapted from Melton *et al. J. Org. Chem.* **1975**, *40*, 2138-2139.
To a solution of nitroalkanol (10 mmol) in CH_2Cl_2 (20 mL, 0.5 M) at 0 °C was added MsCl (0.85 mL, 11 mmol). The resulting solution was stirred for 15 min at the same temperature before NEt_3 (3.50 mL, 25 mmol) was added dropwise over a period of 20 min. The reaction mixture was further stirred 2 h until completion (reaction monitored by TLC). The reaction mixture was then diluted with 50 mL of H_2O and 15 mL of a 1.0 M aqueous solution of HCl to quench the remaining amine. The resulting biphasic mixture was transferred in a separatory funnel. The organic layer was collected and the aqueous layer was extracted with 3 x 10 mL of CH_2Cl_2. The combined organic layers were washed with a saturated aqueous solution of brine, dried over anhydrous $MgSO_4$ and concentrated under reduced pressure. The resulting dark orange oil was diluted in Petroleum Ether/ Et_2O solution (70:30), passed through a short plug of silica to remove any polymeric side products, and used in the next step without further purification.

Step 5: Michael addition and Henry reaction adapted from Ono *et al. Synthesis* **1987**, *3*, 258-260.

To a solution of nitroalkene (3.0 mmol) and the electrophile (7.5 mmol, 2.5 equiv.) in MeCN (6 mL, 0.5 M) at 0 °C was added DBU (0.20 to 1.25 equiv.). The reaction mixture was then stirred for 4 h at the same temperature. The solution was quenched by a slow addition of 1 mL of a 1.0 M aqueous solution of HCl. The mixture was transferred in a separatory funnel with 20 mL of H_2O. The organic layer was collected and the aqueous layer was extracted with 3 x 15 mL of Et_2O. The combined organic layers were washed with a saturated aqueous solution of brine, dried over anhydrous $MgSO_4$ and concentrated under reduced pressure. The crude residue was purified by flash column chromatography over silica gel using PE/EtOAc as eluent.

Step 5: Fluorination reaction adapted from Peng *et al. Tetrahedron Lett.* **2005**, *46,* 4905-4909.

A solution of nitroalkene (4 mmol, 1.0 equiv.) and Selectfluor (5.0 mmol, 1.2 equiv.) in CH_2Cl_2 (20 mL, 0.2 M) was stirred at 0 °C for 15 min. DBU (1.25 equiv.) was then added dropwise at the same temperature and the solution was stirred until completion (approx. 4 h, reaction monitored by TLC). Once the reaction was complete, water was added together with 6 mL of a 1.0 M aqueous solution of HCl. The biphasic mixture was transferred to a separatory funnel. The organic layer was collected and the aqueous layer was extracted with 3 x 10 mL of CH_2Cl_2. The combined organic layers were washed with a saturated aqueous solution of brine, dried over anhydrous $MgSO_4$ and concentrated under reduced pressure. The crude residue was purified by flash column chromatography over silica gel using PE/EtOAc as eluent.

Note: in the case of benzylic substituents bearing a strong electron-withdrawing group (CN or CF_3), the reaction must be monitored by TLC and quenched upon completion (approx. 30 min) to avoid degradation of the fluoro allylic nitro compound.

General sequence 4 (GS4)

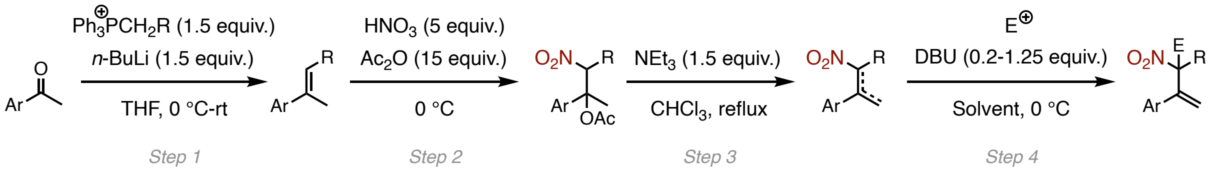

Step 1: Procedure adapted from Martin *et al. J. Am. Chem. Soc.* 2007, *129*, 29, 8976–8977

To a solution of phosphonium bromide (1.5 equiv) in THF at 0 °C was added *n*-BuLi (1.5 equiv). After stirring the reaction mixture for 4 h at the same temperature, the ketone (1.0 equiv) was added dropwise. The reaction mixture was then stirred overnight, filtered, poured into water and extracted three times with Et_2O. The combined organic layers were dried over anhydrous $MgSO_4$, concentrated under reduced pressure. The crude alkene was passed through a small pad of silica using pentane a the eluent and used without further purification in the next step.

Step 2: Procedure adapted from Martin *et al. J. Am. Chem. Soc.* **2007**, *129*, 8976-8977.
In a two-neck round-bottomed flask was added acetic anhydride (28.5 mL, 300 mmol, 15.0 equiv.). The flask was cooled to −10 °C and 69% nitric acid (4.9 mL, 75 mmol, 5.0 equiv.) was slowly added. The reaction mixture was allowed to warm to 0 ºC over 3 h. (CAUTION: a fast addition of the nitric acid can result in a significant increase of the temperature generating high amounts of nitrogen oxide which can potentially lead to explosions). The alkene (20 mmol, 1.0 equiv.) was then added dropwise to the solution and the reaction mixture was stirred for 3 h at 0 ºC and quenched at low temperature by the addition of water (300 mL). The resulting mixture was stirred for 1 h. The two phases were separated and the aqueous phase extracted with Et_2O (3x 25 mL). The combined organic phases were washed with an aqueous solution of $NaHCO_3$ and water, dried over anhydrous $MgSO_4$. The volatile compounds were removed under reduced pressure and the crude product was used in the next step without further purification.

Step 3: Procedure adapted from Martin *et al. J. Am. Chem. Soc.* **2007**, *129*, 8976-8977.
To a solution of the nitro acetate in $CHCl_3$ (100 mL) was added triethylamine (4.2 mL, 30 mmol, 1.5 equiv.). The reaction was stirred at reflux until completion (approx. 6 h, up to 16 h for hindered substrates, reaction monitored by TLC). After completion, water was added together with 50 mL of a 0.10 M aqueous solution of HCl. The biphasic mixture was transferred to a separatory funnel. The organic layer was collected and the aqueous layer was extracted with 3 x 20 mL of Ch_2Cl_2. The combined organic layers were washed with a saturated aqueous solution of brine, dried over anhydrous $MgSO_4$ and concentrated under reduced pressure. The crude residue was passed through a small pad of silica to remove any traces of polymeric residue using a 5% Et_2O in PE solution. The crude residue was used in the next step without further purification.

Step 4: Michael addition and Henry reaction adapted from Ono *et al. Synthesis* **1987**, *3*, 258-260.
To a solution of nitroalkene (3.0 mmol) and electrophile (7.5 mmol, 2.5 equiv.) in the appropriate solvent (6 mL, 0.5 M), is added DBU (0.20 to 1.25 equiv.) at 0 °C; the resulting solution is stirred for 4 h at 0 °C. The solution is quenched by a slow addition of 1 mL of a 0.10 M aqueous solution of HCl. The solution is transferred in a separatory funnel with 20 mL of H_2O. The organic layer is collected, and the aqueous layer is extracted with 3 x 15 mL of Et_2O. The combined organic layers are washed with a saturated aqueous solution of brine, dried over anhydrous $MgSO_4$ and the solvent is removed *in vacuo*. The crude residue is purified by flash column chromatography over silica gel using PE/EtOAc as eluent.

Step 4: Fluorination reaction adapted from Peng *et al Tetrahedron Lett.* **2005***, 46,* 4905-4909.
A solution of nitroalkene (4 mmol, 1.0 equiv.) and Selectfluor (5.0 mmol, 1.2 equiv.) in CH_2Cl_2 (20 mL, 0.2 M) was stirred at 0 °C for 15 min. DBU (1.25 equiv.) was then added dropwise at 0 °C and the solution was stirred until completion (approx. 4 h). After completion, water is added together with 6 mL of 0.10 M aqueous solution of HCl. The biphasic mixture is transferred to a separatory funnel, the organic layer is collected, and the aqueous layer is extracted with 3 x 10 mL of CH_2Cl_2. The combined organic layers were washed with a saturated aqueous solution of brine dried over anhydrous $MgSO_4$

and concentrated in vacuo. The crude residue is purified by flash column chromatography over silica gel using PE/EtOAc as eluent.

General sequence 5 (GS5)

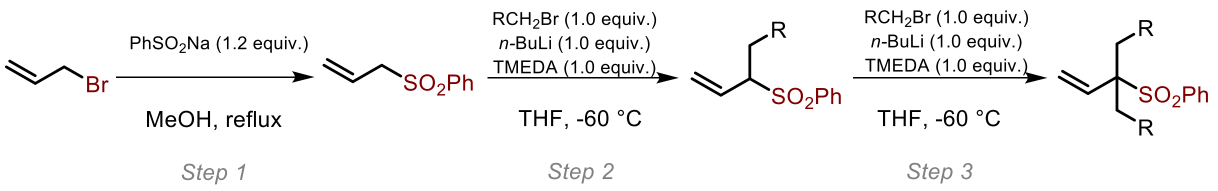

Step 1: Procedure adapted from Uno *et al. J. Org. Chem*. **2019**, *84*, 9330-9338.

To a solution of $PhSO_2Na$ (1.97 g, 12 mmol, 1.2 equiv.) in MeOH (30 mL, 0.3 M) was added allylbromide (0.87 mL, 10 mmol, 1.0 equiv.). The reaction mixture was heated to reflux for 2 h. The solvent was evaporated under reduced pressure. The mixture was then dissolved in 50 mL of Et_2O, washed with a saturated aqueous solution of brine, dried over anhydrous $MgSO_4$ and concentrated under reduced pressure. The resulting transparent oil was used in the next step without further purification (1.8 g, 99%).

Steps 2 and 3: Procedures adapted from Savoia *et al. J. Chem. Soc., Perkin Trans. 1* **1977**, 123-125.

In a dry round bottom flask equipped with a magnetic stirrer and a septum under argon was added the allylic sulfone derivative (2.7 mmol, 1.0 equiv.) and TMEDA (0.41 mL, 2.7 mmol, 1.0 equiv.) in THF (5.4 mL, 0.5 M). A titrated solution of *n*-Buli in hexane (1.5 mL, 1.88 M, 2.7 mmol, 1.0 equiv.) was added dropwise at –60 °C. After stirring for 30 min, a solution of benzylbromide in THF (508 mg, 2.7 mmol, 2.5 M, 1.0 equiv.) was added dropwise at –60 °C. The reaction mixture was stirred at –60 °C then allowed to warm up to 0 °C. It was finally quenched by pouring into ice water and transferred into a separatory funnel. The organic layer was extracted with Et_2O (3 x 20 mL). The combined organic layers were washed with a saturated aqueous solution of brine, dried over anhydrous $MgSO_4$ and concentrated under reduced pressure. The resulting oil was purified by flash column chromatography over silica gel.

General procedure for the synthesis of Allylboranes (GP-A)

To a solution of the allylic nitroalkane (0.50 mmol, 1.00 equiv.) in MeOH (0.50 M) was added CuCN (10 mol%) and B_2Pin_2 (2.00 equiv.). The reaction mixture was stirred for 5 min before MeOLi (2.00 equiv.) was added in one portion. The reaction was stirred for 3 h at rt and quenched with an aqueous solution of HCl (0.01 M). The aqueous layer was extracted with 3 x 10 mL of EtOAC. The combined organic layer was washed with a saturated aqueous solution of brine, dried over anhydrous $MgSO_4$ and concentrated under reduced pressure. The crude residue was purified by flash column chromatography over borylated silica gel (Hitosugi *et al. Chem. Lett.* **2012**, *41*, 972973) affording the desired allylborane.

General procedure for the $Sc(OTf)_3$ catalyzed allylation of aldehydes (GP-B):

To a solution of allylboronate (0.22 mmol, 1.0 equiv.) in toluene (0.3 mL, 1 M) at 0 °C was added *p*-chlorobenzaldehyde (0.27 mmol, 1.2 equiv.) followed by scandium triflate (0.02 mmol, 0.1 equiv.), and the reaction mixture was stirred for 24 h at rt or 40 h at 40 °C in the case of highly substituted allylboronate. The reaction was then quenched with water (3 mL) and the aqueous layer was extracted with 3 x 10 mL of Et_2O. The combined organic layer was washed with a saturated aqueous solution of brine, dried over anhydrous $MgSO_4$ and concentrated under reduced pressure. The crude residue was eventually purified by flash column chromatography over silica gel.

General procedure for the allylation of aldehydes with fluorinated allylboranes (GP-C):

Procedure adapted from Akiyama *et al.*, *Angew. Chem. Int. Ed.* **2019**, *58*, 11998–12003.

In an oven-dried reaction vial, under argon atmosphere, a solution of allylboronate (0.33 mmol, 1.0 equiv.) in THF (3.3 mL, 0.1 M) was treated with *n*-BuLi in hexane (1.9 M, 0.36 mmol, 1.1 equiv.), added dropwise at –78 °C. The solution was allowed to stir for 15 min. Trifluoroacetic anhydride (0.39 mmol, 1.2 equiv.) was added dropwise to the mixture and the reaction was allowed to stir for 30 min at –78 °C. Aldehyde (0.49 mmol, 1.5 equiv.) was then added at –78 °C and the mixture was allowed to stir for 2h and to slowly warm up to room temperature. The reaction was then quenched with a saturated aqueous solution of $NaHCO_3$ (3 mL). The aqueous layer was extracted with 3 x 10 mL Et_2O, washed with a saturated aqueous solution of brine, dried over anhydrous $MgSO_4$ and solvent was removed in vacuo. The crude residue was purified by flash column chromatography affording the desired product.

General procedure for allylation of paraformaldehyde (GP-D):

Procedure adapted from Ozawa *et al.*, *J. Am. Chem. Soc.* **2021**, *143*, 13865-13877.

To a solution of allylboronate (0.29 mmol, 1.0 equiv.) in THF (1.5 mL, 0.2 M) under an argon atmosphere was added paraformaldehyde (1.5 mmol, 5.0 equiv.). The reaction was stirred for 17 h at 65 °C with a reflux apparatus. After full conversion of the starting material (reaction monitored by TLC), PTSA was added (0.03 mmol, 0.1 equiv.) and the reaction was allowed to stir for an additional 7 h at the same temperature to allow complete lactonisation of the allylated product. The reaction was then quenched with water (3 mL). The aqueous layer was extracted with 3 x 10 mL Et_2O and the combined organic layer was washed with a saturated aqueous solution of brine, dried over anhydrous $MgSO_4$ and concentrated under reduced pressure. The crude residue was ultimately purified by flash column chromatography over silica gel to afford the desired lactone.

General procedure for the α-oxydation of allylboronate (GP-E):

Procedure adapted from Smith *et al.*, *J. Am. Chem. Soc.* **2017**, *139*, 7721-7724.

A solution of allylboronate (0.22 mmol, 1.0 equiv.) in THF (1.5 mL) under argon atmosphere was treated with sodium perborate monohydrate (0.67 mmol, 3.0 equiv.), which was added in one portion. H_2O (1.5 mL) was then added and the mixture was allowed to stir for 2 h at rt. The reaction was then quenched with a saturated aqueous solution of $Na_2S_2O_3$ (3 mL). The aqueous layer was extracted with

3 x 10 mL Et_2O, and the combined organic layer was washed with a saturated aqueous solution of brine, dried over anhydrous $MgSO_4$ and concentrated under reduced pressure. The crude residue was purified by flash column chromatography over silica gel to afford the desired product.

General procedure for the γ-oxydation of allylboronate (GP-F):

To a solution of allylboronate (0.16 mmol, 1.0 equiv.) in toluene (0.2 mL, 1 M) at 0 °C was added pivalaldehyde (0.19 mmol, 1.2 equiv.) folowed by scandium triflate (0.02 mmol, 0.1 equiv.) and the reaction mixture was stirred for 64 h at 40 °C. The reaction was then quenched with water (3 mL). The aqueous layer was extracted with 3 x 10 mL Et_2O and the combined organic layer was washed with a saturated aqueous solution of brine, dried over anhydrous $MgSO_4$ and concentrated under reduced pressure. The crude residue was purified by flash column chromatography over silica gel to afford the desired product.

General procedure for the Suzuki/dehydrofluorination cascade (GP-G):

To a solution of allylboronate (0.33 mmol, 1.0 equiv.) in dioxane (3 mL) under an argon atmosphere was added $Pd(OAc)_2$ (0.02 mmol, 0.05 equiv) and $[PPh^tBu_2H]BF_4$ (0.05 mmol, 0.15 equiv.) followed by water (0.3 mL) and Cs_2CO_3 (0.49 mmol, 1.5 equiv.). The reaction mixture was then allowed to stir for 10 min before *p*-methoxybenzylbromide (0.39 mmol, 1.2 equiv.) was added, and the reaction was allowed to stirre at 80 °C overnight. The reaction was then quenched with water (3 mL). The aqueous layer was extracted with 3 x 10 mL of Et_2O. The combined organic layer was washed with a saturated aqueous solution of brine, dried over anhydrous $MgSO_4$ and concentrated under reduced pressure. The crude residue was purified by flash column chromatography affording the desired product.

General procedure for the synthesis of allylboranes (GP-H):

To a solution of the allylic sulfone (0.46 mmol, 1.0 equiv.) in MeOH (4.6 mL, 0.1 M) were added CuCN (4.1 mg, 0.046 mmol, 0.1 equiv.) and B_2Pin_2 (363 mg, 1.43 mmol, 3.1 equiv.). The reaction mixture was stirred for 5 min before *t*-BuOK (155 mg, 1.38 mmol, 3.0 equiv.) was added in one portion. The reactionwas stirred for 2 h at rt and quenched with H_2O. The organic layer was extracted with Et_2O (3 x 10 mL). The combined organic layers were washed with a saturated aqueous solution of brine, dried over anhydrous $MgSO_4$ and concentrated under reduced pressure. The crude residue was purified on a pad of borylated silica gel (Hitosugi *et al. Chem. Lett.* **2012**, *41*, 972973) affording the desired allylboronate (92%, 160 mg, 0.425 mmol).

Entry[a]	Catalyst	Base	Solvent	Conversion	NMR Yield
1	CuI	*t*-BuOK	THF	11%	6%
2	CuCN·2LiCl	*t*-BuOK	THF	16%	15%
3	$Cu(OAc)_2$	*t*-BuOK	THF	27%	26%
4	CuClXantPhos	*t*-BuOK	THF	84%	84%
5	CuCN	*t*-BuOK	THF	34%	34%
**6**	**CuCN**	***t*-BuOK**	**MeOH**	**86%**	**86%**
7	CuCN	MeOLi	MeOH	73%	71%
8	CuCN	Cs_2CO_3	MeOH	56%	48%
9	CuCN	MeOK	MeOH	77%	73%
10	CuCN	*t*-BuOK	DCM	<5%	traces
11	$Fe(acac)_3$	*t*-BuOK	THF	<5%	traces

[a]All reactions were run on a 0.5 mmol scale (0.1 M concentration), catalyst (23 mol%), B_2pin_2 (1.5 equiv.), base (1.5 equiv.).

Entry[a]	CuCN (equiv.)	Base (equiv.)	B_2pin_2 (equiv.)	Conversion	NMR Yield
1	0.1	1.5	1.5	94%	93%
2	0.1	1.5	3.0	96%	94%
3	0.1	3.5	3.0	87%	86%
4	0.1	3.0	3.5	99%	99%
**5**	**0.1**	**3.0**	**3.1**	**99%**	**99%**

[a]All reactions were run on a 0.5 mmol scale (0.1 M concentration).

General procedure for the synthesis of allylboranes (GP-I):

To a solution of the allylic sulfone (0.66 mmol, 1.0 equiv.) in THF (6.6 mL, 0.1 M) were added CuClXantPhos (44 mg, 0.066 mmol, 0.1 equiv.) and B_2Pin_2 (522 mg, 2.06 mmol, 3.1 equiv.). The reaction mixture was stirred for 5 min before *t*-BuOK (223 mg, 1.99 mmol, 3.0 equiv.) was added in one portion. The reaction was stirred for 2 h at rt and quenched with H_2O. The organic layer was extracted with Et_2O (3 x 10 mL). The combined organic layers were washed with a saturated aqueous solution of brine, dried over anhydrous $MgSO_4$ and concentrated under reduced pressure. The crude residue was purified on a pad of borylated silica gel (Hitosugi *et al. Chem. Lett.* **2012**, *41*, 972973) affording the desired allylboronate (90%, 190 mg, 0.597 mmol).

Entry[a]	CuClXantPhos (equiv.)	Base	Conditions	Conversion	NMR Yield
1	0.5	*t*-BuONa	0.25 M, rt, 2 h	62%	62%
2	0.5	*t*-BuONa	0.25 M, rt, 17 h	66%	66%
3	0.5	*t*-BuONa	0.1 M, 50 °C, 2 h	86%	58%
4	0.23	*t*-BuONa	0.1 M, rt, 2 h	72%	72%
5	0.23	*t*-BuOK	0.1 M, rt, 2 h	84%	84%
6	0.23	*t*-BuOLi	0.1 M, rt, 2 h	82%	79%
7	0.23	Cs2CO3	0.1 M, rt, 2 h	56%	48%
8	0.1	*t*-BuOK	0.1 M, rt, 2 h	84%	83%
**9[b]**	**0.1**	***t*-BuOK**	**0.1 M, rt, 2 h**	**100%**	**94%**

[a]All reactions were run on a 0.5 mmol scale (0.1 M concentration), B_2pin_2 (1.5 equiv.), base (1.5 equiv.) unless otherwise specified. [b]B_2pin_2 (3.1 equiv.), base (3.0 equiv.).

Methyl 4-nitro-4-vinylheptanedioate (1a)

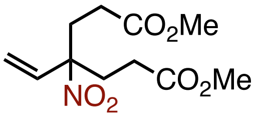

MW (g/mol): 259.26 **Molecular formula: $C_{11}H_{17}NO_6$**

Prepared following general sequence **GS1**. Purification by flash column chromatography over silica gel (PE/EtOAc 95:05 to 80:20) afforded **1a** as a pale-yellow oil in 57% yield (5.2 g, 20.1 mmol).

^{1}H NMR (400 MHz, $CDCl_3$) δ 6.19 (dd, J = 17.7, 11.2 Hz, 1H), 5.46 (d, J = 11.2 Hz, 1H), 5.31 (d, J = 17.7 Hz, 1H), 3.67 (s, 6H), 2.49-2.23 (m, 8H).

^{13}C NMR (101 MHz, $CDCl_3$) δ 172.4, 134.1, 118.6, 93.3, 52.0, 32.3, 28.8.

IR (neat) cm^{-1}: 2953 (w), 1732 (s), 1539 (s), 1436 (m), 1364 (m), 1350 (m), 1195 (s), 1172 (s), 986 (w), 891 (w), 840 (w), 801 (w).

HRMS *m/z* (ESI+) calcd for $[C_{11}H_{17}NNaO_6]^+$ = 282.0948, found: 282.0936.

Methyl 4-methyl-4-nitrohex-5-enoate (1b)

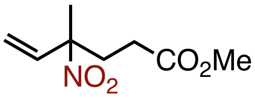

MW (g/mol): 187.19 **Molecular formula: $C_8H_{13}NO_4$**

Prepared following general sequence **GS2**. Purification by flash column chromatography over silica gel (PE/EtOAc 95:05 to 90:10) afforded **1b** as a colorless oil in 47% yield (1.06 g, 5.64 mmol).

^{1}H NMR (400 MHz, $CDCl_3$) δ 6.18 (dd, J = 17.4, 10.9 Hz, 1H), 5.38 (d, J = 10.9 Hz, 1H), 5.35 (d, J = 17.4 Hz, 1H), 3.68 (s, 3H), 2.60-2.08 (m, 4H), 1.68 (s, 3H).

^{13}C NMR (101 MHz, $CDCl_3$) δ 172.5, 136.6, 90.5, 52.0, 34.2, 29.1, 22.0.

IR (neat) cm^{-1}: 2954 (w), 1736 (s), 1539 (s), 1438 (m), 1418 (w), 1384 (w), 1346 (w), 1200 (m), 1175 (m), 991 (w), 941 (w).

HRMS *m/z* (ESI+) calcd for $[C_8H_{13}O_2]^+$ = 141.0910, found: 141.0910.

4-Methyl-4-nitrohex-5-enenitrile (1c)

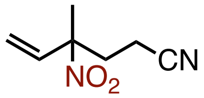

MW (g/mol): 154.17 **Molecular formula: $C_7H_{10}N_2O_2$**

Prepared following general sequence **GS2**. Purification by flash column chromatography over silica gel (PE/EtOAc 90:10 to 75:25) afforded **1c** as a pale yellow oil in 47% yield (217 mg, 1.41 mmol).

^{1}H NMR (400 MHz, CDCl3) δ 6.15 (ddd, *J* = 17.3, 10.9, 1.7 Hz, 1H), 5.47 (dd, *J* = 10.9, 1.8 Hz, 1H), 5.40 (dd, *J* = 17.4, 1.7 Hz, 1H), 2.51-2.33 (m, 4H), 1.75 (s, 3H).

^{13}C NMR (101 MHz, $CDCl_3$) δ 135.5, 119.1, 118.3, 89.9, 34.7, 22.2, 12.9.

IR (neat) cm^{-1}: 2997 (w), 2250 (w), 1642 (w), 1538 (s), 1418 (m), 1386 (m), 1345 (m), 1100 (w), 994 (w), 944 (w), 857 (w).

HRMS *m/z* (ESI+) calcd for $[C_7H_{10}N_2NaO_2]^+$ = 177.0634, found: 177.0622.

[(3-Methyl-3-nitropent-4-en-1-yl)sulfonyl]benzene (1d)

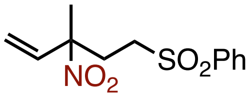

MW (g/mol): 269.32 **Molecular formula: $C_{12}H_{15}NO_4S$**

Prepared following general sequence **GS2**. Purification by flash column chromatography over silica gel (PE/EtOAc 95:05 to 85:15) afforded **1d** as a white gum in 61% yield (493 mg, 1.83 mmol).

^{1}H NMR (400 MHz, $CDCl_3$) δ 7.94-7.87 (m, 2H), 7.75-7.66 (m, 1H), 7.65-7.54 (m, 2H), 6.09 (dd, *J* = 17.4, 10.9 Hz, 1H), 5.41 (d, *J* = 10.9 Hz, 1H), 5.33 (d, *J* = 17.4 Hz, 1H), 3.17-3.00 (m, 2H), 2.52-2.35 (m, 2H), 1.68 (s, 3H).

^{13}C NMR (101 MHz, $CDCl_3$) δ 138.4, 135.6, 134.2, 129.5, 128.0, 118.7, 89.5, 51.6, 31.8, 22.8.

IR (neat) cm^{-1}: 3066 (w), 1907 (w), 1538 (s), 1446 (m), 1304 (s), 1290 (s), 1144 (s), 1085 (m), 943 (m), 793 (m), 741 (m).

HRMS *m/z* (ESI+) calcd for $[C_{12}H_{15}NNaO_4S]^+$ = 292.0614, found: 292.0610.

tert-Butyldimethyl[(2-methyl-2-nitrobut-3-en-1-yl)oxy]silane (1e)

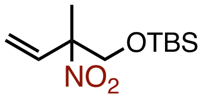

MW (g/mol): 245.39 **Molecular formula: $C_{11}H_{23}NO_3Si$**

Prepared following general sequence **GS2**. Purification by flash column chromatography over silica gel (PE/Et_2O 95:05 to 85:15) afforded **1e** as a colorless oil in 49% yield (361 mg, 1.47 mmol).

^{1}H NMR (400 MHz, $CDCl_3$) δ 6.17 (dd, *J* = 17.5, 10.9 Hz, 1H), 5.39 (d, *J* = 17.5 Hz, 1H), 5.38 (d, *J* = 11.0 Hz, 1H), 4.10 (d, *J* = 10.4 Hz, 1H), 3.76 (d, *J* = 10.4 Hz, 1H), 1.69 (s, 3H), 0.86 (s, 9H), 0.05 (s, 3H), 0.04 (s, 3H).

^{13}C NMR (101 MHz, $CDCl_3$) δ 134.9, 118.5, 92.0, 68.8, 25.7, 18.7, 18.2, –5.6, –5.6.

IR (neat) cm^{-1}: 2953 (w), 2931 (w), 2858 (w), 1546 (s), 1463 (s), 1254 (m), 1109 (s), 938 (w), 835 (s), 777 (s), 670 (w).

HRMS *m/z* (ESI+) calcd for $[C_{11}H_{23}LiNO_3Si+]^+$ = 252.1602, found: 252.1607.

Methyl 4-(4-methoxybenzyl)-4-nitrohex-5-enoate (1f)

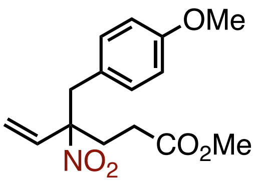

MW (g/mol): 293.32 **Molecular formula: $C_{15}H_{19}NO_5$**

Prepared following general sequence **GS3**. Purification by flash column chromatography over silica gel (PE/EtOAc 90:10 to 70:30) afforded **1f** as a colorless oil in 41% yield (361 mg, 1.23 mmol).

^{1}H NMR (400 MHz, $CDCl_3$) δ 7.00 (d, *J* = 8.7 Hz, 2H), 6.80 (d, *J* = 8.7 Hz, 2H), 6.16 (dd, *J* = 17.8, 11.3 Hz, 1H), 3.78 (s, 3H), 3.67 (s, 3H), 3.29 (d, *J* = 14.1 Hz, 1H), 3.22 (d, *J* = 14.1 Hz, 1H), 2.54-2.27 (m, 4H).

^{13}C NMR (101 MHz, $CDCl_3$) δ 172.8, 159.2, 134.8, 131.4, 125.6, 118.0, 114.0, 95.0, 55.3, 52.04, 45.2, 30.7, 29.0.

IR (neat) cm^{-1}: 2953 (w), 1737 (m), 1612 (w), 1540 (s), 1514 (m), 1438 (w), 1303 (w), 1252 (m), 1179 (m), 1034 (w), 939 (w), 840 (w).

HRMS *m/z* (ESI+) calcd for $C_{15}H_{19}NNaO_5^+$ [M$^+$]: 316.1155 ; Found: 316.1156.

Methyl 4-nitro-7-oxo-4-vinyloctanoate (1g)

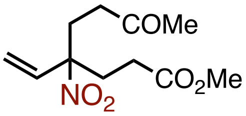

MW (g/mol): 243.26 **Molecular formula: $C_{11}H_{17}NO_5$**

Prepared following general sequence **GS1**. Purification by flash column chromatography over silica gel (PE/EtOAc 95:5 to 80:20) afforded **1g** as a colorless oil in 50% yield (161 mg, 0.66 mmol).

^{1}H NMR (400 MHz, $CDCl_3$) δ

6.19 (dd, *J* = 17.7, 11.2 Hz, 1H), 5.44 (d, *J* = 11.2 Hz, 1H), 5.28 (d, *J* = 17.7 Hz, 1H), 3.67 (s, 3H), 2.47-2.42 (m, 3H), 2.37-2.29 (m, 5H), 2.14 (s, 3H).

^{13}C NMR (101 MHz, $CDCl_3$) δ 206.3, 172.6, 134.4, 118.5, 93.6, 52.1, 38.0, 32.7, 30.8, 30.2, 28.9.

IR (neat) cm^{-1}: 1735, 1716, 1537, 1437, 1350, 1170, 941.

HRMS *m/z* (ESI+) calcd for $[C_{11}H_{18}NO_5]^+$ = 244.1179, found: 244.1178.

[(2-Benzyl-2-nitrobut-3-en-1-yl)oxy](*tert*-butyl)dimethylsilane (1h)

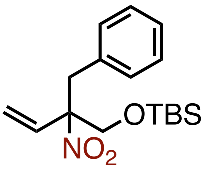

MW (g/mol): 321.49 **Molecular formula: $C_{17}H_{27}NO_3Si$**

Prepared following general sequence **GS3**. Purification by flash column chromatography over silica gel (PE/EtOAc 100:00 to 90:10) afforded **1h** as a colorless oil in 47% yield (455 mg, 1.42 mmol).

^{1}H NMR (400 MHz, $CDCl_3$) δ 7.30-7.24 (m, 3H), 7.21 (dd, *J* = 7.4, 2.1 Hz, 2H), 5.92 (dd, *J* = 17.6, 11.1 Hz, 1H), 5.39 (d, *J* = 11.1 Hz, 1H), 5.28 (d, *J* = 17.6 Hz, 1H), 4.13 (dd, *J* = 10.8, 0.7 Hz, 1H), 3.82 (d, *J* = 10.7 Hz, 1H), 3.51 (d, *J* = 13.4 Hz, 1H), 3.39 (d, *J* = 13.4 Hz, 1H), 0.92 (s, 9H), 0.09 (s, 3H), 0.08 (s, 3H).

^{13}C NMR (101 MHz, $CDCl_3$) δ 134.5, 133.6, 130.7, 128.3, 127.3, 118.6, 95.0, 77.3, 77.0, 76.7, 63.9, 39.3, 25.7, 18.2, –5.6, –5.7.

IR (neat) cm^{-1}: 2941 (w), 2928 (w), 2855 (w), 1553 (s), 1460 (s), 1262 (m), 1110 (s), 937 (w), 831 (s), 775 (s), 688 (w).

HRMS *m/z* (ESI+) calcd for $[C_{17}H_{27}OSi]^+$ = 275.1826, found: 275.1829.

Dimethyl 4-nitro-4-(1-phenylvinyl)heptanedioate (1i)

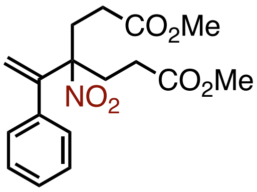

MW (g/mol): 335.35 **Molecular formula: $C_{17}H_{21}NO_6$**

Prepared following general sequence **GS4**. Purification by flash column chromatography over silica gel (PE/EtOAc 95:05 to 85:15) afforded **1i** as a pale-yellow oil in 66% yield (812 mg, 2.42 mmol).

^{1}H NMR (400 MHz, $CDCl_3$) δ 7.43-7.33 (m, 3H), 7.21-7.08 (m, 2H), 5.66 (s, 1H), 5.54 (s, 1H), 3.73 (s, 6H), 2.71-2.53 (m, 2H), 2.53-2.15 (m, 6H).

^{13}C NMR (101 MHz, $CDCl_3$) δ 172.4, 146.2, 138.3, 128.5, 128.4, 128.2, 121.1, 95.4, 52.1, 28.9, 28.7.

IR (neat) cm^{-1}: 2979 (w), 1737 (s), 1437 (w), 1341 (m), 1271 (w), 1168 (m), 1144 (m), 968 (w), 882 (w), 846 (w), 704 (w).

HRMS *m/z* (ESI+) calcd for $[C_{17}H_{21}O_4]^+$ = 289.1434, found: 289.1442.

Dimethyl 4-[1-(4-chlorophenyl)vinyl]-4-nitroheptanedioate (1j)

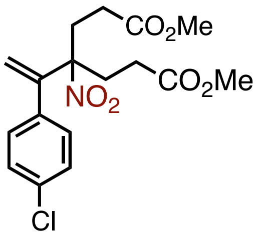

MW (g/mol): 369.80 **Molecular formula: $C_{17}H_{20}ClNO_6$**

Prepared following general sequence **GS4**. Purification by flash column chromatography over silica gel (PE/EtOAc 95:05 to 85:15) afforded **1j** as a pale-yellow gum in 61% yield (1.124 g, 3.04 mmol).

^{1}H NMR (400 MHz, $CDCl_3$) δ 7.28 (d, *J* = 8.5 Hz, 2H), 7.02 (d, *J* = 8.5 Hz, 2H), 5.63 (s, 1H), 5.48 (s, 1H), 3.69 (s, 6H), 2.59-2.50 (m, 2H), 2.41-2.21 (m, 6H).

^{13}C NMR (101 MHz, $CDCl_3$) δ 172.2, 145.1, 136.7, 134.5, 129.6, 128.7, 121.6, 95.2, 52.1, 28.8, 28.6.

IR (neat) cm^{-1}: 2955 (w), 1737 (s), 1543 (s), 1438 (m), 1346 (w), 1199 (m), 1178 (m), 1045 (w), 777 (w).

HRMS *m/z* (ESI+) calcd for $[C_{17}H_{20}ClNNaO_6]^+$ = 392.0871, found: 392.0878.

Dimethyl 4-[1-(2-fluorophenyl)vinyl]-4-nitroheptanedioate (1k)

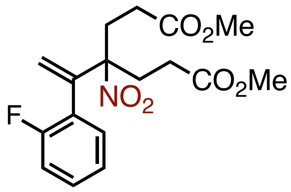

MW (g/mol): 353.34 **Molecular formula: $C_{17}H_{20}FNO_6$**

Prepared following general sequence **GS4**. Purification by flash column chromatography over silica gel (PE/EtOAc 95:05 to 85:15) afforded **1k** as a colorless oil in 53% yield (940 mg, 2.66 mmol)

^{1}H NMR (400 MHz, $CDCl_3$) δ 7.33-7.28 (m, 1H), 7.11-7.02 (m, 3H), 5.79 (s, 1H), 5.52 (s, 1H), 3.68 (s, 6H), 2.58-2.33 (m, 6H), 2.28-2.20 (m, 2H).

^{13}C NMR (101 MHz, $CDCl_3$) δ 172.4, 159.6 (d, J = 245.4 Hz), 140.1, 131.0 (d, J = 2.9 Hz), 130.3 (d, J = 8.3 Hz), 125.5 (d, J = 16.4 Hz), 124.2 (d, J = 3.6 Hz), 123.4, 115.7 (d, J = 22.9 Hz), 95.4, 52.0, 28.7 (d, J = 1.8 Hz), 28.5.

IR (neat) cm^{-1}: 2095 (m), 1733 (s), 1542 (s), 1490 (m), 1437 (m), 1344 (m), 1196 (s), 1176 (s), 987 (m), 762 (s), 498 (w), 420 (m).

HRMS *m/z* (ESI+) calcd for $[C_{17}H_{20}FNNaO_6]^+$ = 376.1167, found: 376.1181.

4-Nitro-4-(1-phenylvinyl)heptanedinitrile (1l)

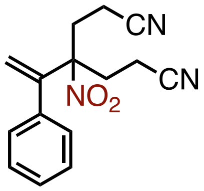

MW (g/mol): 269.30 **Molecular formula: $C_{15}H_{15}N_3O_2$**

Prepared following general sequence **GS4** Purification by flash column chromatography over silica gel (PE/EtOAc 85:15 to 60:40) afforded **1l** as a pale-yellow gum in 51% yield (545 mg, 2.02 mmol).

^{1}H NMR (400 MHz, $CDCl_3$) δ 7.40-7.35 (m, 3H), 7.12-7.00 (m, 2H), 5.59 (s, 1H), 5.55 (s, 1H), 2.67-2.33 (m, 8H).

^{13}C NMR (101 MHz, $CDCl_3$) δ 144.4, 137.0, 129.0, 129.0, 127.9, 122.0, 117.7, 94.4, 30.0, 12.8.

IR (neat) cm^{-1}: 2945 (w), 1543 (s), 1482 (w), 1446 (w), 1421 (w), 1374 (m), 1311 (w), 753 (w), 708 (s), 620 (w), 403 (w).

HRMS *m/z* (ESI+) calcd for $[C_{13}H_{14}N_2NaO_2]^+$ = 253.0947, found: 253.0948.

4-Methyl-4-nitro-5-phenylhex-5-enenitrile (1m)

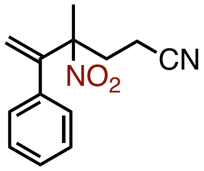

MW (g/mol): 230.26 **Molecular formula: $C_{13}H_{14}N_2O_2$**

Prepared following general sequence **GS4**. Purification by flash column chromatography over silica gel (PE/EtOAc 90:10 to 75:25) afforded **1m** as a pale yellow oil in 59% yield (543 mg, 2.36 mmol).

^{1}H NMR (400 MHz, $CDCl_3$) δ 7.38-7.31 (m, 3H), 7.11-7.04 (m, 2H), 5.58 (s, 1H), 5.45 (s, 1H), 2.72-2.62 (m, 1H), 2.46-2.33 (m, 3H), 1.81 (s, 3H).

^{13}C NMR (101 MHz, $CDCl_3$) δ 147.0, 137.9, 128.6, 128.5, 128.2, 120.1, 118.4, 92.2, 33.2, 23.2, 13.0.
IR (neat) cm^{-1}: 2951 (w), 1547 (s), 1492 (w), 1442 (w), 1423 (w), 1370 (m), 1307 (w), 751 (w), 703 (s), 624 (w), 406 (w).

HRMS *m/z* (ESI+) calcd for $[C_{13}H_{14}N_2NaO_2]^+$ = 253.0947, found: 253.0948.

Methyl 4-fluoro-4-nitrohex-5-enoate (1n)

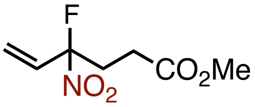

MW (g/mol): 191.16 **Molecular formula: $C_7H_{10}FNO_4$**

Prepared following general sequence **GS4**. Purification by flash column chromatography over silica gel (PE/EtOAc 100:00 to 90:10) afforded **1n** as a pale yellow oil in 37% yield (355 mg, 1.86 mmol).

^{1}H NMR (400 MHz, $CDCl_3$) δ 6.15 (ddd, *J* = 18.0, 17.2, 10.9 Hz, 1H), 5.78 (d, *J* = 17.3 Hz, 1H), 5.60 (dd, *J* = 10.9, 1.7 Hz, 1H), 3.68 (s, 3H), 2.68-2.46 (m, 3H), 2.33 (ddd, *J* = 16.4, 10.0, 6.0 Hz, 1H).

^{13}C NMR (101 MHz, $CDCl_3$) δ 171.5, 130.3 (d, *J* = 22.5 Hz), 121.9 (d, *J* = 10.3 Hz), 117.7 (d, *J* = 242.6 Hz), 52.1, 32.1 (d, *J* = 21.8 Hz), 27.1 (d, *J* = 3.0 Hz).

IR (neat) cm^{-1}: 2973 (w), 1739 (s), 1567 (s), 1439 (m), 1416 (m), 1357 (w), 1203 (m), 1178 (m), 982 (w), 957 (w), 836 (w).

HRMS *m/z* (ESI+) calcd for $[C_7H_{10}FNNaO_4]^+$ = 214.0486, found: 214.0479.

(2-Fluoro-2-nitrobut-3-en-1-yl)benzene (1o)

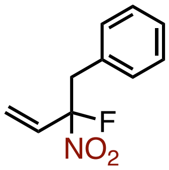

MW (g/mol): 195.19 **Molecular formula: $C_{10}H_{10}FNO_2$**

Prepared following general sequence **GS4**. Purification by flash column chromatography over silica gel (PE/Et_2O 100:00 to 90:10) afforded **2o** as a colorless oil in 48% yield (279 mg, 1.43 mmol).

^{1}H NMR (400 MHz, $CDCl_3$) δ 7.32-7.21 (m, 3H), 7.13 (ddt, *J* = 5.4, 2.7, 1.2 Hz, 2H), 6.19 (ddd, *J* = 18.2, 17.2, 10.9 Hz, 1H), 5.65 (d, *J* = 17.2 Hz, 1H), 5.48 (dd, *J* = 10.9, 1.7 Hz, 1H), 3.54 (dd, *J* = 25.0, 14.6 Hz, 1H), 3.38 (dd, *J* = 18.1, 14.7 Hz, 1H).

^{13}C NMR (101 MHz, $CDCl_3$) δ 130.8, 130.7 (d, *J* = 20.2 Hz), 130.3, 128.6, 128.1, 121.5 (d, *J* = 9.9 Hz), 117.9 (d, *J* = 243.6 Hz), 43.4 (d, *J* = 21.4 Hz).

IR (neat) cm^{-1}: 3034 (w), 2926 (w), 1703 (w), 1563 (s), 1497 (m), 1456 (m), 1413 (m), 1358 (m), 1279 (w), 1252 (w), 953 (m), 838 (m), 728 (m).

HRMS *m/z* (ESI+) calcd for $[C_7H_7]^+$ = 91.0542, found: 91.0541.

1-Chloro-4-(2-fluoro-2-nitrobut-3-en-1-yl)benzene (1p)

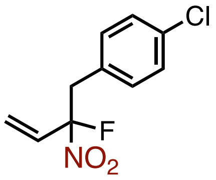

MW (g/mol): 229,64 **Molecular formula: $C_{10}H_9ClFNO_2$**

Prepared following general sequence **GS4**. Purification by flash column chromatography over silica gel (PE/Et_2O 100:00 to 90:10) afforded **2p** as a colorless oil in 44% yield (303 mg, 1.32 mmol)

^{1}H NMR (400 MHz, $CDCl_3$) δ 7.31-7.26 (m, 2H), 7.16-7.11 (m, 2H), 6.23 (ddd, *J* = 18.2, 17.2, 10.9 Hz, 1H), 5.72 (d, *J* = 17.2 Hz, 1H), 5.56 (dd, *J* = 10.9, 1.7 Hz, 1H), 3.58 (dd, *J* = 25.3, 14.7 Hz, 1H), 3.41 (dd, *J* = 17.6, 14.7 Hz, 1H).

^{13}C NMR (101 MHz, $CDCl_3$) δ 134.3, 131.6, 130.4 (dd, *J* = 22.3, 1.7 Hz), 129.3, 128.8, 121.8 (d, *J* = 9.7 Hz), 117.6 (d, *J* = 243.6 Hz), 42.6 (d, *J* = 21.6 Hz).

IR (neat) cm^{-1}: 2931 (w), 1907 (w), 1562 (s), 1491 (m), 1412 (m), 1356 (w), 1250 (w), 1091 (m), 1017 (m), 953 (m), 847 (m), 802 (m), 749 (m).

HRMS *m/z* (ESI+) not found.

4-(2-Fluoro-2-nitrobut-3-en-1-yl)benzonitrile (1q)

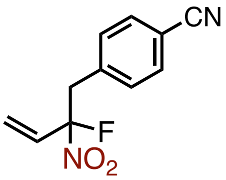

MW (g/mol): 220.20 **Molecular formula: $C_{11}H_9FN_2O_2$**

Prepared following general sequence **GS4**. Purification by flash column chromatography over silica gel (PE/EtOAc 95:05 to 80:20) afforded **1q** as an orange gum in 34% yield (224 mg, 1.02 mmol).

^{1}H NMR (400 MHz, $CDCl_3$) δ 7.61 (d, J = 8.6 Hz, 2H), 7.32 (ddd, J = 8.0, 1.0, 0.5 Hz, 2H), 6.23 (ddd, J = 17.2, 10.9 Hz, J_F = 18.2, 1H), 5.73 (d, J = 17.2 Hz, 1H), 5.59 (dd, J = 10.9, J_F = 1.8 Hz, 1H), 3.67 (dd, J = 14.7 Hz, J_F = 25.1, 1H), 3.50 (dd, J = 14.7 Hz, J_F = 17.2, 1H).

^{13}C NMR (101 MHz, $CDCl_3$) δ 136.3, 132.5, 131.3 (d, J_F = 1.1 Hz), 130.3 (d, J_F = 22.1 Hz), 122.4 (d, J_F = 9.9 Hz), 118.4, 117.40 (d, J_F = 244.2 Hz), 112.5, 43.2 (d, J_F = 21.3 Hz).

IR (neat) cm^{-1}: 2230 (m), 1723 (w), 1564 (s), 1507 (w), 1415 (w), 1358 (w), 1280 (w), 1253 (w), 1178 (w), 957 (w), 857 (w).

HRMS *m/z* (ESI+) calcd for $[C_{11}H_{10}FN_2O_2]^+$ = 221.0721, found: 221.0721.

1-(2-Fluoro-2-nitrobut-3-en-1-yl)-4-(trifluoromethyl)benzene (1r)

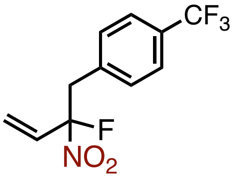

MW (g/mol): 263.19 **Molecular formula: $C_{11}H_9F_4NO_2$**

Prepared following general sequence **GS4**. Purification by flash column chromatography over silica gel (PE/Et_2O 100:00 to 90:10) afforded **2r** as a pale-yellow oil in 30% yield (234 mg, 0.89 mmol).

^{1}H NMR (400 MHz, $CDCl_3$) δ 7.58 (d, J = 8.4 Hz, 2H), 7.33 (d, J = 8.4 Hz, 2H), 6.24 (ddd, J = 17.2, 11.0 Hz, J_F = 18.3, 1H), 5.74 (d, J = 17.2 Hz, 1H), 5.59 (dd, J = 11.0, J_F = 1.8 Hz, 1H), 3.67 (dd, J = 14.7 Hz, J_F = 25.1, 1H), 3.51 (dd, J = 14.7 Hz, J_F = 17.4, 1H).

^{13}C NMR (101 MHz, $CDCl_3$) δ 134.8 (d, J_F = 1.5 Hz), 130.7 (d, J_F = 0.9 Hz), 130.5 (q, J_F = 32.6 Hz), 130.3 (d, J_F = 22.1 Hz), 125.6 (q, J_F = 3.7 Hz), 123.9 (q, J_F = 272.2 Hz), 122.1 (d, J_F = 10.0 Hz), 117.5 (d, J_F = 244.0 Hz), 43.0 (d, J_F = 21.4 Hz).

IR (neat) cm^{-1}: 2998 (w), 1621 (w), 1566 (s), 1420 (w), 1359 (w), 1323 (s), 1255 (w), 1166 (m), 1122 (s), 1067 (s), 956 (m) 853 (w), 630 (w).

HRMS (EI+): Calculated for $C_{11}H_9F_4^+$ [M^+]: 217.0635; Found: 217.0604.

1-(2-Fluoro-2-nitrobut-3-en-1-yl)-4-methoxybenzene (1s)

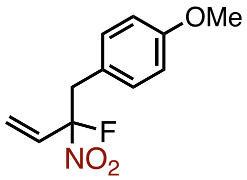

MW (g/mol): 225.22 **Molecular formula: $C_{11}H_{12}FNO_3$**

Prepared following general sequence **GS4**. Purification by flash column chromatography over silica gel (PE/Et_2O 100:00 to 80:20) afforded **1s** as a pale yellow oil in 41% yield (276 mg, 1.22 mmol).

^{1}H NMR (400 MHz, $CDCl_3$) δ 7.16-7.00 (m, 2H), 6.92-6.75 (m, 2H), 6.24 (ddd, J = 18.2, 17.2, 10.9 Hz, 1H), 5.71 (d, J = 17.2 Hz, 1H), 5.54 (dd, J = 10.9, 1.7 Hz, 1H), 3.79 (s, 3H), 3.54 (dd, J = 25.5, 14.8 Hz, 1H), 3.38 (dd, J = 17.8, 14.8 Hz, 1H).

^{13}C NMR (101 MHz, CDCl3) δ 159.4, 131.4, 130.8 (d, J = 22.3 Hz), 122.7, 121.4 (d, J = 9.8 Hz), 118.1 (d, J = 242.9 Hz), 114.0, 55.2, 42.7 (d, J = 21.6 Hz).

IR (neat) cm^{-1}: 2942 (w), 2840 (w), 1563 (s), 1496 (m), 1464 (w), 1358 (w), 1250 (s), 1178 (w), 1117 (w), 1028 (m), 813 (s), 754 (m).

HRMS *m/z* (ESI+) calcd for $[C_{11}H_{12}FO]^+$ = 179.0867, found: 179.0866.

1-(2-Fluoro-2-nitrobut-3-en-1-yl)-3-methoxybenzene (1t)

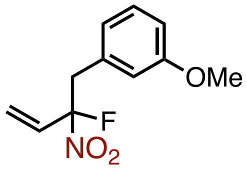

MW (g/mol): 225.22 **Molecular formula: $C_{11}H_{12}FNO_3$**

Prepared following general sequence **GS4**. Purification by flash column chromatography over silica gel (PE/Et_2O 100:00 to 80:20) afforded **1t** as a pale-yellow oil in 41% yield (276 mg, 1.22 mmol).

^{1}H NMR (400 MHz, $CDCl_3$) δ 7.23 (dd, J = 8.3, 7.5 Hz, 1H), 6.85 (ddd, J = 8.4, 2.6, 0.9 Hz, 1H), 6.80-6.76 (m, 1H), 6.75-6.72 (m, 1H), 6.24 (ddd, J = 18.2, 17.2, 10.9 Hz, 1H), 5.72 (d, J = 17.2 Hz, 1H), 5.55 (dd, J = 10.9, 1.7 Hz, 1H), 3.79 (s, 3H), 3.57 (dd, J = 24.8, 14.6 Hz, 1H), 3.42 (dd, J = 18.2, 14.7 Hz, 1H).

^{13}C NMR (101 MHz, $CDCl_3$) δ 159.6, 132.2, 130.7 (d, J = 22.2 Hz), 129.6, 122.6, 121.5 (d, J = 9.9 Hz), 117.9 (d, J = 243.7 Hz), 116.1, 113.5, 55.2, 43.4 (d, J = 21.5 Hz).

IR (neat) cm^{-1}: 2942(w), 2838 (w), 1561 (s), 1500 (m), 1461 (w), 1359 (w), 1253 (s), 1180 (w), 1115 (w), 1029 (m), 810 (s), 755 (m).

HRMS *m/z* (ESI+) calcd for $C_{11}H_{12}FO]^+$ = 179.0867, found: 179.0865.

1-(2-Fluoro-2-nitrobut-3-en-1-yl)-2-methoxybenzene (1u)

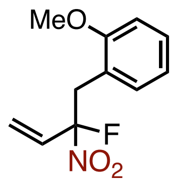

MW (g/mol): 225.22 **Molecular formula: $C_{11}H_{12}FNO_3$**

Prepared following general sequence **GS4**. Purification by flash column chromatography over silica gel (PE/Et_2O 100:00 to 80:20) afforded **1u** as a pale-yellow oil in 40% yield (271 mg, 1.20 mmol).

^{1}H NMR (400 MHz, $CDCl_3$) δ 7.35-7.25 (m, 1H), 7.16 (dt, *J* = 7.6, 1.5 Hz, 1H), 6.96-6.82 (m, 2H), 6.30 (ddd, *J* = 18.8, 17.2, 11.0 Hz, 1H), 5.62 (d, *J* = 17.2 Hz, 1H), 5.46 (dd, *J* = 10.9, 1.8 Hz, 1H), 3.82 (s, 3H), 3.75 (dd, *J* = 21.1, 14.4 Hz, 1H), 3.49 (dd, *J* = 20.7, 14.4 Hz, 1H).

^{13}C NMR (101 MHz, $CDCl_3$) δ 157.9, 131.8 (d, *J* = 1.1 Hz), 130.8 (d, *J* = 21.7 Hz), 129.5, 120.5 (d, *J* = 10.3 Hz), 120.5, 119.6 (d, *J* = 1.2 Hz), 118.0 (d, *J* = 244.5 Hz), 110.7, 55.3, 37.0 (d, *J* = 22.0 Hz).

IR (neat) cm^{-1}: 2940 (w), 2843 (w), 1560 (s), 1495 (m), 1467 (w), 1353 (w), 1256 (s), 1177 (w), 1114 (w), 1030 (m), 811 (s), 757 (m).

HRMS *m/z* (ESI+) calcd for $C_{11}H_{12}FO]^+$ = 179.0867, found: 179.0862.

2-(2-Fluoro-2-nitrobut-3-en-1-yl)furan (1v)

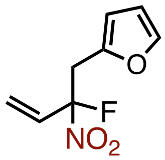

MW (g/mol): 185.15 **Molecular formula: $C_8H_8FNO_3$**

Prepared following general sequence **GS4**. Purification by flash column chromatography over silica gel (PE/Et_2O 100:00 to 90:10) afforded **1v** as a orange oil in 36% yield (201 mg, 1.09 mmol).

^{1}H NMR (400 MHz, $CDCl_3$) δ 7.36 (dd, *J* = 1.9, 0.8 Hz, 1H), 6.32 (dd, *J* = 3.3, 1.9 Hz, 1H), 6.29-6.15 (m, 2H), 5.77 (d, *J* = 17.2 Hz, 1H), 5.58 (dd, *J* = 10.9, 1.7 Hz, 1H), 3.77-3.48 (m, 2H).

^{13}C NMR (101 MHz, $CDCl_3$) δ 145.1, 143.0, 130.2 (d, *J* = 22.0 Hz), 121.8 (d, *J* = 10.0 Hz), 116.6 (d, *J* = 244.1 Hz), 110.7, 110.1, 36.4 (d, *J* = 22.9 Hz).

IR (neat) cm^{-1}: 2983 (m), 1712 (w), 1503 (w), 1331 (s), 1127 (s), 1017 (m), 983 (m), 869 (m), 841 (m), 744 (m), 608 (w).

HRMS *m/z* (ESI+) calcd for $C_{11}H_{12}FO]^+$ = 179.0867, found: 179.0865.

(3-Fluoro-3-nitrobut-1-en-2-yl)benzene (1w)

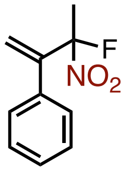

MW (g/mol): 195.19 **Molecular formula: $C_{10}H_{10}FNO_2$**

Prepared following general sequence **GS4**. Purification by flash column chromatography over silica gel (PE/Et_2O 100:00 to 90:10) afforded **1w** as a pale-yellow oil in 53% yield (310 mg, 1.59 mmol). *[Note: this compound was stored at –20 °C as it is relatively sensitive to heat and acid and tends to spontaneously rearrange through a [2,3] sigmatropic rearrangement at rt]*

^{1}H NMR (400 MHz, $CDCl_3$) δ 7.29-7.23 (m, 3H), 7.22-7.12 (m, 2H), 5.82 (d, *J* = 2.3 Hz, 1H), 5.59 (d, *J* = 1.4 Hz, 1H), 2.02 (d, *J* = 19.6 Hz, 3H).

^{13}C NMR (101 MHz, $CDCl_3$) δ 143.62 (d, *J* = 21.2 Hz), 135.61, 128.72, 128.40, 128.24 (d, *J* = 1.4 Hz), 122.18 (d, *J* = 7.9 Hz), 118.53 (d, *J* = 237.9 Hz), 23.40 (d, *J* = 24.2 Hz).

IR (neat) cm^{-1}: 3026 (w), 1691 (m), 1552 (s), 1494 (w), 1445 (), 1428 (m), 1386 (m), 1370 (m), 1198 (m), 1021 (s), 745 (m)**.**

HRMS *m/z* (ESI+) calcd for $[C_{10}H_{10}F]^+$ = 149.0761, found: 149.0760.

2-(2-Fluoro-2-nitrobut-3-en-1-yl)naphthalene (1x)

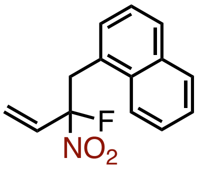

MW (g/mol): 245.25 **Molecular formula: $C_{14}H_{12}FNO_2$**

Prepared following general sequence **GS4**. Purification by flash column chromatography over silica gel (PE/Et_2O 100:00 to 90:10) afforded **1x** as a yellow oil in 39% yield (289 mg, 1.18 mmol).

^{1}H NMR (400 MHz, $CDCl_3$) δ 8.01 (ddd, *J* = 8.5, 2.1, 1.1 Hz, 1H), 7.87 (dd, *J* = 8.1, 1.6 Hz, 1H), 7.83 (dt, *J* = 8.2, 1.1 Hz, 1H), 7.57 (ddd, *J* = 8.5, 6.9, 1.6 Hz, 1H), 7.51 (ddd, *J* = 8.0, 6.8, 1.3 Hz, 1H), 7.42 (dd, *J* = 8.2, 7.1 Hz, 1H), 7.36 (dt, *J* = 7.1, 1.1 Hz, 1H), 6.34 (ddd, *J* = 18.6, 17.2, 10.9 Hz, 1H), 5.68 (d, *J* = 17.2 Hz, 1H), 5.49 (dd, *J* = 10.9, 1.8 Hz, 1H), 4.06 (d, *J* = 2.6 Hz, 1H), 4.01 (d, *J* = 5.2 Hz, 1H).

^{13}C NMR (101 MHz, $CDCl_3$) δ 133.9, 132.5, 130.7 (d, *J* = 22.0 Hz), 129.2, 129.1, 128.8, 127.3, 126.5, 125.9, 125.2, 123.7 (d, *J* = 3.0 Hz), 121.4 (d, *J* = 10.0 Hz), 118.5 (d, *J* = 245.6 Hz), 39.6 (d, *J* = 21.9 Hz).

IR (neat) cm^{-1}: 3103 (w), 1564 (s), 1512 (w), 1412 (w), 1356 (w), 1254 (w), 1114 (w), 1020 (w), 954 (w), 818 (w), 779 (s), 498 (w).

HRMS *m/z* (ESI+) calcd for $[C_{14}H_{12}F]^+$ = 199.0918, found: 199.0909.

Dimethyl (*E*)-4-nitro-4-(prop-1-en-1-yl)heptanedioate (3a)

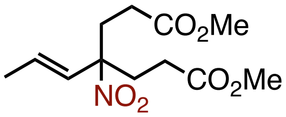

MW (g/mol): 273.28 **Molecular formula: $C_{12}H_{19}NO_6$**

Prepared following general sequence **GS1**. Purification by flash column chromatography over silica gel (PE/EtOAc 95:05 to 80:20) afforded **1ba** as a pale-yellow oil in 52% yield (983 mg, 3.60 mmol).

^{1}H NMR (400 MHz, $CDCl_3$) δ 5.84-5.71 (m, 2H), 3.68 (s, 6H), 2.47-2.19 (m, 8H), 1.82-1.76 (m, 3H).

^{13}C NMR (101 MHz, $CDCl_3$) δ 172.5, 130.2, 127.4, 93.0, 52.0, 32.2, 28.9, 18.2.

IR (neat) cm^{-1}: 2949 (w), 1730 (s), 1536 (s), 1439 (m), 1354 (m), 1345 (m), 1201 (s), 1178 (s), 992 (w), 883 (w), 841 (w), 801 (w).

HRMS *m/z* (ESI+) calcd for $[C_{12}H_{19}NNaO_6]^+$ = 296.1105, found: 296.1103.

Methyl 3-(1-nitrocyclohex-2-en-1-yl)propanoate (3b)

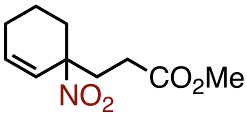

MW (g/mol): 213.23 **Molecular formula: $C_{10}H_{15}NO_4$**

Methyl 3-(1-nitrocyclohex-2-en-1-yl)propanoate **3b** was synthesized following a reported procedures.[1]

^{1}H NMR (400 MHz, CD_3OD) δ 6.11 (dddd, J = 10.1, 4.5, 3.0, 0.7 Hz, 1H), 5.90 (dddd, J = 10.1, 2.5, 1.9, 1.4 Hz, 1H), 3.66 (s, 3H), 2.57-2.45 (m, 1H), 2.44-2.30 (m, 2H), 2.34-2.19 (m, 2H), 2.23-2.07 (m, 1H), 2.12-1.98 (m, 1H), 1.87-1.69 (m, 2H), 1.63-1.47 (m, 1H).

^{13}C NMR (101 MHz, CD_3OD) δ 174.3, 135.3, 126.3, 89.6, 52.3, 36.5, 32.5, 29.4, 25.7, 19.9.

The spectroscopic data of the product were identical with those reported in the literature.[1]

Dimethyl 4-[2-(4,4,5,5-tetramethyl-1,3,2-dioxaborolan-2-yl)ethylidene]heptanedioate (2a)

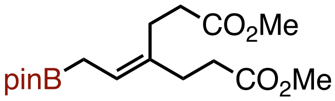

MW (g/mol): 340.22 **Molecular formula: $C_{17}H_{29}BO_6$**

Prepared following general sequence **GP-A**. Purification by flash column chromatography over silica gel (PE/EtOAc 95:05 to 85:15) afforded **2a** as a colorless oil in 89% yield (152 mg, 0.45 mmol).

---

[1] Alameda-Angulo, C.; Quiclet-Sire, B.; Schmidt, E.; Zard, S. Z. *Org. Lett.* **2005**, *7*, 3489-3492.

^{1}**H NMR** (400 MHz, $CDCl_3$) δ 5.30 (t, *J* = 8.0 Hz, 1H), 3.65 (dd, *J* = 2.2, 0.7 Hz, 6H), 2.45-2.23 (m, 8H), 1.62 (d, *J* = 7.9 Hz, 2H), 1.22 (d, *J* = 0.7 Hz, 12H).

^{13}**C NMR** (101 MHz, $CDCl_3$) δ 173.8, 135.4, 121.4, 83.2, 51.6, 33.1, 32.6, 31.6, 25.2, 24.7.

IR (neat) cm^{-1}: 2975 (w), 2918 (w), 1746 (s), 1439 (w), 1363 (m), 1335 (s), 1274 (m), 1218 (m), 1133 (s), 1094 (w), 977 (m), 888 (m), 841 (m).

HRMS *m/z* (ESI+) calcd for $[C_{17}H_{30}BO_6]^+$ = 340.2230, found: 179.0862.

Methyl (*E*)-4-methyl-6-(4,4,5,5-tetramethyl-1,3,2-dioxaborolan-2-yl)hex-4-enoate (2b)

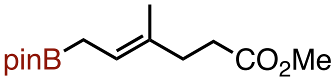

MW (g/mol): 268.16 **Molecular formula: $C_{14}H_{25}BO_4$**

Prepared following general sequence **GP-A**. Purification by flash column chromatography over silica gel (PE/EtOAc 95:05 to 85:15) afforded **2b** as a mixture of *E* and *Z* diastereoisomers in 90% yield (121 mg, 0.45 mmol).

Major Isomer (E)

^{1}**H NMR** (400 MHz, C_6D_6) δ 5.54-5.44 (m, 1H), 3.34 (s, 3H), 2.43-2.20 (m, 4H), 1.74 (d, *J* = 7.8 Hz, 2H), 1.51 (d, *J* = 1.2 Hz, 3H), 1.02 (s, 12H).

^{13}**C NMR** (101 MHz, $CDCl_3$) δ 174.0, 133.3, 119.6, 83.1, 51.5, 34.8, 33.2, 24.8, 15.8.

Minor Isomer Z

^{1}**H NMR** (400 MHz, C_6D_6) δ 5.5-5.44 (m, 1H), 3.34 (s, 3H), 2.43-2.20 (m, 4H), 1.79 (d, *J* = 7.9 Hz, 2H), 1.57 (q, *J* = 1.3 Hz, 3H), 1.03 (s, 12H).

^{13}**C NMR** (101 MHz, $CDCl_3$) δ 174.0, 133.2, 120.6, 83.2, 51.5, 32.5, 27.1, 24.8, 23.1.

IR (neat) cm^{-1}: 2976 (w), 2926 (w), 1738 (s), 1437 (w), 1370 (m), 1321 (s), 1272 (m), 1214 (m), 1143 (s), 1081 (w), 967 (m), 884 (m), 843 (m).

HRMS *m/z* (ESI+) calcd for $[C_{14}H_{25}BLiO_4]^+$ = 275.2000, found: 275.1998.

(*E*)-4-Methyl-6-(4,4,5,5-tetramethyl-1,3,2-dioxaborolan-2-yl)hex-4-enenitrile (2c)

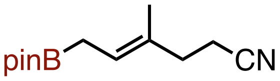

MW (g/mol): 235.13 **Molecular formula: $C_{13}H_{22}BNO_2$**

Prepared following general sequence **GP-A**. Purification by flash column chromatography over silica gel (PE/EtOAc 95:05 to 85:15) afforded **2c** as a mixture of *E* and *Z* diastereoisomers in 75% yield (89 mg, 0.38 mmol).

Major Isomer E

^{1}H NMR (400 MHz, C_6D_6) δ 5.47 (tdd, *J* = 6.6, 2.7, 1.4 Hz, 1H), 1.85-1.73 (m, 4H), 1.62 (t, *J* = 7.3 Hz, 2H), 1.42 (d, *J* = 0.9 Hz, 3H), 1.16 (s, 12H).

^{13}C NMR (101 MHz, $CDCl_3$) δ 131.1, 122.2, 119.7, 83.3, 35.1, 24.8, 16.5, 15.6.

Minor Isomer Z

^{1}H NMR (400 MHz, C_6D_6) δ 5.57 (t, *J* = 8.1 Hz, 1H), 1.99 (t, *J* = 7.6 Hz, 2H), 1.85-1.73 (m, 2H), 1.63 (d, *J* = 6.9 Hz, 2H), 1.50 (q, *J* = 1.3 Hz, 3H), 1.14 (s, 12H).

^{13}C NMR (101 MHz, $CDCl_3$) δ 131.1, 122.8, 119.7, 83.4, 27.5, 24.8, 23.0, 15.7.

IR (neat) cm^{-1}: 3208 (w), 2978 (w), 2254 (s), 1449 (m), 1371 (m), 1323 (s), 1273 (m), 1214 (m), 1142 (s), 967 (m), 864 (m), 843 (m), 745 (w).

HRMS *m/z* (ESI+) calcd for $[C_{13}H_{22}BLiNO_2]^+$ = 242.1889, found: 242.1898.

(*E*)-4,4,5,5-Tetramethyl-2-[3-methyl-5-(phenylsulfonyl)pent-2-en-1-yl]-1,3,2-dioxaborolane (2d)

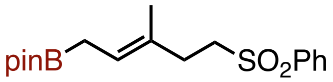

MW (g/mol): 350.28 **Molecular formula: $C_{18}H_{27}BO_4S$**

Prepared following general sequence **GP-A**. Purification by flash column chromatography over silica gel (PE/EtOAc 95:05 to 85:15) afforded **2d** as a mixture of *E* and *Z* diastereoisomers in 95% yield (166 mg, 0.48 mmol).

Major Isomer E

^{1}H NMR (400 MHz, $CDCl_3$) δ 7.92-7.86 (m, 2H), 7.65-7.60 (m, 1H), 7.57-7.51 (m, 2H), 5.22 (tq, *J* = 7.8, 1.3 Hz, 1H), 3.16-3.10 (m, 2H), 2.38-2.30 (m, 2H), 1.52 (d, *J* = 7.8 Hz, 2H), 1.49 (d, *J* = 1.3 Hz, 3H), 1.19 (s, 12H).

^{13}C NMR (101 MHz, $CDCl_3$) δ 139.2, 133.7, 130.6, 129.3, 128.2, 121.7, 83.3, 55.2, 32.4, 24.8, 15.8.

Minor Isomer Z

^{1}H NMR (400 MHz, $CDCl_3$) δ 7.92-7.86 (m, 2H), 7.65-7.60 (m, 1H), 7.57-7.51 (m, 2H), 5.26 (t, *J* = 7.8 Hz, 1H), 3.16-3.10 (m, 2H), 2.38-2.30 (m, 2H), 1.49 (d, *J* = 1.3 Hz, 3H), 1.44 (d, *J* = 7.8 Hz, 2H), 1.19 (s, 12H).

^{13}C NMR (101 MHz, $CDCl_3$) δ 139.2, 133.7, 130.4, 129.3, 128.1, 122.5, 83.4, 54.3, 32.4, 24.8, 15.8.

HRMS *m/z* (ESI+) calcd for $[C_{18}H_{27}BLiO_4S]^+$ = 357.1878, found: 357.1872.

(E)-*tert*-Butyldimethyl[(2-methyl-4-(4,4,5,5-tetramethyl-1,3,2-dioxaborolan-2-yl)but-2-en-1-yl)oxy]silane (2e)

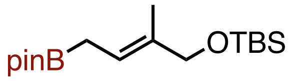

MW (g/mol): 326.36 **Molecular formula: $C_{17}H_{35}BO_3Si$**

Prepared following general sequence **GP-A**. Purification by flash column chromatography over silica gel (PE/Et_2O 95:05 to 85:15) afforded **2e** as a mixture of *E* and *Z* diastereoisomers in 79% yield (129 mg, 0.40 mmol).

<u>*Major Isomer E*</u>

^{1}H NMR (400 MHz, C_6D_6) δ 5.79 (tq, *J* = 7.9, 1.4 Hz, 1H), 4.04 (s, 2H), 1.84 (d, *J* = 7.9 Hz, 2H), 1.69 (s, 3H), 1.04 (s, 12H), 1.00 (s, 9H), 0.08 (s, 6H).

^{13}C NMR (101 MHz, C_6D_6) δ 133.0, 119.2, 81.6, 68.0, 24.8, 23.5, 20.0, 17.2, 12.2, –6.4.

<u>*Minor Isomer Z*</u>

^{1}H NMR (400 MHz, C_6D_6) δ 5.58 (t, *J* = 7.5 Hz, 1H), 4.27 (s, 2H), 1.87 (s, H), 1.86 (d, *J* = 9.3 Hz, 2H), 1.03 (s, 12H), 0.99 (s, 9H), 0.09 (s, 6H).

^{13}C NMR (101 MHz, C_6D_6) δ 133.3, 119.9, 81.7, 60.7, 24.8, 23.5, 20.0, 17.2, 12.2, –6.5.

IR (neat) cm^{-1}: 2955 (w), 2928 (w), 2856 (m), 1471 (w), 1360 (m), 1340 (s), 1322 (s), 1251 (m), 1145 (s), 1104 (m), 1062 (s), 967 (w).

HRMS *m/z* (ESI+) calcd for $[C_{17}H_{35}BLiO_3Si]^+$ = 333.2603, found: 333.2599.

Methyl (Z)-4-(4-methoxybenzyl)-6-(4,4,5,5-tetramethyl-1,3,2-dioxaborolan-2-yl)hex-4-enoate (2f)

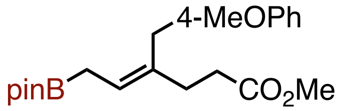

MW (g/mol): 374.28 **Molecular formula: $C_{21}H_{31}BO_5$**

Prepared following general sequence **GP-A**. Purification by flash column chromatography over silica gel (PE/EtOAc 90:10 to 75:25) afforded **2f** as a mixture of *E* and *Z* diastereoisomers in 88% yield (165 mg, 0.44 mmol).

<u>*Major compound Z*</u>

^{1}H NMR (400 MHz, $CDCl_3$) δ 7.16-6.95 (m, 2H), 6.87-6.70 (m, 2H), 5.51-5.33 (m, 1H), 3.75 (s, 3H), 3.60 (s, 3H), 3.31 (s, 2H), 2.40-2.19 (m, 4H), 1.74 (d, *J* = 7.9 Hz, 1H), 1.23 (s, 12H).

^{13}C NMR (101 MHz, $CDCl_3$) δ 173.7, 157.7, 136.0, 131.8, 129.3, 121.2, 113.6, 83.1, 55.1, 51.3, 34.8, 33.0, 32.5, 24.7.

Minor isomer E

^{1}H NMR (400 MHz, $CDCl_3$) δ 7.16-6.95 (m, 2H), 6.87-6.70 (m, 2H), 5.51-5.33 (m, 1H), 3.76 (s, 3H), 3.61 (s, 3H), 3.24 (s, 2H), 2.40-2.19 (m, 4H), 1.67 (d, J = 8.0 Hz, 1H), 1.23 (s, 12H).

^{13}C NMR (101 MHz, $CDCl_3$) δ 173.6, 157.8, 136.5, 132.2, 129.6, 122.9, 113.5, 83.1, 55.1, 51.3, 42.6, 31.6, 24.7, 24.5.

IR (neat) cm^{-1}: 2976 (w), 1737 (s), 1452 (m), 1436 (m), 1322 (s), 1271 (m), 1165 (m), 1142 (s), 1052 (w) 967 (m), 884 (w), 844 (m).

HRMS *m/z* (ESI+) calcd for $[C_{21}H_{31}BLiO_5]^+$ = 381.2419, found: 381.2410.

Methyl 7-oxo-4-(2-(4,4,5,5-tetramethyl-1,3,2-dioxaborolan-2-yl)ethylidene)octanoate (2g)

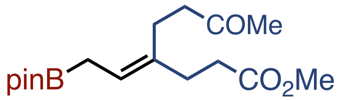

MW (g/mol): 324.22 **Molecular formula: $C_{17}H_{29}BO_5$**

Prepared following general sequence **GP-A**. Purification by flash column chromatography over silica gel (PE/Et_2O 95:5 to 85:15) afforded **2g** in 19% yield (30 mg, 0.092 mmol) as an unseparable 1:1 mixture of the *E* and the *Z* isomeres.

^{1}H NMR (400 MHz, $CDCl_3$) δ 5.28 (t, J = 8.0 Hz, 1H), 3.66 (s, 3H, diastereoisomer 1), 3.65 (s, 3H, diastereoisomer 2), 2.54-2.47 (m, 2H), 2.42-2.35 (m, 2H), 2.35-2.22 (m, 4H), 2.13 (s, 3H), 1.22 (s, 12H).

^{13}C NMR (101 MHz, C_6D_6) for the mixture of d*iastereoisomers* δ 208.9, 208.8, 174.0, 173.9, 136.0, 135.8, 121.3, 121.0, 83.4, 83.3, 51.7, 51.7, 42.5, 42.2, 33.2, 32.7, 31.9, 30.6, 30.1, 30.0, 25.4, 24.9, 24.9, 24.1.

IR (neat) cm^{-1} : 2977, 1735, 1715, 1323, 1142, 967, 845.

HRMS *m/z* (ESI+) calcd for $[C_{17}H_{30}BO_5]^+$ = 325.2181, found: 325.2189.

[(2-Benzyl-4-(4,4,5,5-tetramethyl-1,3,2-dioxaborolan-2-yl)but-2-en-1-yl)oxy](*tert*-butyl)dimethylsilane (2h)

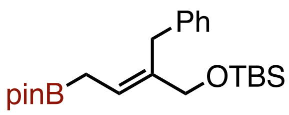

MW (g/mol): 402.45 **Molecular formula: $C_{23}H_{39}BO_3Si$**

Prepared following general sequence **GP-A** (reaction run over 24 h instead of 3 h). Purification by flash column chromatography over silica gel (PE/Et_2O 95:05 to 85:15) afforded **2h** in 46% yield (93 mg, 0.23 mmol) as well as **2h'** (see below).

^{1}H NMR (400 MHz, $CDCl_3$) δ 7.29-7.12 (m, 5H), 5.71 (t, *J* = 8.0 Hz, 1H), 3.97 (d, *J* = 1.2 Hz, 2H), 3.44 (s, 2H), 1.80 (d, *J* = 8.0 Hz, 2H), 1.26 (s, 12H), 0.89 (s, 9H), 0.00 (s, 6H).

^{13}C NMR (101 MHz, C_6D_6) δ 140.5, 132.9, 130.6, 129.0, 128.4, 122.0, 83.1, 55.2, 32.9, 25.5, 24.9, 23.0, 15.6.

IR (neat) cm^{-1}: 2956 (m), 2928 (m), 2856 (m), 1532 (m), 1360 (m), 1322 (s), 1145 (s), 1062 (s), 885 (m), 834 (s), 773 (s).

HRMS *m/z* (ESI+) calcd for $[C_{17}H_{24}BO_2]^+$ = 271.1864, found: 271.1862.

5-Benzyl-3,6-dihydro-2H-1,2-oxaborinin-2-ol (2h')

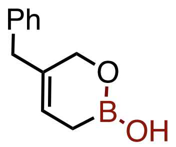

MW (g/mol): 188.03 **Molecular formula: $C_{11}H_{13}BO_2$**

Prepared following general sequence **GP-A** (reaction run over 24 h instead of 3 h). Purification by flash column chromatography over silica gel (PE/EtOAc 85:15 to 65:35) afforded **2h'** in 48% yield (45 mg, 0.24 mmol)

^{1}H NMR (400 MHz, CD_3OD) δ 7.25 (t, *J* = 7.3 Hz, 2H), 7.16 (t, *J* = 8.6 Hz, 3H), 5.59 (s, 1H), 4.38 (s, 2H), 3.22 (s, 2H), 1.36 (s, 2H).

^{13}C NMR (101 MHz, CD_3OD) δ 140.6, 136.8, 129.6, 129.4, 127.3, 122.0, 68.1, 40.8.

IR (neat) cm^{-1}: 2928 (m), 2857 (m), 1494 (s), 1471 (m), 1453 (m), 1371 (s), 1344 (s), 1255 (m), 1143 (s), 1073 (w), 836 (s), 777 (m), 700 (m).

HRMS *m/z* (ESI+) calcd for $[C_7H_7]^+$ = 91.0542, found: 91.0541.

Dimethyl-[1-phenyl-2-(4,4,5,5-tetramethyl-1,3,2-dioxaborolan-2-yl)ethylidene] heptanedioate (2i)

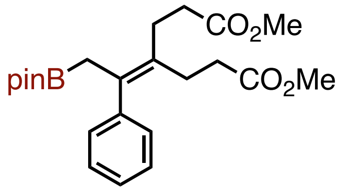

MW (g/mol): 416.32 **Molecular formula: $C_{23}H_{33}BO_6$**

Prepared following general sequence **GP-A**. Purification by flash column chromatography over silica gel (PE/EtOAc 95:05 to 85:15) afforded **2i** as a colorless gum in 89% yield (185 mg, 0.45 mmol).

^{1}H NMR (400 MHz, $CDCl_3$) δ 7.34-7.29 (m, 2H), 7.25-7.19 (m, 1H), 7.17-7.10 (m, 2H), 3.74 (s, 3H), 3.62 (s, 3H), 2.65-2.43 (m, 4H), 2.41-2.15 (m, 4H), 2.02 (s, 2H), 1.20 (s, 12H).

^{13}C NMR (101 MHz, $CDCl_3$) δ 173.88, 173.57, 144.38, 135.57, 130.54, 128.06, 128.03, 126.12, 83.23, 51.54, 51.42, 33.28, 32.80, 27.69, 26.52, 24.70.

IR (neat) cm^{-1}: 2977 (w), 2951 (w), 1734 (s), 1436 (m), 1371 (m), 1338 (s), 1269 (m), 1165 (s), 1141 (s), 967 (m), 880 (s), 846 (m), 770 (s).

HRMS *m/z* (ESI+) calcd for $[C_{23}H_{33}BLiO_6]^+$ = 423.2525, found: 423.2526.

Dimethyl-4-[1-(4-chlorophenyl)-2-(4,4,5,5-tetramethyl-1,3,2-dioxaborolan-2-yl)ethylidene] heptanedioate (2j)

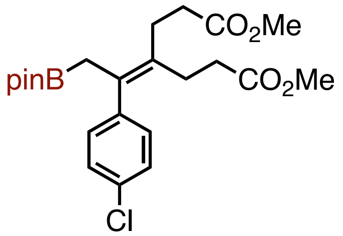

MW (g/mol): 450.76 **Molecular formula: $C_{23}H_{32}BClO_6$**

Prepared following general sequence **GP-A**. Purification by flash column chromatography over silica gel (PE/EtOAc 95:05 to 85:15) afforded **2j** as a pale-yellow gum in 91% yield (205 mg, 0.46 mmol).

^{1}H NMR (400 MHz, $CDCl_3$) δ 7.24 (d, J = 8.4 Hz, 2H), 7.03 (d, J = 8.5 Hz, 2H), 3.68 (s, 3H), 3.58 (s, 3H), 2.51-2.41 (m, 4H), 2.27-2.20 (m, 2H), 2.19-2.11 (m, 2H), 1.26 (s, 2H), 1.15 (s, 12H).

^{13}C NMR (101 MHz, $CDCl_3$) δ 173.8, 173.4, 142.8, 134.4, 131.9, 131.3, 129.6, 128.3, 83.4, 51.6, 51.5, 33.1, 32.7, 27.7, 26.4, 24.7, 24.5.

IR (neat) cm^{-1}: 2981 (w), 1741 (s), 1480 (w), 1438 (m), 1369 (m), 1344 (s), 1272 (m), 1161 (s), 1145 (s), 960 (w), 848 (w), 754 (m).

HRMS *m/z* (ESI+) calcd for $[C_{23}H_{32}BClNaO]^+$ = 473.1873, found: 473.1880.

Dimethyl4-(1-(2-fluorophenyl)-2-(4,4,5,5-tetramethyl-1,3,2-dioxaborolan-2-yl)ethylidene) heptanedioate (2k)

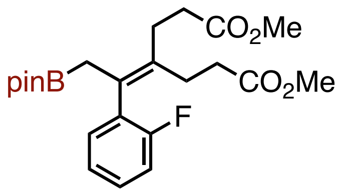

MW (g/mol): 434.31 **Molecular formula: $C_{23}H_{32}BFO_6$**

Prepared following general sequence **GP-A**. Purification by flash column chromatography over silica gel (PE/EtOAc 95:05 to 85:15) afforded **2k** as a pale-yellow oil in 94% yield (204 mg, 0.47 mmol).

^{1}H NMR (400 MHz, $CDCl_3$) δ 7.18 (dddd, *J* = 8.2, 7.2, 5.2, 2.0 Hz, 1H), 7.13-6.96 (m, 3H), 3.69 (s, 3H), 3.57 (s, 3H), 2.58-2.44 (m, 4H), 2.31-2.22 (m, 2H), 2.16 (s, 2H), 1.92 (s, 2H), 1.14 (s, 12H).

^{13}C NMR (101 MHz, $CDCl_3$) δ 173.8, 173.6, 159.2 (d, *J* = 244.2 Hz), 133.1, 131.1 (d, *J* = 17.4 Hz), 130.8 (d, *J* = 4.1 Hz), 128.8, 128.2 (d, *J* = 7.9 Hz), 123.7 (d, *J* = 3.5 Hz), 115.5 (d, *J* = 22.6 Hz), 83.3, 51.6, 51.5, 32.8, 32.7, 28.0, 26.3, 24.7.

IR (neat) cm^{-1}: 2979 (w), 2952 (w), 1736 (s), 1487 (w), 1437 (m), 1371 (m), 1340 (s), 1271 (m), 1168 (s), 1143 (s), 968 (w), 846 (w), 760 (m).

HRMS *m/z* (ESI+) calcd for $[C_{23}H_{33}BFO_6]^+$ = 435.2349, found: 435.2370.

4-[1-Phenyl-2-(4,4,5,5-tetramethyl-1,3,2-dioxaborolan-2yl)ethylidene]heptanedinitrile (2l)

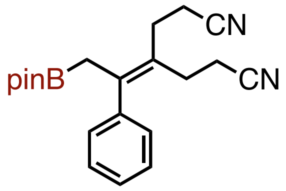

MW (g/mol): 350.27 **Molecular formula: $C_{21}H_{27}BN_2O_2$**

Prepared following general sequence **GP-A**. Purification by flash column chromatography over silica gel (PE/EtOAc 85:15 to 75:25) afforded **2l** as a yellow gum in 94% yield (165 mg, 0.47 mmol).

^{1}H NMR (400 MHz, $CDCl_3$) δ 7.29-7.22 (m, 2H), 7.20-7.14 (m, 1H), 7.08-7.02 (m, 2H), 2.63-2.46 (m, 4H), 2.28-2.09 (m, 4H), 1.94 (s, 2H), 1.12 (s, 12H).

^{13}C NMR (101 MHz, $CDCl_3$) δ 143.0, 140.5, 128.5, 127.8, 127.2, 126.9, 119.5, 119.1, 83.7, 28.1, 27.1, 24.8, 16.4, 16.2.

IR (neat) cm^{-1}: 3204 (w), 2979 (w), 2239 (s), 1442 (m), 1357 (m), 1339 (s), 1287 (m), 1232 (m), 1153 (s), 949 (m), 877 (m), 836 (m), 751 (w).

HRMS *m/z* (ESI+): not found.

(*Z*)-4-Methyl-5-phenyl-6-(4,4,5,5-tetramethyl-1,3,2-dioxaborolan-2-yl)hex-4-enenitrile (2m)

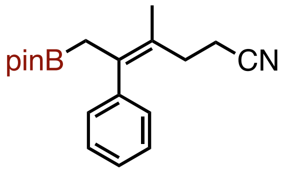

MW (g/mol): 311.23 **Molecular formula: $C_{19}H_{26}BNO_2$**

Prepared following general sequence **GP-A**. Purification by flash column chromatography over silica gel (PE/EtOAc 90:10 to 80:20) afforded **2m** as a mixture of *E* and *Z* diastereoisomers in 91% yield (142 mg, 0.46 mmol).

Major isomer Z

^{1}H NMR (400 MHz, C_6D_6) δ 7.34-7.27 (m, 2H), 7.24-7.17 (m, 2H), 7.10-7.02 (m, 1H), 2.09 (s, 2H), 1.87 (t, *J* = 7.2 Hz, 1H), 1.60 (s, 3H), 1.53 (t, *J* = 7.2 Hz, 2H), 1.01 (s, 12H).

^{13}C NMR (101 MHz, C_6D_6) δ 145.0, 136.2 128.7, 128.6, 126.6, 83.2, 31.0, 24.9, 17.5, 15.9.

Minor isomer E

^{1}H NMR (400 MHz, C_6D_6) δ 7.34-7.27 (m, 2H), 7.24-7.17 (m, 2H), 7.10-7.02 (m, 1H), 2.18-2.11 (m, 2H), 2.03 (s, 2H), 1.95-1.89 (m, 2H), 1.35 (s, 3H), 0.96 (s, 12H).

^{13}C NMR (101 MHz, C_6D_6) δ 145.3, 135.2, 126.5, 126.2, 119.2, 83.3, 30.4, 24.8, 19.4, 15.6.

IR (neat) cm^{-1}: 3198 (w), 2981 (w), 2243 (s), 1432 (m), 1366 (m), 1330 (s), 1278 (m), 1211 (m), 1146 (s), 961 (m), 868 (m), 841 (m), 747 (w).

HRMS *m/z* (ESI+) calcd for $[C_{19}H_{27}BNO_2]^+$ = 312.2129, found: 312.2122.

Methyl (*Z*)-4-fluoro-6-(4,4,5,5-tetramethyl-1,3,2-dioxaborolan-2-yl)hex-4-enoate (2n)

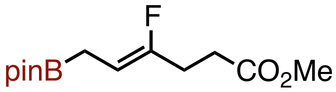

MW (g/mol): 272.12 **Molecular formula: $C_{13}H_{22}BFO_4$**

Compound **2m** was synthesized following general procedure GP-A. Purification by flash column chromatography over silica gel (Pentane/Et_2O 100:00 to 95:05) afforded **2n** as a mixture of *E* and *Z* diastereoisomers in 90% yield (122 mg, 0.45 mmol).

Major isomer Z

^{1}H NMR (400 MHz, $CDCl_3$) δ 4.60 (dt, *J* = 37.5, 7.7 Hz, 1H), 3.63 (s, 3H), 2.66-2.24 (m, 4H), 1.57 (d, *J* = 7.7 Hz, 2H), 1.19 (s, 12H).

^{13}C NMR (101 MHz, $CDCl_3$) δ 172.8, 157.4 (d, *J* = 251.3 Hz), 100.8 (d, *J* = 16.3 Hz), 83.2, 51.6, 31.1, 27.5 (d, *J* = 29.0 Hz), 24.6.

Minor isomer E

^{1}H NMR (400 MHz, $CDCl_3$) δ 5.08 (dt, *J* = 21.3, 8.3 Hz, 1H), 3.63 (s, 3H), 2.66-2.24 (m, 4H), 1.46 (d, *J* = 8.3 Hz, 2H), 1.19 (s, 12H).

^{13}C NMR (101 MHz, $CDCl_3$) δ 172.8, 157.6 (d, *J* = 243.7 Hz), 101.7 (d, *J* = 24.1 Hz), 83.4, 51.6, 30.6, 27.5 (d, *J* = 29.0 Hz), 24.6.

IR (neat) cm^{-1}: 3207 (m), 2979 (w), 1739 (m), 1708 (w), 1438 (m), 1354 (m), 1327 (m), 1195 (m), 1142 (s), 967 (w), 844 (m).

HRMS *m/z* (ESI+) calcd for $[C_{13}H_{22}BFNaO_4]^+$ = 295.1487, found: 295.1493.

(*Z*)-2-(3-Fluoro-4-phenylbut-2-en-1-yl)-4,4,5,5-tetramethyl-1,3,2-dioxaborolane (2o)

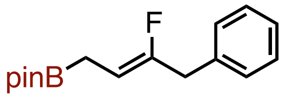

MW (g/mol): 276.16 **Molecular formula: $C_{16}H_{22}BFO_2$**

Prepared following general sequence **GP-A**. Purification by flash column chromatography over silica gel (Pentane/Et_2O 100:00 to 98:02) afforded **2o** as a mixture of *E* and *Z* diastereoisomers in 96% yield (133 mg, 0.48 mmol).

Major isomer Z

^{1}H NMR (400 MHz, $CDCl_3$) δ 7.26-7.12 (m, 5H), 4.65 (dtt, *J* = 36.9, 7.9, 0.8 Hz, 1H), 3.40 (d, *J* = 18.4 Hz, 2H), 1.59 (d, *J* = 6.6 Hz, 2H), 1.17 (s, 12H).

^{13}C NMR (101 MHz, $CDCl_3$) δ 157.8 (d, *J* = 252.6 Hz), 137.1, 128.7, 128.4, 126.5, 102.0 (d, *J* = 16.2 Hz), 83.3, 38.6 (d, *J* = 28.9 Hz), 24.8.

Minor isomer E

^{1}H NMR (400 MHz, $CDCl_3$) δ 7.26-7.12 (m, 5H), 5.18 (dt, *J* = 20.9, 8.4 Hz, 1H), 3.48 (d, *J* = 23.4 Hz, 2H), 1.55 (d, *J* = 8.3 Hz, 1H), 1.17 (s, 12H).

^{13}C NMR (101 MHz, $CDCl_3$) δ 157.7 (d, *J* = 244.7 Hz), 137.0, 128.5, 128.4, 126.5, 102.10 (d, *J* = 24.4 Hz), 83.50, 34.1 (d, *J* = 29.3 Hz), 24.8.

IR (neat) cm^{-1}: 3238 (w), 2978 (s), 1703 (s), 1496 (s), 1454 (s), 1326 (m), 1142 (s), 967 (s), 880 (m), 844 (s), 733 (m).

HRMS *m/z* (ESI+) calcd for $[C_{16}H_{22}BFLiO_2]^+$ = 283.1853, found: 283.1851.

(*Z*)-2-[4-(4-Chlorophenyl)-3-fluorobut-2-en-1-yl]-4,4,5,5-tetramethyl-1,3,2-dioxa-borolane (2p)

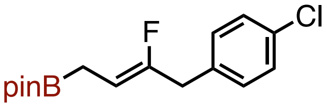

MW (g/mol): 310.13 **Molecular formula: $C_{16}H_{21}BClFO_2$**

Prepared following general sequence **GP-A**. Purification by flash column chromatography over silica gel (Pentane/Et_2O 100:00 to 98:02) afforded **2p** as a mixture of *E* and *Z* diastereoisomers in 95% yield (147 mg, 0.48 mmol).

Major Isomer Z

^{1}H NMR (400 MHz, $CDCl_3$) δ 7.21-7.17 (m, 2H), 7.16-7.09 (m, 2H), 4.65 (dtt, *J* = 36.8, 7.8, 0.8 Hz, 1H), 3.36 (d, *J* = 18.3 Hz, 2H), 1.58 (d, *J* = 7.1 Hz, 2H), 1.17 (s, 12H).

^{13}C NMR (101 MHz, $CDCl_3$) δ 157.20 (d, *J* = 252.5 Hz), 135.6, 132.4, 130.0, 128.5, 102.4 (d, *J* = 16.0 Hz), 83.4, 37.9 (d, *J* = 29.1 Hz), 24.8.

Minor Isomer E

^{1}H NMR (400 MHz, $CDCl_3$) δ 7.21-7.17 (m, 2H), 7.16-7.09 (m, 2H), 5.19 (dt, *J* = 20.9, 8.4 Hz, 1H), 3.44 (d, *J* = 23.1 Hz, 2H), 1.52 (d, *J* = 8.2 Hz, 2H), 1.17 (s, 12H).

^{13}C NMR (101 MHz, $CDCl_3$) δ 157.2 (d, *J* = 243.9 Hz), 135.5, 132.3, 129.9, 128.5, 102.5 (d, *J* = 24.5 Hz), 83.6, 33.5 (d, *J* = 29.5 Hz), 24.8.

IR (neat) cm^{-1}: 2978 (w), 2931 (w), 1704 (w), 1492 (m), 1326 (s), 1142 (s), 1089 (m), 1016 (m), 967 (m), 881 (m), 844 (m), 796 (m).

HRMS *m/z* (ESI+) calcd for $[C_{16}H_{21}BClFNaO_2]^+$ = 333.1199, found: 333.1192.

(*Z*)-4-[2-Fluoro-4-(4,4,5,5-tetramethyl-1,3,2-dioxaborolan-2-yl)but-2-en-1-yl] benzonitrile (2q)

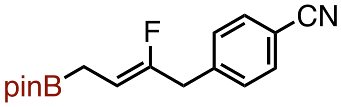

MW (g/mol): 301.17 **Molecular formula: $C_{17}H_{21}BFNO_2$**

Prepared following general sequence **GP-A**. Purification by flash column chromatography over silica gel (Pentane/Et_2O 100:00 to 85:15) afforded **2q** as a mixture of *E* and *Z* diastereoisomers in 86% yield (129 mg, 0.43 mmol).

Major Isomer Z

^{1}H NMR (400 MHz, $CDCl_3$) δ 7.58-7.52 (m, 2H), 7.42-7.35 (m, 2H), 4.77 (dt, *J* = 36.7, 7.7 Hz, 1H), 3.52 (d, *J* = 18.7 Hz, 2H), 1.60 (d, *J* = 8.4 Hz, 2H), 1.25 (s, 12H).

^{13}C NMR (101 MHz, $CDCl_3$) δ 156.1 (d, *J* = 252.4 Hz), 142.8, 132.2, 129.5, 118.9, 110.6, 103.6 (d, *J* = 15.9 Hz), 83.5, 38.7 (d, *J* = 29.2 Hz), 24.8.

Minor Isomer E

^{1}H NMR (400 MHz, $CDCl_3$) δ 7.58-7.52 (m, 2H), 7.42-7.35 (m, 2H), 5.30 (dt, *J* = 20.8, 8.5 Hz, 1H), 3.61 (d, *J* = 22.9 Hz, 2H), 1.67 (d, *J* = 7.7 Hz, 2H), 1.24 (s, 12H).

^{13}C NMR (101 MHz, $CDCl_3$) δ 156.2 (d, *J* = 244.6 Hz), 142.6 (d, *J* = 2.6 Hz), 132.3, 129.4, 118.9, 110.5, 103.5 (d, *J* = 23.6 Hz), 83.7, 34.3 (d, *J* = 29.6 Hz), 24.8.

IR (neat) cm^{-1}: 2980 (w), 1705 (w), 1619 (w), 1321 (s), 1274 (w), 1162 (m), 1142 (m), 1121 (s), 1108 (s), 1066 (s), 1019 (m), 967 (m), 844 (m).

HRMS *m/z* (ESI+) calcd for $[C_{17}H_{21}BFLiNO_2]^+$ = 308.1804, found: 308.1814.

(*Z*)-2-[3-Fluoro-4-(4-(trifluoromethyl)phenyl)but-2-en-1-yl]-4,4,5,5-tetramethyl-1,3,2-dioxaborolane (2r)

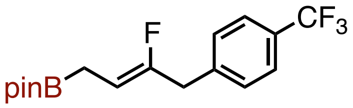

MW (g/mol): 344.16 **Molecular formula: $C_{17}H_{21}BF_4O_2$**

Prepared following general sequence **GP-A**. Purification by flash column chromatography over silica gel (Pentane/Et_2O 100:00 to 98:02) afforded **2r** as a mixture of *E* and *Z* diastereoisomers in 97% yield (167 mg, 0.49 mmol).

Major Isomer Z

^{1}H NMR (400 MHz, $CDCl_3$) δ 7.63-7.55 (m, 2H), 7.41-7.35 (m, 2H), 4.79 (dt, *J* = 36.7, 7.8 Hz, 1H), 3.52 (d, *J* = 19.0 Hz, 2H), 1.67 (d, *J* = 9.1 Hz, 2H), 1.24 (s, 12H).

^{13}C NMR (101 MHz, $CDCl_3$) δ 156.8 (d, J_F = 252.2 Hz), 141.4, 129.13, 129.08 (q, J_F = 10.0 Hz), 125.4 (q, J_F = 3.9 Hz), 124.4 (q, J_F = 271.8 Hz), 103.1 (d, J_F = 16.1 Hz), 83.6, 38.5 (d, J_F = 29.0 Hz), 24.9.

Minor Isomer E

^{1}H NMR (400 MHz, $CDCl_3$) δ 7.63-7.55 (m, 2H), 7.41-7.35 (m, 2H), 5.31 (dt, *J* = 20.8, 8.5 Hz, 1H), 3.60 (d, *J* = 22.7 Hz, 1H), 1.59 (d, *J* = 8.5 Hz, 1H), 1.24 (s, 12H).

^{13}C NMR (101 MHz, $CDCl_3$) δ 156.9 (d, J_F = 245.7 Hz), 141.3, 129.1 (q, J_F = 10.0 Hz), 129.0, 125.4 (q, J_F = 3.9 Hz), 124.4 (q, J_F = 271.8 Hz), 103.1 (d, *J* = 23.7 Hz), 83.8, 34.1 (d, J_F = 29.6 Hz), 24.7.

IR (neat) cm^{-1}: 2980 (w), 1620 (w), 1372 (w), 1325 (s), 1124 (m), 1067 (m), 1020 (w), 968 (w), 882 (w), 846 (w), 673 (w).

HRMS *m/z* (ESI+) calcd for $[C_{17}H_{21}BF_4LiO_2]^+$ = 351.1725, found: 351.1739.

(*Z*)-2-[3-Fluoro-4-(4-methoxyphenyl)but-2-en-1-yl]-4,4,5,5-tetramethyl-1,3,2-dioxa-borolane (2s)

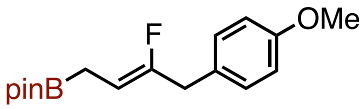

MW (g/mol): 306.18 **Molecular formula: $C_{17}H_{24}BFO_3$**

Prepared following general sequence **GP-A**. Purification by flash column chromatography over silica gel (Pentane/Et_2O 100:00 to 95:05) afforded **2s** as a mixture of *E* and *Z* diastereoisomers in 98% yield (150 mg, 0.49 mmol).

Major Isomer Z

^{1}H NMR (400 MHz, $CDCl_3$) δ 7.15-7.05 (m, 2H), 6.81-6.72 (m, 2H), 4.61 (dt, *J* = 36.9, 7.8 Hz, 1H), 3.72 (s, 3H), 3.33 (d, *J* = 18.1 Hz, 2H), 1.58 (d, *J* = 8.5 Hz, 2H), 1.17 (s, 12H).

^{13}C NMR (101 MHz, $CDCl_3$) δ 158.3, 158.1 (d, *J* = 252.6 Hz), 129.7, 129.1, 113.8, 101.5 (d, *J* = 16.4 Hz), 83.30, 55.2, 37.71 (d, *J* = 28.9 Hz), 24.8.

Minor Isomer E

^{1}H NMR (400 MHz, CDCl3) δ 7.15-7.05 (m, 3H), 6.81-6.72 (m, 3H), 5.15 (dt, *J* = 21.0, 8.4 Hz, 1H), 3.71 (s, 3H), 3.42 (d, *J* = 23.4 Hz, 2H), 1.54 (d, *J* = 8.4 Hz, 2H), 1.18 (s, 12H).

^{13}C NMR (101 MHz, CDCl3) δ 158.3, 158.0 (d, *J* = 244.7 Hz), 129.5, 129.0, 113.9, 101.7 (d, *J* = 24.4 Hz), 83.5, 55.2, 33.2 (d, *J* = 29.3 Hz), 24.8.

IR (neat) cm^{-1}: 2977 (w), 1703 (w), 1612 (w), 1511 (s), 1465 (m), 1326 (s), 1245 (s), 1175 (m), 1142 (s), 1035 (m), 967 (m), 845 (m), 760 (w).

HRMS *m/z* (ESI+) calcd for $[C_{17}H_{24}BFNaO_3]^+$ = 329.1695, found: 329.1691.

(*Z*)-2-[3-Fluoro-4-(3-methoxyphenyl)but-2-en-1-yl]-4,4,5,5-tetramethyl-1,3,2-dioxa-borolane (2t)

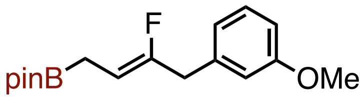

MW (g/mol): 306.18 **Molecular formula: $C_{17}H_{24}BFO_3$**

Prepared following general sequence **GP-A**. Purification by flash column chromatography over silica gel (Pentane/Et_2O 100:00 to 95:05) afforded **2t** as a mixture of *E* and *Z* diastereoisomers in 92% yield (141 mg, 0.46 mmol)

Major Isomer Z

^{1}H NMR (400 MHz, $CDCl_3$) δ 7.21 (t, *J* = 7.8 Hz, 1H), 6.99-6.69 (m, 3H), 4.73 (ddd, *J* = 36.8, 8.2, 7.4 Hz, 1H), 3.80 (s, 3H), 3.45 (d, *J* = 18.5 Hz, 2H), 1.66 (d, *J* = 7.7 Hz, 3H), 1.25 (s, 12H).

^{13}C NMR (101 MHz, $CDCl_3$) δ 159.6, 157.6 (d, *J* = 252.6 Hz), 138.6, 129.3, 121.1, 114.3, 112.0, 102.1 (d, *J* = 16.2 Hz), 83.3, 55.1, 38.6 (d, *J* = 28.9 Hz), 24.7.

Minor Isomer E

^{1}H NMR (400 MHz, $CDCl_3$) δ 7.21 (t, *J* = 7.8 Hz, 1H), 6.99-6.69 (m, 3H), 5.25 (dt, *J* = 20.8, 8.3 Hz, 1H), 3.79 (s, 3H), 3.53 (d, *J* = 23.4 Hz, 1H), 1.61 (d, *J* = 8.6 Hz, 1H), 1.25 (s, 12H).

^{13}C NMR (101 MHz, $CDCl_3$) δ 159.7, 157.6 (d, *J* = 245.0 Hz), 138.5, 129.4, 120.9, 114.2, 111.9, 102.2 (d, *J* = 24.2 Hz), 83.5, 55.1, 34.1 (d, *J* = 29.3 Hz), 24.7.

IR (neat) cm^{-1}: 2975 (w), 1701 (w), 1615 (w), 1516 (s), 1461 (m), 1328 (s), 1242 (s), 1177 (m), 1140 (s), 1030 (m), 969 (m), 846 (m), 760 (w).

HRMS *m/z* (ESI+) calcd for $[C_{17}H_{25}BFO_3]^+$ = 307.1875, found: 307.1883.

(*Z*)-2-[3-Fluoro-4-(2-methoxyphenyl)but-2-en-1-yl]-4,4,5,5-tetramethyl-1,3,2-dioxa-borolane (2u)

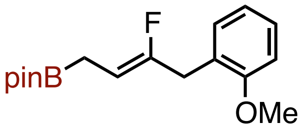

MW (g/mol): 306.18 **Molecular formula: $C_{17}H_{24}BFO_3$**

Prepared following general sequence **GP-A**. Purification by flash column chromatography over silica gel (Pentane/Et_2O 100:00 to 95:05) afforded **2u** as a mixture of *E* and *Z* diastereoisomers in 85% yield (130 mg, 0.43 mmol).

Major Isomer Z

^{1}H NMR (400 MHz, $CDCl_3$) δ 7.25-7.17 (m, 2H), 6.95-6.82 (m, 2H), 4.67 (dt, *J* = 37.2, 7.8 Hz, 1H), 3.82 (s, 3H), 3.49 (d, *J* = 17.9 Hz, 2H), 1.66 (d, *J* = 7.6 Hz, 2H), 1.25 (s, 12H).

^{13}C NMR (101 MHz, $CDCl_3$) δ 157.5 (d, *J* = 252.4 Hz), 157.4, 129.8, 127.7, 125.6, 120.4, 110.4, 101.7 (d, *J* = 16.2 Hz), 83.3, 55.4, 32.2 (d, *J* = 29.2 Hz), 24.8.

Minor Isomer E

^{1}H NMR (400 MHz, $CDCl_3$) δ 7.25-7.17 (m, 2H), 6.95-6.82 (m, 2H), 5.25 (dt, *J* = 21.1, 8.2 Hz, 1H), 3.83 (s, 3H), 3.55 (d, *J* = 23.5 Hz, 2H), 1.63 (d, *J* = 8.4 Hz, 2H), 1.24 (s, 4H).

^{13}C NMR (101 MHz, $CDCl_3$) δ 157.4 (d, *J* = 244.0 Hz), 157.3, 129.6, 127.7, 125.1, 120.4, 110.2, 102.2 (d, *J* = 24.3 Hz), 83.4, 55.3, 28.1 (d, *J* = 29.2 Hz), 24.8.

IR (neat) cm^{-1}: 2971 (w), 1704 (w), 1618 (w), 1513 (s), 1460 (m), 1325 (s), 1243 (s), 1176 (m), 1146 (s), 1038 (m), 970 (m), 846 (m), 764 (w).

HRMS *m/z* (ESI+) calcd for $[C_{17}H_{24}BFNaO_3]^+$ = 329.1695, found: 329.1691.

(*Z*)-2-[3-Fluoro-4-(furan-2-yl)but-2-en-1-yl]-4,4,5,5-tetramethyl-1,3,2-dioxaborolane (2v)

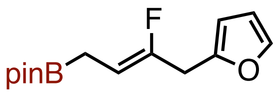

MW (g/mol): 306.18 **Molecular formula: $C_{14}H_{20}BFO_3$**

Prepared following general sequence **GP-A**. Purification by flash column chromatography over silica gel (Pentane/Et_2O 100:00 to 98:02) afforded **2v** as an inseparable mixture of *E* and *Z* diastereoisomers in 80% yield (106 mg, 0.40 mmol).

Major Isomer Z

^{1}H NMR (400 MHz, $CDCl_3$) δ 7.36-7.29 (m, 1H), 6.32-6.27 (m, 1H), 6.17-6.10 (m, 1H), 4.74 (dtt, *J* = 36.6, 7.8, 0.8 Hz, 1H), 3.50 (d, *J* = 16.6 Hz, 2H), 1.67 (d, *J* = 5.9 Hz, 2H), 1.25 (s, 12H).

^{13}C NMR (101 MHz, $CDCl_3$) δ 155.1 (d, *J* = 252.7 Hz), 150.8, 141.5, 110.4, 106.7, 102.3 (d, *J* = 15.7 Hz), 83.4, 31.5 (d, *J* = 30.5 Hz), 24.7.

Minor Isomer E

^{1}H NMR (400 MHz, $CDCl_3$) δ 7.36-7.29 (m, 1H), 6.32-6.27 (m, 1H), 6.17-6.10 (m, 1H), 5.27 (dt, *J* = 20.6, 8.4 Hz, 1H), 3.57 (d, *J* = 21.5 Hz, 2H), 1.57 (d, *J* = 8.3 Hz, 2H), 1.24 (s, 12H).

^{13}C NMR (101 MHz, $CDCl_3$) δ 155.1 (d, *J* = 245.4 Hz), 150.5 (d, *J* = 2.5 Hz), 141.4, 110.4, 106.4, 102.8 (d, *J* = 23.6 Hz), 83.5, 27.4 (d, *J* = 30.4 Hz), 24.7.

IR (neat) cm^{-1}: 2979 (m), 1708 (w), 1506 (w), 1329 (s), 1144 (s), 1010 (m), 968 (m), 883 (m), 845 (m), 731 (m), 600 (w).

HRMS *m/z* (ESI+) calcd for $[C_{14}H_{21}BFO_3]^+$ = 267.1562, found: 267.1567.

(*E*)-2-(3-Fluoro-2-phenylbut-2-en-1-yl)-4,4,5,5-tetramethyl-1,3,2-dioxaborolane (2w)

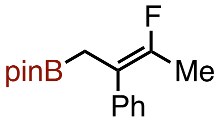

MW (g/mol): 276.16 **Molecular formula: $C_{16}H_{22}BFO_2$**

Prepared following general sequence **GP-A**. Purification by flash column chromatography over silica gel (Pentane/Et_2O 100:00 to 98:02) afforded **2w** as a mixture of *E* and *Z* diastereoisomers in 94% yield (130 mg, 0.47 mmol).

Major Isomer E

^{1}H NMR (400 MHz, C_6D_6) δ 7.68-7.56 (m, 1H), 7.30-7.01 (m, 4H), 1.92 (s, 2H), 1.87 (d, *J* = 17.2 Hz, 3H), 0.93 (s, 12H).

^{13}C NMR (101 MHz, C_6D_6) δ 151.8 (d, *J* = 248.3 Hz), 139.5 (d, *J* = 1.2 Hz), 128.8 (d, *J* = 3.8 Hz), 128.0, 126.6, 113.7 (d, *J* = 14.1 Hz), 83.2, 24.6, 15.6 (d, *J* = 31.6 Hz).

Minor Isomer Z

^{1}H NMR (400 MHz, C_6D_6) δ 7.68-7.56 (m, 1H), 7.30-7.01 (m, 4H), 2.29 (d, *J* = 2.4 Hz, 2H), 1.73 (dt, *J* = 17.8, 1.2 Hz, 3H), 0.99 (s, 12H).

^{13}C NMR (101 MHz, C_6D_6) δ 153.3 (d, *J* = 246.8 Hz), 141.2 (d, *J* = 9.2 Hz), 129.1 (d, *J* = 3.0 Hz), 128.2, 126.7, 115.9 (d, *J* = 20.7 Hz), 83.1, 24.7, 15.6 (d, *J* = 30.7 Hz).

IR (neat) cm^{-1}: 2978 (w), 1748 (w), 1380 (m), 1371 (m), 1344 (s), 1327 (s), 1290 (w), 1194 (m), 1142 (s), 966 (m), 881 (m), 845 (m), 766 (m), 696 (s).

HRMS *m/z* (ESI+) calcd for $[C_{16}H_{22}BFNaO_2]^+$ = 299.1589, found: 299.1581.

(*Z*)-2-[3-Fluoro-4-(naphthalen-1-yl)but-2-en-1-yl]-4,4,5,5-tetramethyl-1,3,2-dioxa-borolane (2x)

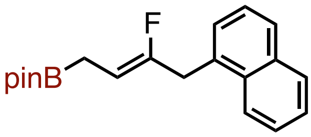

MW (g/mol): 326.18 **Molecular formula: $C_{20}H_{24}BFO_2$**

Prepared following general sequence **GP-A**. Purification by flash column chromatography over silica gel (Pentane/Et_2O 100:00 to 98:02) afforded **2x** as a mixture of *E* and *Z* diastereoisomers in 99% yield (161 mg, 0.50 mmol).

Major Isomer Z

^{1}H NMR (400 MHz, $CDCl_3$) δ 8.04 (dq, *J* = 7.6, 0.8 Hz, 1H), 7.89-7.83 (m, 1H), 7.76 (dd, *J* = 7.1, 2.5 Hz, 1H), 7.55-7.40 (m, 4H), 4.67 (dt, *J* = 37.2, 7.8 Hz, 1H), 3.94 (d, *J* = 15.0 Hz, 2H), 1.65 (d, *J* = 6.8 Hz, 2H), 1.23 (s, 12H).

^{13}C NMR (101 MHz, $CDCl_3$) δ 157.4 (d, *J* = 252.6 Hz), 133.8, 132.8 (d, *J* = 2.2 Hz), 132.0, 128.6, 127.4, 127.0, 126.2, 125.9, 125.5 (d, *J* = 3.9 Hz), 123.8, 102.5 (d, *J* = 16.1 Hz), 83.3, 35.7 (d, *J* = 29.4 Hz), 24.7.

Minor Isomer E

^{1}H NMR (400 MHz, $CDCl_3$) δ 8.04 (dq, *J* = 7.6, 0.8 Hz, 1H), 7.89-7.83 (m, 1H), 7.76 (dd, *J* = 7.1, 2.5 Hz, 1H), 7.55-7.40 (m, 4H), 5.37 (dt, *J* = 21.1, 8.4 Hz, 1H), 4.02 (d, *J* = 21.4 Hz, 2H), 1.67 (d, *J* = 5.8 Hz, 2H), 1.24 (s, 12H).

^{13}C NMR (101 MHz, $CDCl_3$) δ 157.0 (d, *J* = 244.6 Hz), 133.7, 132.4 (d, *J* = 1.5 Hz), 132.0, 128.7, 127.3, 127.0, 126.2, 126.0, 125.5f (d, *J* = 4.1 Hz), 123.4, 102.9 (d, *J* = 24.5 Hz), 83.5, 31.4 (d, *J* = 29.6 Hz), 24.8.

IR (neat) cm^{-1}: 2977 (m), 1704 (m), 1467 (w), 1350 (s), 1327 (s), 1273 (m), 1166 (m), 1143 (s), 967 (m), 882 (m), 846 (m), 789 (s).

HRMS *m/z* (ESI+) calcd for $[C_{20}H_{24}BFNaO_2]^+$ = 349.1746, found: 349.1748.

Dimethyl 4-[2-(4,4,5,5-tetramethyl-1,3,2-dioxaborolan-2-yl)propylidene]heptanedioate (4a)

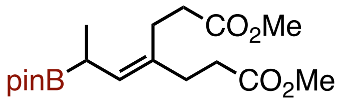

MW (g/mol): 354.25 **Molecular formula: $C_{18}H_{31}BO_6$**

Prepared following general sequence **GP-A**. Purification by flash column chromatography over silica gel (PE/EtOAc 95:05 to 85:15) afforded **4a** as a colorless oil in 88% yield (156 mg, 0.44 mmol).

^{1}H NMR (400 MHz, $CDCl_3$) δ 5.13 (d, J = 10.1 Hz, 1H), 3.66 (s, 2H), 3.65 (s, 3H), 2.47-2.25 (m, 8H), 2.01 (dq, J = 10.3, 7.3 Hz, 1H), 1.20 (s, 10H), 1.00 (d, J = 7.2 Hz, 3H).

^{13}C NMR (101 MHz, $CDCl_3$) δ 173.7, 173.7, 133.9, 129.7, 83.0, 51.5, 51.5, 33.2, 33.0, 31.6, 25.6, 24.6, 24.5, 16.2.

IR (neat) cm^{-1}: 2983 (w), 2920 (w), 1743 (s), 1431 (w), 1373 (m), 1325 (s), 1269 (m), 1215 (m), 1138 (s), 1089 (w), 970 (m), 882 (m), 847 (m).

HRMS *m/z* (ESI+) calcd for $[C_{18}H_{32}BO_6]^+$ = 355.2286, found: 355.2295.

Methyl 3-(4,4,5,5-tetramethyl-1,3,2-dioxaborolan-2-yl)cyclohex-1-en-1-yl)propanoate (4b)

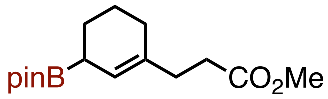

MW (g/mol): 294.19 **Molecular formula: $C_{16}H_{27}BO_4$**

Methyl 3-(1-nitrocyclohex-2-en-1-yl)propanoate **3b** was prepared following general sequence **GP-A** however, we were not able to isolate it as it co-eluted with the starting material. Nonetheless, we were able to determine an NMR yield and as well as a thoretical isolated yield after oxidising **4b** to the corresponding alcohol, which is described in the literature.[1] The spectroscopic data of the product were identical with those reported in the literature.

Methyl 3-(6-oxo-3-vinyltetrahydro-2*H*-pyran-3-yl)propanoate (5)

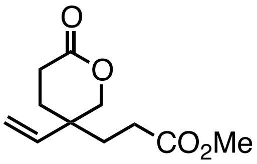

MW (g/mol): 212.24 **Molecular formula: $C_{11}H_{16}O_4$**

Prepared following general sequence **GP-D** starting from allylborane **2a** and *paraformaldehyde*. Purification by flash column chromatography over silica gel (PE/Et_2O = 60:40) afforded **5** in 84% yield as a pale yellow oil.

^{1}H NMR (400 MHz, $CDCl_3$) δ 5.62 (dd, *J* = 17.7, 10.9 Hz, 1H), 5.33 (d, *J* = 10.9 Hz, 1H), 5.19 (d, *J* = 17.7 Hz, 1H), 4.26 (dd, *J* = 11.4, 2.1 Hz, 1H), 4.09 (d, *J* = 11.4 Hz, 1H), 3.67 (s, 3H), 2.59-2.54 (m, 2H), 2.27 (ddd, *J* = 8.8, 7.0, 1.4 Hz, 2H), 1.92 (dtd, *J* = 13.8, 6.5, 2.1 Hz, 1H), 1.85-1.69 (m, 3H).

^{13}C NMR (101 MHz, $CDCl_3$) δ 173.5, 170.6, 138.8, 117.4, 74.9, 52.0, 38.6, 31.8, 29.1, 28.7, 27.2.

IR (neat) cm^{-1} : 2953 (w), 1730 (s), 1550 (w), 1437 (w), 1363 (w), 1256 (w), 1195 (m), 1169 (m), 1006 (w), 926 (w), 852 (w), 673 (w).

HRMS *m/z* (ESI+) calcd for $C_{11}H_{16}NaO_4^+$ [M^+]: 235.0941; Found: 235.0928.

Methyl 3-[2-(4-chlorophenyl)-6-oxo-3-vinyltetrahydro-2*H*-pyran-3-yl]propanoate (6)

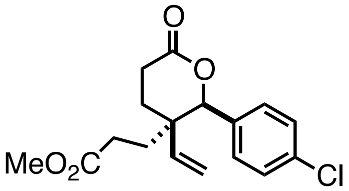

MW (g/mol): 322.78 **Molecular formula: $C_{17}H_{19}ClO_4$**

Prepared following general sequence **GP-B** starting from allylborane **2a** and *p*-chlorobenzaldehyde. Purification by flash column chromatography over silica gel (PE/Et_2O = 80:20) afforded **6** in 72% yield as a separable mixture of *cis/trans* isomers (*cis/trans* = 55:45).

Cis isomer

^{1}H NMR (400 MHz, $CDCl_3$) δ 7.34 (d, *J* = 8.6 Hz, 2H), 7.24 (d, *J* = 8.6 Hz, 2H), 5.55 (dd, *J* = 17.7, 11.0 Hz, 1H), 5.35 (d, *J* = 11.0 Hz, 1H), 5.15 (s, 1H), 5.04 (d, *J* = 17.7 Hz, 1H), 3.63 (s, 3H), 2.85-2.69 (m, 2H), 2.20-2.09 (m, 3H), 1.88-1.79 (m, 1H), 1.72 (ddd, *J* = 13.7, 10.0, 6.4 Hz, 1H), 1.50 (ddd, *J* = 13.7, 10.4, 5.9 Hz, 1H).

^{13}C NMR (101 MHz, $CDCl_3$) δ 173.6, 170.9, 135.1, 134.7, 133.4, 129.6, 128.3, 119.0, 86.6, 51.9, 43.0, 31.8, 28.5, 27.1, 26.0.

Trans isomer

^{1}H NMR (400 MHz, $CDCl_3$) δ 7.33 (d, *J* = 8.6 Hz, 2H), 7.20 (d, *J* = 8.6 Hz, 2H), 5.44 (dd, *J* = 17.4, 10.9 Hz, 1H), 5.32 (d, *J* = 10.9 Hz, 1H), 5.16 (d, *J* = 17.4, 1H), 5.15 (s, 1H), 3.62 (s, 3H), 2.80 (ddd, *J* = 18.7, 7.2, 4.3 Hz, 1H), 2.67 (ddd, *J* = 18.7, 10.1, 7.2 Hz, 1H), 1.90-1.82 (m, 2H), 1.52-1.43 (m, 1H).

^{13}C NMR (101 MHz, $CDCl_3$) δ 173.5, 170.3, 139.9, 134.5, 133.7, 129.3, 128.3, 117.5, 87.6, 52.0, 41.6, 28.4, 27.0, 25.9, 24.5.

IR (neat) cm^{-1}: 2924 (w), 1736 (s), 1493 (w), 1436 (w), 1349 (w), 1196 (m), 1090 (w), 1057 (w), 1014 (w), 930 (w), 847 (w), 698 (w).

HRMS *m/z* (ESI+) calcd for $C_{17}H_{19}ClNaO_4^+$ [M^+]: 345.0864 ; Found: 345.0856.

1-(4-Chlorophenyl)-2-fluoro-2-(3-methoxybenzyl)but-3-en-1-ol (7)

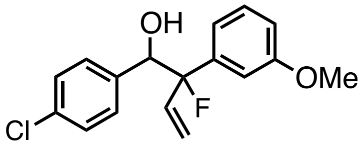

MW (g/mol): 320.79 **Molecular formula: $C_{18}H_{18}ClFO_2$**

Prepared following general sequence **GP-C** starting from allylborane **2t**. Purification by flash column chromatography over silica gel (PE/Et_2O = 100:0 to 90:10) afforded **7** in 75% yield as an separable mixture of *syn/anti* isomers (major/minor = 68:32).

Major isomer

^{1}H NMR (400 MHz, $CDCl_3$) δ 7.31 (s, 4H), 7.19 (td, *J* = 7.6, 0.9 Hz, 1H), 6.81-6.75 (m, 3H), 5.79 (ddd, *J* = 22.1, 17.3, 11.3 Hz, 1H), 5.19-5.15 (m, 1H), 5.14 (dd, *J* = 17.3, 1.3 Hz, 1H), 4.70 (dd, *J* = 10.2, 2.6 Hz, 1H), 3.78 (s, 3H), 3.24 (dd, *J* = 20.5, 14.4 Hz, 1H), 2.83 (dd, *J* = 29.9, 14.4 Hz, 1H), 2.39 (d, *J* = 3.3 Hz, 1H).

^{13}C NMR (101 MHz, $CDCl_3$) δ 159.4, 137.3 (d, *J* = 2.6 Hz), 137.0, 135.4 (d, *J* = 20.5 Hz), 134.0, 129.4, 129.3, 129.1, 128.2, 123.3 (d, *J* = 1.4 Hz), 117.2 (d, *J* = 11.5 Hz), 116.6 (d, *J* = 1.5 Hz), 112.2, 98.6 (d, *J* = 183.3 Hz), 76.6 (d, *J* = 27.6 Hz), 55.3, 40.8 (d, *J* = 20.9 Hz).

Minor isomer

^{1}H NMR (400 MHz, $CDCl_3$) δ 7.32 (s, 4H), 7.18 (td, *J* = 15.4, 0.8 Hz, 1H). 6.81-6.76 (m, 3H), 5.77-5.62 (m, 1H), 5.19-5.09 (m, 1H), 4.76 (dd, *J* = 14.9, 3.4 Hz, 1H), 3.78 (s, 3H), 3.05 (s, 1H), 3.00 (d, *J* = 5.8 Hz, 1H), 2.48 (d, *J* = 4.2 Hz, 1H).

^{13}C NMR (101 MHz, $CDCl_3$) δ 159.4, 137.3 (d, *J* = 3.0 Hz), 136.8 (d, *J* = 3.7 Hz), 134.7 (d, *J* = 20.7 Hz), 129.5, 129.5, 129.0, 128.4, 123.3 (d, *J* = 1.2 Hz), 117.1 (d, *J* = 11.6 Hz), 116.6 (d, *J* = 2.3 Hz), 112.2, 99.2 (d, *J* = 184.4 Hz), 77.1 (d, *J* = 23.5 Hz), 55.3, 41.4 (d, *J* = 22.3 Hz).

IR (neat) cm^{-1} : 3466 (w), 2935 (w), 1601 (m), 1584 (m), 1489 (s), 1454 (w), 1438 (w), 1412 (w), 1263 (s), 1191 (w), 1155 (m), 1090 (m), 1047 (m), 1014 (m), 992 (w), 932 (w), 838 (w), 781 (w), 744 (w), 728 (w), 695 (w).

HRMS *m/z* (ESI+) calcd for $C_{18}H_{18}ClFNaO_2^+$ [M^+]: 343.0872 ; Found: 343.0877.

1-Methoxy-4-(1-phenylbuta-1,3-dien-2-yl) benzene (8)

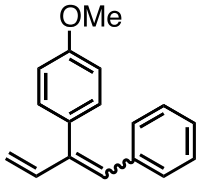

MW (g/mol): 236.31 **Molecular formula: $C_{17}H_{16}O$**

Prepared following general sequence **GP-G** starting from allylborane **2o** Purification by flash column chromatography over silica gel (Pentane/Et_2O 100:0 to 95:5) afforded **8** in 90% yield as an inseparable mixture of *E* and *Z* isomers (*E/Z* = 67:33).

¹H NMR (400 MHz, $CDCl_3$) δ 7.45-7.24 (m, 3H), 7.16-7.10 (m, 3H), 7.06 (dd, *J* = 17.4, 10.8 Hz, 1H), 6.96 (m, 3H), 6.60 (s, 1H), 5.40 (dt, *J* = 10.8, 1.7 Hz, 1H), 5.30 (dd, *J* = 17.4, 1.7 Hz, 1H), 3.87 (s, 3H).

¹³C NMR (101 MHz, $CDCl_3$) δ 159.2, 158.9, 142.1, 141.5, 140.9, 137.6, 137.0, 135.1, 134.3, 131.6, 130.8, 130.4, 130.2, 130.0, 129.7, 129.5, 128.3, 128.1, 127.0, 126.9, 119.7, 116.4, 114.3, 113.6, 55.3..

IR (neat) cm^{-1} : 2929 (w), 1677 (w), 1605 (m), 1511 (s), 1450 (w), 1248 (s), 1176 (m), 1030 (m), 835 (w), 698 (w).

HRMS *m/z* (ESI+) calcd for $C_{17}H_{17}O^+$ [M^+]: 237.1274 ; Found: 237.1271

Dimethyl 4-(1-(4-chlorophenyl)-2-hydroxyethylidene)heptanedioate (9)

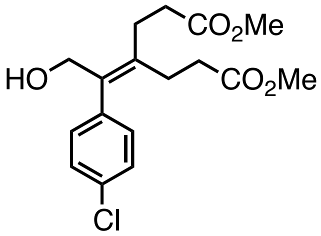

MW (g/mol): 340.80 **Molecular formula: $C_{17}H_{21}ClO_5$**

Prepared following general sequence **GP-E** starting from allylborane **2j**. Purification by flash column chromatography over silica gel (PE/Et_2O = 60:40) afforded **9** in 83% yield as a colorless oil.

¹H NMR (400 MHz, $CDCl_3$) δ 7.31 (d, *J* = 8.4 Hz, 2H), 7.06 (d, *J* = 8.4 Hz, 2H), 4.32 (s, 2H), 3.71 (s, 3H), 3.60 (s, 3H), 2.62-2.53 (m, 4H), 2.28-2.21 (m, 4H), 2.11 (br. s, 1H).

¹³C NMR (101 MHz, $CDCl_3$) δ 173.9, 173.2, 139.6, 138.4, 136.6, 133.0, 130.2, 128.8, 63.2, 52.0, 51.8, 32.9, 32.8, 27.4, 25.6.

IR (neat) cm^{-1}: 3473 (w), 2952 (w), 1734 (s), 1488 (w), 1437 (w), 1261 (w), 1170 (m), 1090 (w), 1014 (w), 834 (w).

HRMS *m/z* (ESI+) calcd for $C_{17}H_{21}ClNaO_5^+$ [M^+]: 363.0970 ; Found: 363.0969.

Methyl-3-[2-(4-chlorophenyl)-3-(1-(4-chlorophenyl)vinyl)-6-oxotetrahydro-2*H*-pyran-3-yl] propanoate (10)

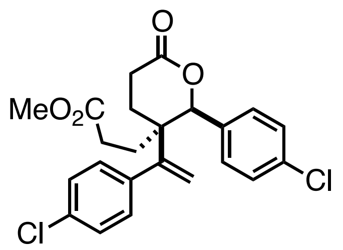

MW (g/mol): 433.32 **Molecular formula: $C_{23}H_{22}Cl_2O_4$**

Prepared following general sequence **GP-B** starting from allylborane **2i**. Purification by flash column chromatography over silica gel (PE/Et_2O = 70:30) afforded **10** in 71% yield as a mixture of *cis/trans* isomers (*cis/trans* = 98:2).

<u>*Cis isomer*</u>

^{1}H NMR (400 MHz, $CDCl_3$) δ 7.33 (d, *J* = 8.5 Hz, 2H), 7.32 (d, *J* = 8.5 Hz, 2H), 7.14 (d, *J* = 8.5 Hz, 2H), 7.07 (d, *J* = 8.5 Hz, 2H), 5.36 (s, 1H), 5.28 (d, *J* = 1.8 Hz, 1H), 5.25 (s, 1H), 3.65 (s, 3H), 2.85-2.68 (m, 2H), 2.38 (ddd, *J* = 16.0, 11.4, 4.6 Hz, 1H), 2.27-2.17 (m, 1H), 2.06 (dddd, *J* = 14.2, 8.0, 4.0, 1.8 Hz, 1H), 1.84 (ddd, *J* = 14.2, 11.4, 4.6 Hz, 1H), 1.75 (dt, *J* = 14.6, 8.7 Hz, 1H), 1.40 (ddd, *J* = 14.2, 11.4, 5.7 Hz, 1H).

^{13}C NMR (101 MHz, $CDCl_3$) δ 173.3, 170.0, 147.1, 138.9, 134.8, 134.6, 134.0, 130.1, 129.2, 128.7, 128.6, 121.6, 85.1, 52.0, 45.3, 28.9, 28.5, 27.4, 23.6.

IR (neat) cm^{-1}: 2917 (w), 2849 (w), 1779 (w), 1735 (s), 1489 (w), 1436 (w), 1373 (w), 1250 (w), 1175 (m), 1091 (w), 1059 (w), 1013 (w), 924 (w), 836 (w), 730 (w).

HRMS (EI+): Calculated for $C_{17}H_{22}Cl_2NaO_4^+$ [M^+]: 455.0787; Found: 455.0802.

Methyl 3-[2-(1-(4-chlorophenyl)vinyl)-5-oxotetrahydrofuran-2-yl]propanoate (11)

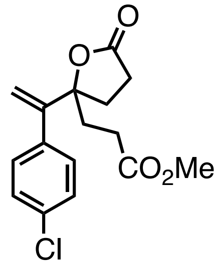

MW (g/mol): 308.76 **Molecular formula: $C_{16}H_{17}ClO_4$**

Prepared following general sequence **GP-F** starting from allylborane **2j**. Purification by flash column chromatography over silica gel (PE/Et_2O = 60:40 to 50:50) afforded **11** in 57% yield as a colorless oil.

^{1}H NMR (400 MHz, $CDCl_3$) δ 7.31 (d, *J* = 8.5 Hz, 2H), 7.19 (d, *J* = 8.5 Hz, 2H), 5.51 (s, 1H), 5.26 (s, 1H), 3.66 (s, 3H), 2.61-2.34 (m, 6H), 2.18-2.12 (m, 2H).

^{13}C NMR (101 MHz, $CDCl_3$) δ 175.7, 173.3, 148.1, 137.6, 134.3, 129.6, 128.8, 117.5, 88.4, 52.0, 33.5, 32.9, 28.8, 28.5.

IR (neat) cm^{-1} : 2952 (w), 1777 (s), 1733 (s), 1593 (w), 1490 (w), 1437 (w), 1394 (w), 1304 (w), 1170 (s), 1089 (w), 1054 (w), 1013 (w), 938 (w), 838 (w), 737 (w), 678 (w).

HRMS *m/z* (ESI+) calcd for $C_{16}H_{17}ClNaO_4^+$ [M$^+$]: 331.0708 ; Found: 331.0708.

4,4'-[2-(Phenylsulfonyl)-2-vinylpropane-1,3-diyl]bis(methylbenzene) (12a)

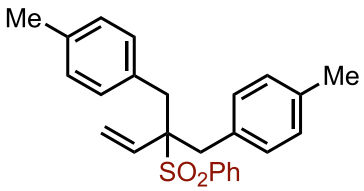

MW (g/mol): 390,54 **Molecular formula: $C_{25}H_{26}SO_2$**

Prepared following general sequence **GS5**. Purification by flash chromatography over silica gel (PE/Et_2O = 90:10) afforded **12a** as a white solid in 65% yield.

^{1}H NMR (400 MHz, $CDCl_3$) δ 7.78-7.76 (d, *J* = 7.5 Hz, 2H), 7.62-7.58 (t, *J* = 7.5 Hz, 1H), 7.49-7.44 (t, *J* = 7.7 Hz, 2H), 7.03 (q, *J* = 8.2 Hz, 8H), 5.96 (dd, *J* = 17.7, 11.1 Hz, 1H), 5.50 (d, *J* = 11.1 Hz, 1H), 5.30 (d, *J* = 17.7 Hz, 1H), 3.27(s, 2H), 3.26 (s, 2H), 2.30 (s, 6H).

^{13}C NMR (101 MHz, $CDCl_3$) δ 136.6, 136.4, 134.4, 133.6, 132.3, 131.4, 131.2, 128.8, 128.4, 121.7, 72.1, 36.9, 21.2.

IR (neat) cm^{-1}: 3023, 2921, 1514, 1445, 1299, 1138, 1081, 931, 808, 758, 731, 709, 690, 607, 553.

HRMS *m/z* (ESI-TOF) calcd for $[C_{25}H_{27}O_2S]^+$ = 391.1726, found: 391.1721.

Melting point: 97-98°C.

5,5'-[2-(Phenylsulfonyl)-2-vinylpropane-1,3-diyl]bis(1,3-dimethoxybenzene) (12b)

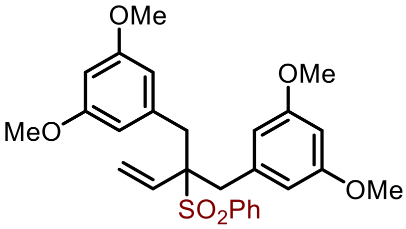

MW (g/mol): 482.59 **Molecular formula: $C_{27}H_{30}SO_6$**

Prepared following general sequence **GS5**. Purification by flash chromatography over silica gel (PE/Et_2O = 50:50) afforded **12b** as a colourless oil in 32% yield.

^{1}**H NMR** (400 MHz, $CDCl_3$) δ 7.82-7.79 (d, *J* = 7.8 Hz, 2H), 7.63-7.60 (t, *J* = 7.7 Hz, 1H), 7.52-7.48 (t, *J* = 7.8 Hz, 2H), 6.31 (d, *J* = 2.2 Hz, 4H), 6.29 (d, *J* = 2.2 Hz, 2H), 5.97 (dd, *J* = 17.7, 11.1 Hz, 1H), 5.55 (d, *J* = 11.1 Hz, 1H), 5.35 (d, *J* = 17.7 Hz, 1H), 3.69 (s, 12H), 3.30 (s, 2H), 3.30 (s, 2H).

^{13}**C NMR** (101 MHz, $CDCl_3$) δ 160.3, 137.6, 136.1, 134.9, 133.7, 131.4, 128.4, 121.9, 109.3, 99.2, 72.1, 54.9, 37.2.

IR (neat) cm^{-1}: 2937, 2837, 1594, 1461, 1429, 1291, 1203, 1147, 1065, 998, 930, 834, 756, 722, 689, 610.

HRMS *m/z* (ESI-TOF) calcd for $[C_{27}H_{31}O_6S]^+$ = 483.1836, found: 483.1841.

[(4-Vinylhepta-1,6-dien-4-yl]sulfonyl)benzene (12c)

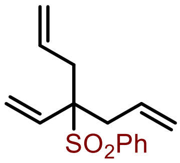

MW (g/mol): 262.37 **Molecular formula: $C_{15}H_{18}SO_2$**

Prepared following general sequence **GS5**. Purification by flash chromatography over silica gel (PE/Et_2O = 95:5) afforded **12c** as a colourless oil in 69% yield.

^{1}**H NMR** (400 MHz, $CDCl_3$) δ 7.83-7.81 (d, *J* = 8.0 Hz, 2H), 7.66-7.62 (t, *J* = 7.4 Hz, 1H), 7.54-7.50 (t, *J* = 7.7 Hz, 2H), 5.93-5.83 (m, 3H), 5.39 (d, *J* = 11.0 Hz, 1H), 5.16-5.14 (m, 3H), 5.11 (q, *J* = 1.6 Hz, 1H), 5.05 (d, *J* = 17.7 Hz, 1H), 2.71 (dd, *J* = 14.8, 7.2 Hz, 2H), 2.58 (dd, *J* = 14.8, 7.0 Hz, 2H).

^{13}**C NMR** (101 MHz, $CDCl_3$) δ 135.5, 135.0, 133.8, 131.7, 130.9, 128.5, 121.0, 119.5, 69.4, 35.0.

IR (neat) cm^{-1}: 3076, 1638, 1446, 1290, 1142, 1080, 996, 920, 752, 714, 690, 616.

HRMS *m/z* (ESI-TOF) calcd for $[C_{15}H_{18}NaO_2S]^+$ = 285.0920, found: 285.0924.

[(1*E*,6*E*)-4-(Phenylsulfonyl)-4-vinylhepta-1,6-diene-1,7-diyl]dibenzene (12d)

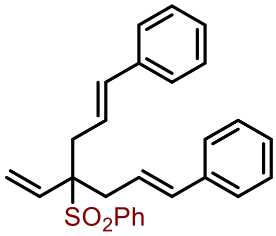

MW (g/mol): 414.56 **Molecular formula: $C_{27}H_{26}SO_2$**

Prepared following general sequence **GS5**. Purification by flash chromatography over silica gel (PE/Et_2O = 95:5) afforded **12d** as a white oil in 44% yield.

^{1}**H NMR** (400 MHz, $CDCl_3$) δ 7.81 (d, *J* = 8.1 Hz, 2H), 7.60 (t, *J* = 7.4 Hz, 1H), 7.48 (t, *J* = 7.7 Hz, 2H), 7.28-7.16 (m, 10H), 6.39 (d, *J* = 15.8 Hz, 2H), 6.22 (dt, *J* = 15.3, 7.1 Hz, 2H), 5.93 (dd, *J* = 17.7,

10.9 Hz, 1H), 5.41 (d, J = 10.9 Hz, 1H), 5.11 (d, J = 17.7 Hz, 1H), 2.86 (dd, J = 14.8, 7.3 Hz, 2H), 2.74 (dd, J = 14.8, 7.0 Hz, 2H).

^{13}C NMR (101 MHz, $CDCl_3$) δ 137.1, 135.6, 134.9, 134.5, 133.9, 131.0, 128.7, 128.5, 127.6, 126.3, 123.3, 121.4, 70.1, 34.7.

IR (neat) cm^{-1}: 1494, 1446, 1411, 1287, 1138, 1078, 965, 907, 723, 688, 610.

HRMS *m/z* (ESI-TOF) calcd for $[C_{27}H_{26}NaO_2S]^+$ = 437.1546, found: 437.1545.

[(2.8-Dimethyl-5-vinylnona-2,7-dien-5-yl)sulfonyl]benzene (12e)

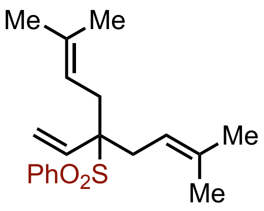

MW (g/mol): 318.48 **Molecular formula: $C_{19}H_{26}SO_2$**

Prepared following general sequence **GS5**. Purification by flash chromatography over silica gel (PE/Et_2O = 95:5) afforded **12e** as a colourless oil in 83% yield.

^{1}H NMR (400 MHz, $CDCl_3$) δ 7.77 (d, J = 7.5 Hz, 2H), 7.58 (t, J = 7.5 Hz, 1H), 7.46 (t, J = 7.7 Hz, 2H), 5.83 (dd, J = 17.6, 11.0 Hz, 1H), 5.34 (d, J = 10.9 Hz, 1H), 5.19-5.12 (m, 2H), 5.02 (d, J = 17.7 Hz, 1H), 2.60 (dd, J = 15.4, 7.3 Hz, 2H), 2.47 (dd, J = 15.4, 6.8 Hz, 2H), 1.66 (s, 6H), 1.53 (s, 6H).

^{13}C NMR (101 MHz, $CDCl_3$) δ 135.9, 135.4, 134.8, 133.5, 130.8, 128.2, 120.7, 117.6, 70.6, 29.3, 26.0, 18.0.

IR (neat) cm^{-1}: 2913, 1446, 1297, 1138, 1082, 998, 927, 758, 689.

HRMS *m/z* (ESI-TOF) calcd for $[C_{19}H_{26}NaO_2S]^+$ = 341.1546, found: 341.1512.

1-Methyl-4-[2-(phenylsulfonyl)but-3-en-1-yl]benzene (12f)

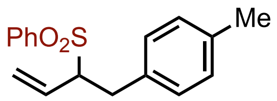

MW (g/mol): 286.39 **Molecular formula: $C_{17}H_{18}SO_2$**

Prepared following general sequence **GS5**. Purification by flash chromatography over silica gel (PE/Et_2O = 90:10) afforded **12f** as a white solid in 76% yield.

^{1}H NMR (400 MHz, $CDCl_3$) δ 7.89-7.87 (d, J = 7.5 Hz, 2H), 7.67-7.63 (t, J = 7.4 Hz, 1H), 7.57-7.53 (t, J = 7.7 Hz, 2H), 7.06 (d, J = 7.9 Hz, 2H), 6.99 (d, J = 7.9 Hz, 2H), 5.64 (ddd, J = 17.1, 10.3, 9.3 Hz, 1H), 5.16 (dd, J = 10.3, 1.0 Hz, 1H), 4.80 (dt, J = 17.1, 1.0 Hz, 1H), 3.72 (ddd, J = 11.7, 9.3, 3.3 Hz, 1H), 3.51 (dd, J = 14.0, 3.3 Hz, 1H), 2.86 (dd, J = 13.7, 11.7 Hz, 1H), 2.29 (s, 3H).

^{13}C NMR (101 MHz, CDCl$_3$) δ 137.4, 136.5, 133.9, 133.6, 129.8, 129.4, 129.4, 129.2, 129.0, 124.5, 71.5, 33.1, 21.2.

IR (neat) cm^{-1}: 2921, 1515, 1446, 1303, 1143, 1083, 1022, 998, 931, 793, 757, 712, 687.

HRMS *m/z* (ESI-TOF) calcd for $[C_{17}H_{19}O_2S]^+$ = 287.1100, found: 287.1103.

Melting point: 67-68°C.

1,3-Dimethoxy-5-[2-(phenylsulfonyl)but-3-en-1-yl]benzene (12g)

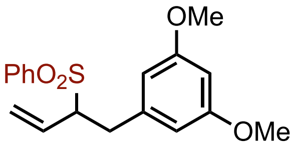

MW (g/mol): 332.41 **Molecular formula: $C_{18}H_{20}SO_4$**

Prepared following general sequence **GS5**. Purification by flash chromatography over silica gel (PE/Et$_2$O = 85:15) afforded **12g** as a colourless oil in 56% yield.

^{1}H NMR (400 MHz, CDCl$_3$) δ 7.88 (d, *J* = 7.1 Hz, 2H), 7.67-7.63 (t, *J* = 7.6 Hz, 1H), 7.55 (t, *J* = 7.6 Hz, 2H), 6.29 (d, *J* = 2.3 Hz, 1H), 6.26 (d, *J* = 2.3 Hz, 2H), 5.65 (ddd, *J* = 17.1, 10.3, 9.3 Hz, 1H), 5.17 (dd, *J* = 10.3, 0.9 Hz, 1H), 4.84 (dt, *J* = 17.1, 0.9 Hz, 1H), 3.74 (s, 6H), 3.73-3.66 (m, 1H), 3.49 (dd, *J* = 13.6, 11.4 Hz, 1H), 2.82 (dd, *J* = 13.6, 11.4Hz, 1H).

^{13}C NMR (101 MHz, CDCl$_3$) δ 160.9, 139.0, 137.3, 133.9, 129.7, 129.4, 129.0, 124.5, 107.4, 98.7, 71.2, 55.4, 33.8.

IR (neat) cm^{-1}: 2838, 1593, 1446, 1288, 1204, 1142, 1064, 992, 930, 830, 755, 724, 687, 628.

HRMS *m/z* (ESI-TOF) calcd for $[C_{18}H_{21}O_4S]^+$ = 333.1155, found: 333.1154.

1-Bromo-2-[2-(phenylsulfonyl)but-3-en-1-yl]benzene (12h)

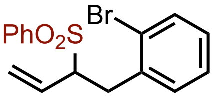

MW (g/mol): 351.26 **Molecular formula: $C_{16}H_{15}BrSO_2$**

Prepared following general sequence **GS5**. Purification by flash chromatography over silica gel (PE/Et$_2$O = 90:10) afforded **12h** as a colourless oil in 50% yield.

^{1}H NMR (400 MHz, CDCl$_3$) δ 7.92 (d, *J* = 7.0 Hz, 2H), 7.66 (t, *J* = 7.4 Hz, 1H), 7.56 (t, *J* = 7.6 Hz, 2H), 7.49 (dd, *J* = 8.0, 1.3 Hz, 1H), 7.20 (td, *J* = 7.5, 1.3 Hz, 1H), 7.12 (dd, *J* = 7.7, 1.8 Hz, 1H), 7.07 (td, *J* = 7.6, 1.9 Hz, 1H), 5.72 (dt, *J* = 17.1, 9.9 Hz, 1H), 5.17 (d, *J* = 10.2 Hz, 1H), 4.83 (d, *J* = 17.1 Hz, 1H), 3.96 (ddd, *J* = 11.3, 9.5, 3.4 Hz, 1H), 3.60 (dd, *J* = 13.6, 3.5 Hz, 1H), 3.04 (dd, *J* = 13.6, 11.4 Hz, 1H).

^{13}**C NMR** (101 MHz, $CDCl_3$) δ 137.5, 136.0, 134.0, 133.1, 132.2, 129.4, 129.1, 129.0, 128.8, 127.5, 124.8, 124.6, 68.1, 34.4.

IR (neat) cm^{-1}: 3064, 1584, 1568, 1446, 1305, 1145, 1084, 1024, 935, 750, 722, 688, 629.

HRMS *m/z* (ESI-TOF) calcd for $[C_{16}H_{16}BrO_2S]^+$ = 351.0049, found: 351.0039.

1-Iodo-2-(2-(phenylsulfonyl)but-3-en-1-yl)benzene (12i)

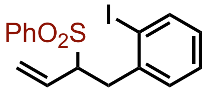

MW (g/mol): 398.26 **Molecular formula: $C_{16}H_{15}ISO_2$**

Prepared following general sequence **GS5**. Purification by flash chromatography over silica gel (PE/Et_2O = 85:15) afforded **12i** as a white oil in 60% yield.

^{1}**H NMR** (400 MHz, $CDCl_3$) δ 7.96-7.90 (m, 2H), 7.76 (dd, *J* = 7.9, 1.3 Hz, 1H), 7.65 (t, *J* = 7.4 Hz, 1H), 7.56 (t, *J* = 7.5 Hz, 2H), 7.28-7.18 (m, 1H), 7.09 (dd, *J* = 7.7, 1.7 Hz, 1H), 6.88 (td, *J* = 7.6, 1.7 Hz, 1H), 5.74 (dt, *J* = 17.0, 10.0 Hz, 1H), 5.17 (dd, *J* = 10.2, 1.0 Hz, 1H), 4.83 (dt, *J* = 17.1, 0.9 Hz, 1H), 3.95 (ddd, *J* = 11.5, 9.5, 3.5 Hz, 1H), 3.55 (dd, *J* = 13.7, 3.5 Hz, 1H), 3.07 (dd, *J* = 13.7, 11.5 Hz, 1H).

^{13}**C NMR** (101 MHz, $CDCl_3$) δ 139.8, 139.2, 137.5, 134.0, 131.6, 129.5, 129.1, 128.9, 128.8, 128.3, 124.9, 100.4, 69.2, 38.6.

IR (neat) cm^{-1}: 1446, 1306, 1146, 1084, 753, 720, 689, 626.

HRMS *m/z* (ESI+) calcd for $[C_{16}H_{16}O_2SI]^+$ = 398.9910, found: 398.9900.

(*E*)-(Hepta-1,5-dien-3-ylsulfonyl)benzene (12j)

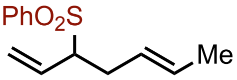

MW (g/mol): 236.33 **Molecular formula: $C_{13}H_{16}SO_2$**

Prepared following general sequence **GS5**. Purification by flash chromatography over silica gel (PE/Et_2O = 95:5) afforded **12j** as a yellow oil in 48% yield.

^{1}**H NMR** (400 MHz, $CDCl_3$) δ 7.85 (dd, *J* = 8.3, 1.4 Hz, 2H), 7.63 (t, *J* = 7.4 Hz, 1H), 7.53 (t, *J* = 7.5 Hz, 2H), 5.67-5.57 (ddd, *J* = 17.0, 10.2, 9.1 Hz, 1H), 5.57-5.47 (ddt, *J* = 15.2, 6.5, 1.3 Hz, 1H), 5.29 (dd, *J* = 10.2, 1.1 Hz, 1H), 5.26-5.22 (ddt, *J* = 8.7, 5.4, 1.6 Hz, 1H), 5.03-4.99 (d, *J* = 15.7 Hz, 1H), 3.54-3.48 (ddd, *J* = 10.7, 9.1, 3.5 Hz, 1H), 2.83-2.77 (dddt, *J* = 14.1, 7.1, 3.6, 1.3 Hz, 1H), 2.41-2.23 (dddt, *J* = 13.9, 10.8, 7.0, 1.3 Hz, 1H), 1.62 (dq, *J* = 6.5, 1.3 Hz, 3H).

^{13}**C NMR** (101 MHz, $CDCl_3$) δ 137.5, 133.8, 130.2, 129.4, 129.4, 129.0, 128.9, 125.3, 123.9, 69.8, 30.6, 24.9, 18.1.

IR (neat) cm^{-1}: 1447, 1305, 1147, 1085, 967, 722, 690.

HRMS *m/z* (ESI-TOF) calcd for $[C_{13}H_{16}NaO_2S]^+$ = 259.0763, found: 259.0760.

[(6-Methylhepta-1,5-dien-3-yl)sulfonyl]benzene (12k)

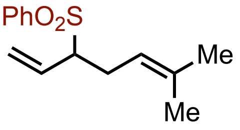

MW (g/mol): 250.36 **Molecular formula: $C_{14}H_{18}SO_2$**

Prepared following general sequence **GS5**. Purification by flash chromatography over silica gel (PE/Et_2O = 95:5) afforded **12k** as a colourless oil in 65% yield.

^{1}H NMR (400 MHz, $CDCl_3$) δ 7.84 (d, *J* = 7.6 Hz, 2H), 7.63 (t, *J* = 7.4 Hz, 1H), 7.53 (t, *J* = 7.7 Hz, 2H), 5.69-5.60 (dt, *J* = 17.1, 9.7 Hz, 1H), 5.27 (d, *J* = 10.2 Hz, 1H), 5.04-4.99 (d, *J* = 17.1 Hz, 1H), 4.98-4.94 (t, 1H), 3.49 (ddd, *J* = 10.6, 9.2, 3.5 Hz, 1H), 2.80 (m, 1H), 2.37 (m, 1H), 1.66 (s, 3H), 1.58 (s, 3H).

^{13}C NMR (101 MHz, $CDCl_3$) δ 137.6, 135.4, 133.7, 130.2, 129.3, 128.9, 123.7, 118.5, 69.9, 26.0, 25.9, 18.1.

IR (neat) cm^{-1}: 2916, 1446, 1303, 1143, 1083, 989, 931, 720, 688.

HRMS *m/z* (ESI-TOF) calcd for $[C_{14}H_{18}NaO_2S]^+$ = 273.0920, found: 273.0913.

1-(1-(Phenylsulfonyl)allyl)cyclopentan-1-ol

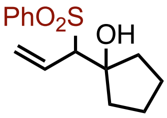

MW (g/mol): 266.36 **Molecular formula: $C_{14}H_{18}O_3S$**

Prepared following general sequence **GS5**. Purification by flash chromatography over silica gel (PE/Et_2O = 90:10) afforded the title product as a colourless oil in 85% yield.

^{1}H NMR (400 MHz, $CDCl_3$) δ 7.84-7.82 (dd, *J* = 8.4, 1.3 Hz, 2H), 7.65-7.61 (t, *J* = 7.4 Hz, 1H), 7.55-7.51 (t, *J* = 7.6 Hz, 2H), 5.85 (dt, *J* = 17.1, 10.3 Hz, 1H), 5.20 (dd, *J* = 10.2, 1.2 Hz, 1H), 4.79 (dt, *J* = 17.1, 0.9 Hz, 1H), 3.86 (d, *J* = 1.2 Hz, 1H), 3.79 (d, *J* = 10.4 Hz, 1H), 2.03-1.57 (m, 8H).

^{13}C NMR (101 MHz, $CDCl_3$) δ 138.5, 133.9, 129.2, 129.1, 128.9, 124.3, 82.7, 77.8, 39.7, 37.9, 23.6, 23.5.

IR (neat) cm^{-1}: 3480, 2952, 1446, 1275, 1138, 1082, 997, 931, 708, 625.

HRMS *m/z* (ESI+) calcd for $[C_{14}H_{19}O_3S]^+$ = 267.1049, found: 267.1038.

tert-Butyldimethyl((1-(1-(phenylsulfonyl)allyl)cyclopentyl)oxy)silane (12l)

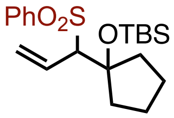

MW (g/mol): 380.62 **Molecular formula: $C_{20}H_{32}O_3SSi$**

1,4-Luditine (4 equiv.) and *tert*-butyldimethylsilyl trifluoromethanesulfonate (4 equiv.) were added at 0 °C to a solution of 1-(1-(phenylsulfonyl)allyl)cyclopentan-1-ol in DCM (0.3 M) at 0 °C, and the reaction was stirred overnight. The reaction mixture was then quenched with water and transferred to a separatory funnel. The organic phase was collected and the aqueous layer was extracted with diethyl ether. The combined organic layers were washed with a saturated aqueous solution of brine, dried over anhydrous $MgSO_4$ and concentrated under reduced pressure. Purification by flash chromatography over silica gel (PE/Et_2O = 95:5) afforded the title compound as a white solid in 87% yield.

Boiling point: 71-72 ºC

^{1}H NMR (400 MHz, $CDCl_3$) δ 7.81 (d, *J* = 7.1 Hz, 2H), 7.57 (t, *J* = 7.4 Hz, 1H), 7.48 (t, *J* = 7.5 Hz, 2H), 6.03 (dt, *J* = 17.2, 10.2 Hz, 1H), 5.17 (dd, *J* = 10.2, 1.3 Hz, 1H), 4.62 (ddd, *J* = 17.2, 1.4, 0.6 Hz, 1H), 3.47 (d, *J* = 10.2 Hz, 1H), 2.48 (m, 1H), 2.09 (m, 1H), 1.94-1.63 (m, 6H), 0.91 (s, 9H), 0.19 (s, 3H), 0.13 (s, 3H).

^{13}C NMR (101 MHz, $CDCl_3$) δ 140.3, 133.2, 130.7, 129.1, 128.6, 123.2, 85.8, 78.4, 40.7, 38.8, 26.1, 24.2, 23.0, 18.6, –2.2.

IR (neat) cm^{-1}: 2952, 2855, 1446, 1304, 1282, 1247, 1200, 1142, 1081, 994, 937, 835, 771, 708, 686, 618.

HRMS *m/z* (ESI+) calcd for $[C_{20}H_{33}O_3SSi]^+$ = 381.1914, found: 381.1914.

4,4,5,5-Tetramethyl-2-[3-(4-methylbenzyl)-4-(_p_-tolyl)but-2-en-1-yl]-1,3,2-dioxaborolane (13a)

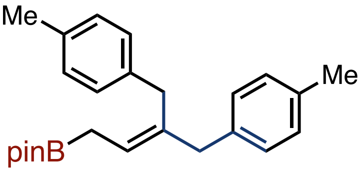

MW (g/mol): 376.35 **Molecular formula: $C_{25}H_{33}BO_2$**

Prepared following general procedure **GP-H**. Purification by flash chromatography over borylated silica gel (PE/Et_2O = 90:10) afforded **13a** as a colourless oil in 92% yield.

^{1}H NMR (400 MHz, $CDCl_3$) δ 7.11-7.06 (m, 8H), 5.60 (t, *J* = 8.0 Hz, 1H), 3.24 (s, 2H), 3.19 (s, 2H), 2.35 (s, 6H), 1.85 (d, *J* = 8.0 Hz, 2H), 1.29 (s, 12H).

^{13}C NMR (101 MHz, $CDCl_3$) δ 137.7, 137.4, 137.2, 135.3, 135.2, 129.0, 129.0, 128.9, 128.8, 122.8, 83.3, 42.7, 34.3, 25.0, 21.2.

IR (neat) cm^{-1}: 2977.1513, 1371, 1343, 1272, 1144, 968, 888, 845, 806.

HRMS *m/z* (ESI-TOF) calcd for $[C_{25}H_{34}BO_2]^+$ = 377.2646, found 377.2643.

2-[3-(3,5-Dimethoxybenzyl)-4-(3,5-dimethoxyphenyl)but-2-en-1-yl)-4,4,5,5-tetramethyl-1,3,2-dioxaborolane (13b)

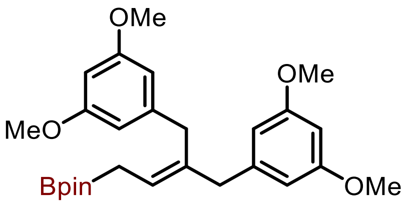

MW (g/mol): 468.40 **Molecular formula: $C_{27}H_{37}BO_6$**

Prepared following general procedure **GP-H**. Purification by flash chromatography over borylated silica gel (PE/Et_2O = 90:10) afforded **13b** as a colourless oil in 67% yield.

^{1}H NMR (400 MHz, $CDCl_3$) δ 6.34-6.32 (m, 4H), 6.31-6.28 (m, 2H), 5.59 (t, *J* = 7.9 Hz, 1H), 3.76 (s, 12H), 3.14 (s, 2H), 3.09 (s, 2H), 1.81 (d, *J* = 7.9 Hz, 2H), 1.25 (s, 12H).

^{13}C NMR (101 MHz, $CDCl_3$) δ 160.8, 160.7, 143.3, 142.7, 136.4, 123.8, 107.6, 107.0, 98.1, 97.9, 83.4, 55.4, 43.6, 35.2, 29.9, 24.9.

IR (neat) cm^{-1}: 2936, 2836, 1595, 1462, 1429, 1343, 1323, 1205, 1154, 1067, 831.

HRMS *m/z* (ESI-TOF) calcd for $[C_{27}H_{38}BO_6]^+$ = 469.2756, found: 469.2763.

2-(3-Allylhexa-2,5-dien-1-yl)-4,4,5,5-tetramethyl-1,3,2-dioxaborolane (13c)

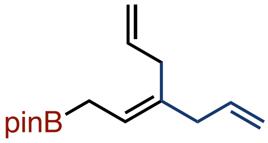

MW (g/mol): 248.17 **Molecular formula: $C_{15}H_{25}BO_2$**

Prepared following general procedure **GP-H**. Purification by flash chromatography over borylated silica gel (PE/Et_2O = 90:10) afforded **13c** as a colourless oil in 79% yield.

^{1}H NMR (400 MHz, $CDCl_3$) δ 5.82-5.67 (m, 2H), 5.38 (t, *J* = 7.9 Hz, 1H), 5.04-4.97 (m, 4H), 2.76 (d, *J* = 6.4 Hz, 2H), 2.73 (dq, *J* = 6.8, 1.2 Hz, 2H), 1.64 (d, *J* = 7.9 Hz, 2H), 1.23 (s, 12H).

^{13}C NMR (101 MHz, $CDCl_3$) δ 137.5, 136.1, 135.1, 121.6, 115.7, 115.2, 83.3, 41.6, 34.4, 24.9

IR (neat) cm^{-1}: 2977, 1636, 1454, 1371, 1323, 1274, 1142, 951, 910, 849, 750, 674.

HRMS *m/z* (ESI-TOF) calcd for $[C_{15}H_{26}BO_2]^+$ = 249.2020, found: 249.2011.

2-[(*E*)-3-Cinnamyl-6-phenylhexa-2,5-dien-1-yl]-4,4,5,5-tetramethyl-1,3,2-dioxaborolane (13d)

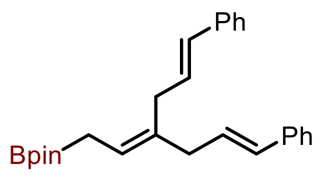

MW (g/mol): 400.37 **Molecular formula: $C_{27}H_{33}BO_2$**

Prepared following general procedure **GP-H**. Purification by flash chromatography over borylated silica gel (PE/Et_2O = 90:10) afforded **13d** as a colourless oil in 99% yield.

^{1}H NMR (400 MHz, $CDCl_3$) δ 7.35-7.26 (m, 8H), 7.21-7.16 (m, 2H), 6.40 (d, *J* = 15.9 Hz, 2H), 6.19 (ddt, *J* = 16.2, 9.4, 6.8 Hz, 2H), 5.49 (d, *J* = 7.9 Hz, 1H), 2.95 (t, *J* = 7.4 Hz, 4H), 1.73 (d, *J* = 7.9 Hz, 2H), 1.24 (s, 12H).

^{13}C NMR (101 MHz, $CDCl_3$) δ 138.0, 137.9, 135.4, 131.1, 130.6, 129.5, 128.6, 128.5, 128.3, 127.0, 126.9, 126.2, 126.1, 122.1, 83.4, 40.9, 34.1, 24.9.

IR (neat) cm^{-1}: 3024, 2976, 2924, 1648, 1598, 1495, 1448, 1371, 1324, 1274, 1142, 965, 886, 847, 746, 693.

HRMS *m/z* (ESI-TOF) calcd for $[C_{27}H_{34}BO_2]^+$ = 401.2646, found: 401.2648.

4,4,5,5-Tetramethyl-2-[6-methyl-3-(3-methylbut-2-en-1-yl)hepta-2,5-dien-1-yl]-1,3,2-dioxaborolane (13e)

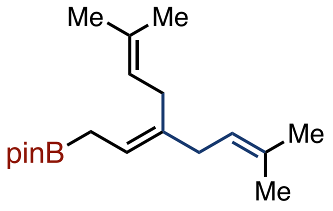

MW (g/mol): 304.28 **Molecular formula: $C_{19}H_{33}BO_2$**

Prepared following general procedure **GP-H**. Purification by flash chromatography over borylated silica gel (PE/Et_2O = 90:10) afforded **13e** as a colourless oil in 87% yield.

^{1}H NMR (400 MHz, $CDCl_3$) δ 5.25 (t, *J* = 7.8 Hz, 1H), 5.11 (tt, *J* = 7.3, 1.4 Hz, 1H), 5.03 (tt, *J* = 7.1, 1.4 Hz, 1H), 2.68 (d, *J* = 7.1 Hz, 2H), 2.63 (d, *J* = 7.3 Hz, 2H), 1.69 (s, 3H), 1.68 (s, 3H), 1.64-1.62 (m, 5H), 1.59 (s, 3H), 1.24 (s, 12H).

^{13}C NMR (101 MHz, $CDCl_3$) δ 138.4, 132.3, 131.6, 123.2, 122.9, 118.8, 83.2, 36.0, 29.2, 26.0, 24.9, 17.9, 17.8.

IR (neat) cm^{-1}: 2974, 2923, 2855, 1371, 1340, 1320, 1144, 1105, 968, 886, 846.

HRMS *m/z* (ESI-TOF) calcd for $[C_{19}H_{34}BO_2]^+$ = 305.2646, found: 305.2651.

(*E*)-4,4,5,5-Tetramethyl-2-[4-(*p*-tolyl)but-2-en-1-yl]-1,3,2-dioxaborolane (13f)

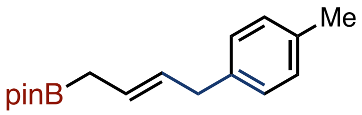

MW (g/mol): 272.20 **Molecular formula: $C_{17}H_{25}BO_2$**

Prepared following general procedure **GP-I**. Purification by flash chromatography over borylated silica gel (PE/Et_2O = 90:10) afforded **13f** as a colourless oil in 70% yield (74% yield overall, *E*/*Z* = 95:5).

^{1}H NMR (400 MHz, C_6D_6) δ 7.09 (d, *J* = 7.8 Hz, 2H), 7.00 (d, *J* = 7.8 Hz, 2H), 5.77 (dt, *J* = 14.6, 7.2 Hz, 1H), 5.65 (dt, *J* = 14.3, 7.5 Hz, 1H), 3.27 (d, *J* = 6.8 Hz, 2H), 2.13 (s, 3H), 1.87 (d, *J* = 7.2 Hz, 2H), 1.04 (s, 12H).

^{13}C NMR (101 MHz, C_6D_6) δ 138.5, 135.2, 129.9, 129.3, 128.9, 127.1, 83.1, 39.3, 24.9, 21.1.

IR (neat) cm^{-1}: 2977, 2928, 1447, 1326, 1141, 982, 849, 804, 725, 673, 583.

HRMS *m/z* (ESI-TOF) calcd for $[C_{17}H_{26}BO_2]^+$ = 273.2020, found: 273.2027.

(*E*)-2-[4-(3,5-Dimethoxyphenyl)but-2-en-1-yl]-4,4,5,5-tetramethyl-1,3,2-dioxaborolane (13g)

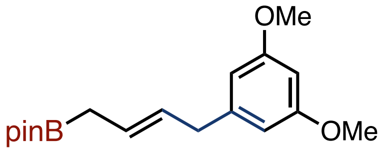

MW (g/mol): 318.22 **Molecular formula: $C_{18}H_{27}BO_4$**

Prepared following general procedure **GP-I**. Purification by flash chromatography over borylated silica gel (PE/Et_2O = 90:10) afforded **13g** as a colourless oil in 81% yield (90% yield overall, *E*/*Z* = 90:10).

^{1}H NMR (400 MHz, $CDCl_3$) δ 6.36 (d, *J* = 2.3 Hz, 2H), 6.29 (t, *J* = 2.3 Hz, 1H), 5.63-5.56 (dt, *J* = 15.0, 6.7 Hz, 1H), 5.49-5.56 (dt, *J* = 15.0, 6.1 Hz, 1H), 3.77 (s, 6H), 3.27 (d, *J* = 6.2 Hz, 2H), 1.70 (d, *J* = 6.6 Hz, 2H), 1.25 (s, 12H).

^{13}C NMR (101 MHz, $CDCl_3$) δ 160.8, 143.8, 128.9, 127.1, 106.5, 98.0, 83.3, 55.3, 39.5, 24.8.

IR (neat) cm^{-1}: 2978, 1596, 1461, 1371, 1328, 1205, 1146, 1066, 951, 850, 692.

HRMS *m/z* (ESI-TOF) calcd for $[C_{18}H_{28}BO_4]^+$ = 319.2075, found: 319.2082.

(*E*)-2-[4-(2-Bromophenyl)but-2-en-1-yl]-4,4,5,5-tetramethyl-1,3,2-dioxaborolane (13h)

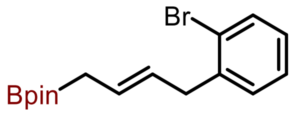

MW (g/mol): 337.06 **Molecular formula: $C_{16}H_{22}BrBO_2$**

Prepared following general procedure **GP-I**. Purification by flash chromatography over borylated silica gel (PE/Et_2O = 90:10) afforded **13h** as a colourless oil in 46% yield (66% yield overall, *E*/*Z* = 70:30).

^{1}H NMR (400 MHz, C_6D_6) δ 7.38 (dd, *J* = 8.0, 1.3 Hz, 1H), 7.05 (dd, *J* = 7.6, 1.8 Hz, 1H), 6.90 (td, *J* = 7.5, 1.3 Hz, 1H), 6.66 (td, *J* = 7.7, 1.8 Hz, 1H), 5.74 (dt, *J* = 14.9, 7.3 Hz, 1H), 5.56 (dt, *J* = 15.2, 6.7 Hz, 1H), 3.40 (d, *J* = 6.6 Hz, 1H), 1.81 (d, *J* = 7.4 Hz, 2H), 1.03 (s, 12H).

^{13}C NMR (101 MHz, C_6D_6) δ 141.0, 132.9, 130.7, 128.6, 127.7, 127.5, 127.4, 125.0, 83.1, 39.7, 24.9.

IR (neat) cm^{-1}: 2980, 2251, 1469, 1323, 1142, 1025, 967, 904, 846, 725, 649.

HRMS *m/z* (ESI-TOF) calcd for $[C_{16}H_{23}BBrO_2]^+$ = 337.0969, found: 337.0965.

(*E*)-2-[4-(2-Iodophenyl)but-2-en-1-yl]-4,4,5,5-tetramethyl-1,3,2-dioxaborolane (13i)

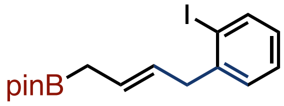

MW (g/mol): 384.06 **Molecular formula: $C_{16}H_{22}BIO_2$**

Prepared following general procedure **GP-I**. Purification by flash chromatography over borylated silica gel (PE/Et_2O = 90:10) afforded **13i** as a colourless oil in 57% yield (63% yield overall, *E*/*Z* = 90:10).

^{1}H NMR (400 MHz, $CDCl_3$) δ 7.80 (dt, *J* = 7.8, 1.2 Hz, 1H), 7.30-7.15 (m, 2H), 6.87 (m, 1H), 5.65-5.48 (m, 2H), 3.44-3.38 (d, *J* = 6.4 Hz, 2H), 1.73-1.66 (d, *J* = 7.5 Hz, 2H), 1.25 (s, 12H).

^{13}C NMR (101 MHz, $CDCl_3$) δ 144.0, 139.4, 129.7, 128.4, 128.1, 127.8, 127.6, 101.0, 83.4, 44.2, 24.9.
IR (neat) cm-1 : 2976, 2925, 1704, 1465, 1324, 1142, 1010, 966, 846, 746, 647.

HRMS *m/z* (ESI+) calcd for $[C_{16}H_{23}BIO_2]^+$ = 385.0830, found: 385.0830.

2-[(2*E*,5*E*)-Hepta-2,5-dien-1-yl]-4,4,5,5-tetramethyl-1,3,2-dioxaborolane (13j)

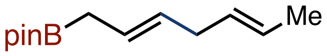

MW (g/mol): 222.14 **Molecular formula: $C_{13}H_{23}BO_2$**

Prepared following general procedure **GP-I**. Purification by flash chromatography over borylated silica gel (PE/Et_2O = 90:10) afforded **13j** as a colourless oil in 59% yield (78% yield overall, *E*/*Z* = 75:25).

^{1}H NMR (400 MHz, C_6D_6) δ 5.74 (dt, *J* = 14.7, 7.3 Hz, 1H), 5.57-5.50 (dd, *J* = 15.2, 6.5 Hz, 2H), 5.50-5.35 (m, 2H), 2.72 (t, *J* = 6.5 Hz, 2H), 1.88 (d, *J* = 7.2 Hz, 2H), 1.55 (d, *J* = 4.6 Hz, 3H), 1.03 (s, 12H).

^{13}C NMR (101 MHz, C_6D_6) δ 130.5, 129.3, 126.4, 125.3, 83.0, 36.3, 24.9, 18.0.

IR (neat) cm^{-1}: 2978, 2931, 1357, 1323, 1143, 965, 883, 846, 674.

HRMS *m/z* (ESI-TOF) calcd for $[C_{13}H_{24}BO_2]^+$ = 223.1864, found: 223.1860.

(*E*)-4,4,5,5-Tetramethyl-2-(6-methylhepta-2,5-dien-1-yl)-1,3,2-dioxaborolane (13k)

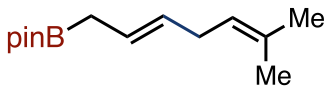

MW (g/mol): 236.16 **Molecular formula: $C_{14}H_{25}BO_2$**

Prepared following general procedure **GP-I**. Purification by flash chromatography over borylated silica gel (PE/Et_2O = 90:10) afforded **13k** as a colourless oil in 86% yield (*E*/*Z*>99:1).

^{1}H NMR (400 MHz, $CDCl_3$) δ 5.49-5.42 (dt, *J* = 15.0, 6.9 Hz, 1H), 5.39-5.32 (dt, *J* = 14.9, 6.4 Hz, 1H), 5.15-5.10 (tt, *J* = 7.3, 1.5 Hz, 1H), 2.67 (t, *J* = 6.8 Hz, 2H), 1.69 (s, 3H), 1.64 (d, *J* = 7.4 Hz, 2H), 1.60 (s, 3H), 1.24 (s, 12H).

^{13}C NMR (101 MHz, $CDCl_3$) δ 132.0, 129.5, 125.0, 123.0, 83.3, 31.7, 25.9, 24.9, 17.8.

IR (neat) cm^{-1}: 2977, 2925, 1357, 1323, 1271, 1214, 1144, 1106, 965, 883, 846, 674.

HRMS *m/z* (ESI-TOF) calcd for $[C_{14}H_{26}BO_2]^+$ = 237.2020, found: 237.2022.

(*E*)-*tert*-Butyldimethyl((1-(3-(4,4,5,5-tetramethyl-1,3,2-dioxaborolan-2-yl)prop-1-en-1-yl)cyclopentyl)oxy)silane (13l)

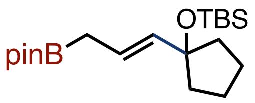

MW (g/mol): 366.42 **Molecular formula: $C_{20}H_{39}B\ O_3Si$**

Prepared following general procedure **GP-I**. Purification by flash chromatography over borylated silica gel (PE/Et_2O = 97:3) afforded **13l** as a colourless oil in 55% yield (*E*/*Z*>99:1).

^{1}H NMR (400 MHz, $CDCl_3$) δ 5.66-5.56 (m, 2H), 1.77-1.55 (m, 10H), 1.24 (s, 12H), 0.84 (s, 9H), 0.00 (s, 6H).

^{13}C NMR (101 MHz, $CDCl_3$) δ 136.2, 123.2, 83.8, 83.3, 39.9, 26.0, 25.8, 25.0, 22.9, 18.3, –2.3, –2.8.

IR (neat) cm^{-1}: 2954, 2856, 1438, 1301, 1252, 1148, 953, 833, 784, 678.

HRMS couldn't be obtained due to fragmentation of the product. However the fragmented product (- TBSOH) could be recorded: **HRMS** *m/z* (ESI+) calcd for $[C_{14}H_{24}BO_2]^+$ = 235,1864, found 235,1870.

3. ¹H and ¹³C NMR spectra copies

Methyl 4-nitro-4-vinylheptanedioate (1a)

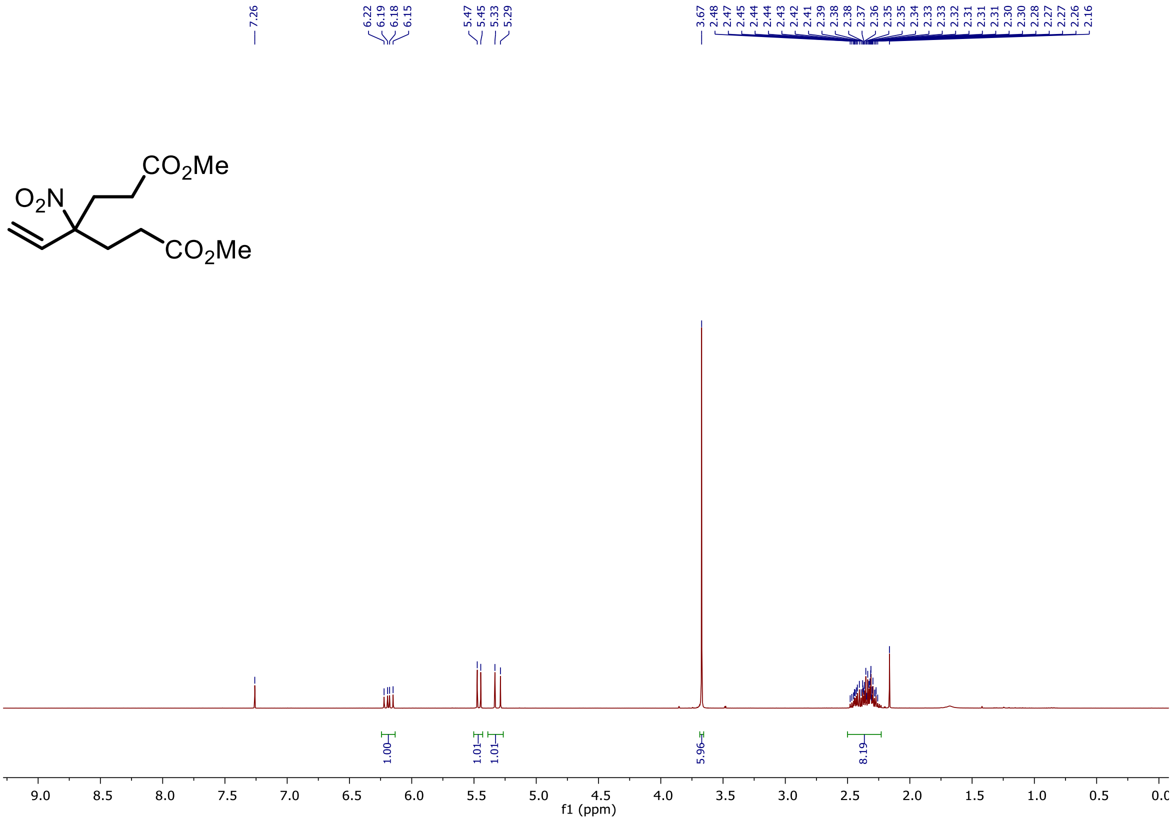

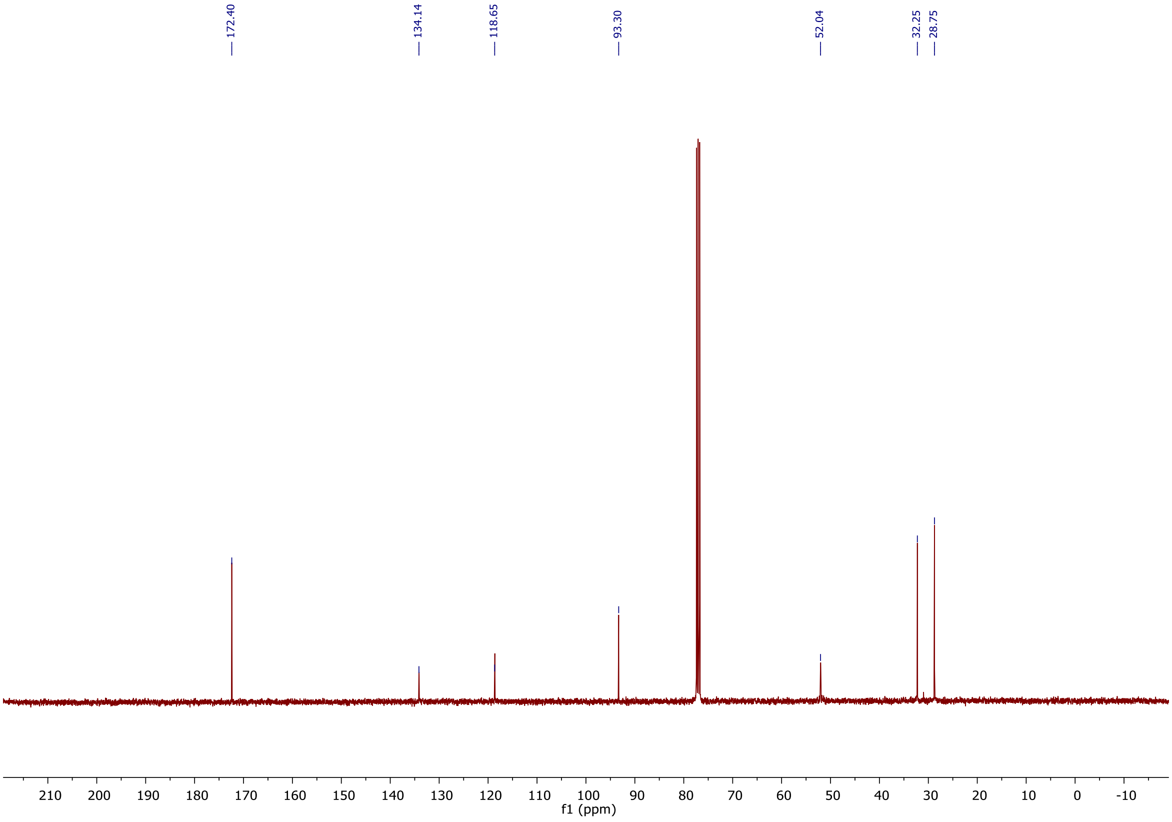

Methyl 4-methyl-4-nitrohex-5-enoate (1b)

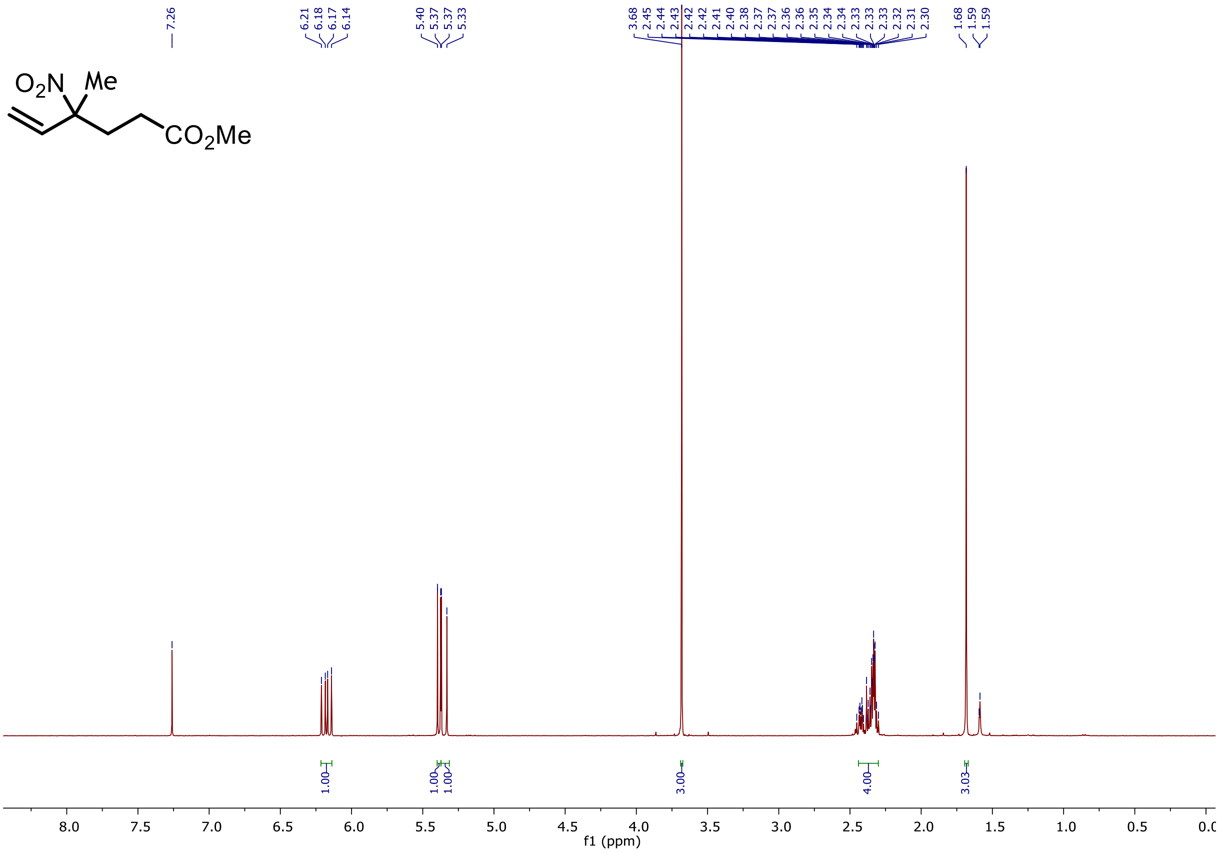

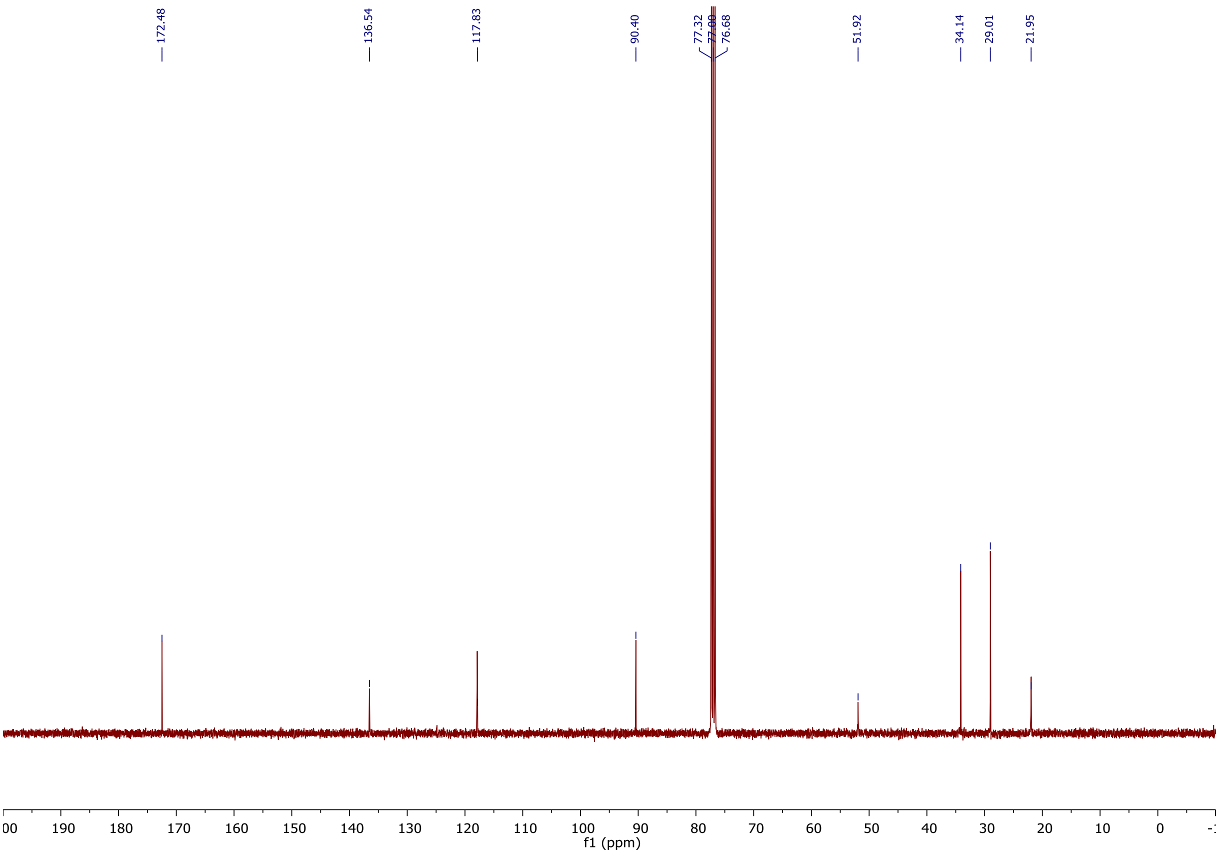

4-Methyl-4-nitrohex-5-enenitrile (1c)

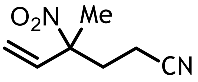

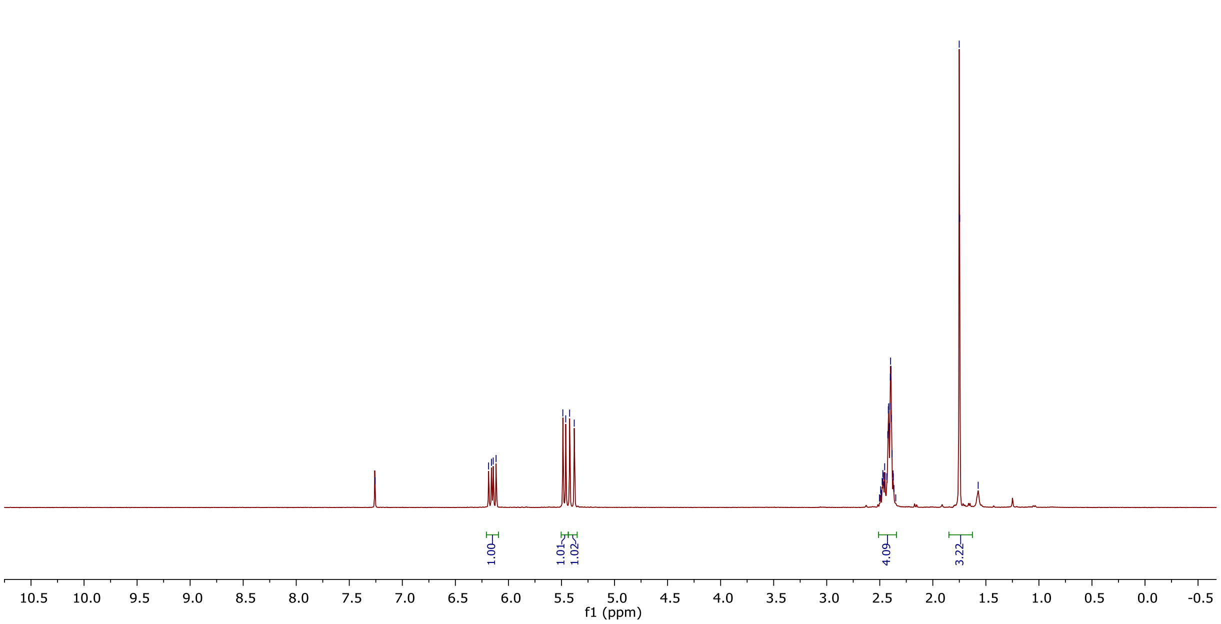

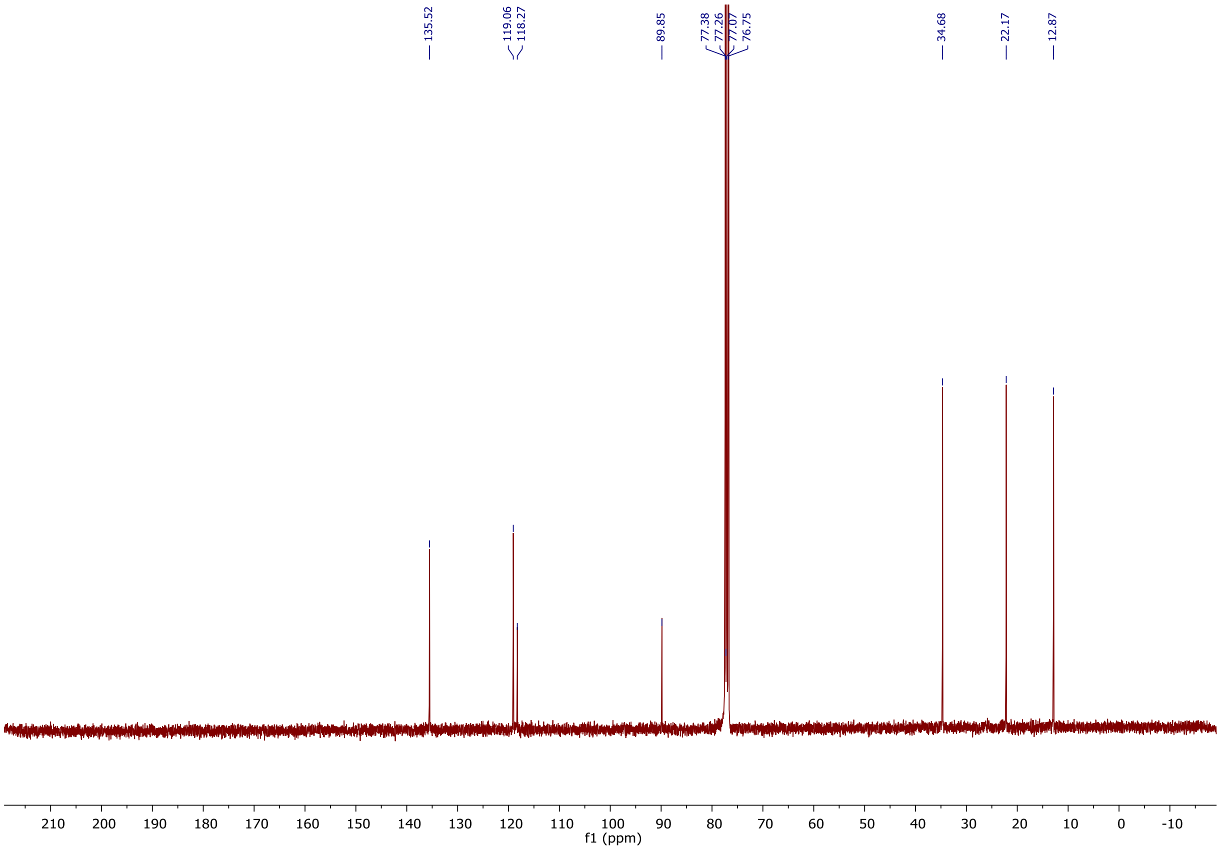

((3-Methyl-3-nitropent-4-en-1-yl)sulfonyl)benzene (1d)

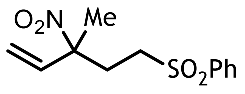

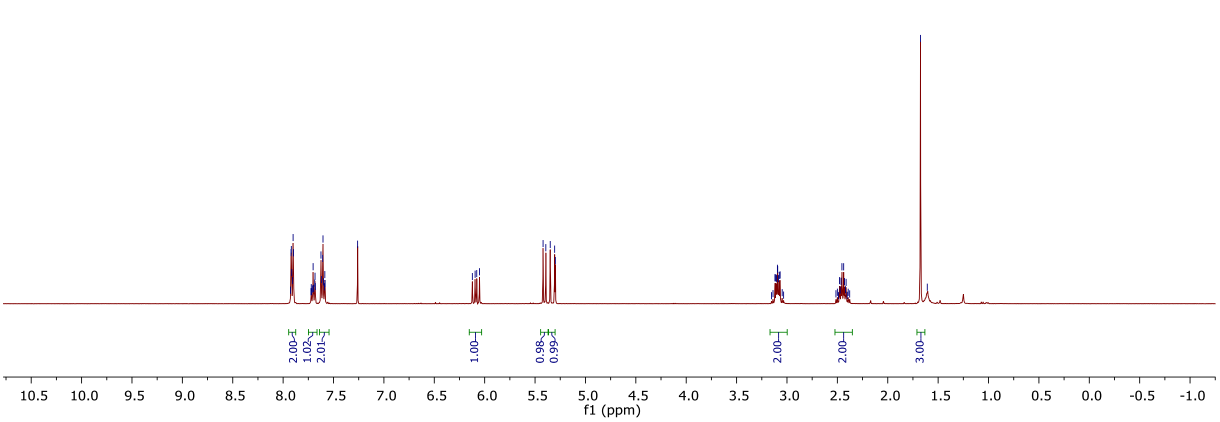

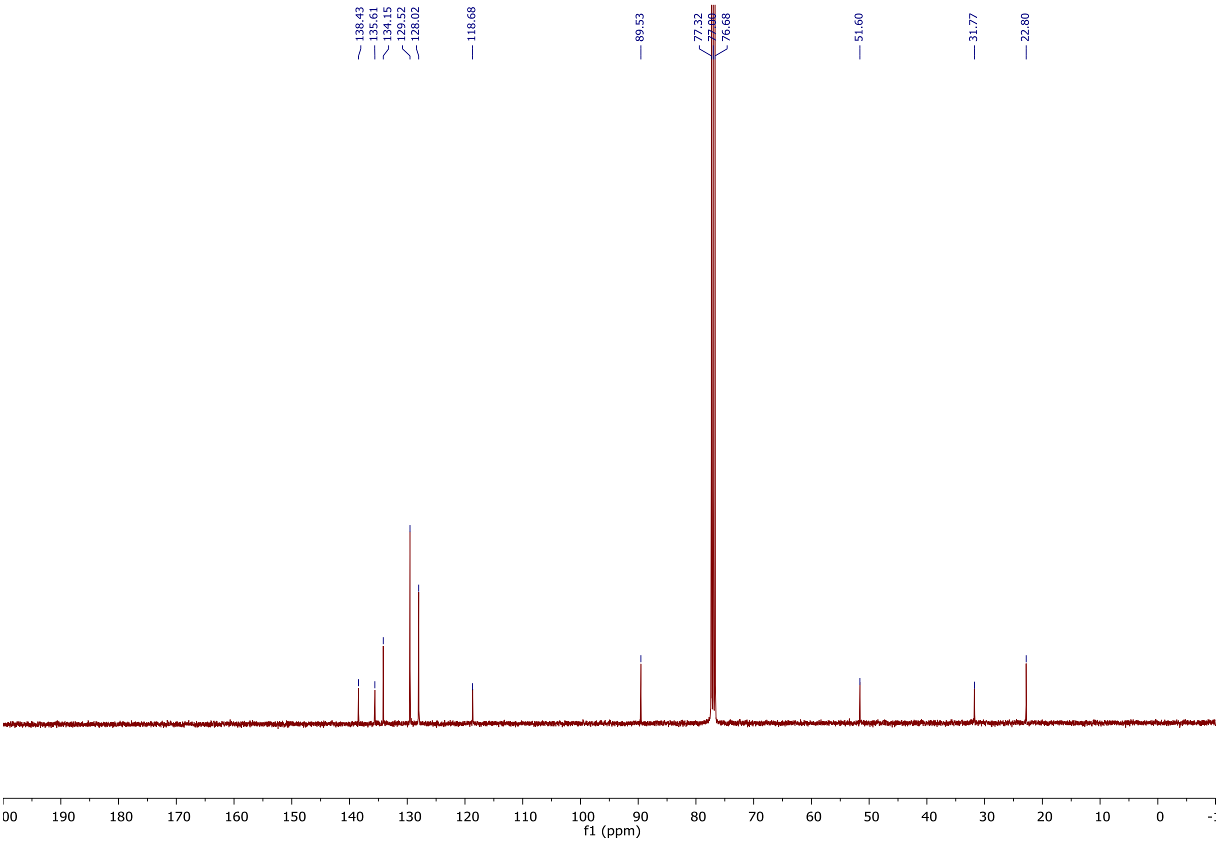

***tert*-Butyldimethyl((2-methyl-2-nitrobut-3-en-1-yl)oxy)silane (1e)**

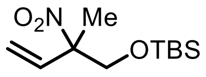

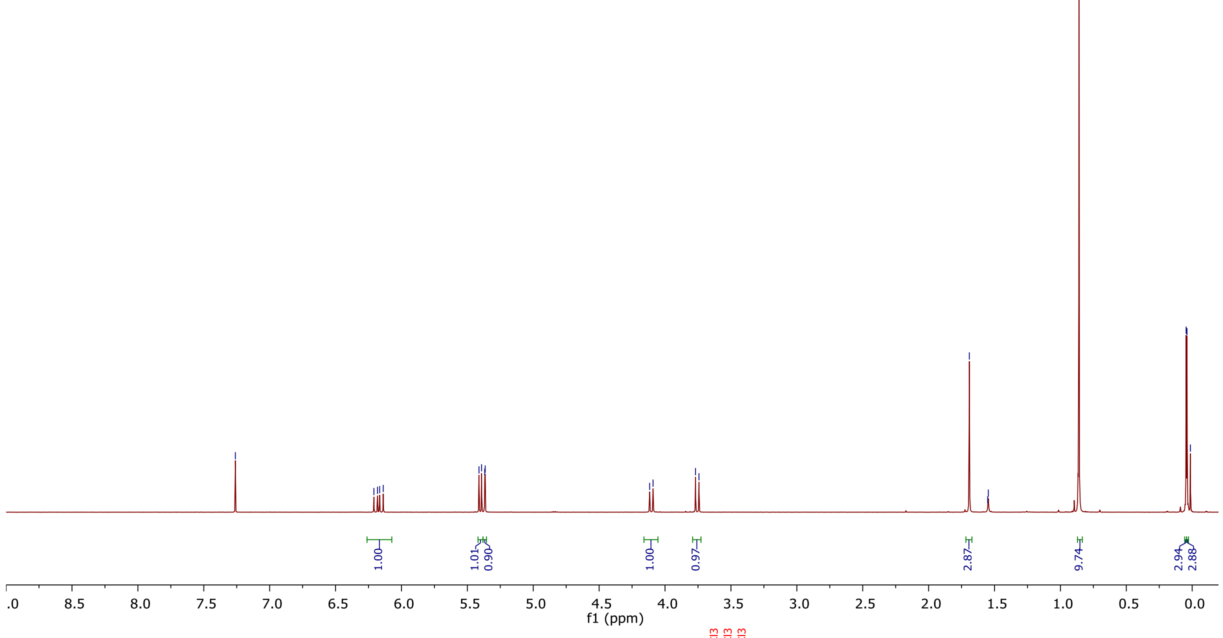

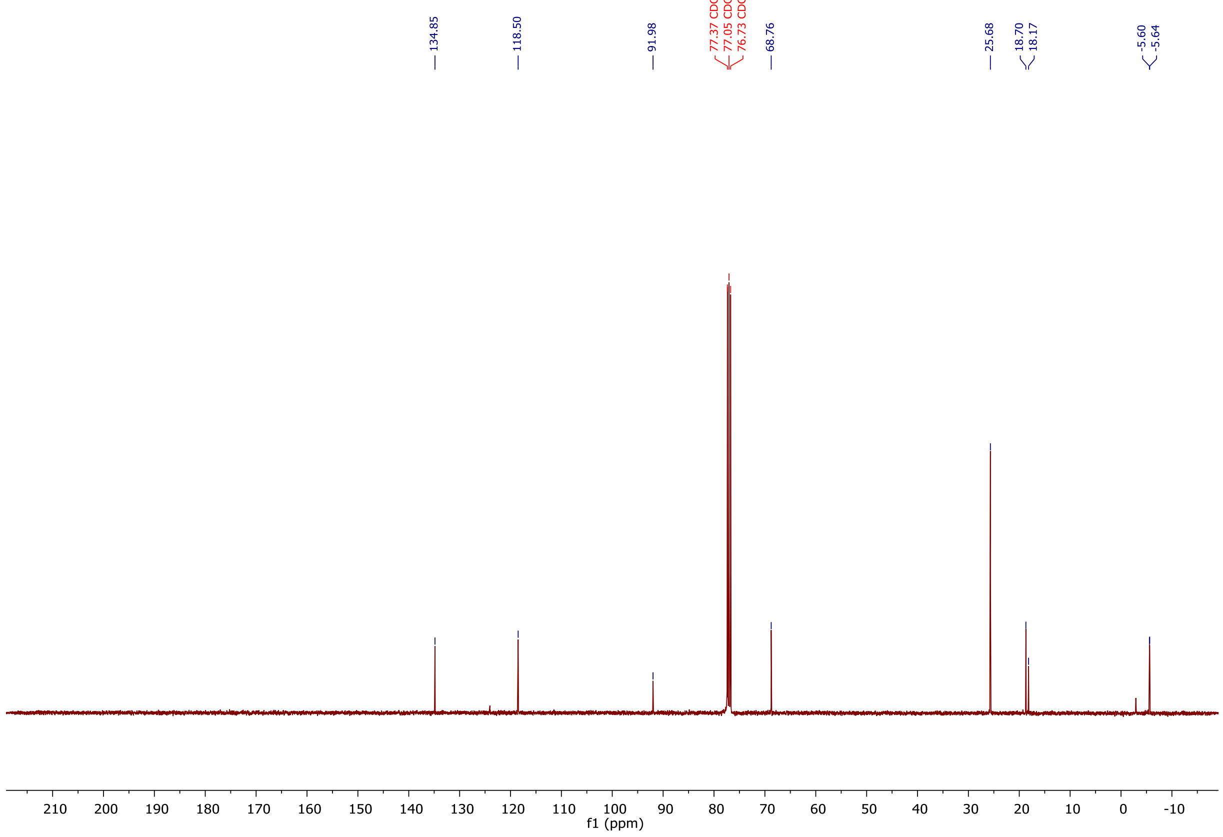

Methyl 4-(4-methoxybenzyl)-4-nitrohex-5-enoate (1f)

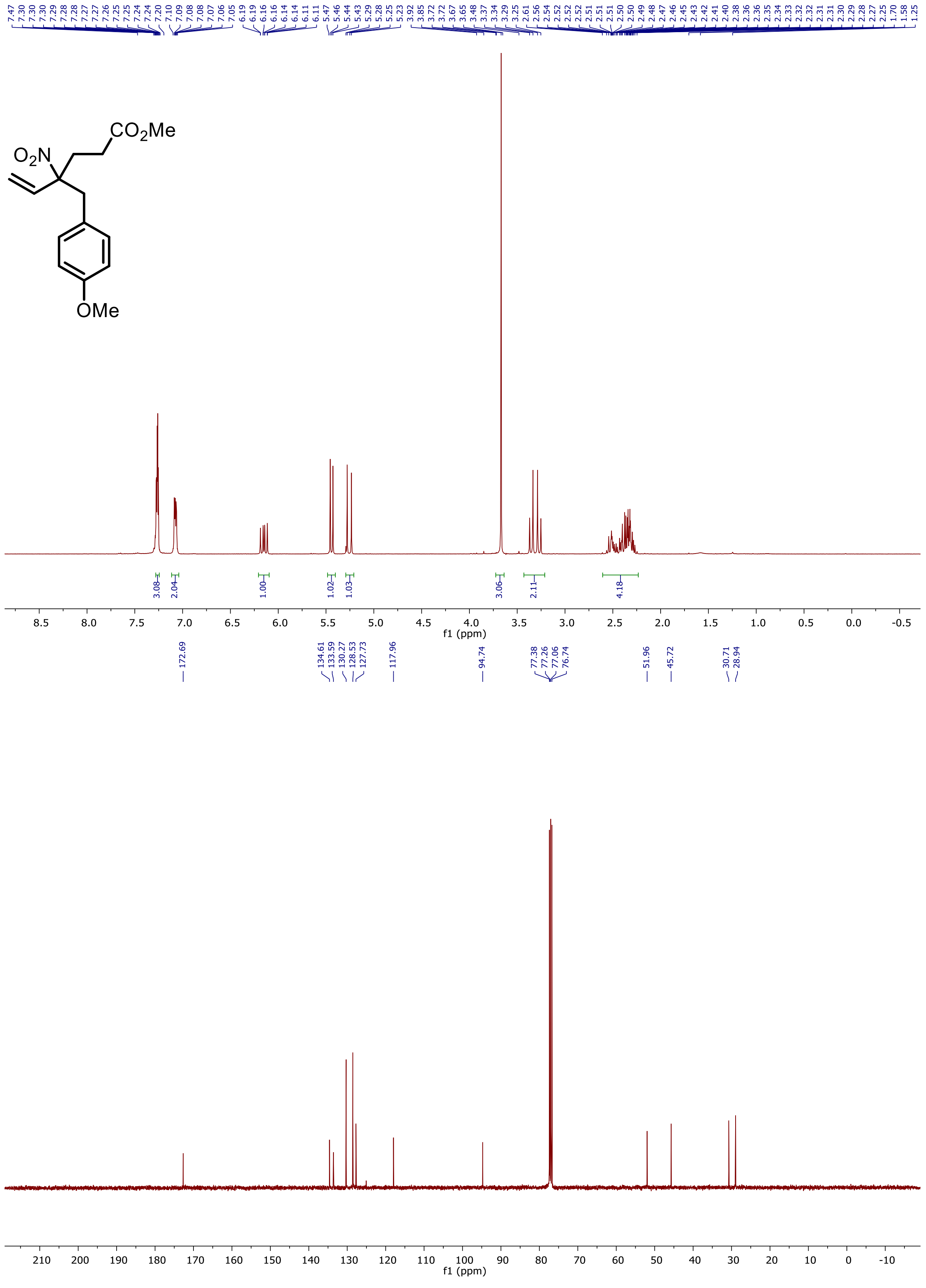

methyl 4-nitro-7-oxo-4-vinyloctanoate

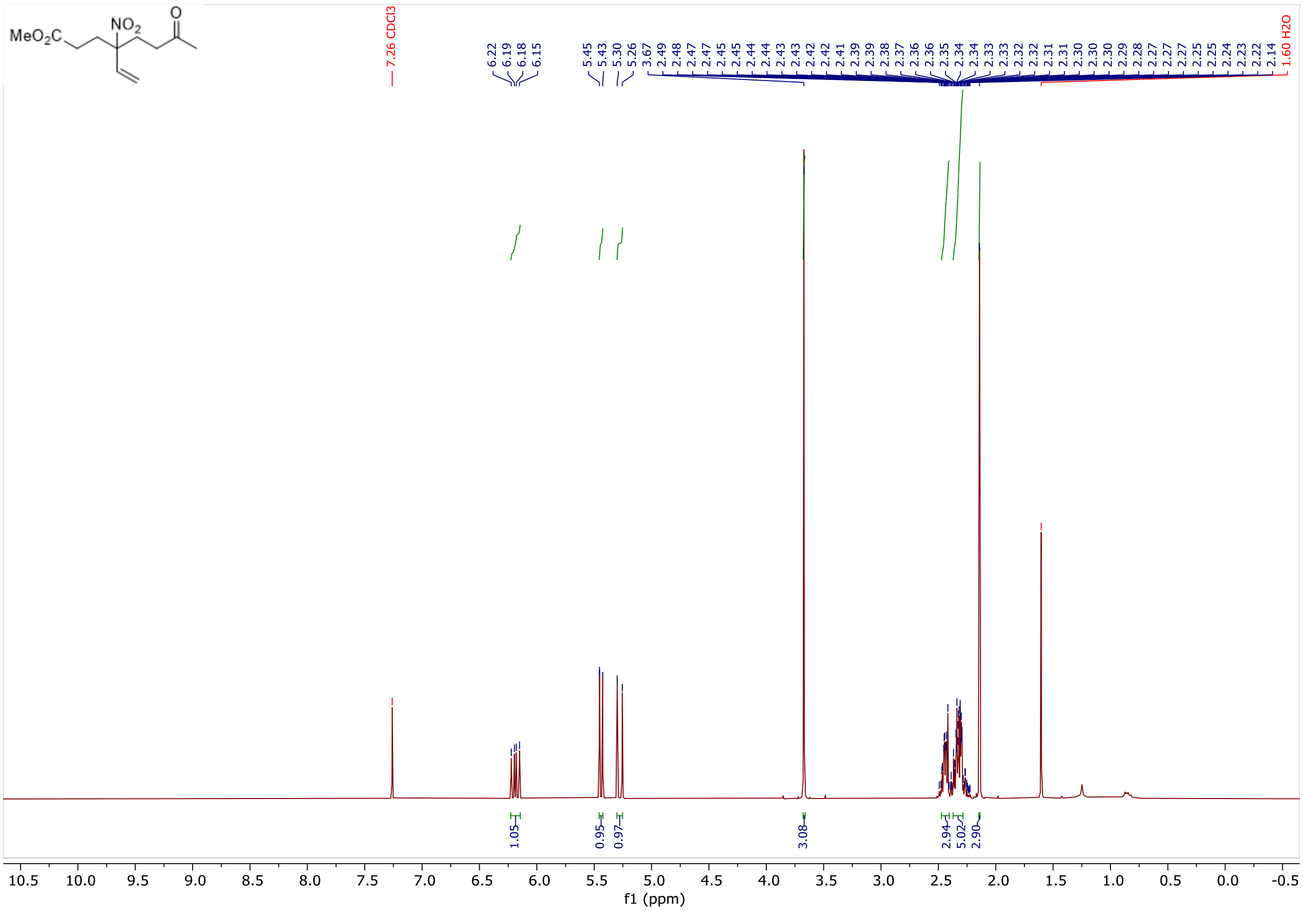

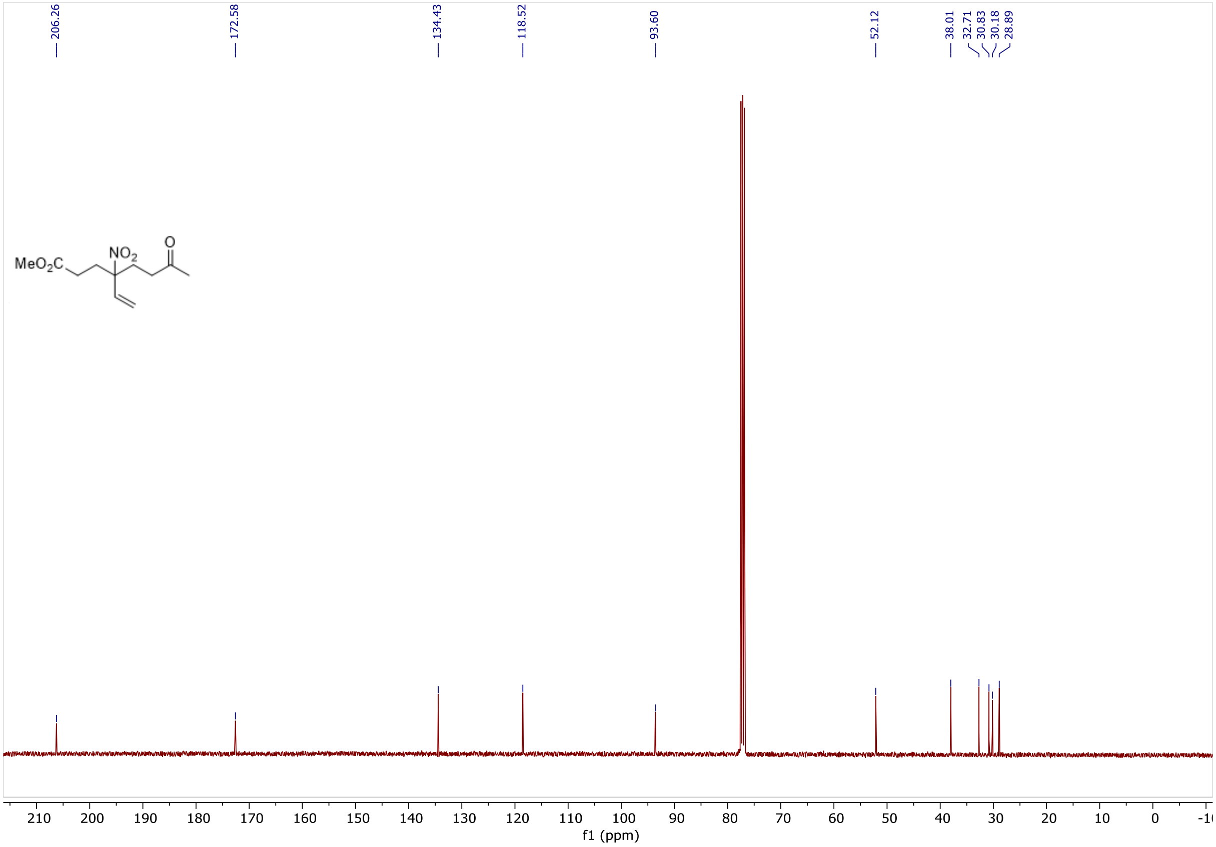

((2-Benzyl-2-nitrobut-3-en-1-yl)oxy)(tert-butyl)dimethylsilane (1h)

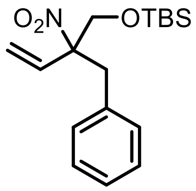

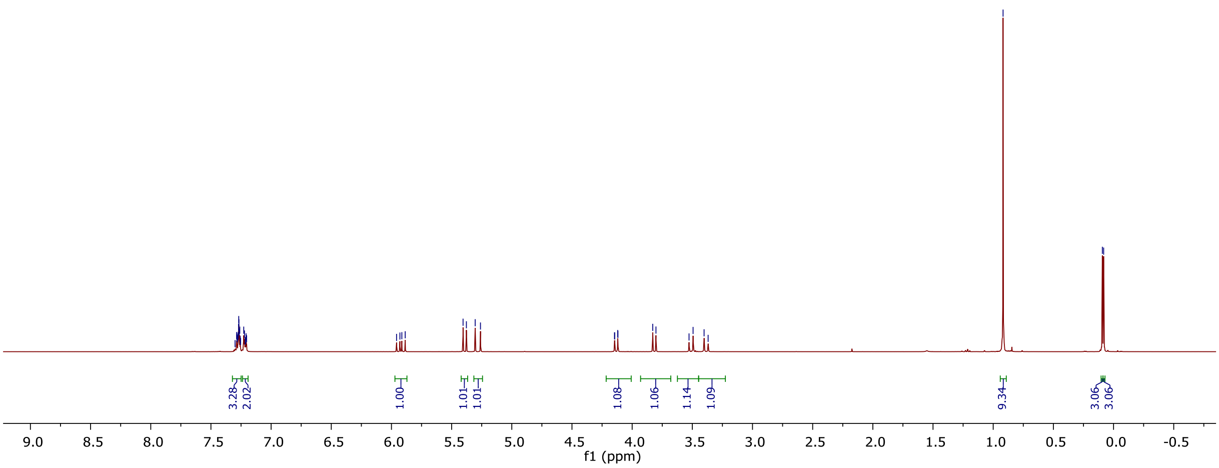

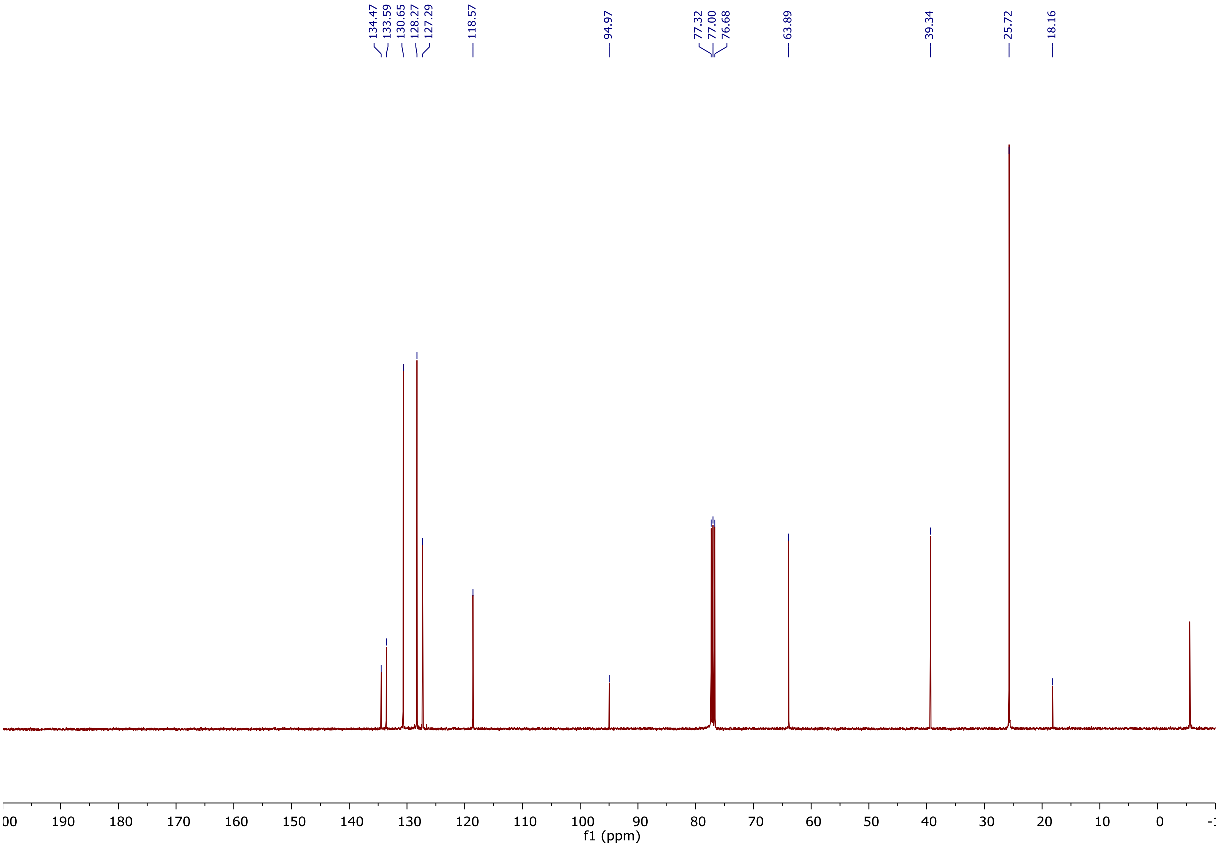

Dimethyl 4-nitro-4-(1-phenylvinyl)heptanedioate (1i)

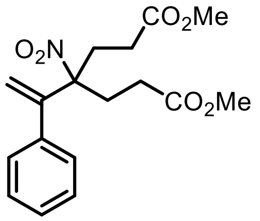

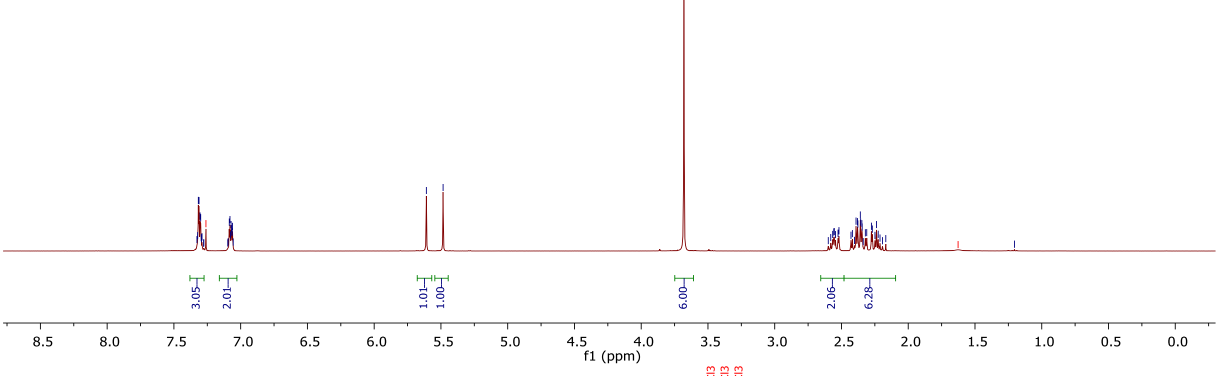

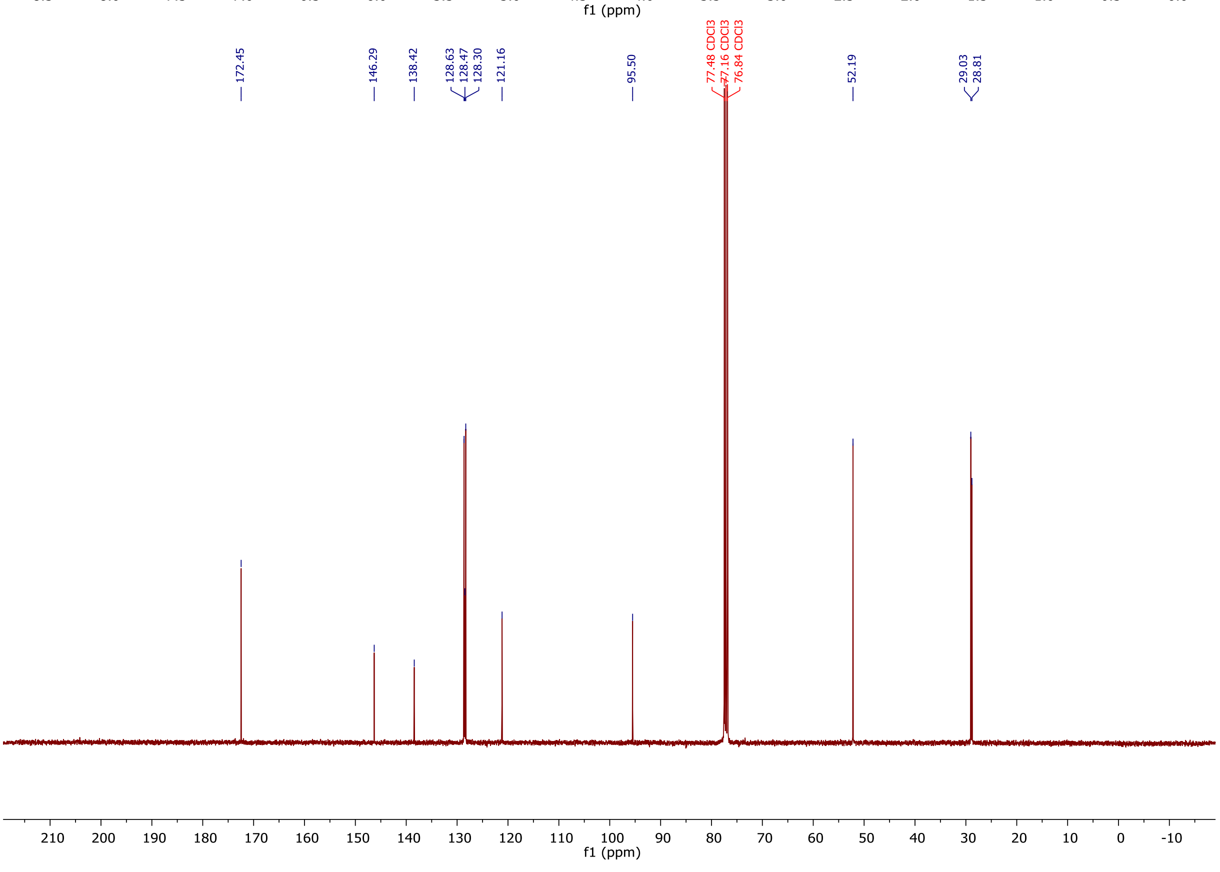

Dimethyl 4-(1-(4-chlorophenyl)vinyl)-4-nitroheptanedioate (1j)

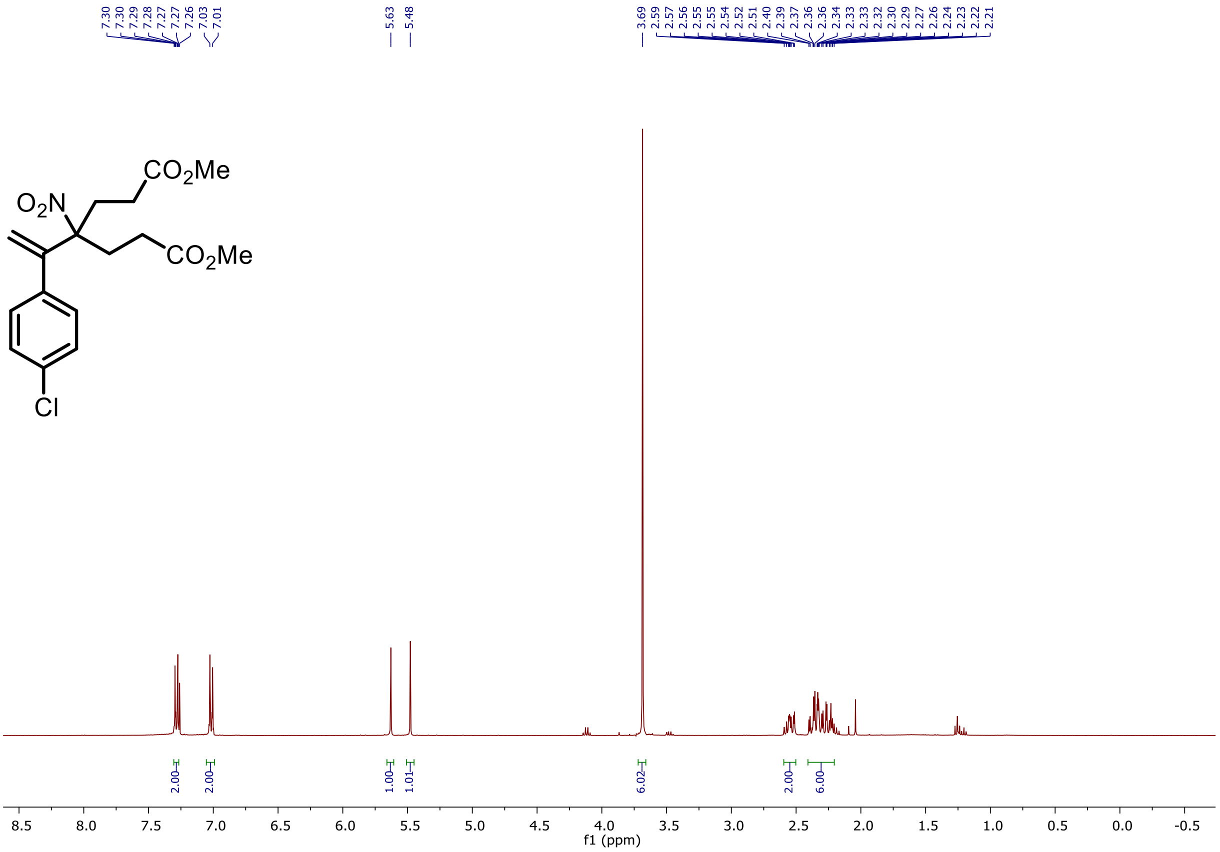

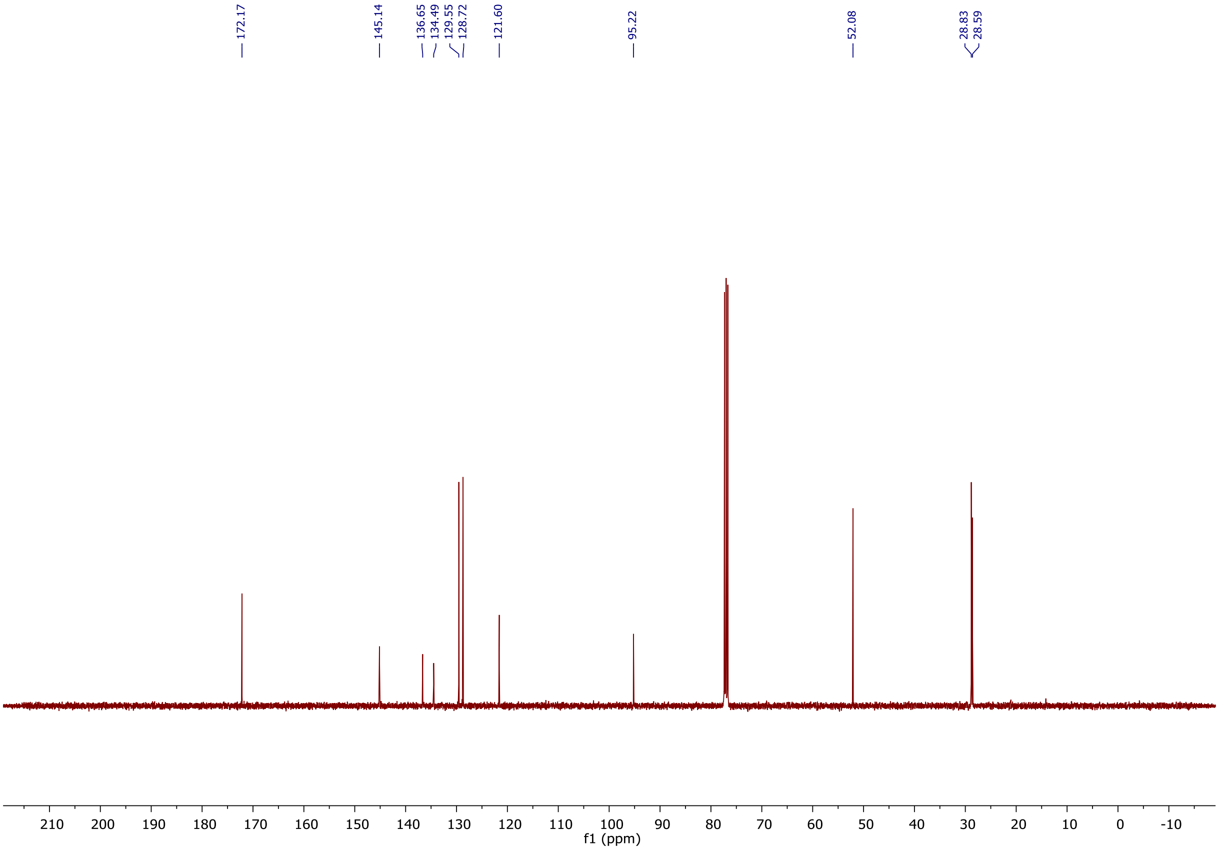

Dimethyl 4-(1-(2-fluorophenyl)vinyl)-4-nitroheptanedioate (1k)

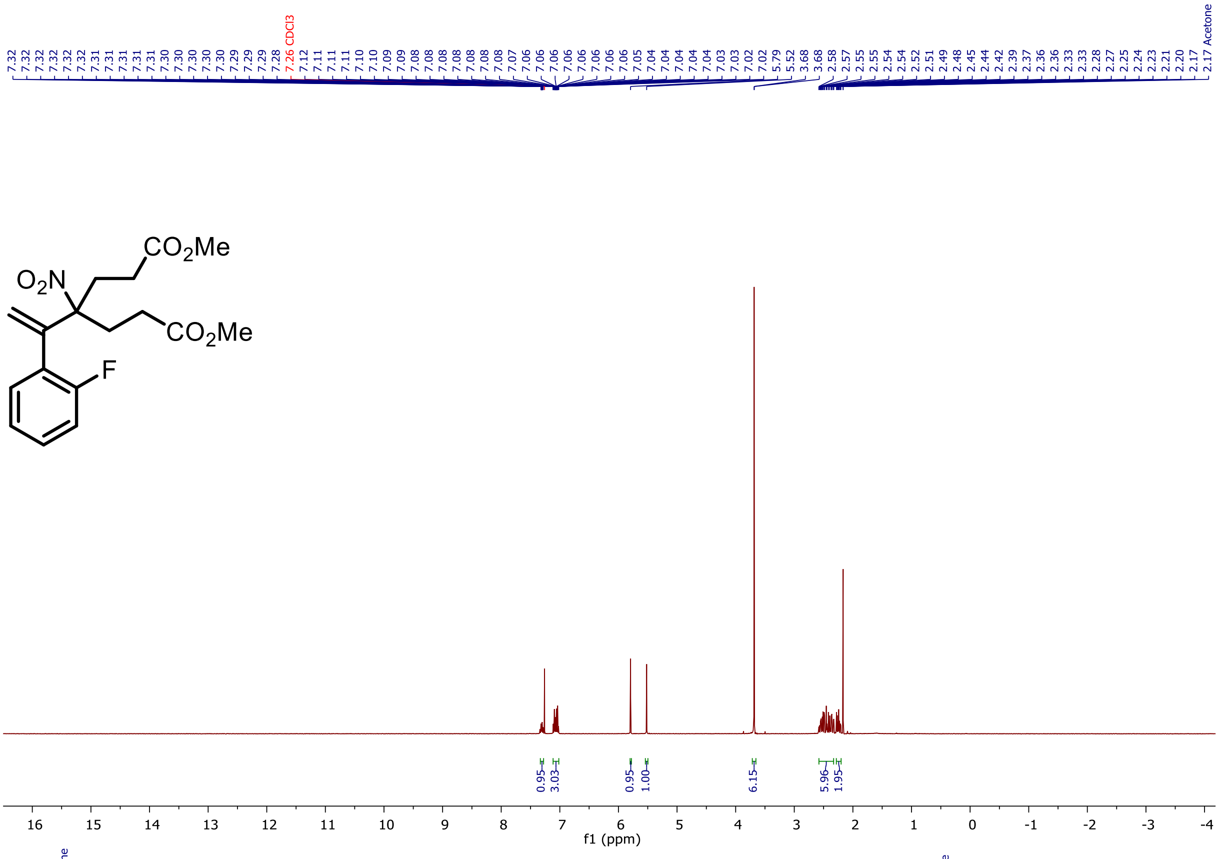

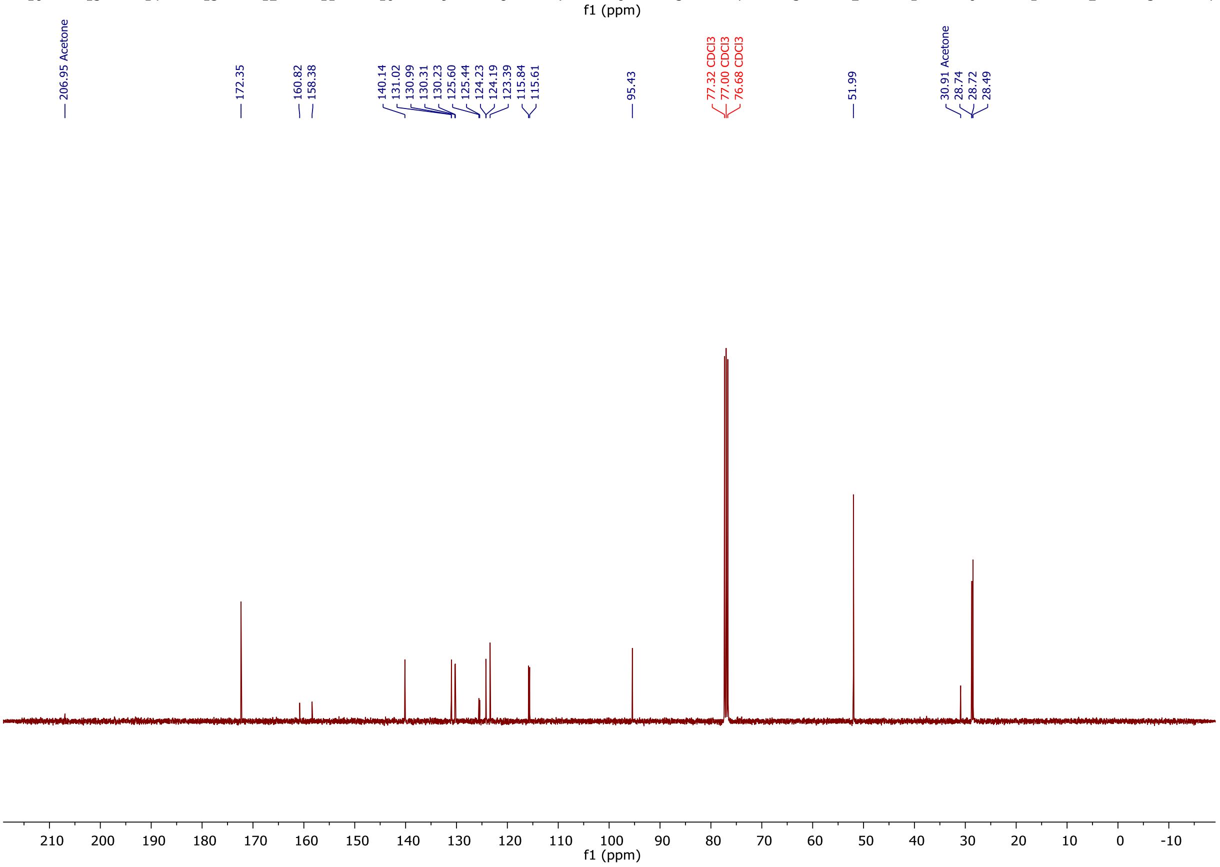

4-Nitro-4-(1-phenylvinyl)heptanedinitrile (1l)

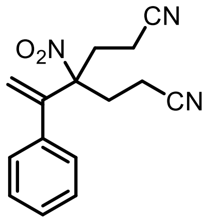

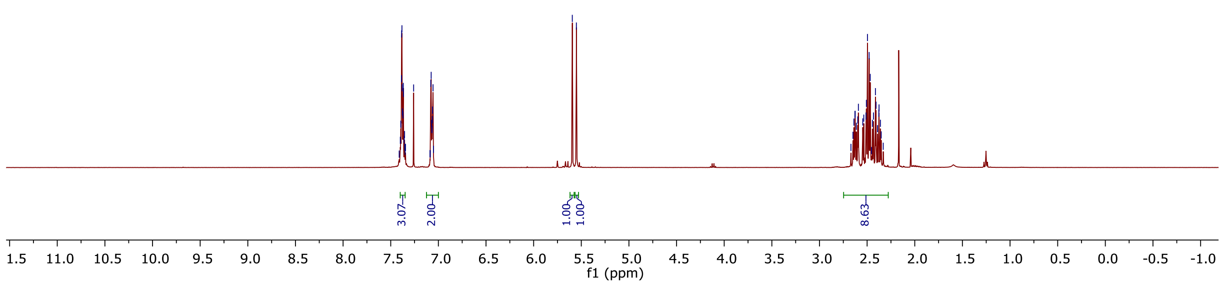

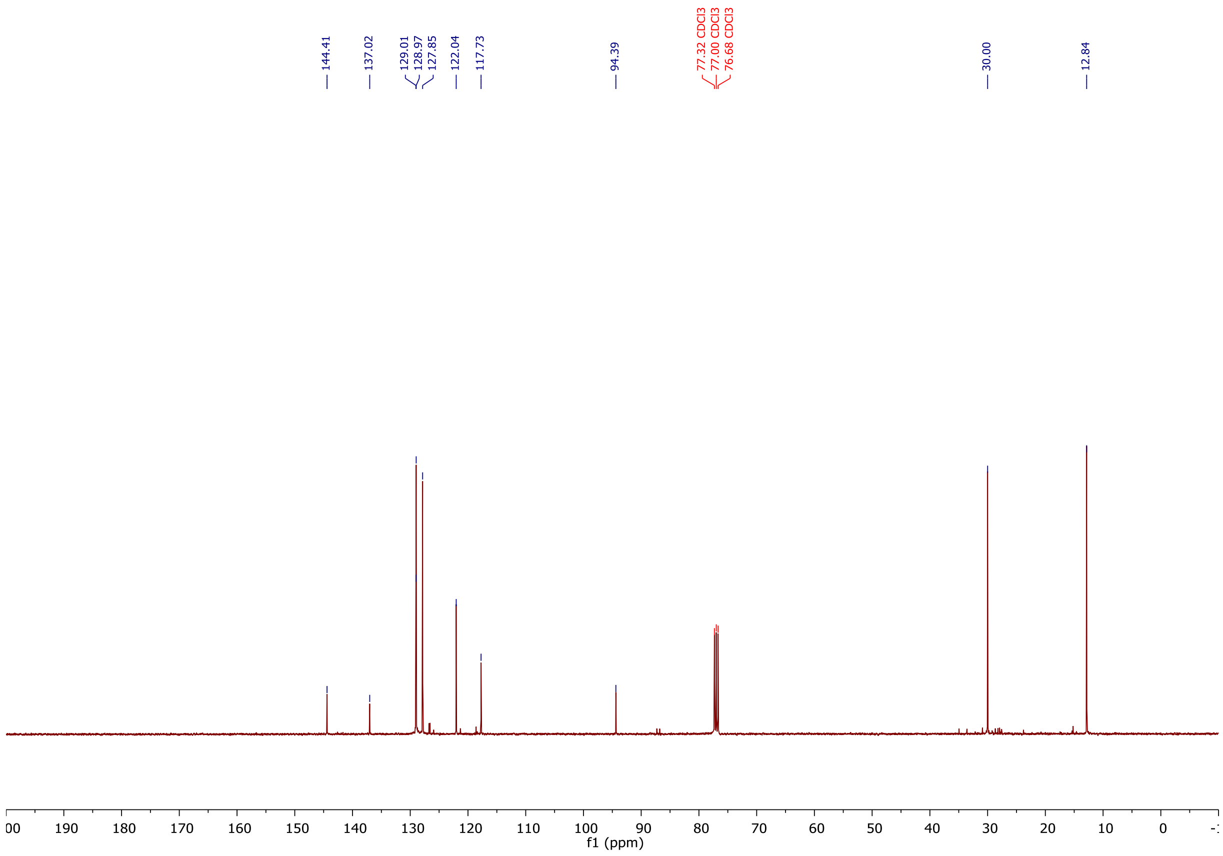

4-Methyl-4-nitro-5-phenylhex-5-enenitrile (1m)

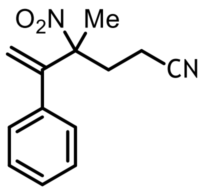

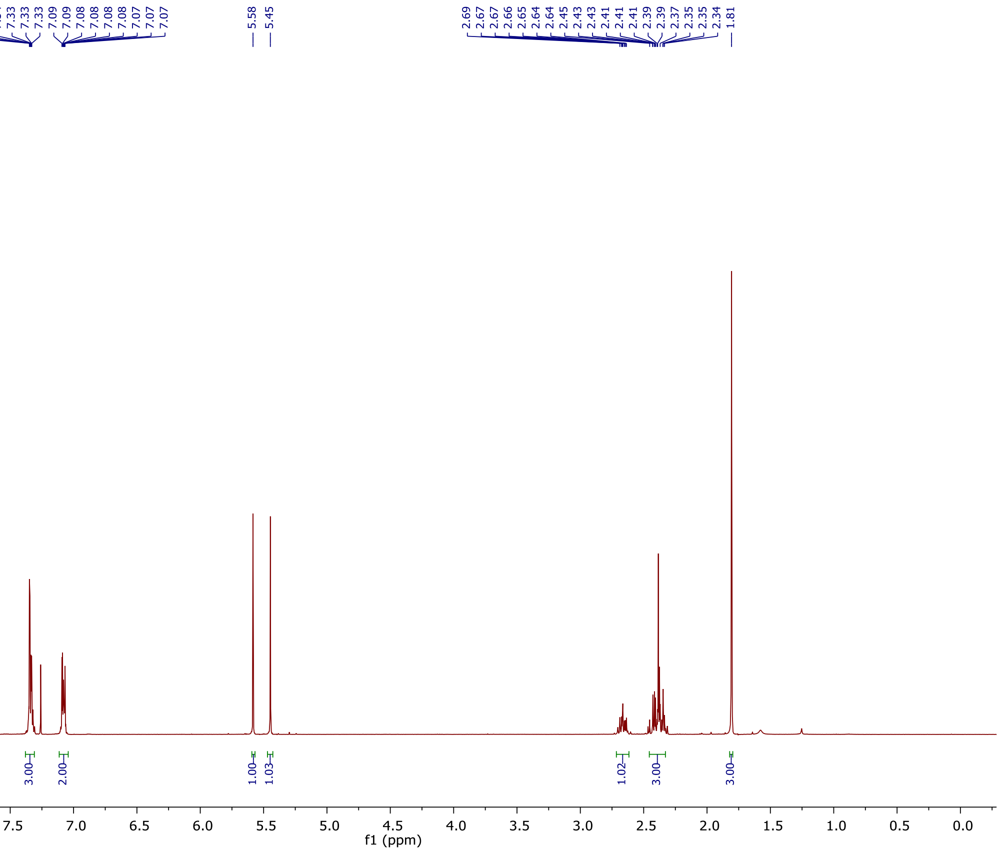

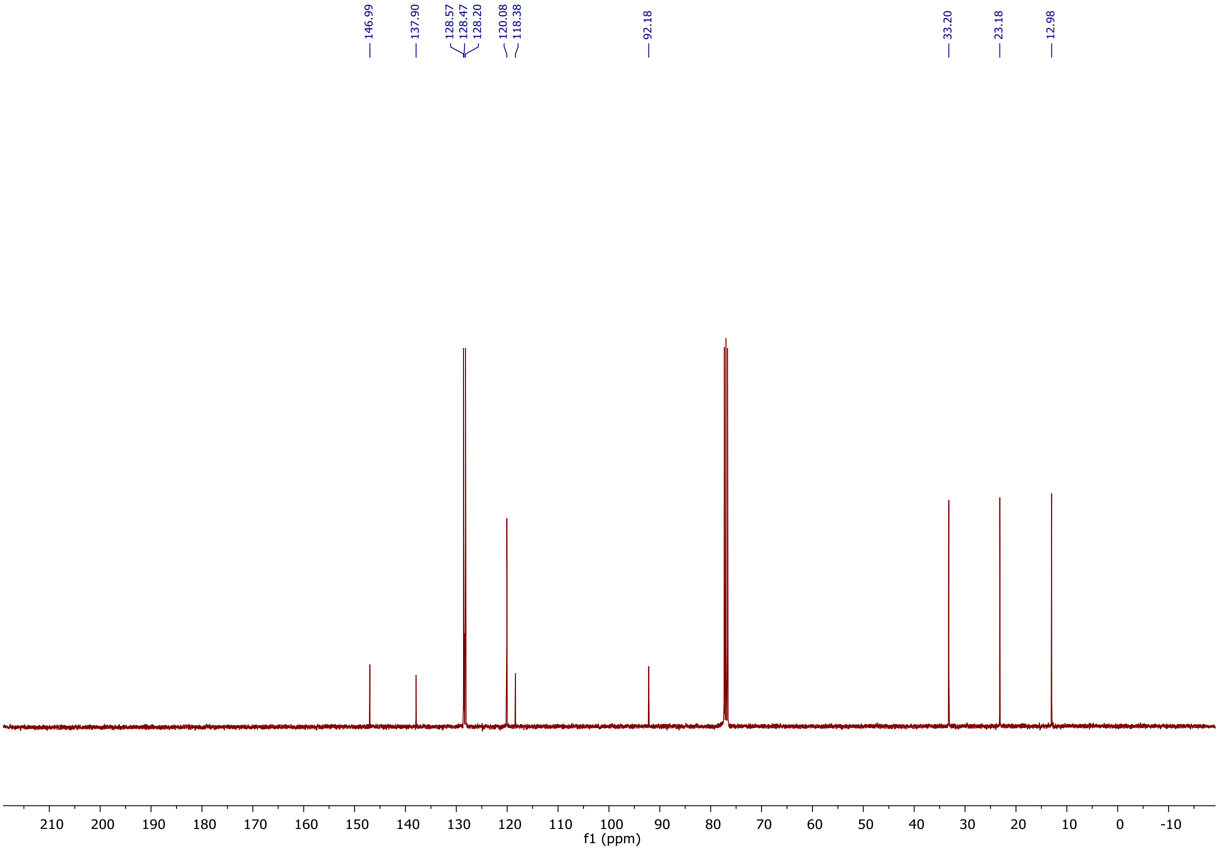

Methyl 4-fluoro-4-nitrohex-5-enoate (1n)

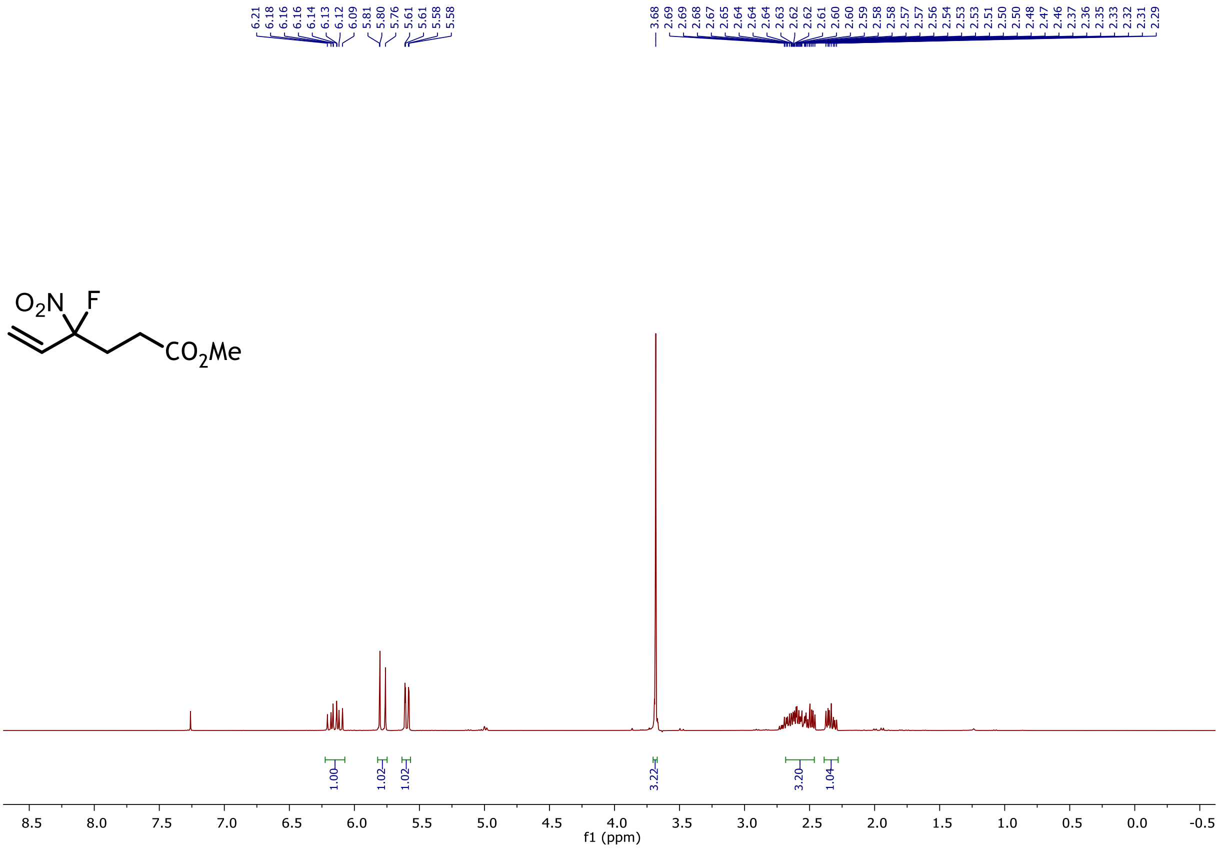

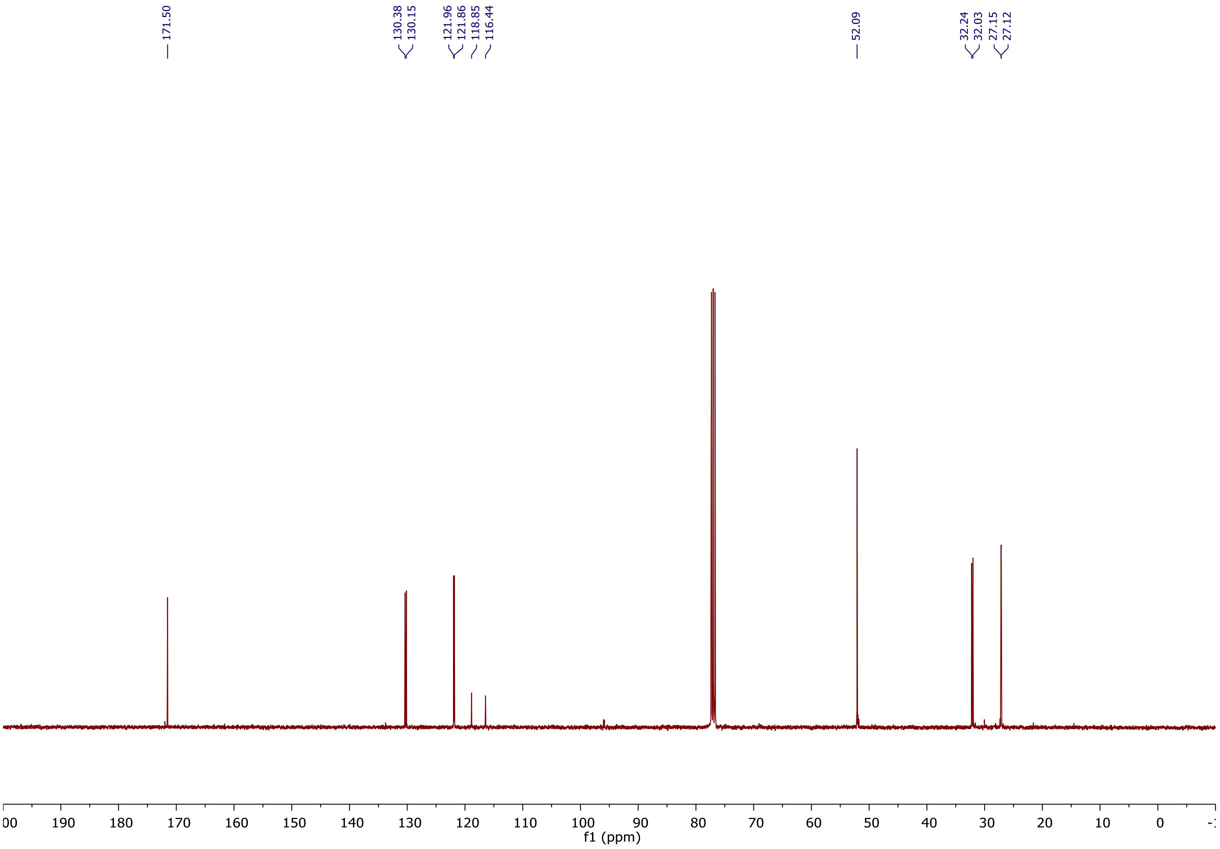

(2-Fluoro-2-nitrobut-3-en-1-yl)benzene (1o)

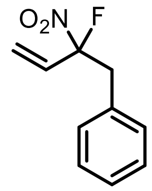

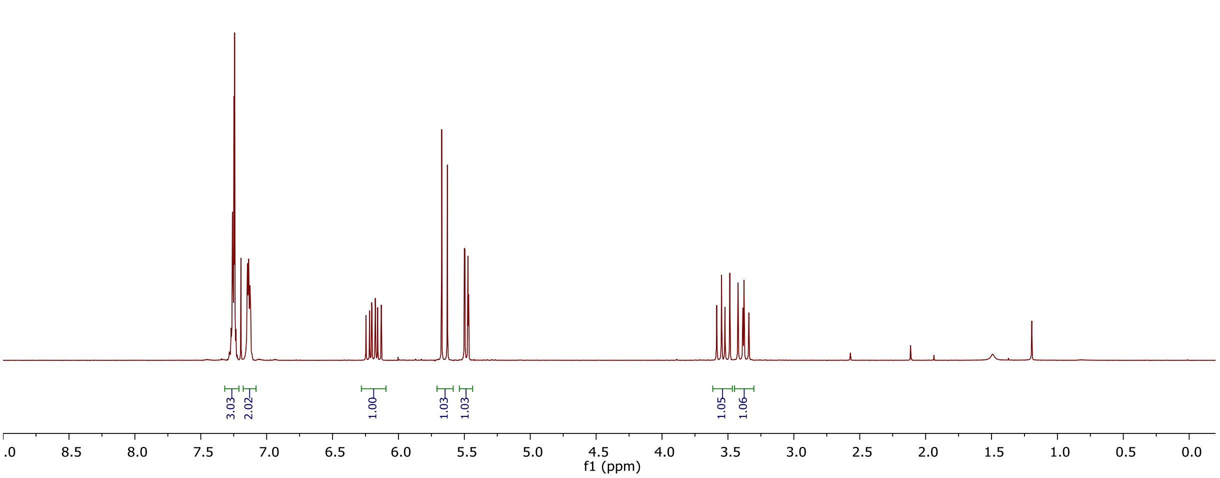

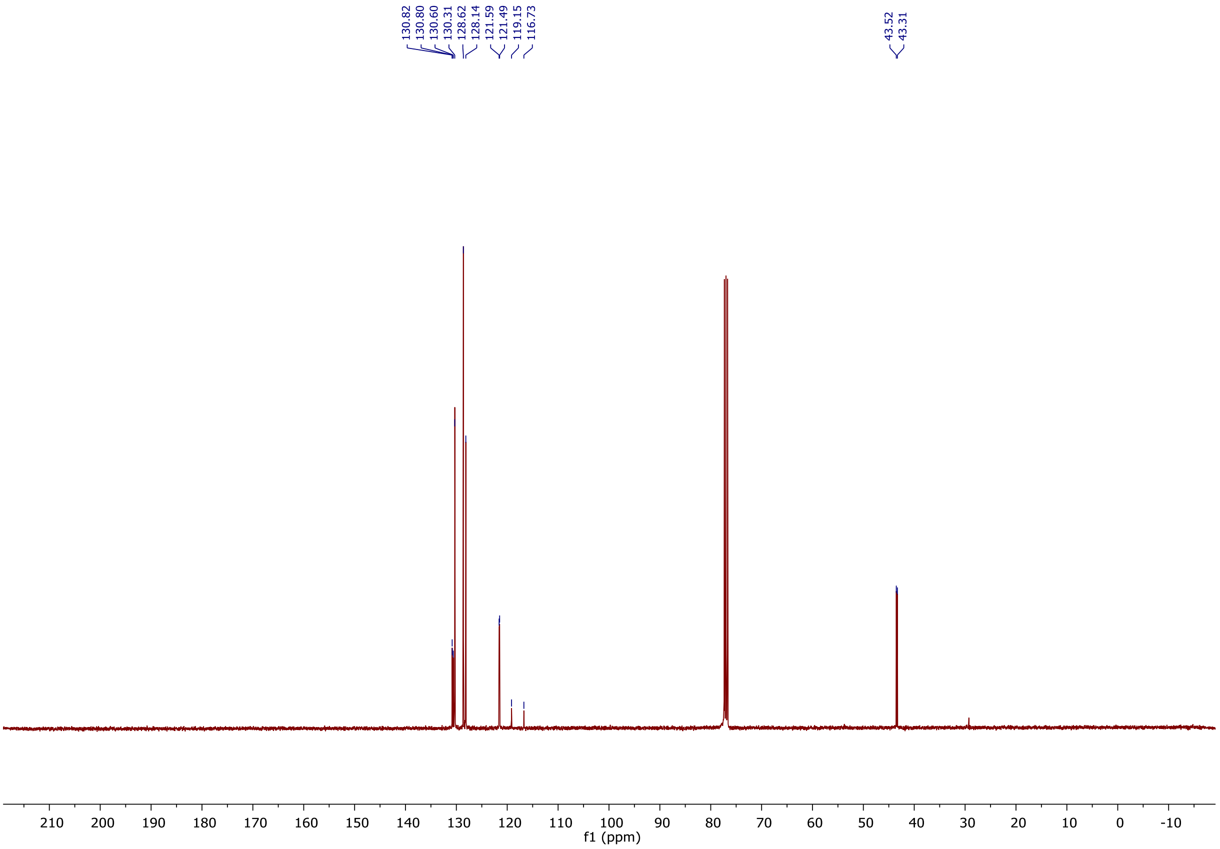

1-Chloro-4-(2-fluoro-2-nitrobut-3-en-1-yl)benzene (1p)

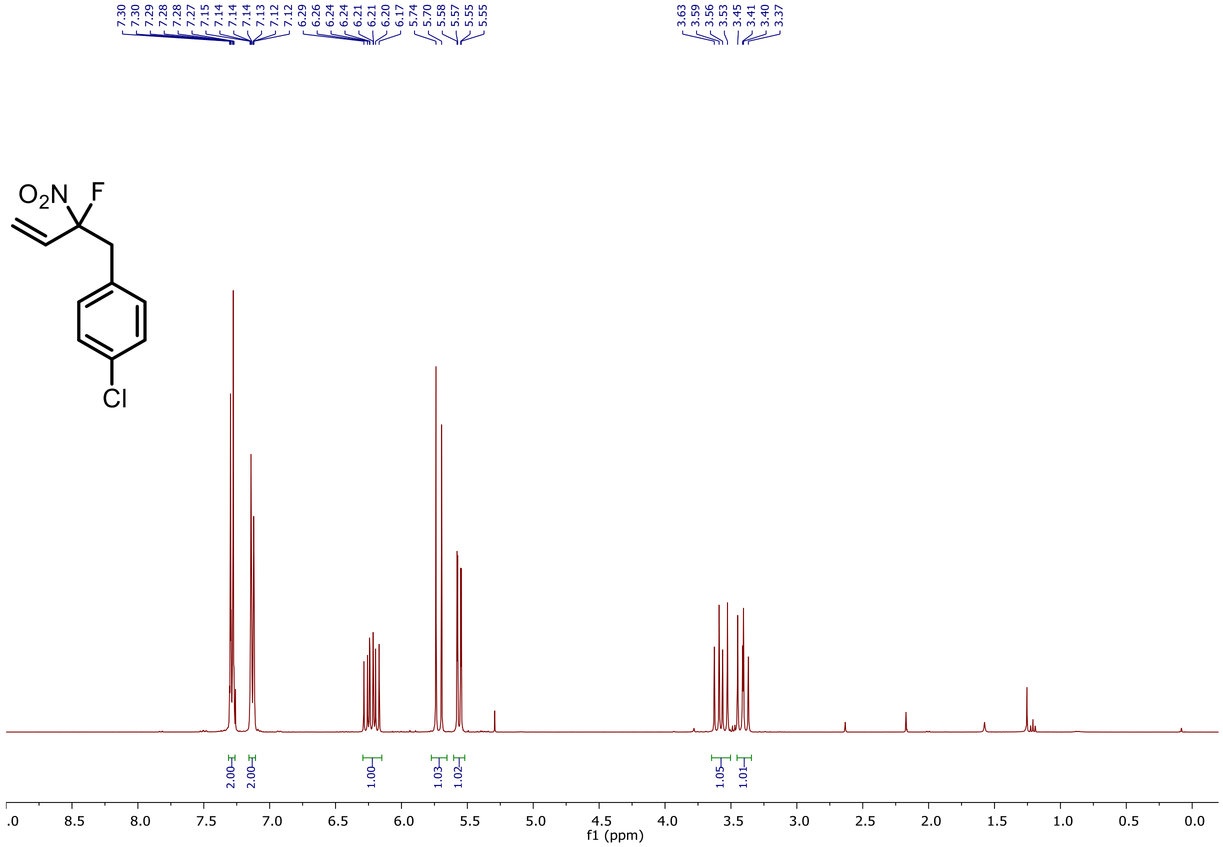

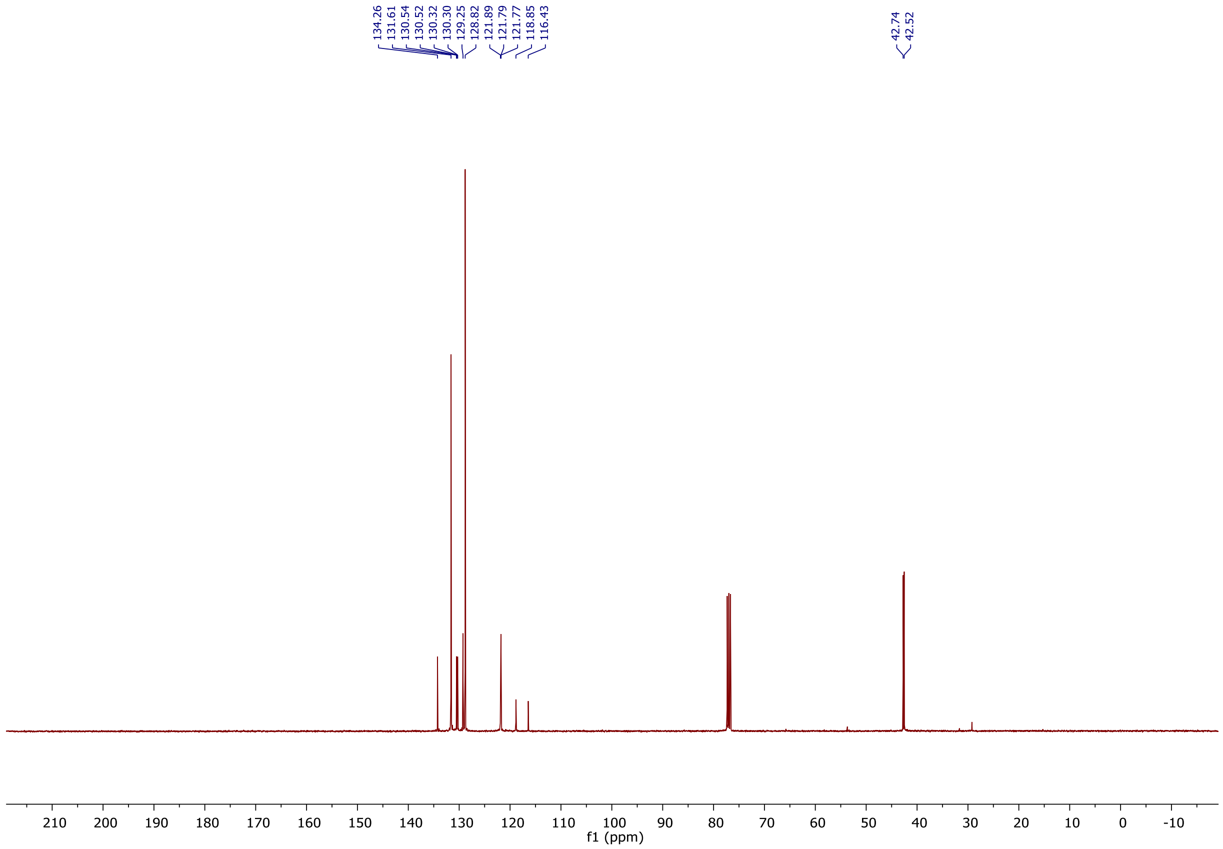

4-(2-Fluoro-2-nitrobut-3-en-1-yl)benzonitrile (1q)

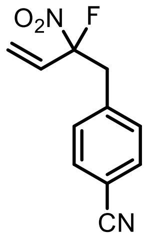

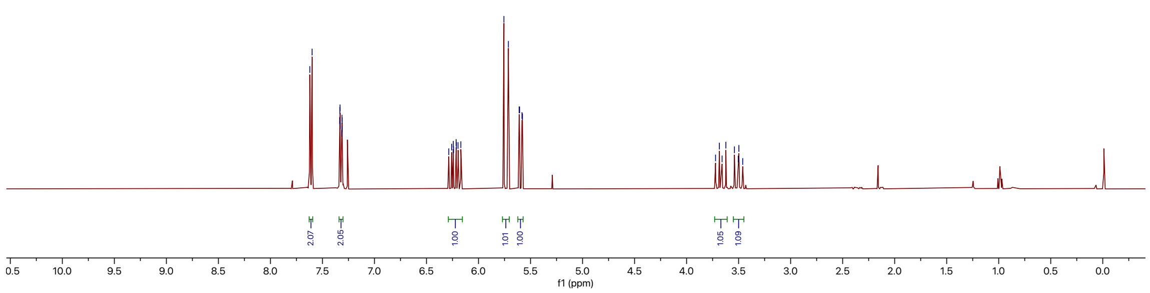

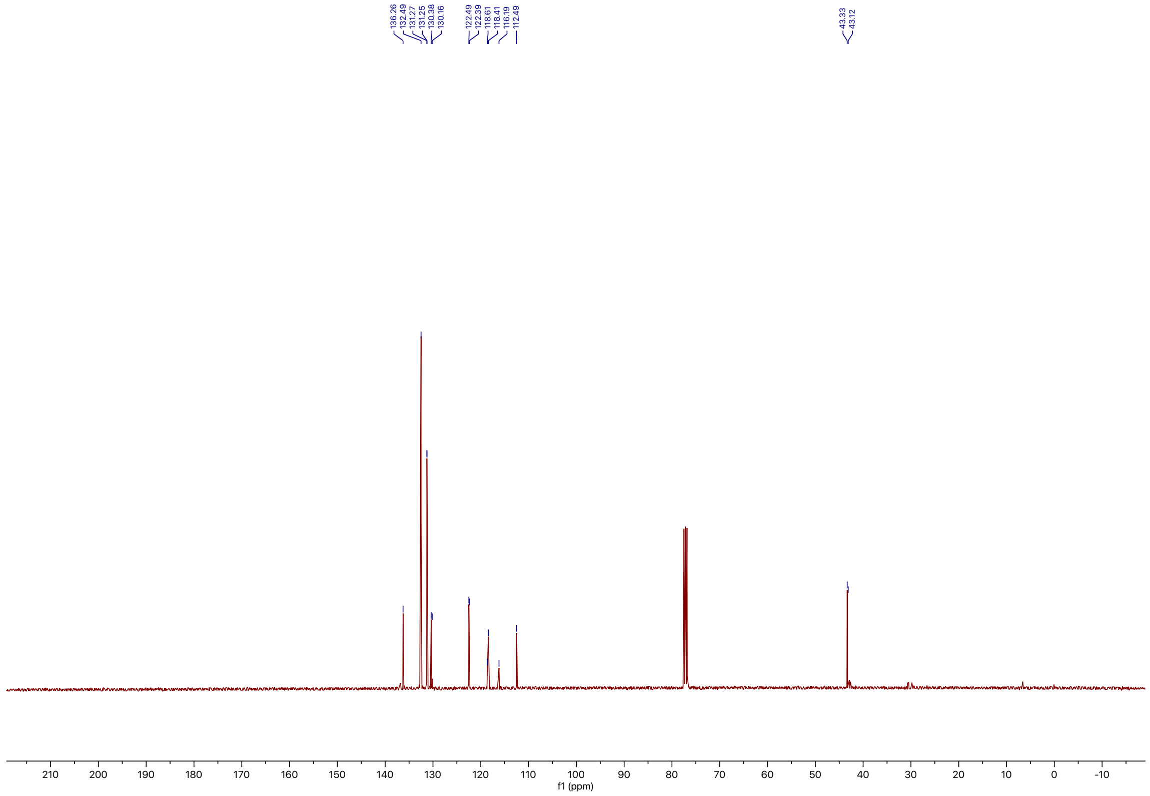

1-(2-Fluoro-2-nitrobut-3-en-1-yl)-4-(trifluoromethyl)benzene (1r)

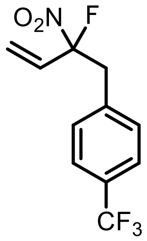

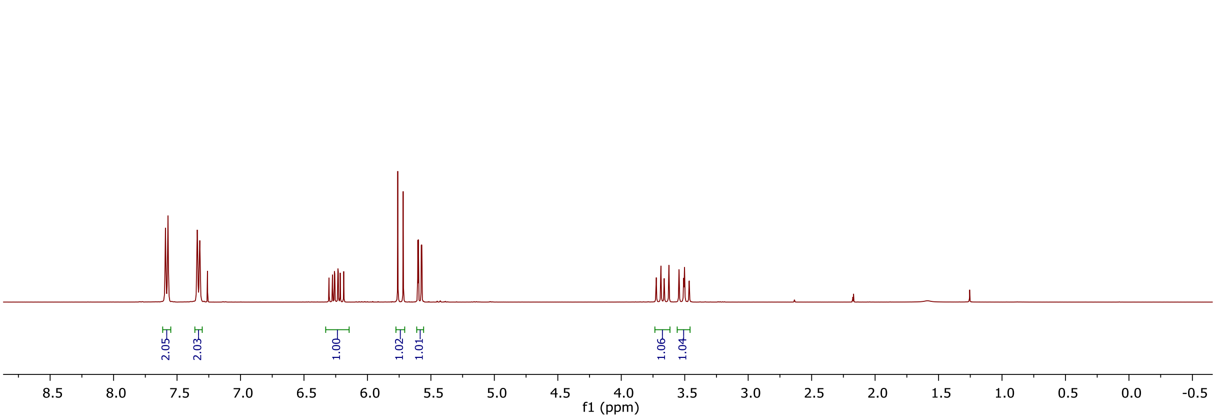

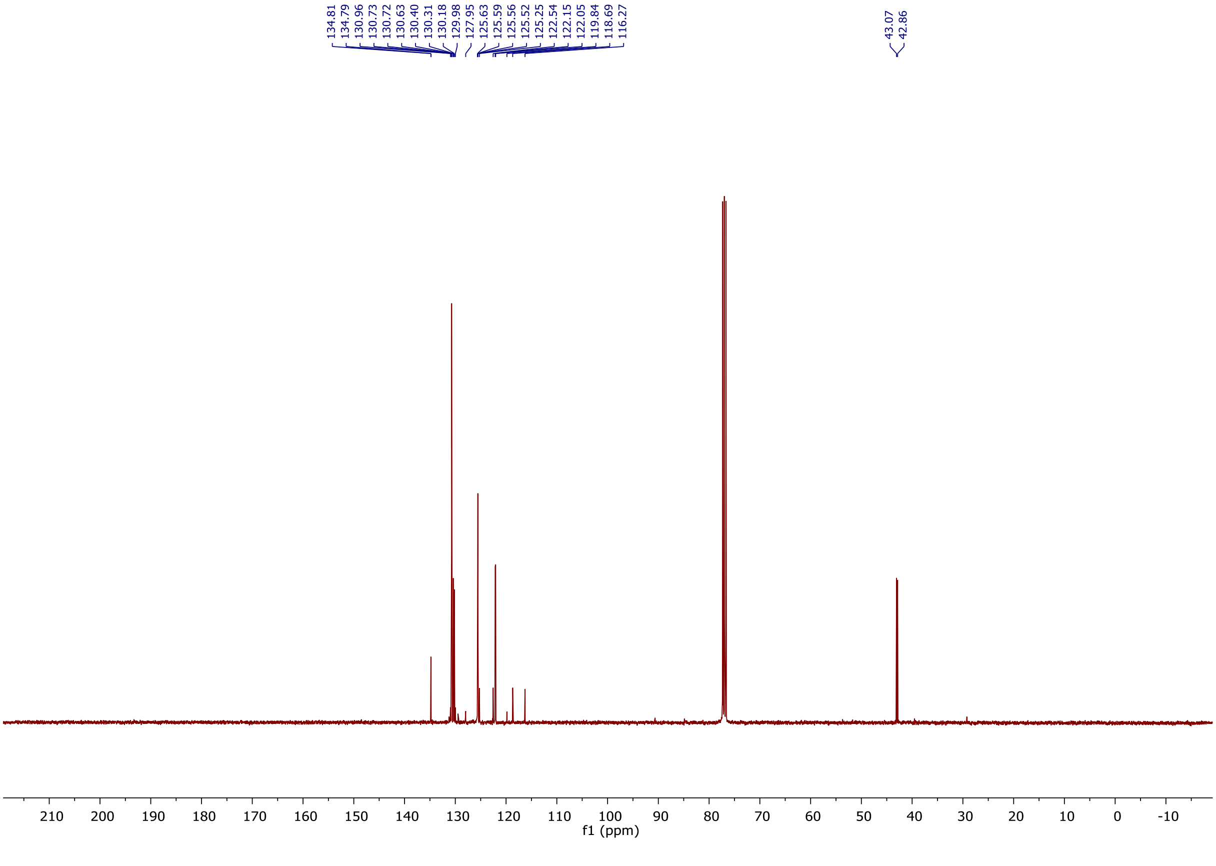

1-(2-Fluoro-2-nitrobut-3-en-1-yl)-4-methoxybenzene (1s)

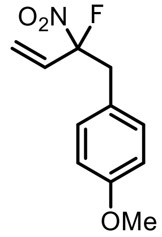

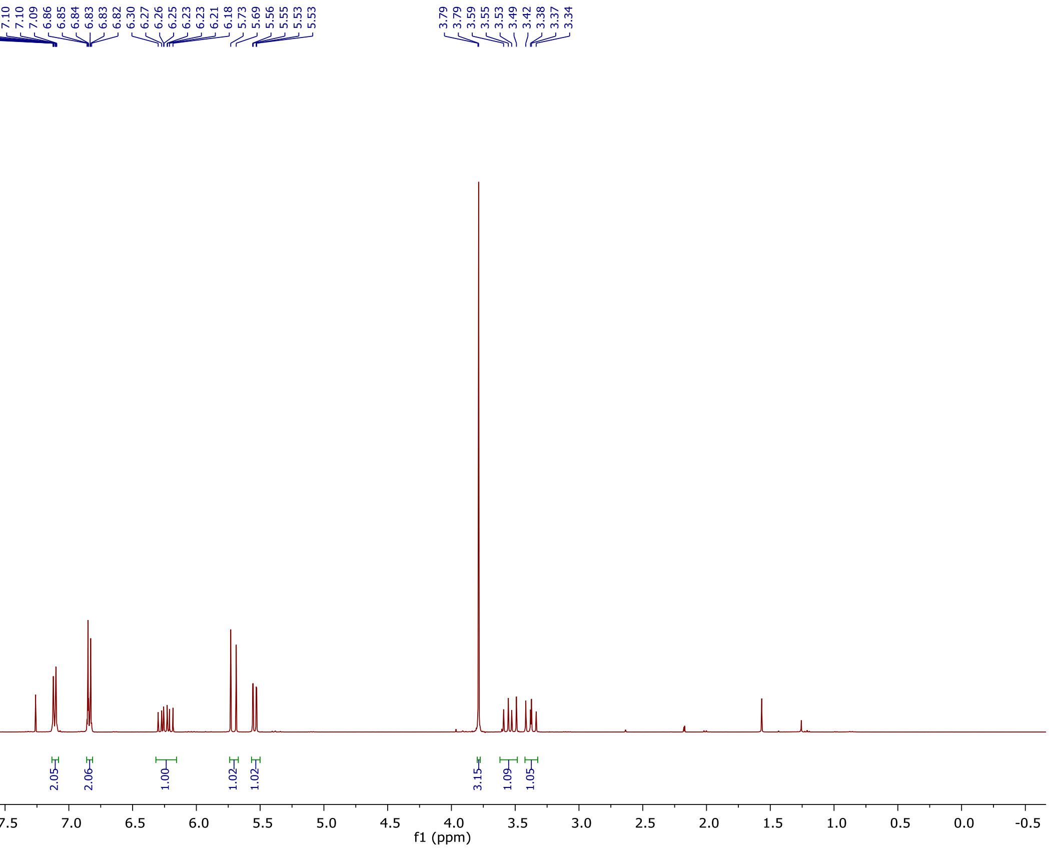

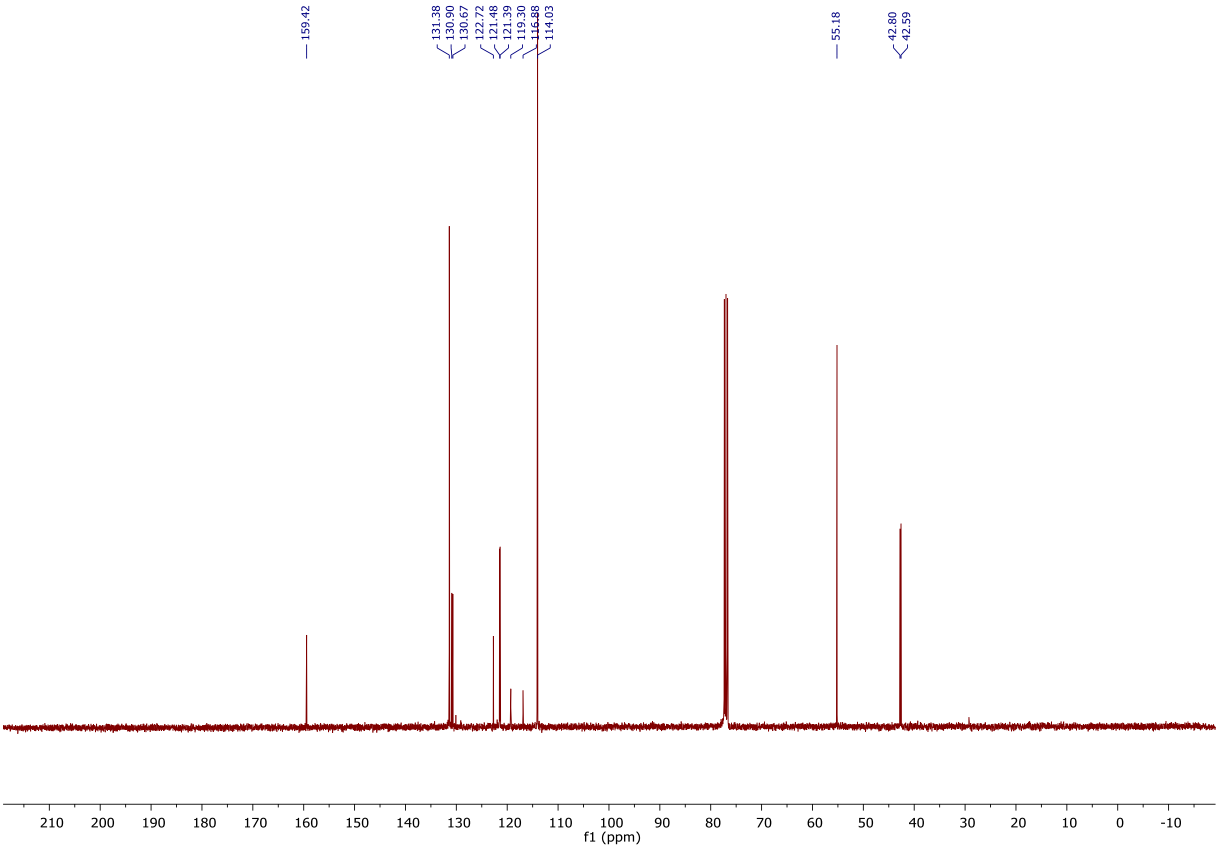

1-(2-Fluoro-2-nitrobut-3-en-1-yl)-3-methoxybenzene (1t)

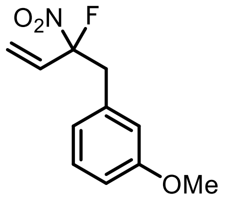

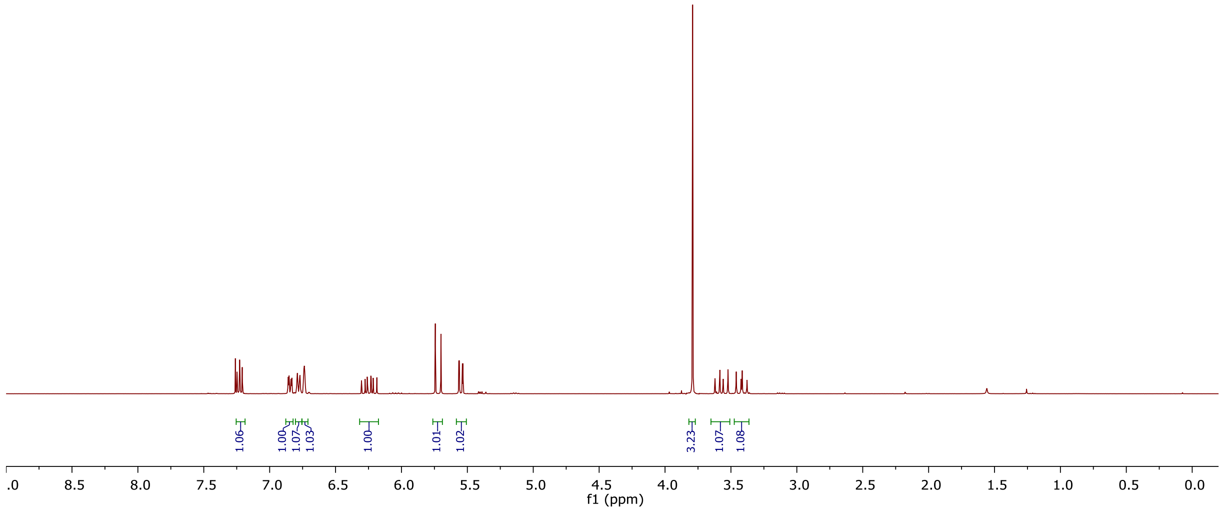

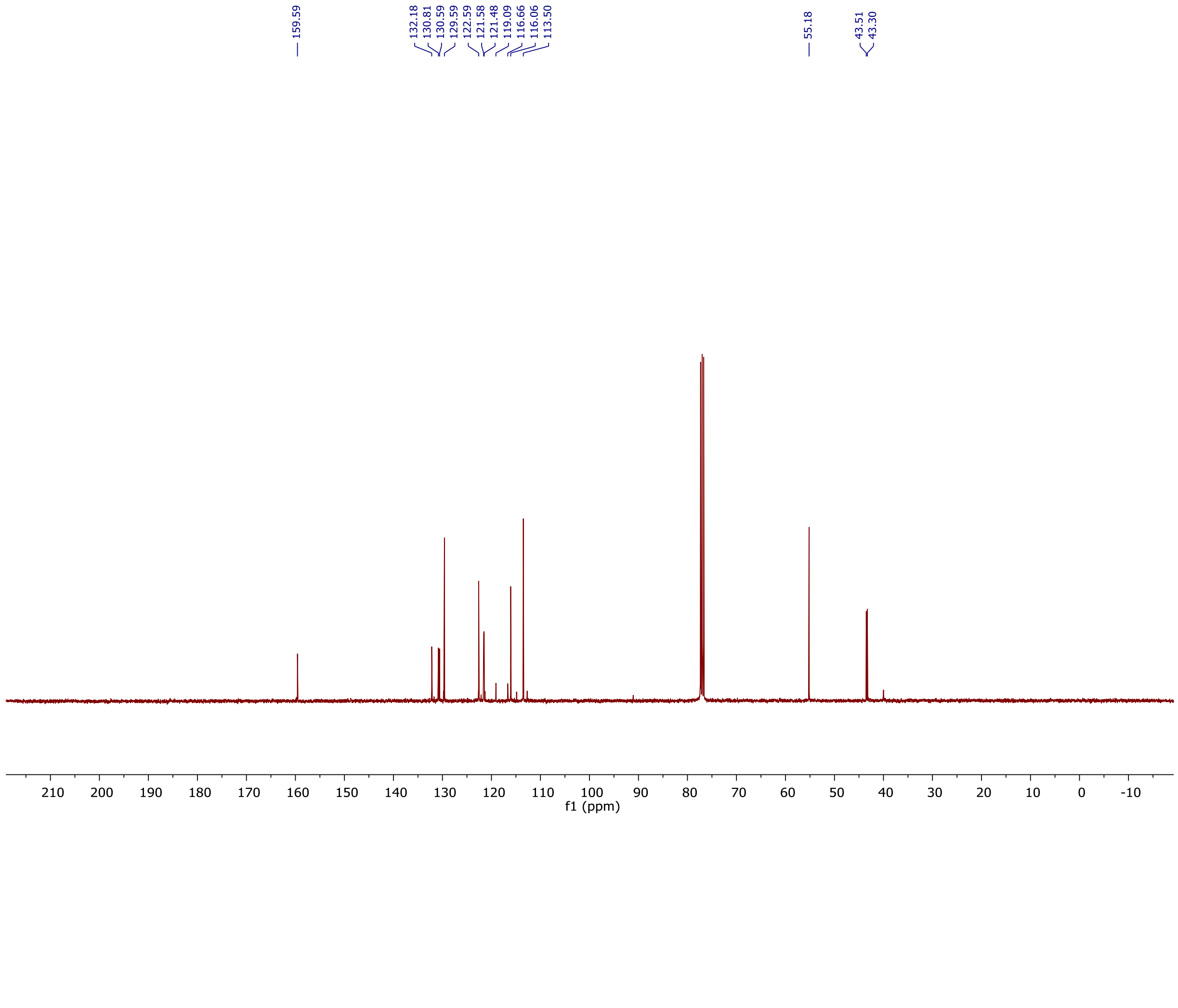

1-(2-Fluoro-2-nitrobut-3-en-1-yl)-2-methoxybenzene (1u)

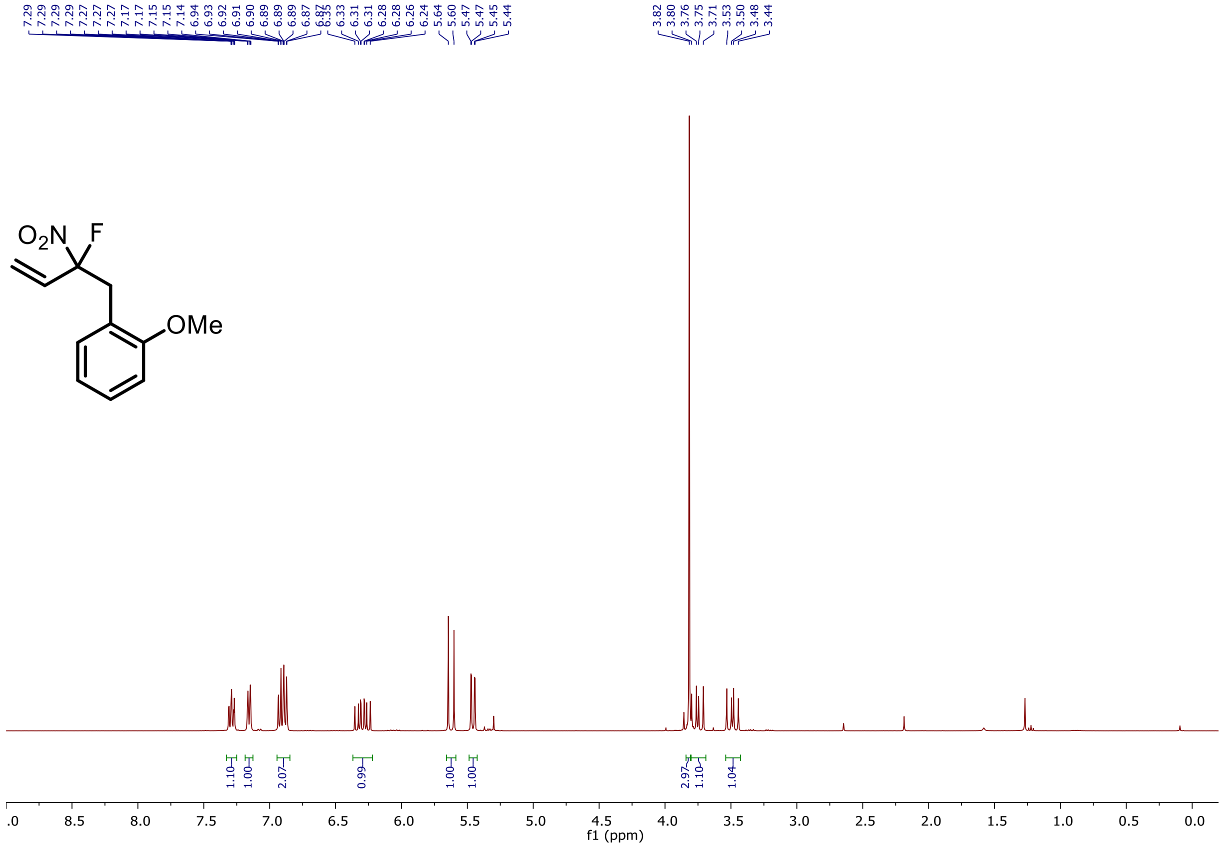

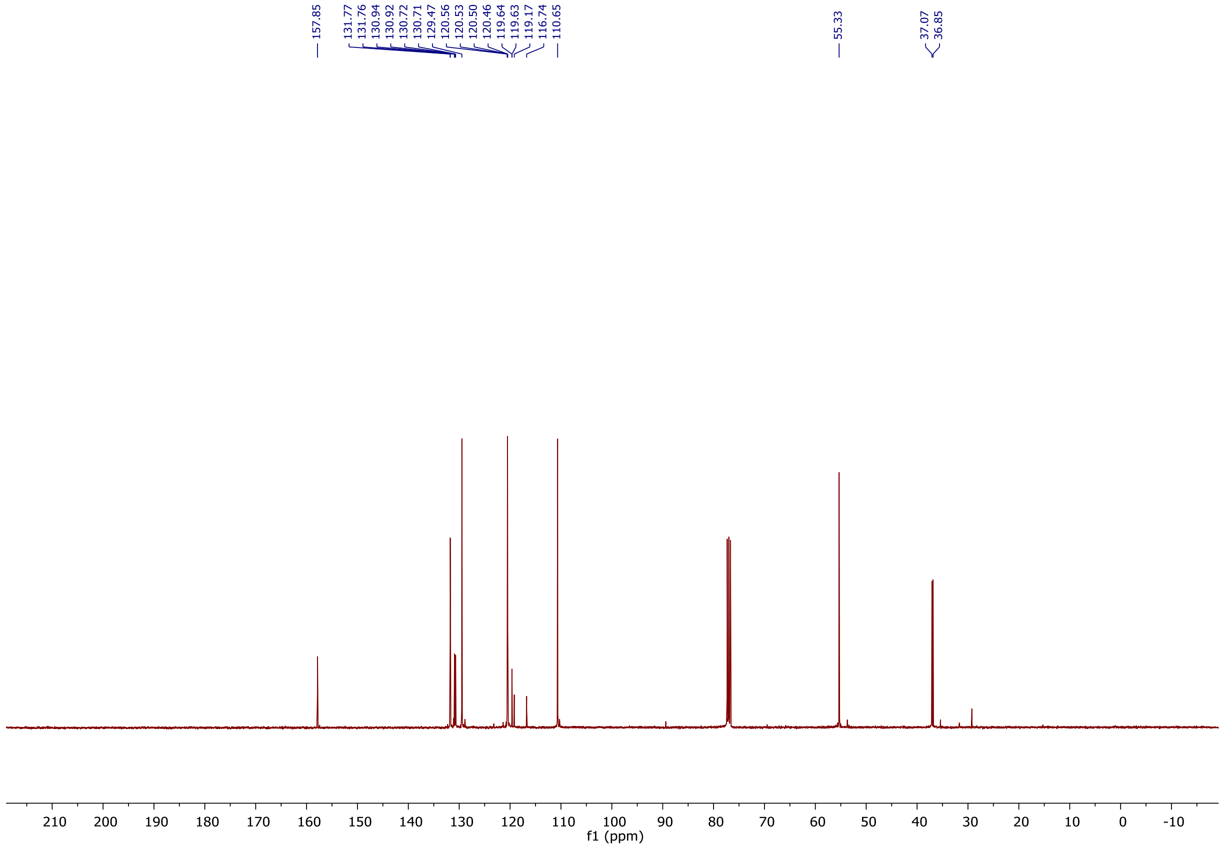

2-(2-Fluoro-2-nitrobut-3-en-1-yl)furan (1v)

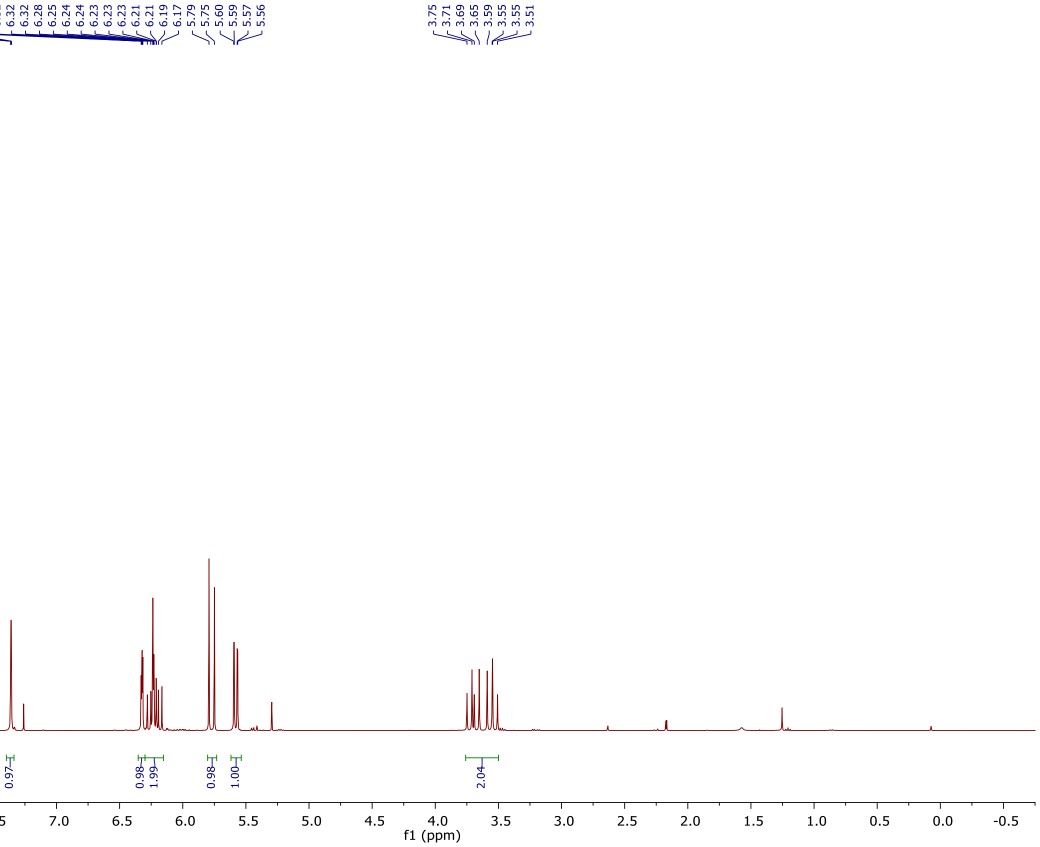

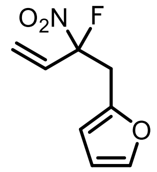

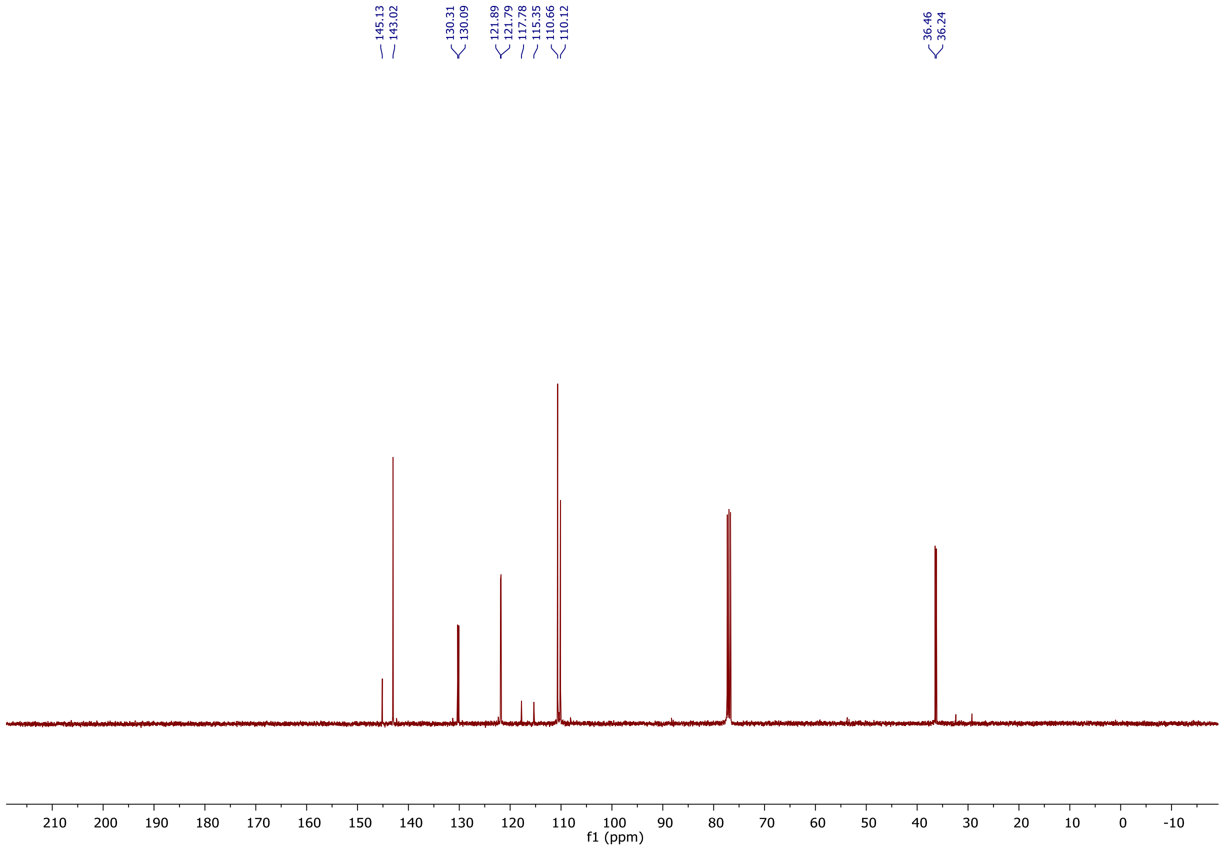

(3-Fluoro-3-nitrobut-1-en-2-yl)benzene (1w)

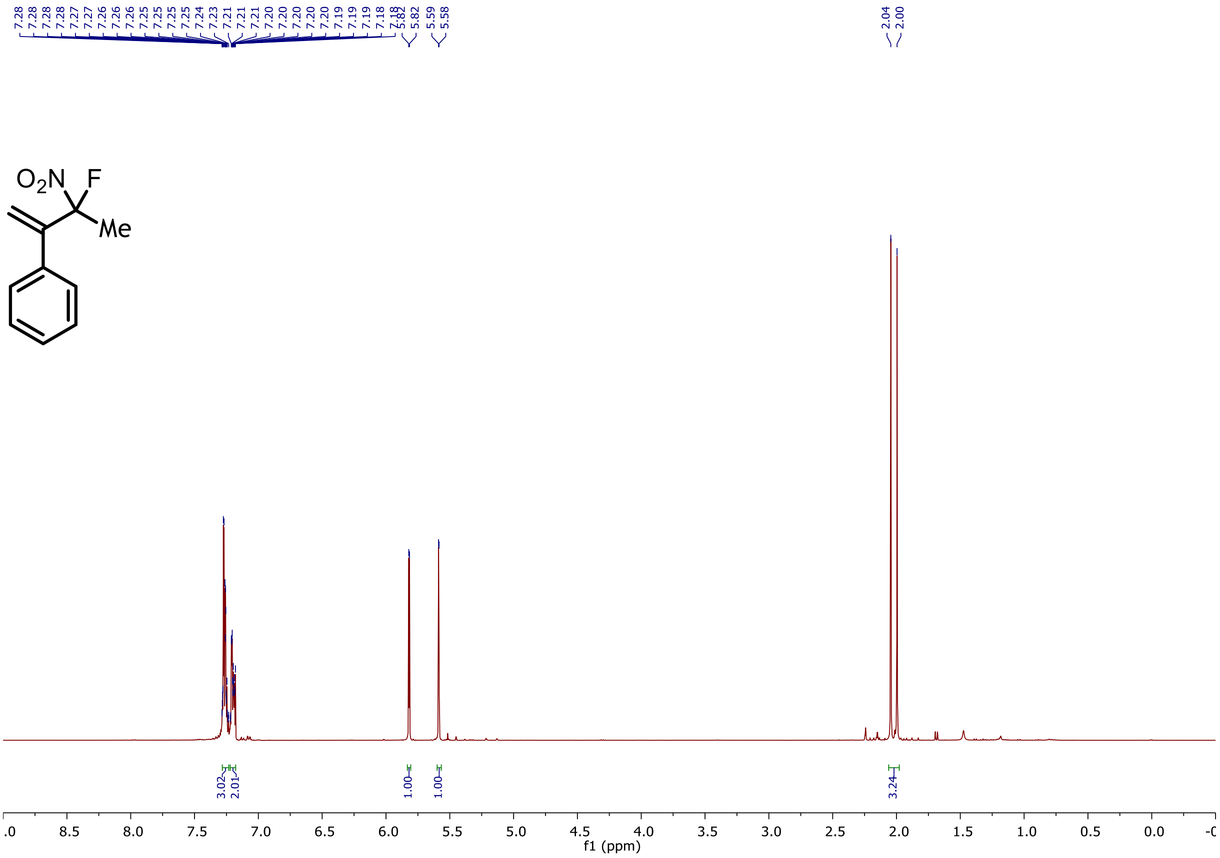

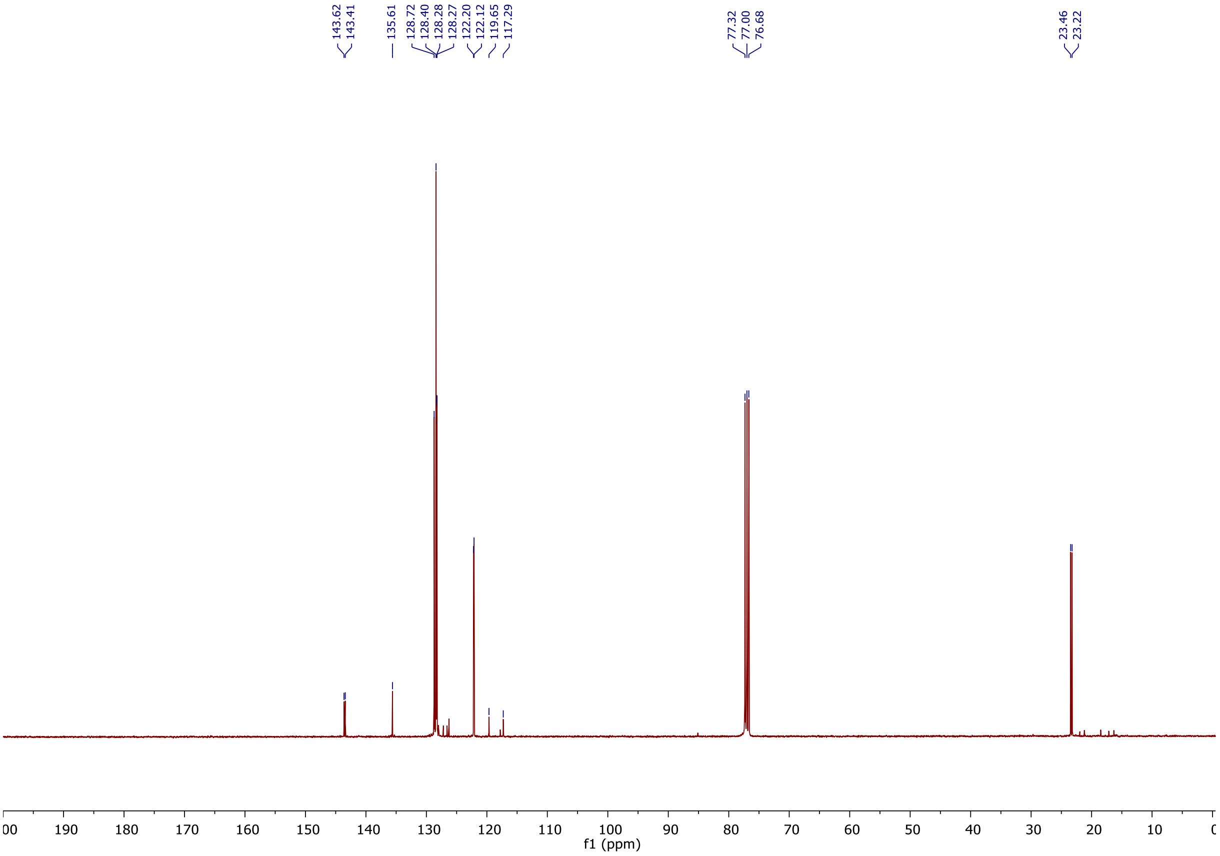

2-(2-Fluoro-2-nitrobut-3-en-1-yl)naphthalene (1x)

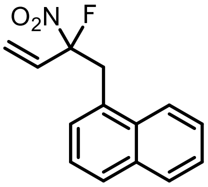

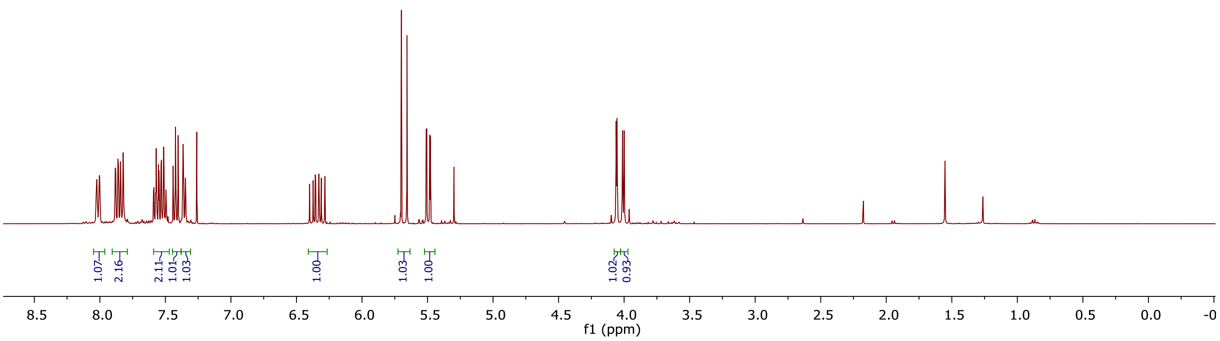

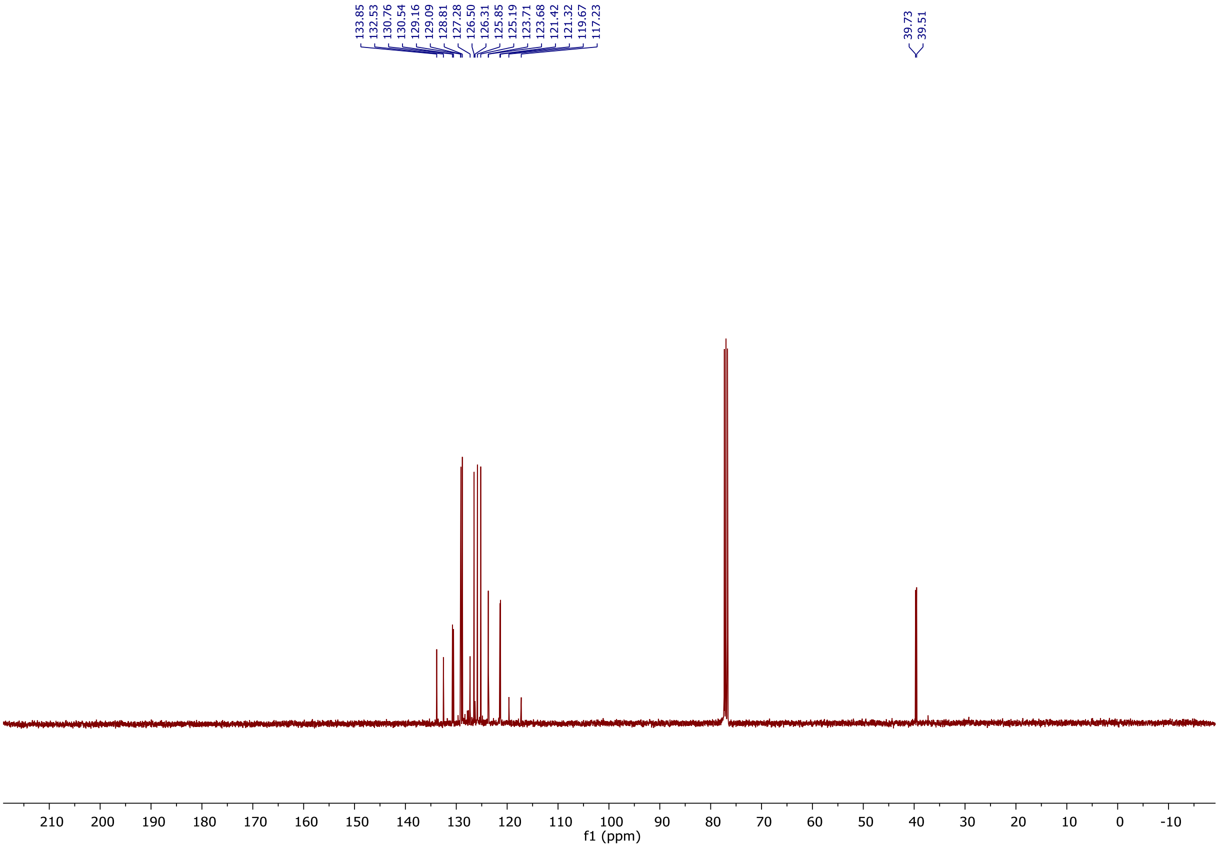

Dimethyl 4-(2-(4,4,5,5-tetramethyl-1,3,2-dioxaborolan-2-yl)ethylidene)heptanedioate (2a)

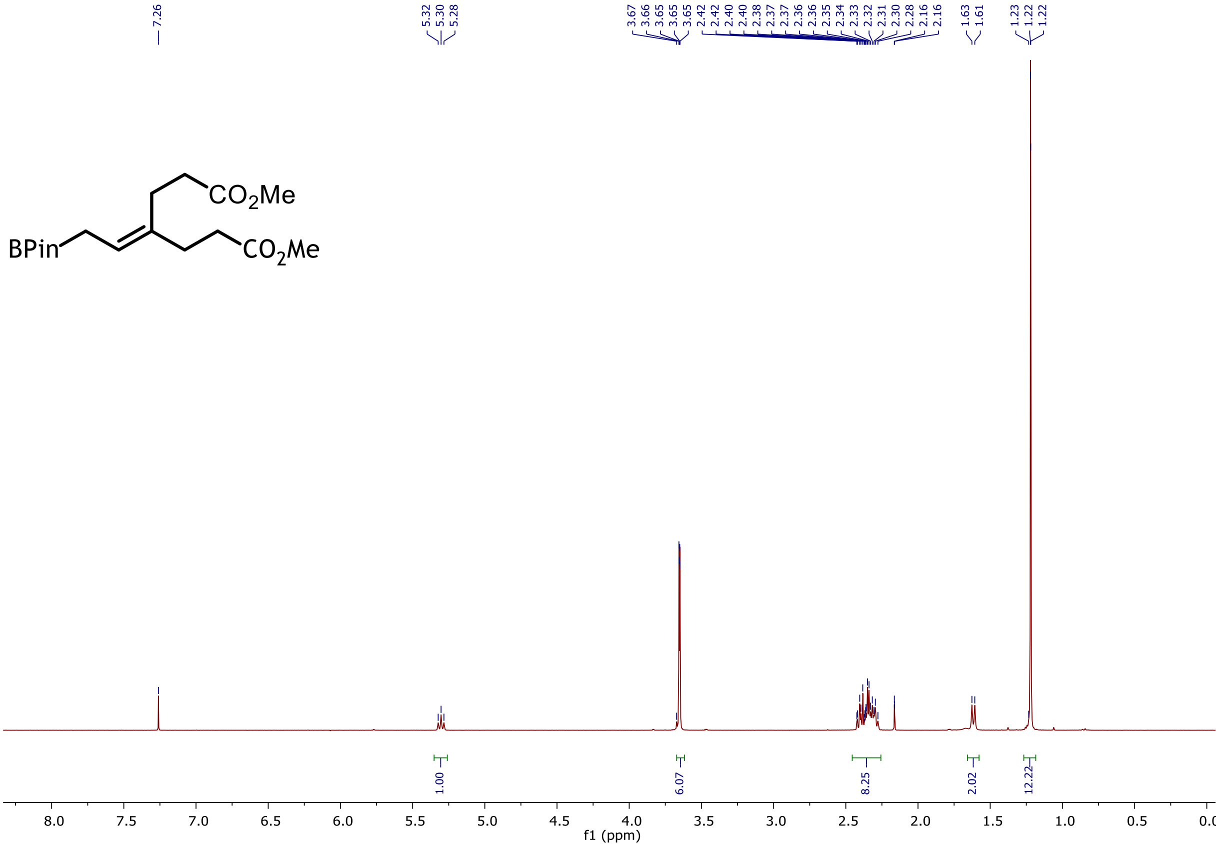

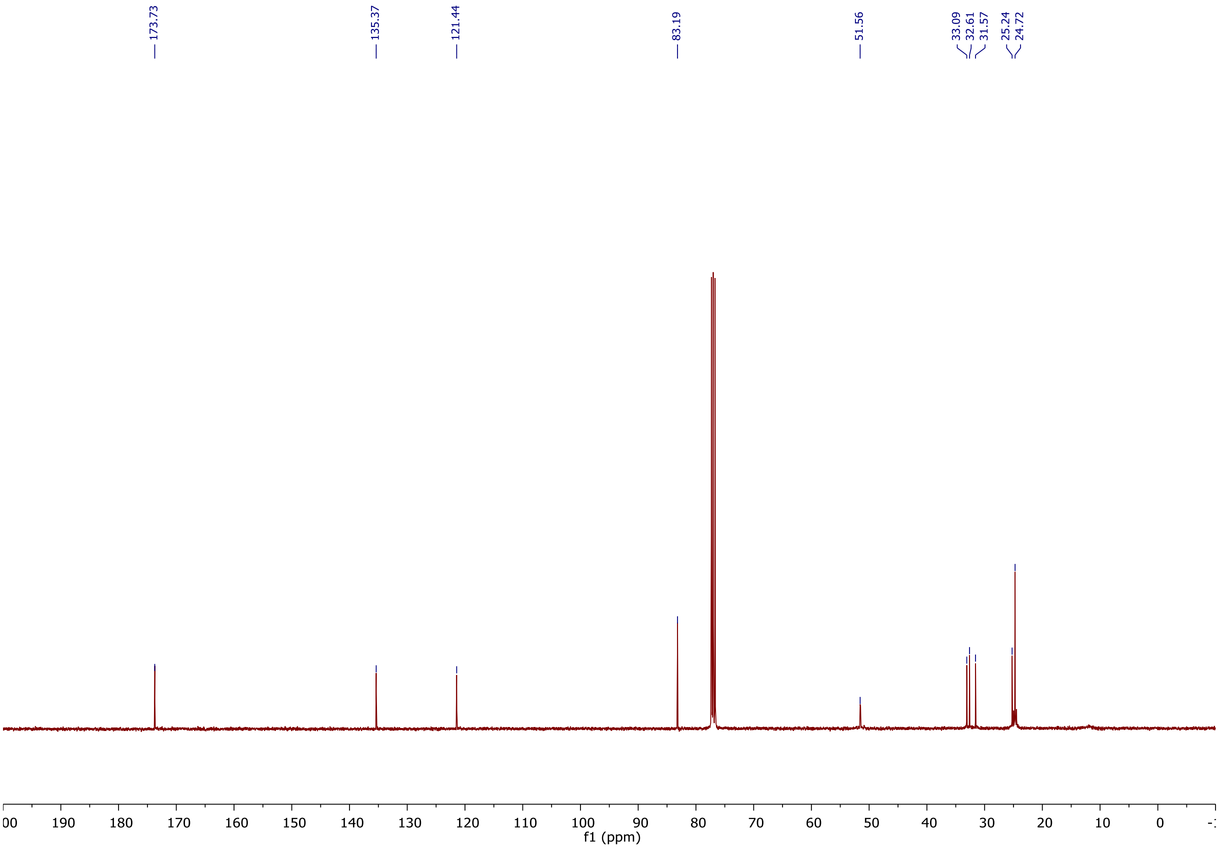

Methyl (*E*)-4-methyl-6-(4,4,5,5-tetramethyl-1,3,2-dioxaborolan-2-yl)hex-4-enoate (2b)

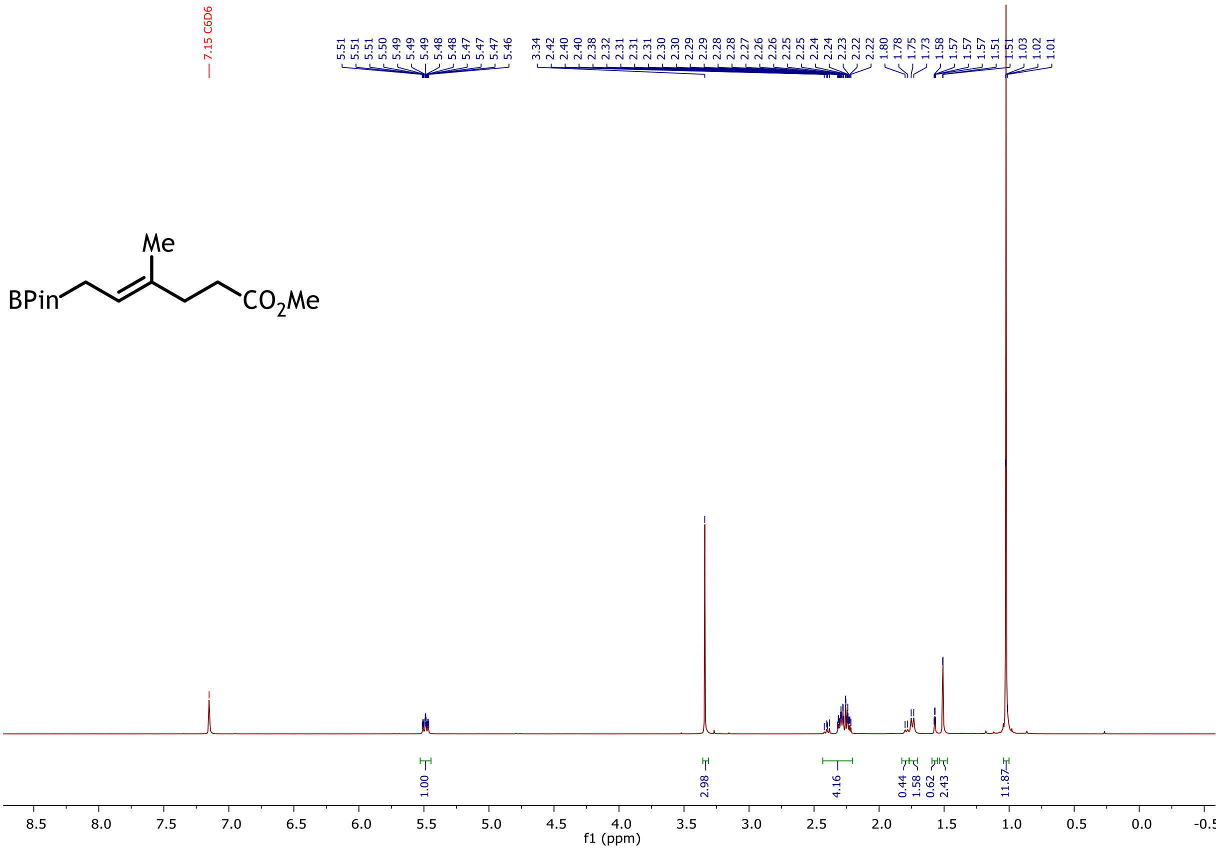

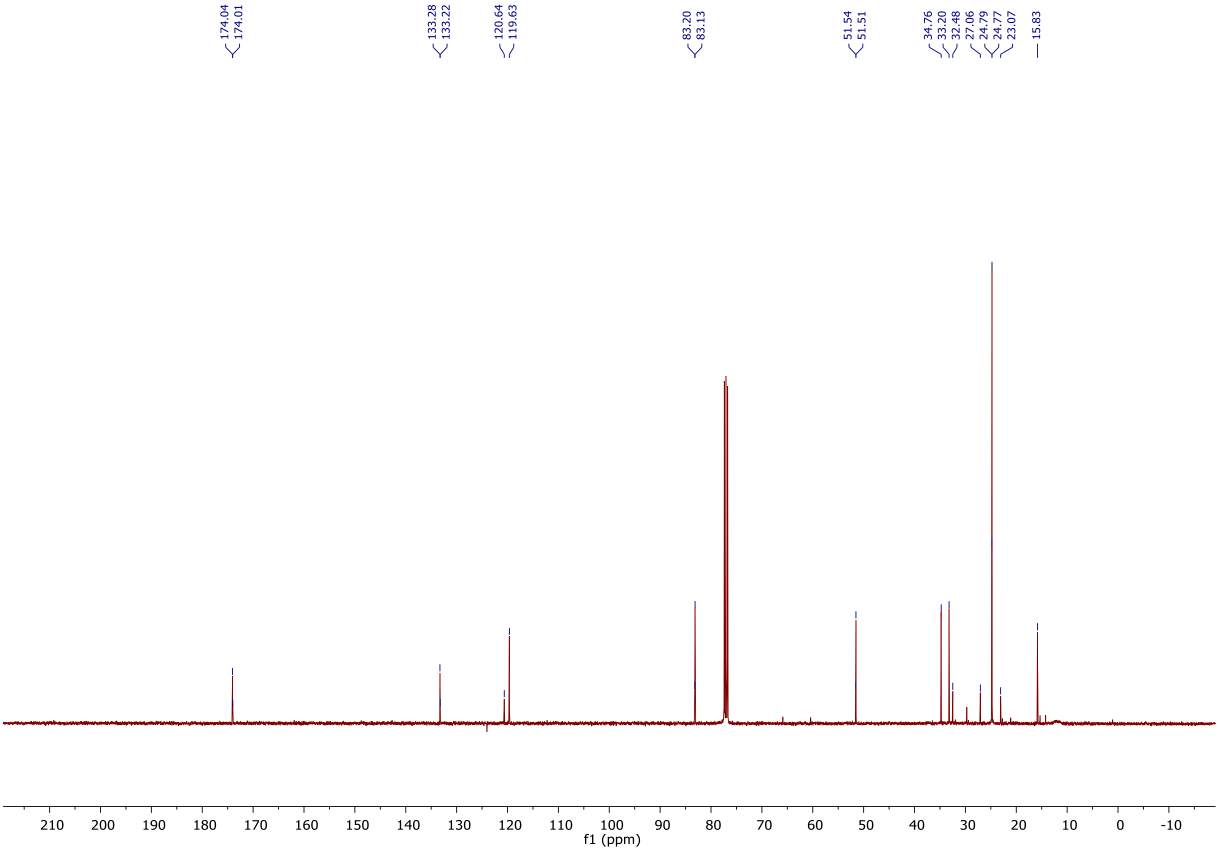

(*E*)-4-Methyl-6-(4,4,5,5-tetramethyl-1,3,2-dioxaborolan-2-yl)hex-4-enenitrile (2c)

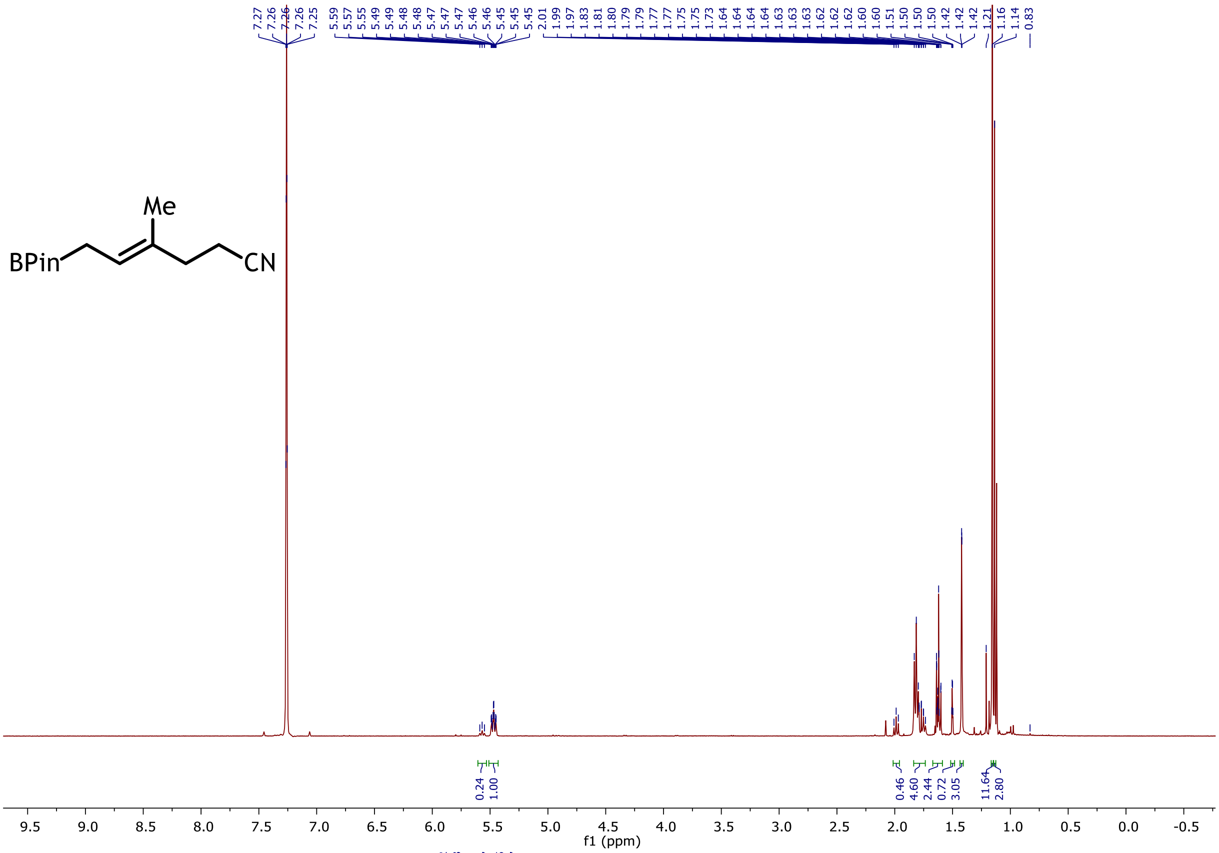

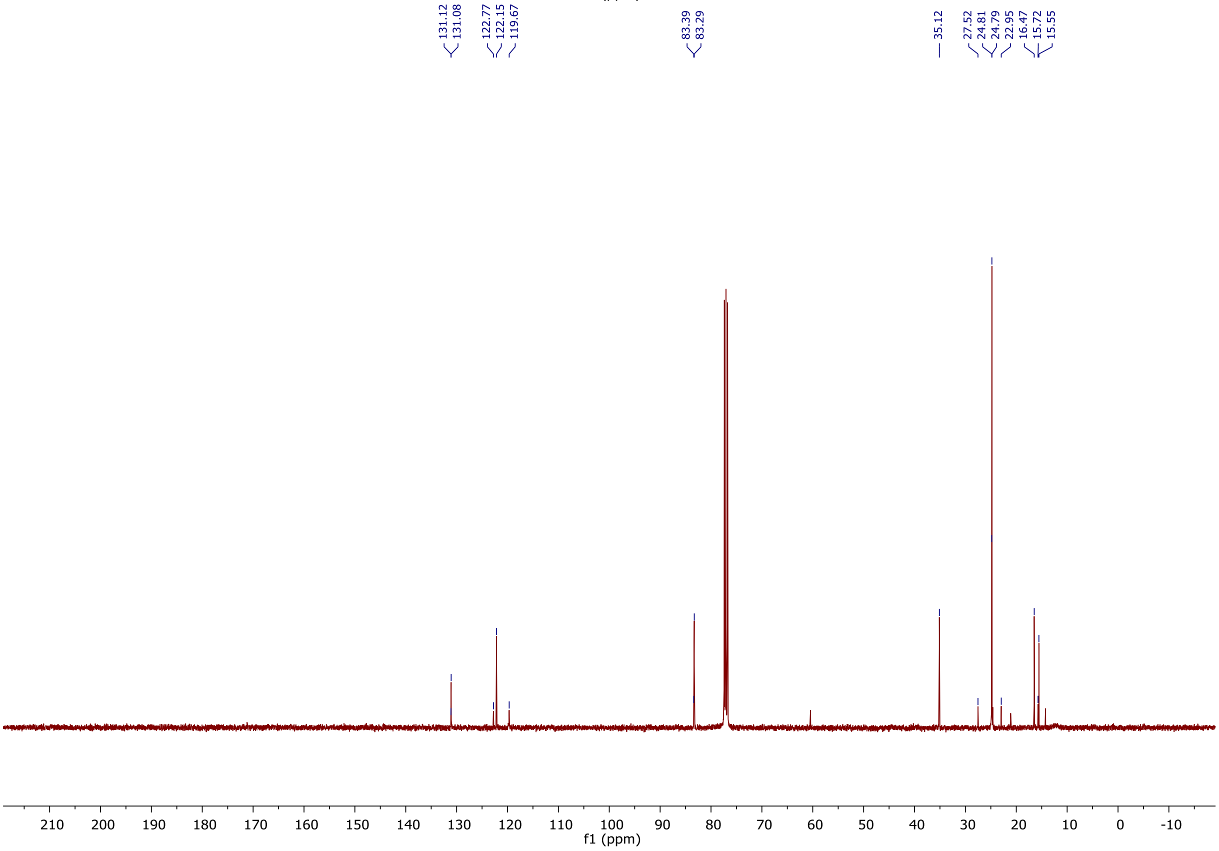

(*E*)-4,4,5,5-Tetramethyl-2-(3-methyl-5-(phenylsulfonyl)pent-2-en-1-yl)-1,3,2-dioxaborolane (2d)

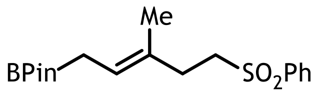

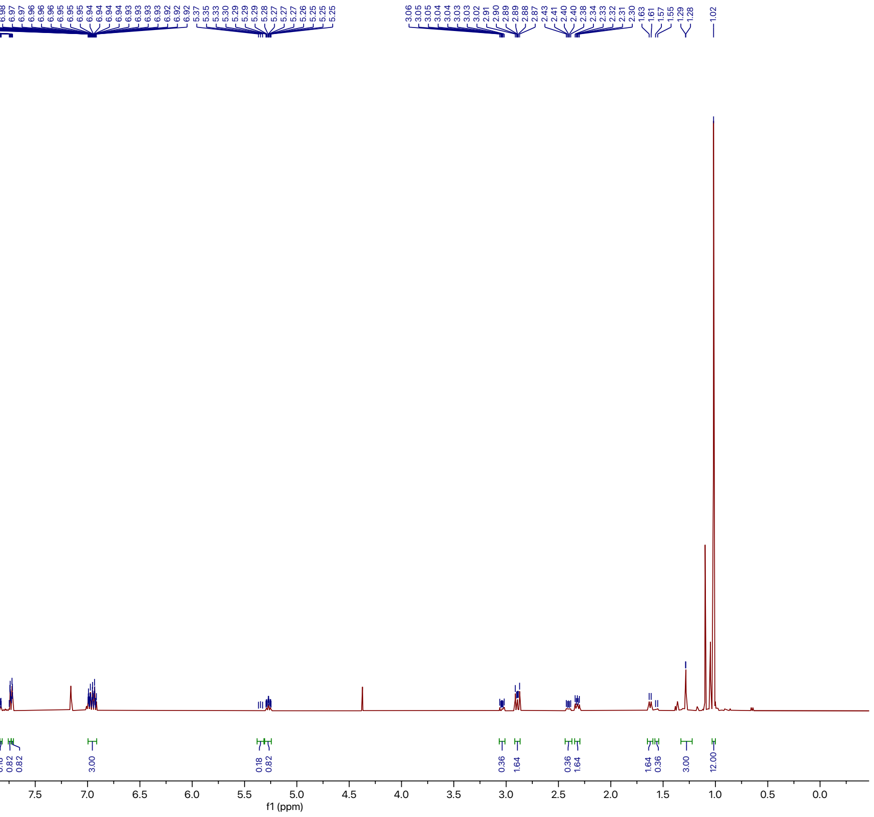

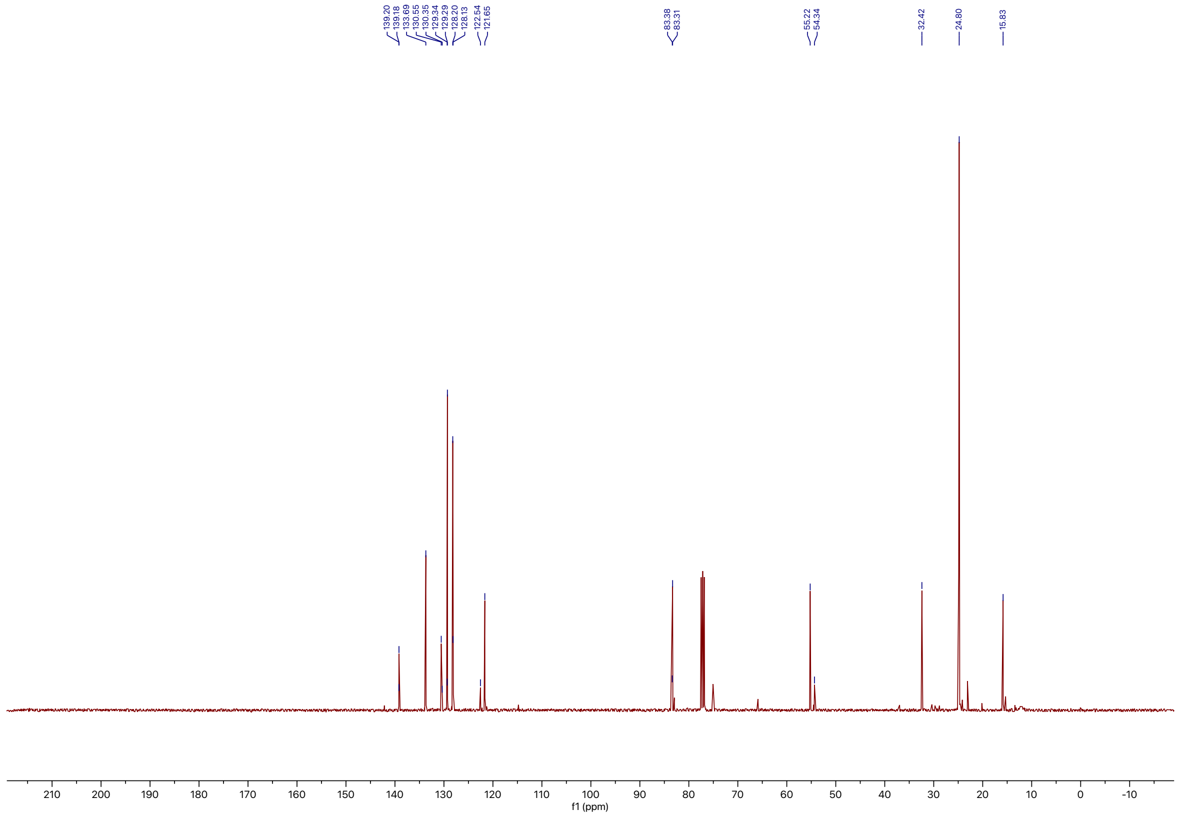

(*E*)-Tert-butyldimethyl((2-methyl-4-(4,4,5,5-tetramethyl-1,3,2-dioxaborolan-2-yl)but-2-en-1-yl)oxy)silane (2e)

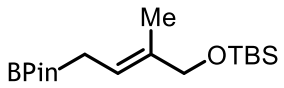

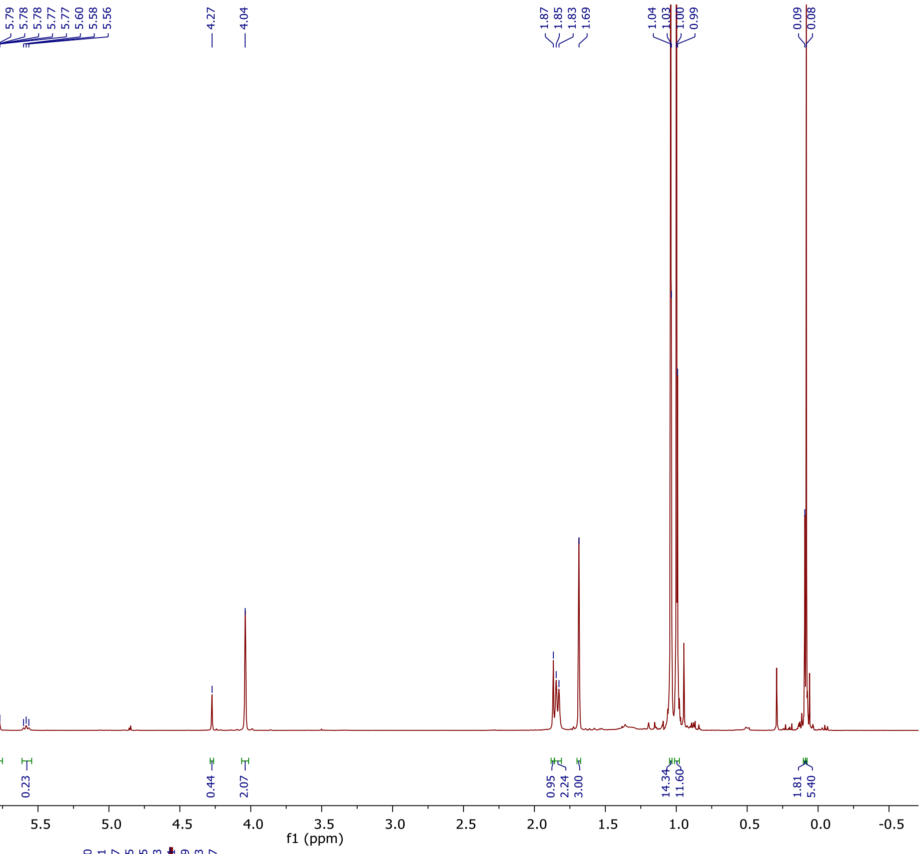

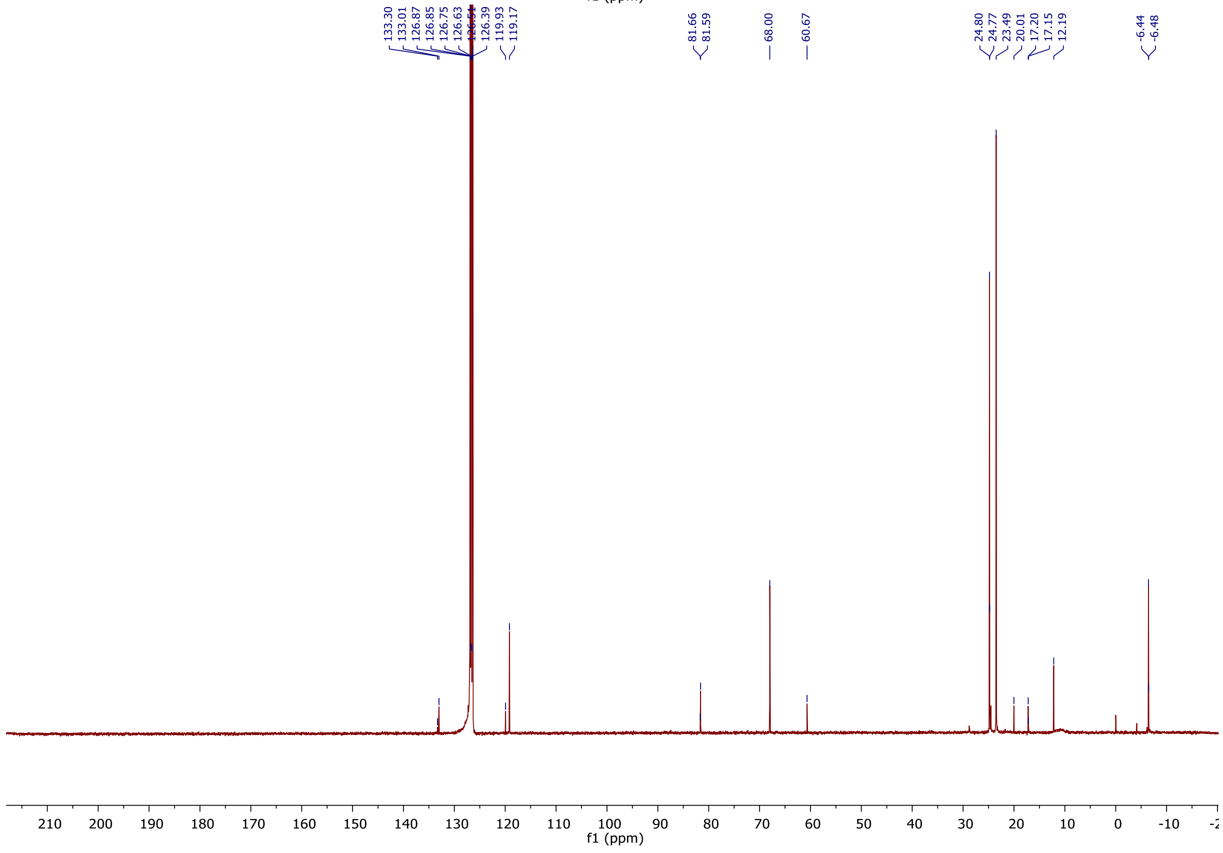

Methyl (*Z*)-4-(4-methoxybenzyl)-6-(4,4,5,5-tetramethyl-1,3,2-dioxaborolan-2-yl)hex-4-enoate (2f)

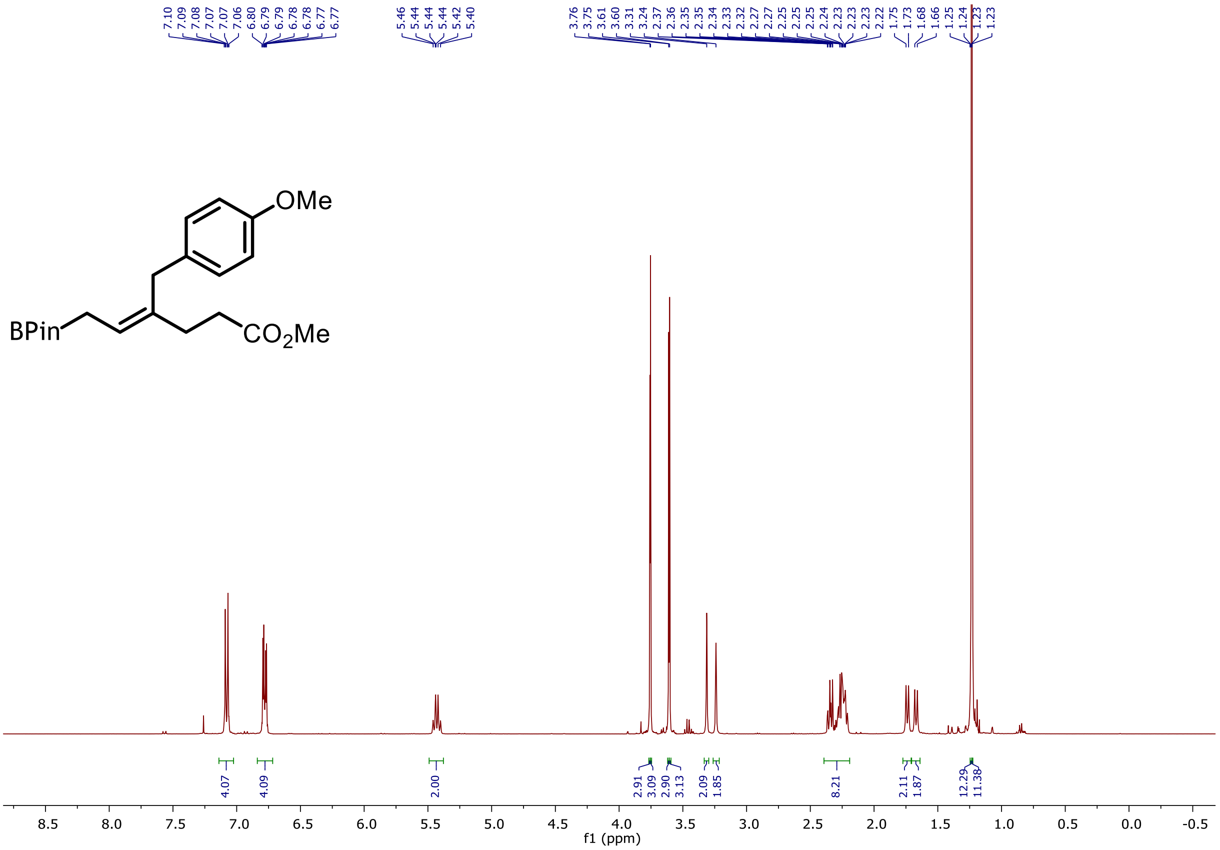

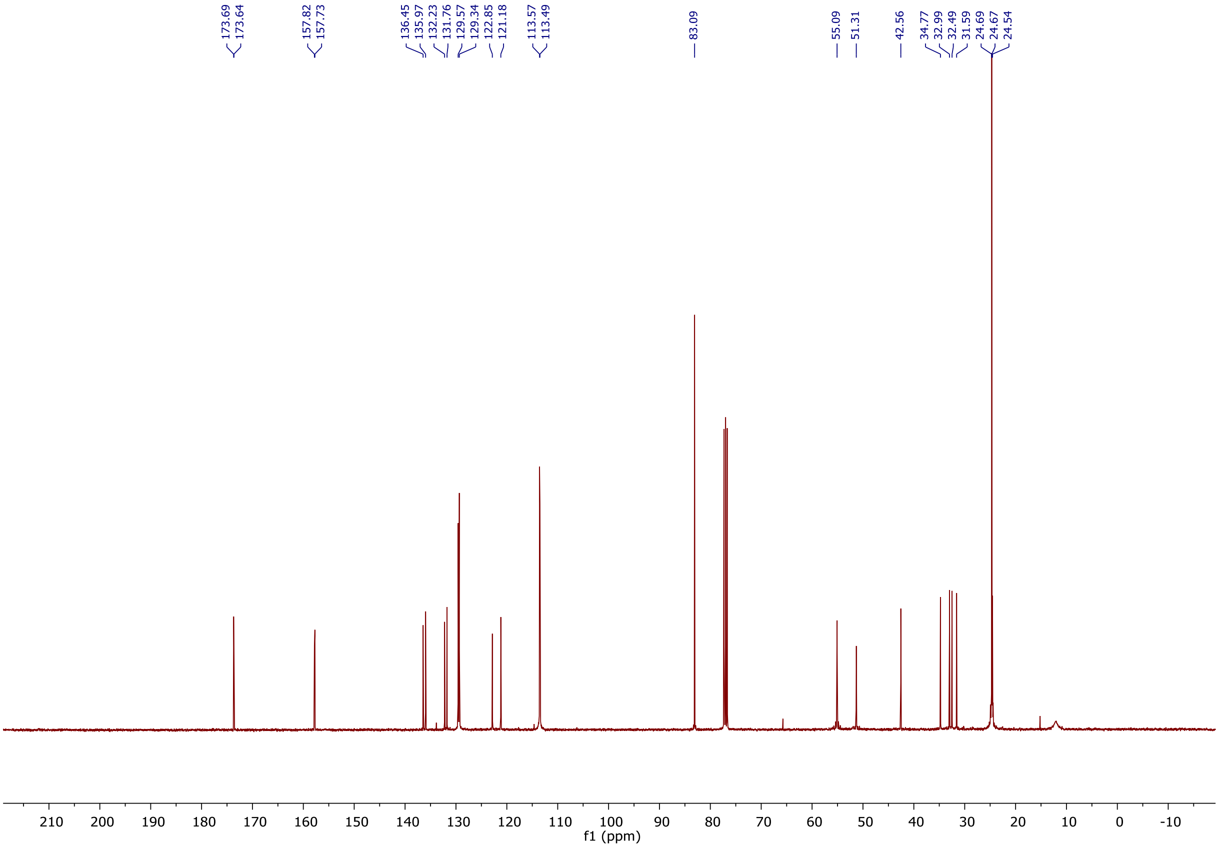

methyl 7-oxo-4-(2-(4,4,5,5-tetramethyl-1,3,2-dioxaborolan-2-yl)ethylidene)octanoate

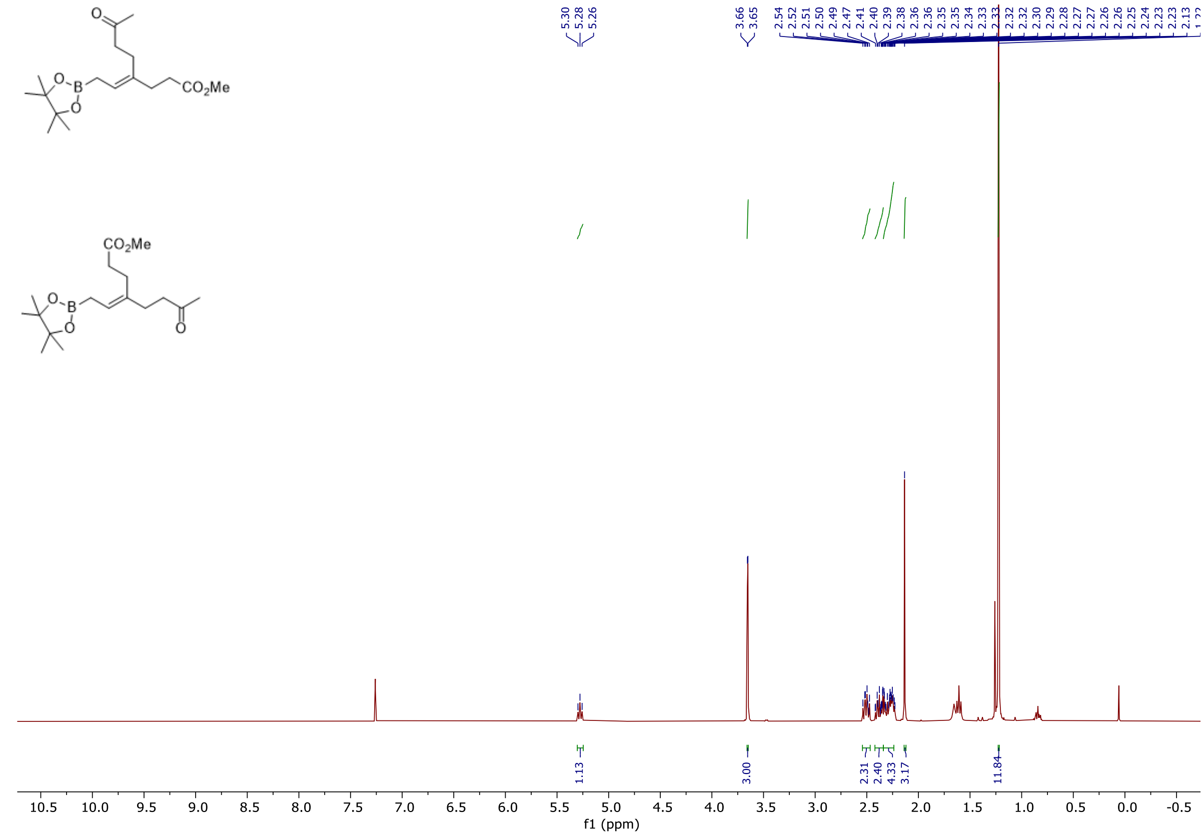

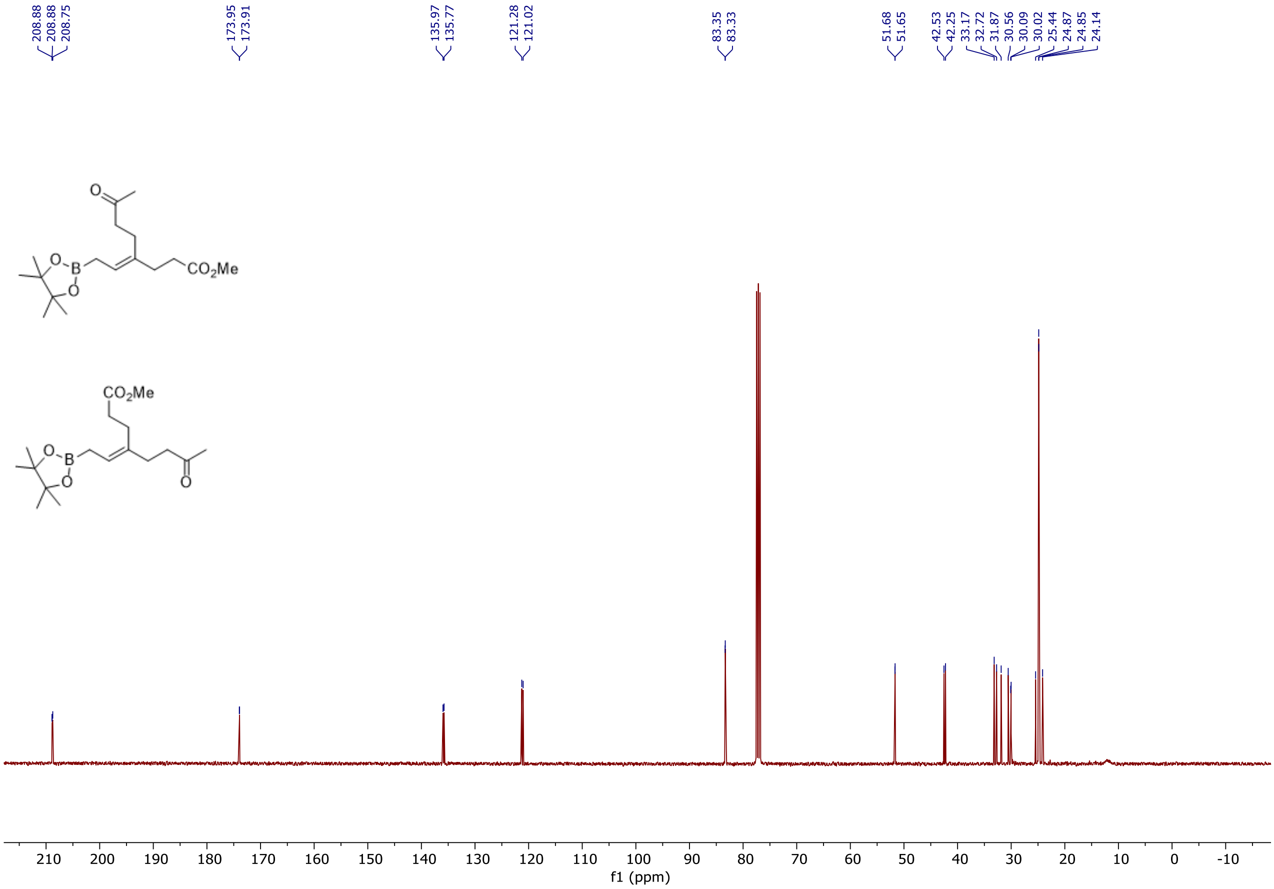

(*E*)-((2-Benzyl-4-(4,4,5,5-tetramethyl-1,3,2-dioxaborolan-2-yl)but-2-en-1-yl)oxy)(tert-butyl)dimethylsilane (2h)

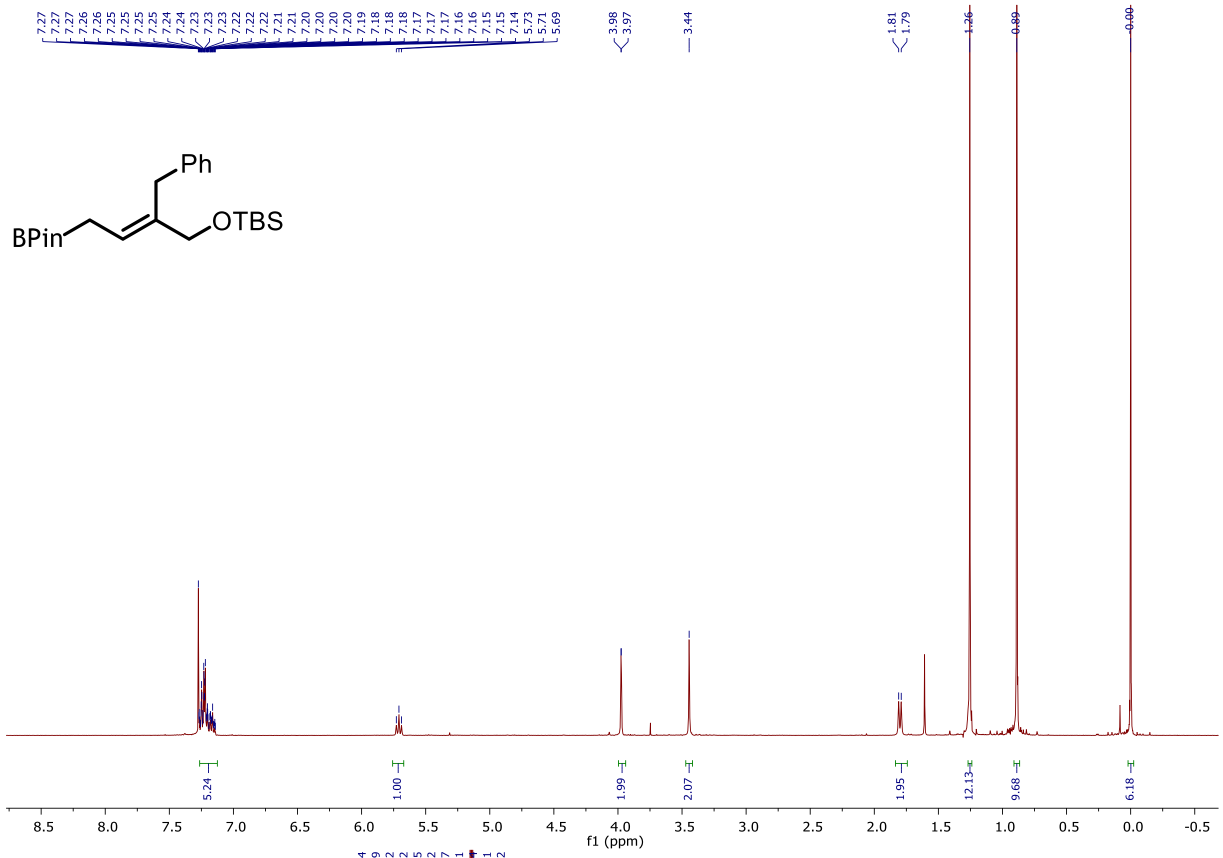

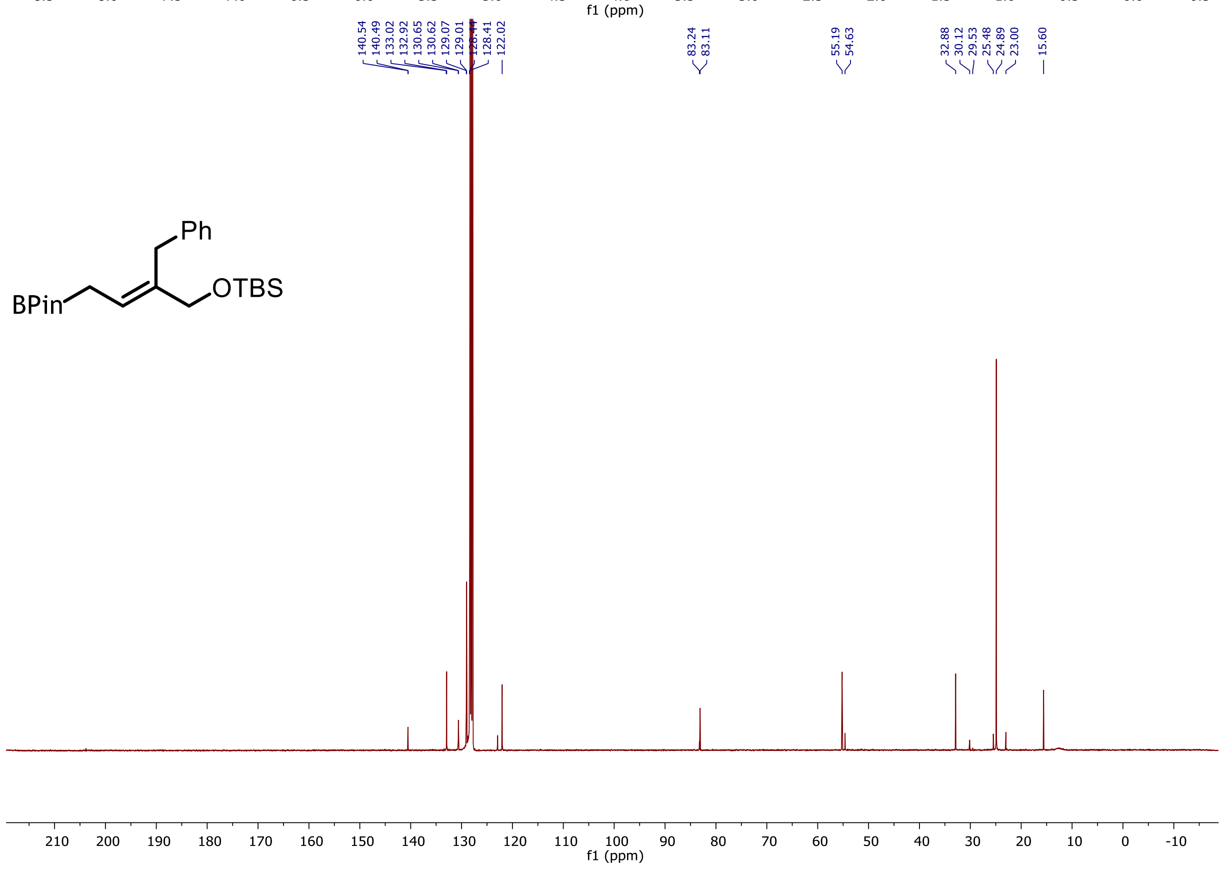

5-Benzyl-3,6-dihydro-2H-1,2-oxaborinin-2-ol (2h’)

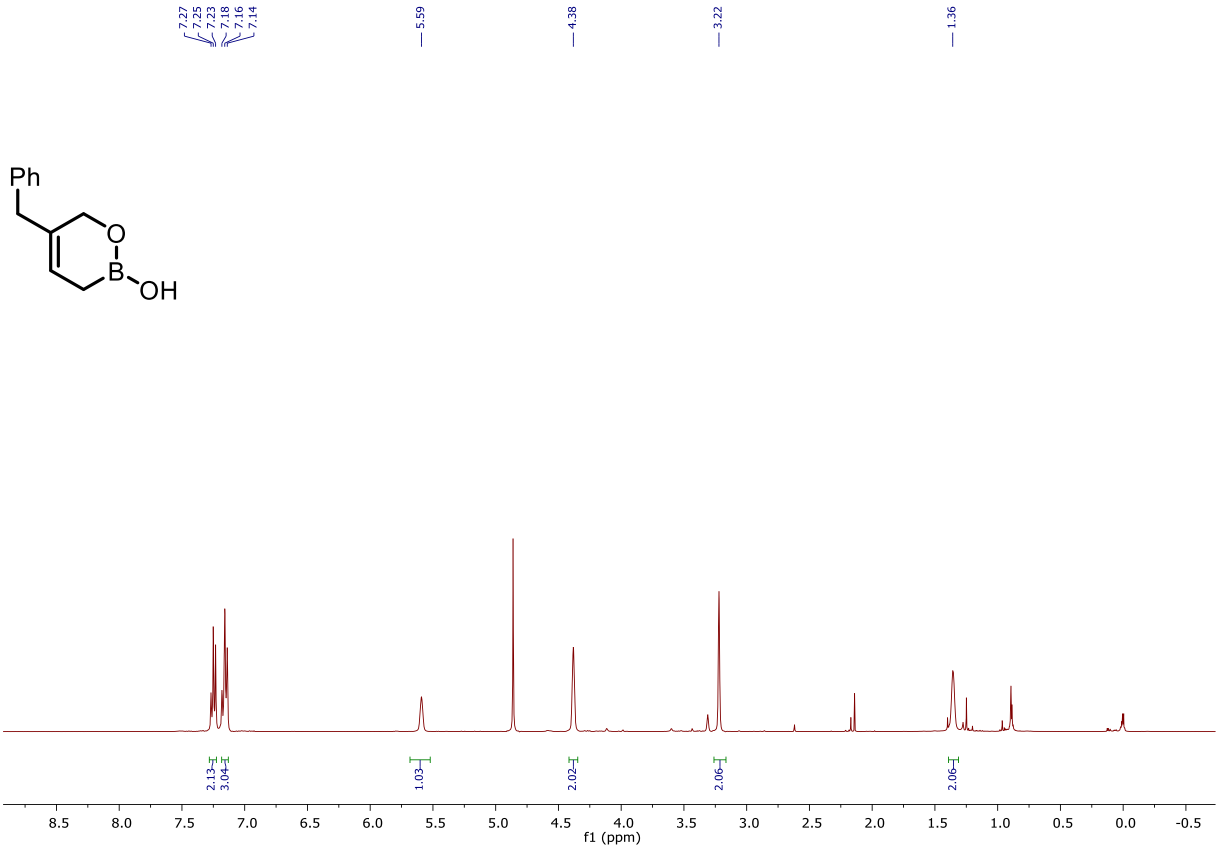

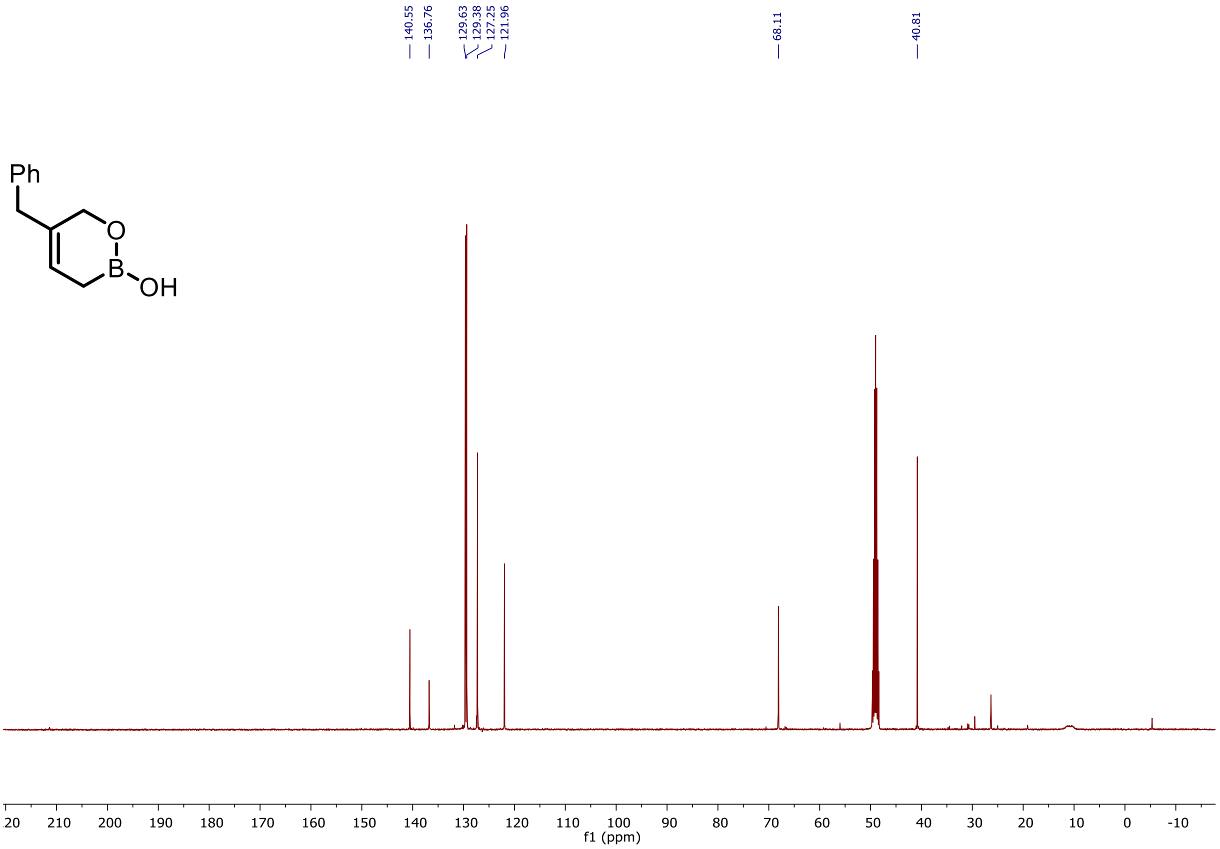

Dimethyl-(1-phenyl-2-(4,4,5,5-tetramethyl-1,3,2-dioxaborolan-2-yl)ethylidene) heptanedioate (2i)

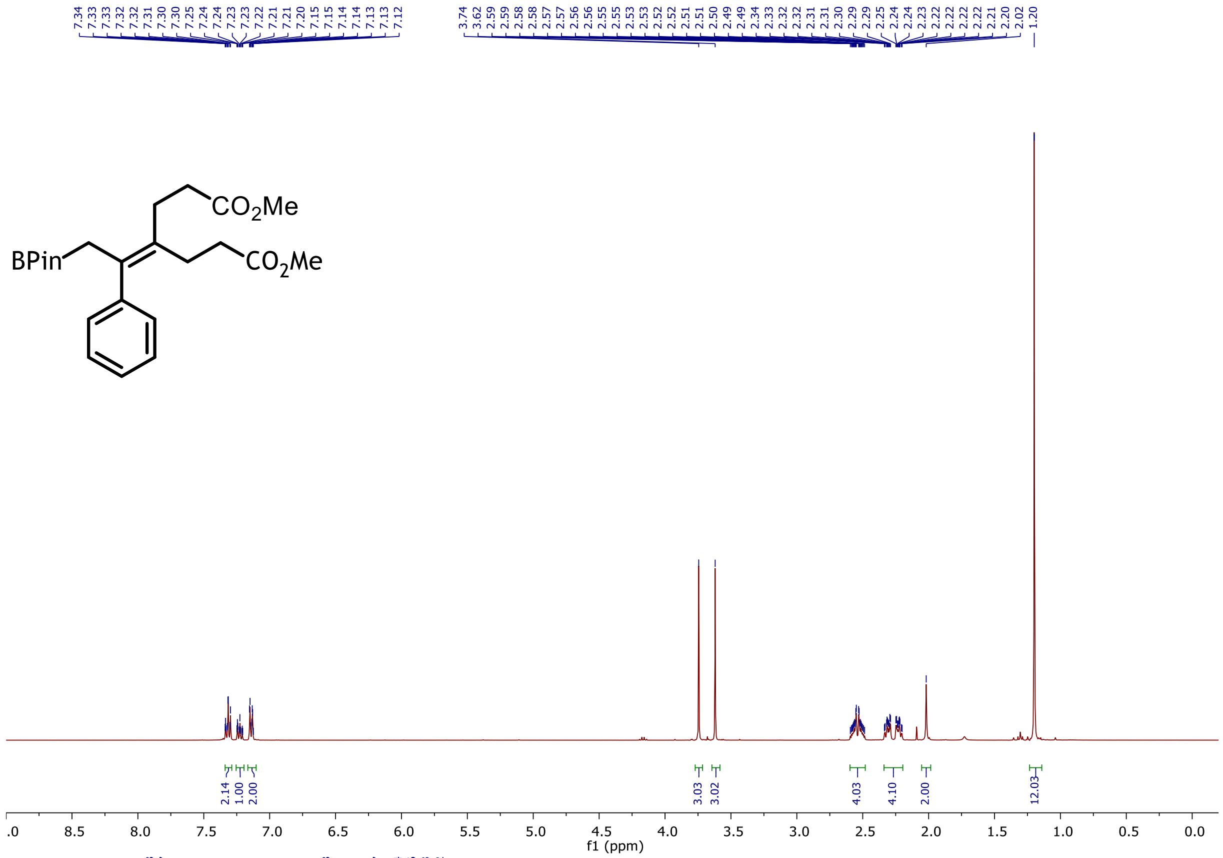

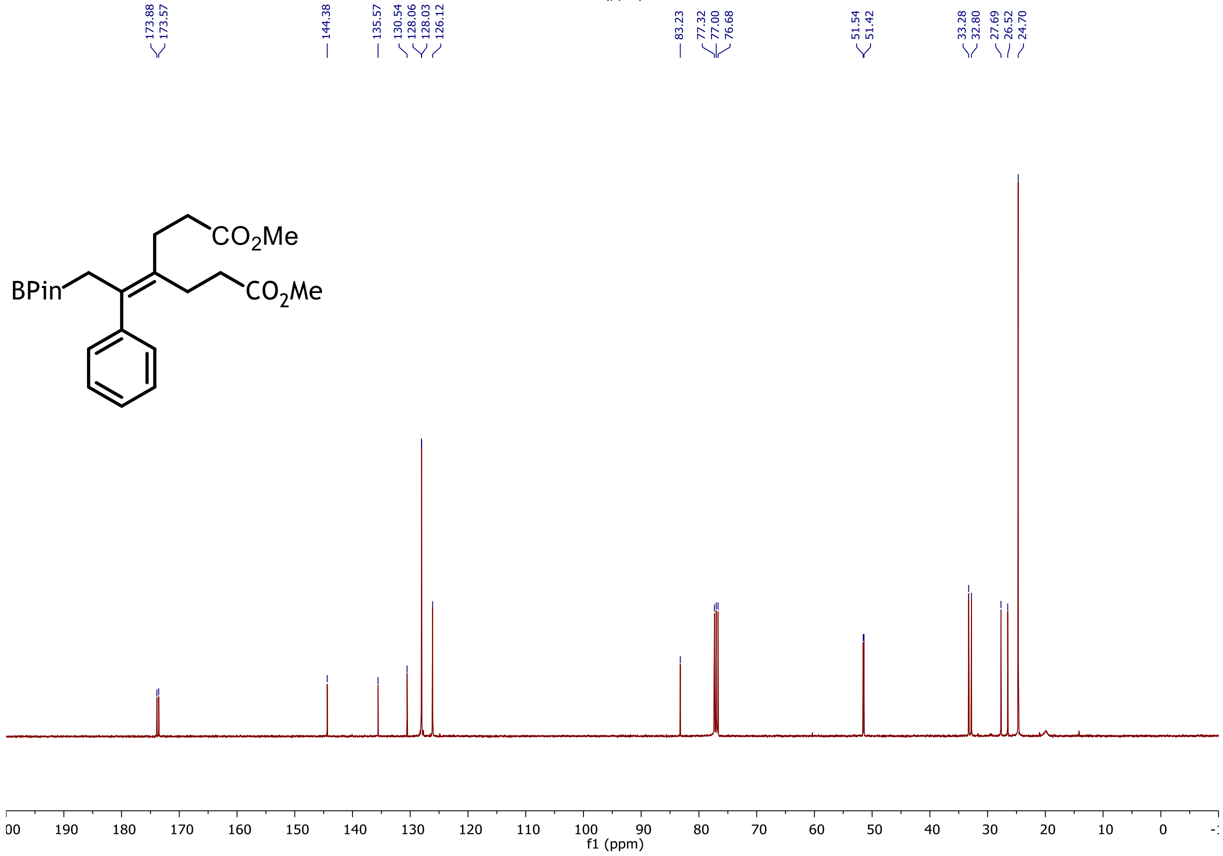

Dimethyl-4-(1-(4-chlorophenyl)-2-(4,4,5,5-tetramethyl-1,3,2-dioxaborolan-2-yl)ethylidene) heptanedioate (2j)

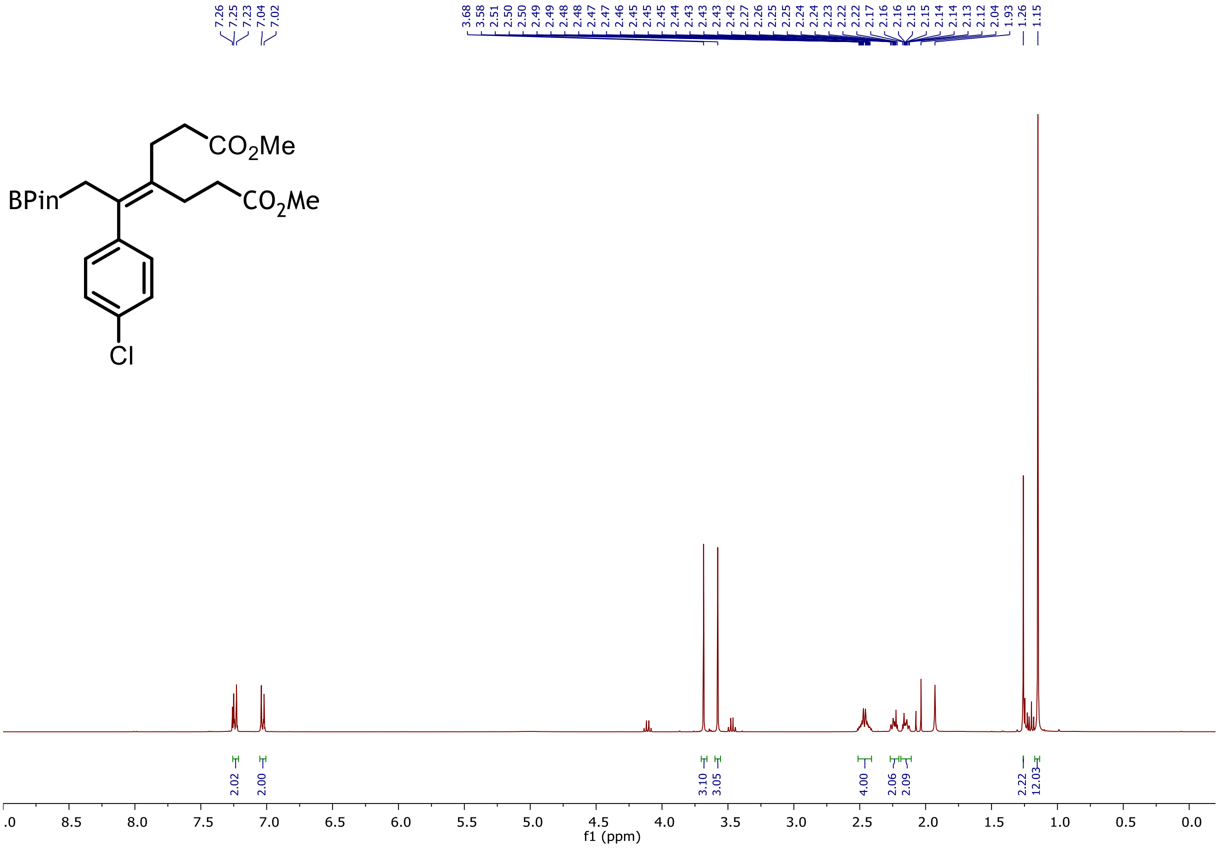

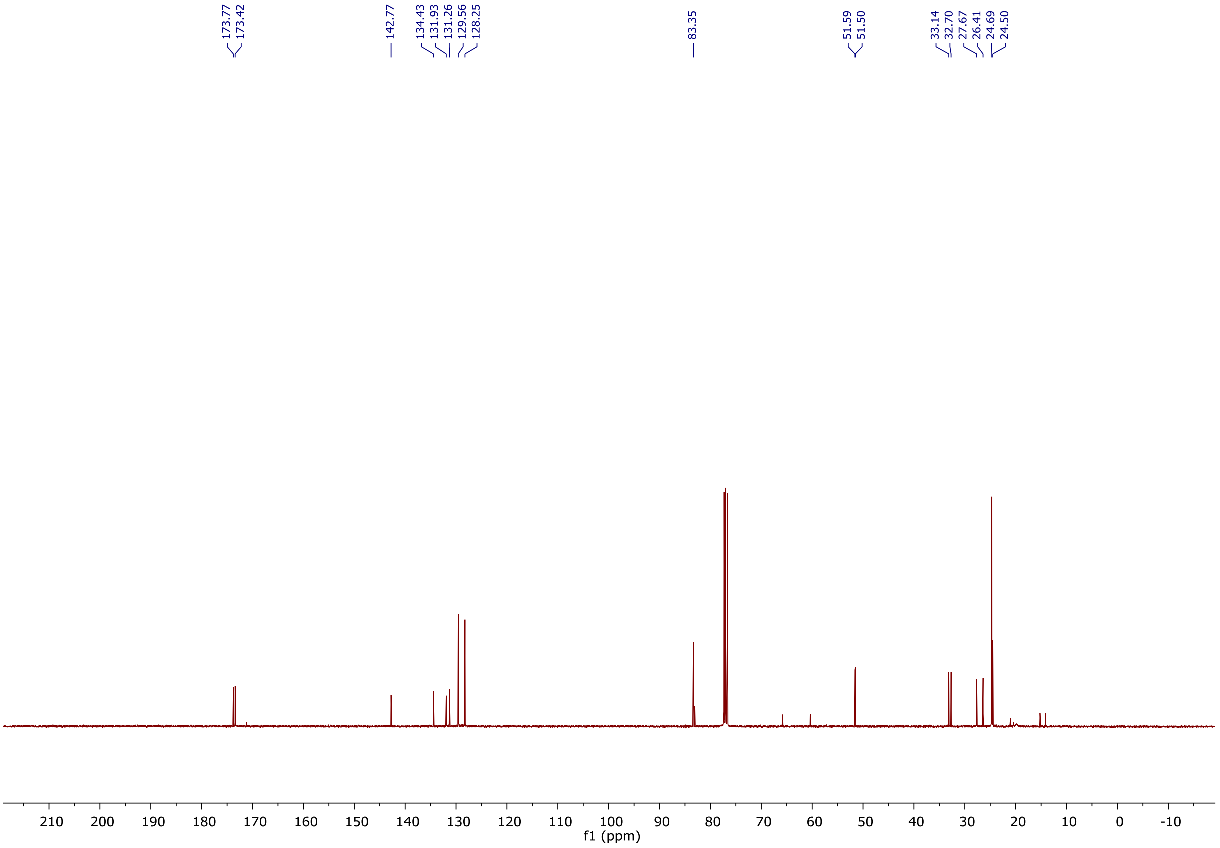

Dimethyl4-(1-(2-fluorophenyl)-2-(4,4,5,5-tetramethyl-1,3,2-dioxaborolan-2-yl)ethylidene) heptanedioate (2k)

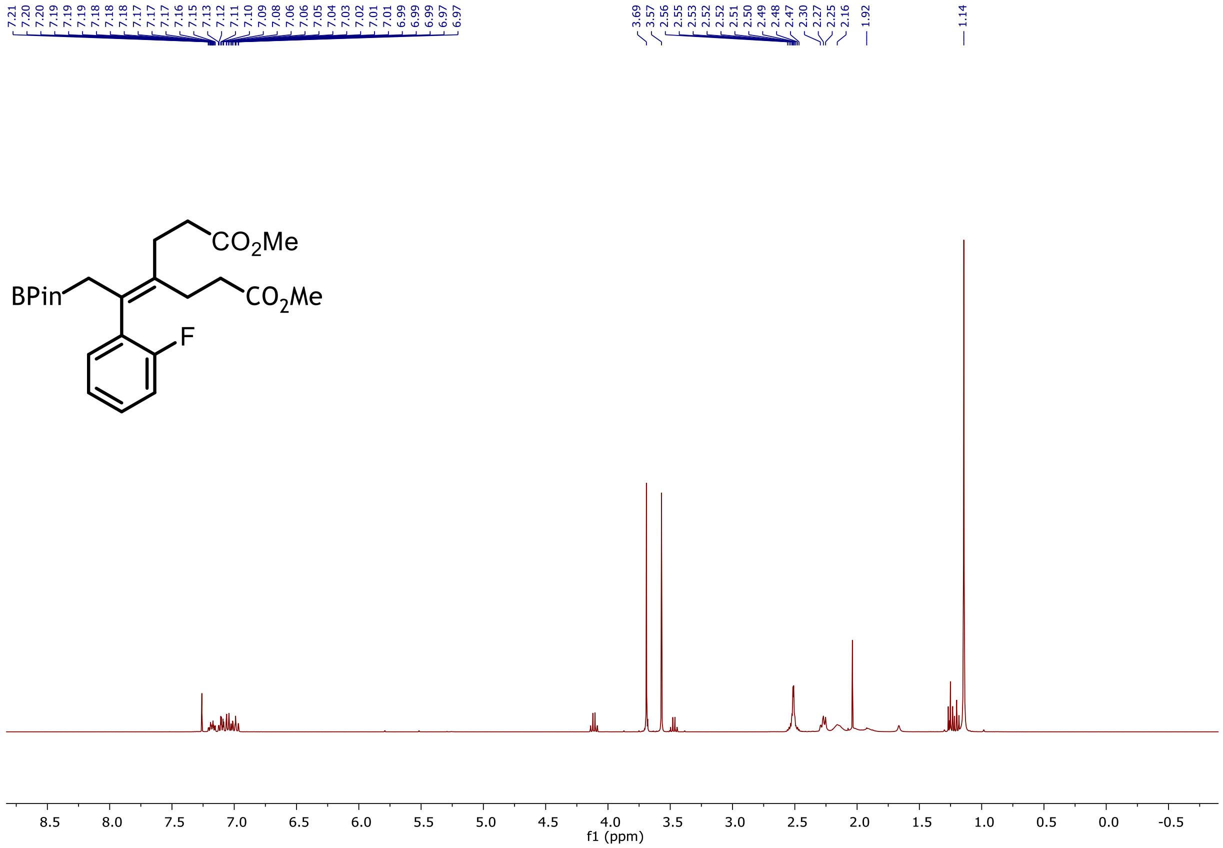

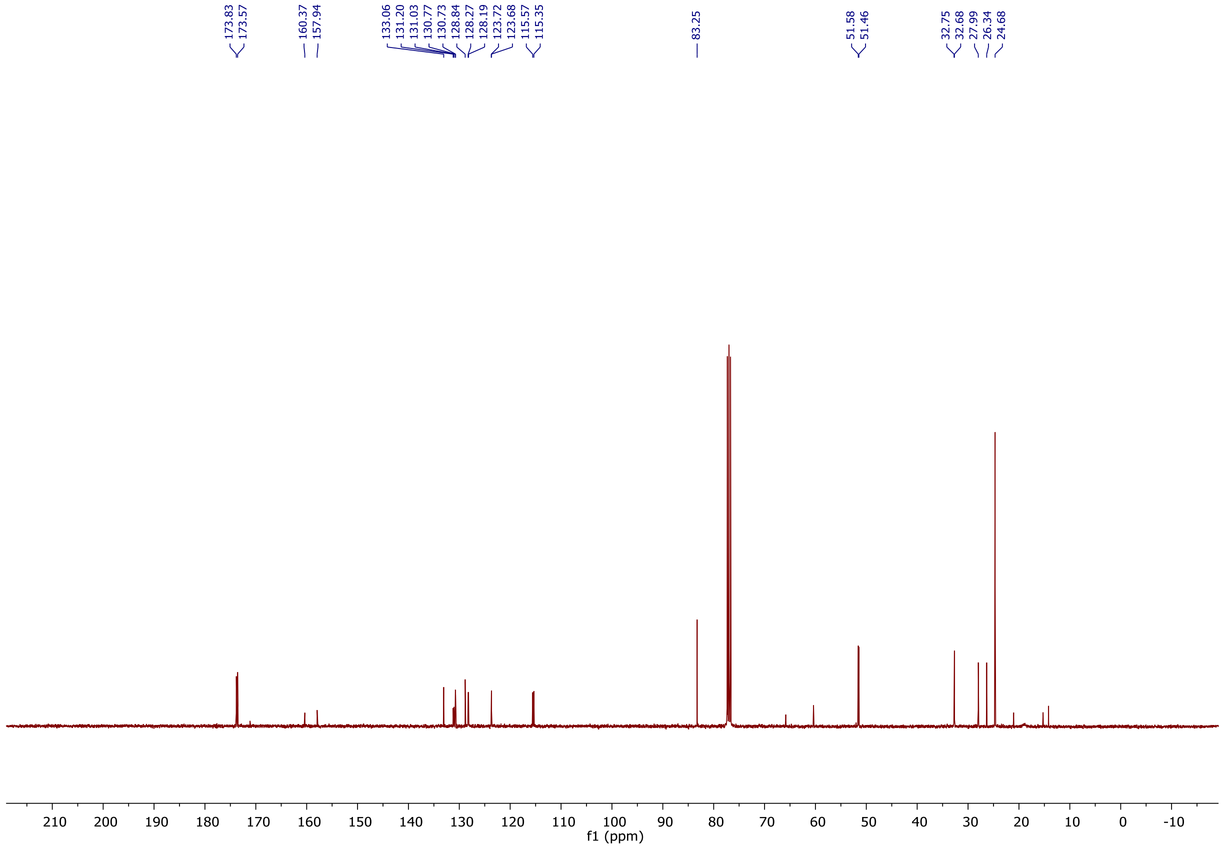

4-(1-Phenyl-2-(4,4,5,5-tetramethyl-1,3,2-dioxaborolan-2yl)ethylidene)heptanedinitrile (2l)

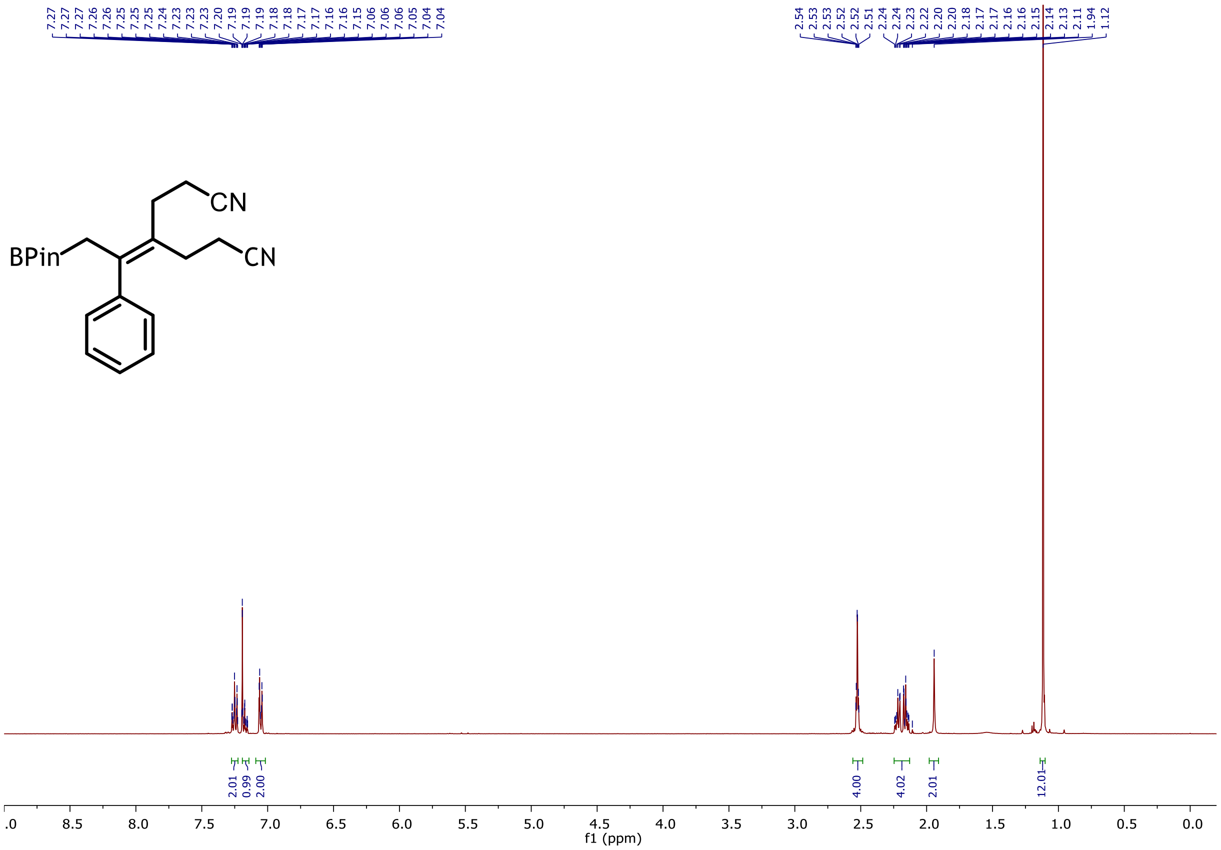

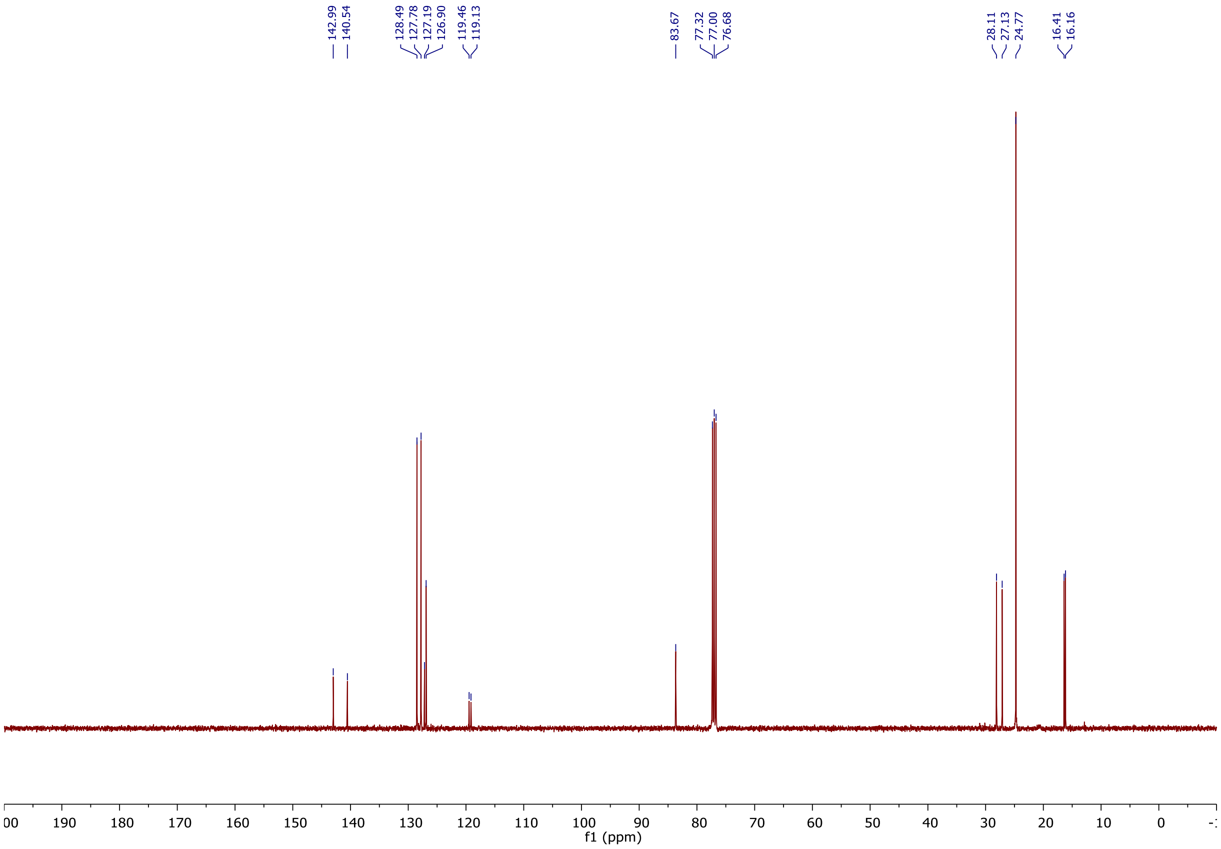

(*Z*)-4-Methyl-5-phenyl-6-(4,4,5,5-tetramethyl-1,3,2-dioxaborolan-2-yl)hex-4-enenitrile (2m)

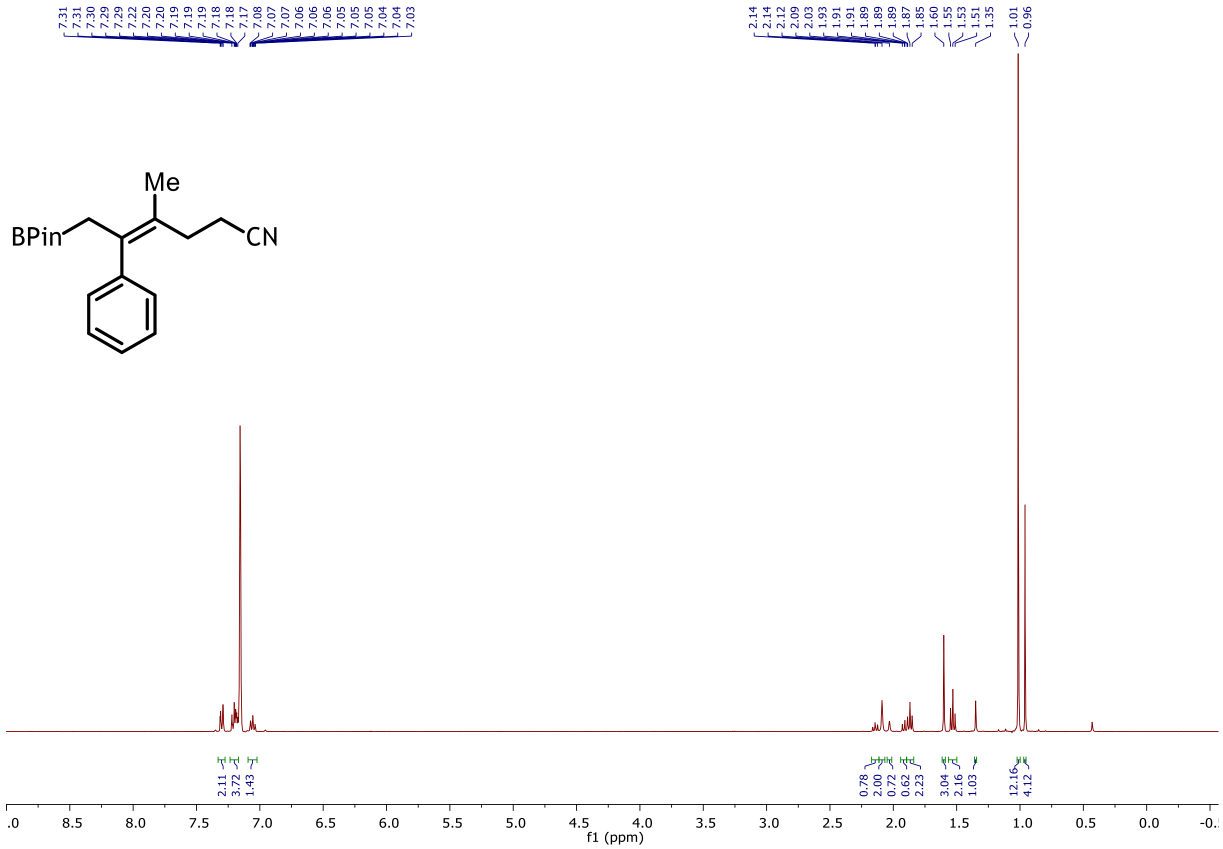

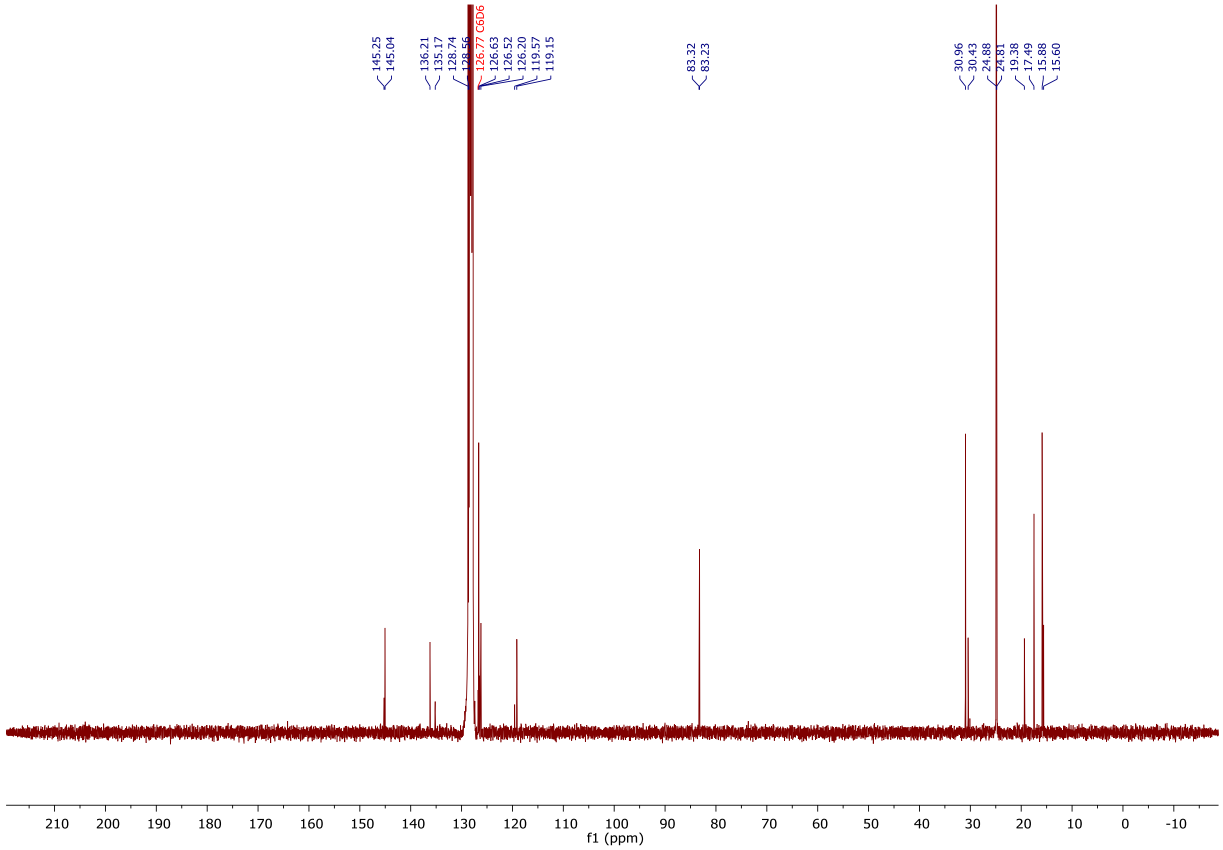

Methyl (Z)-4-fluoro-6-(4,4,5,5-tetramethyl-1,3,2-dioxaborolan-2-yl)hex-4-enoate (2n)

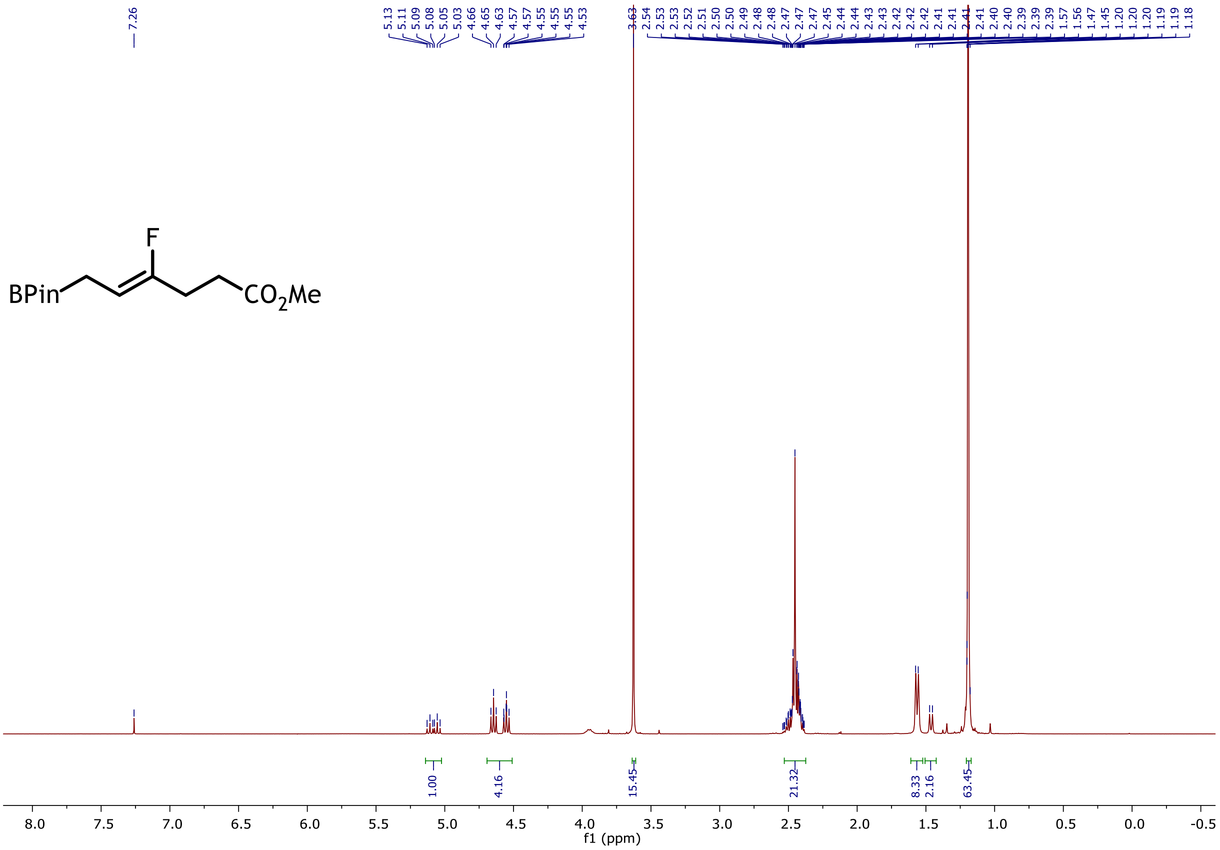

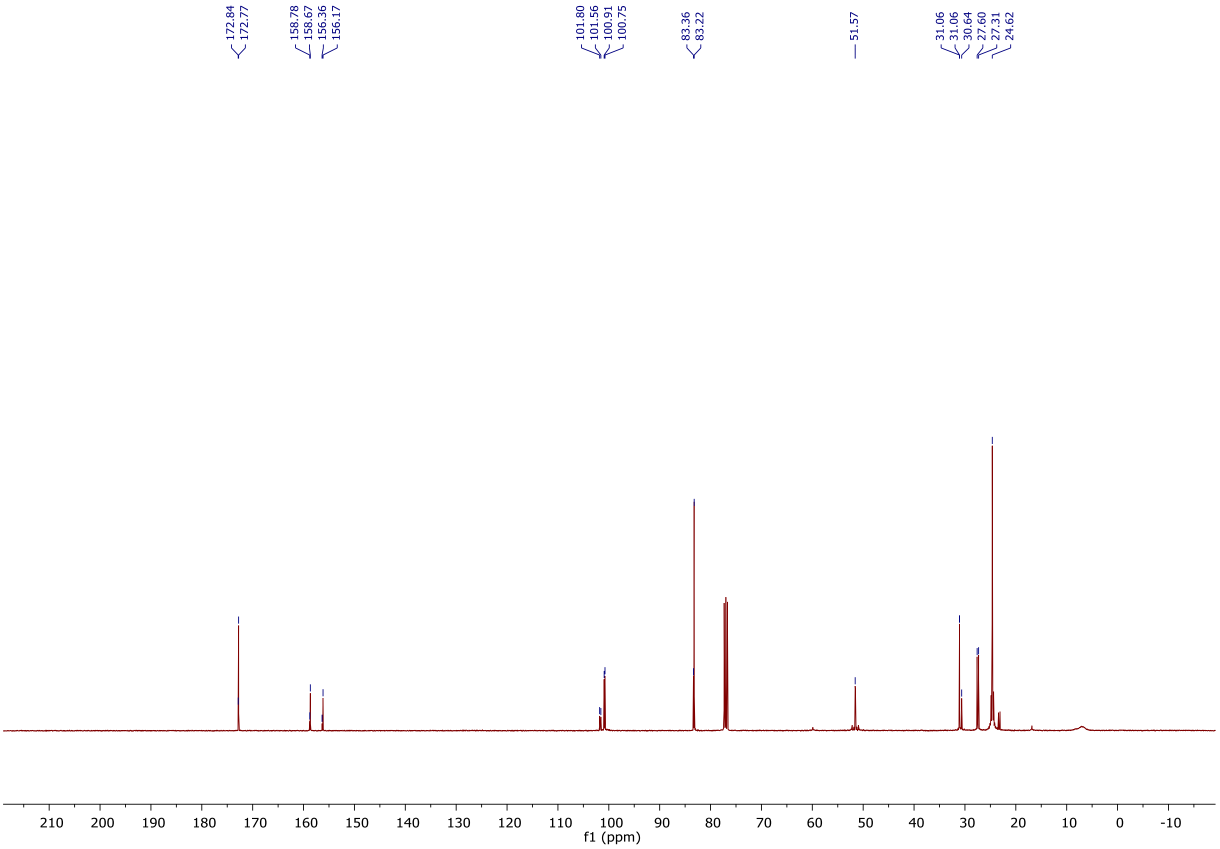

(*Z*)-2-(3-Fluoro-4-phenylbut-2-en-1-yl)-4,4,5,5-tetramethyl-1,3,2-dioxaborolane (2o)

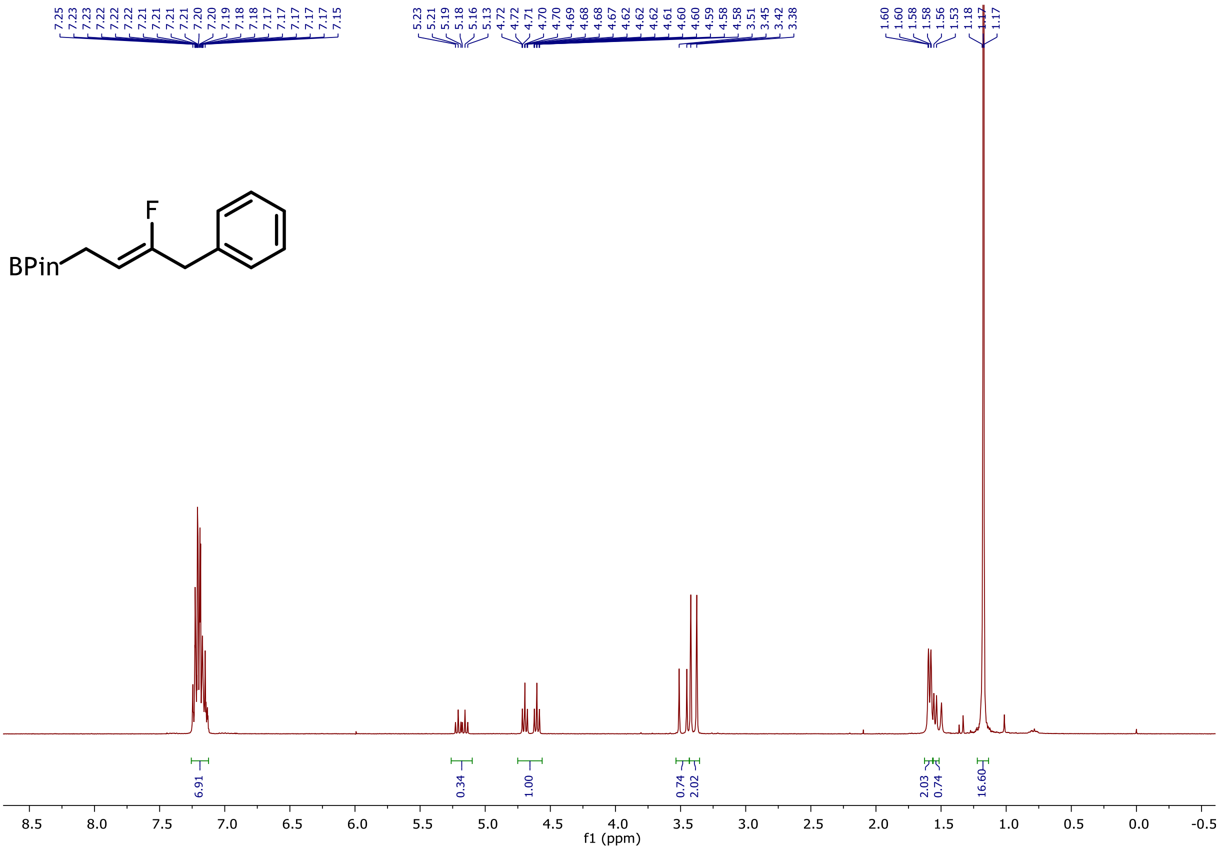

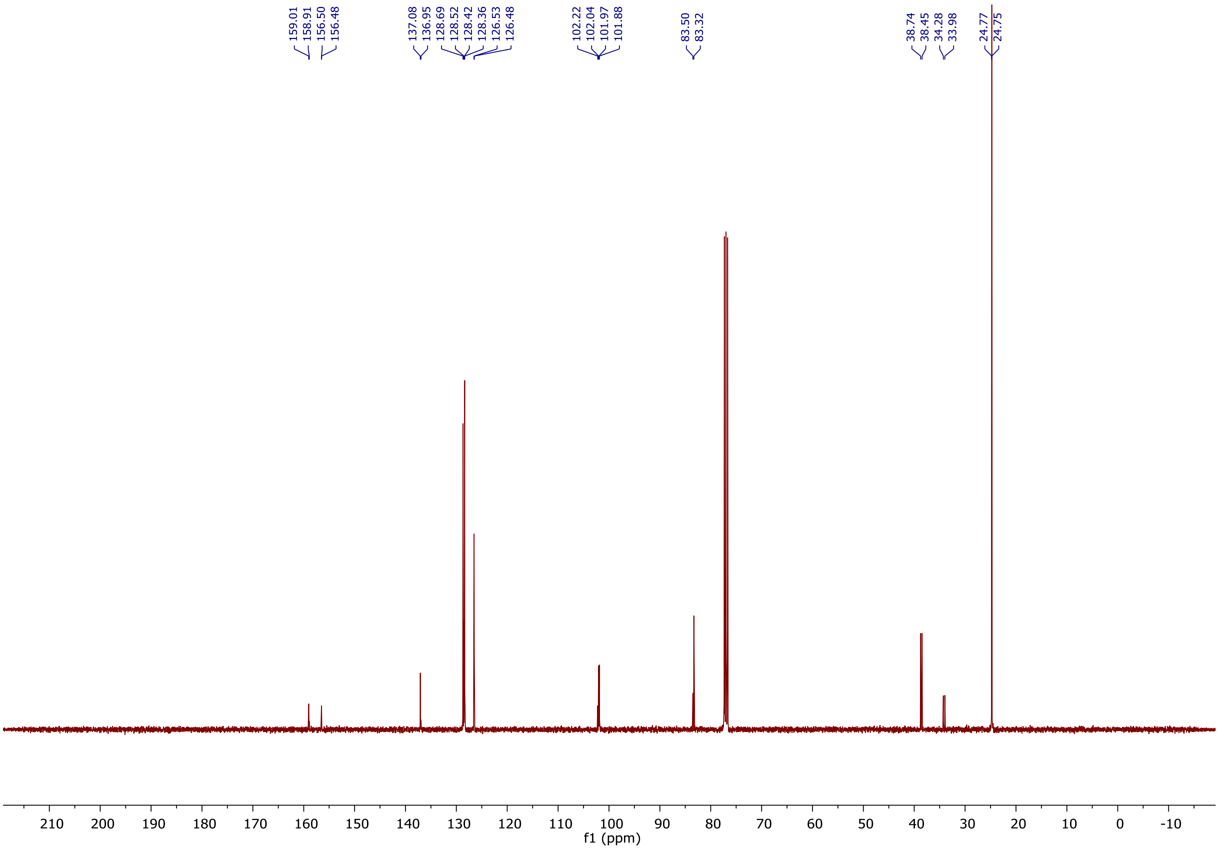

(*Z*)-2-(4-(4-Chlorophenyl)-3-fluorobut-2-en-1-yl)-4,4,5,5-tetramethyl-1,3,2-dioxa-borolane (2p)

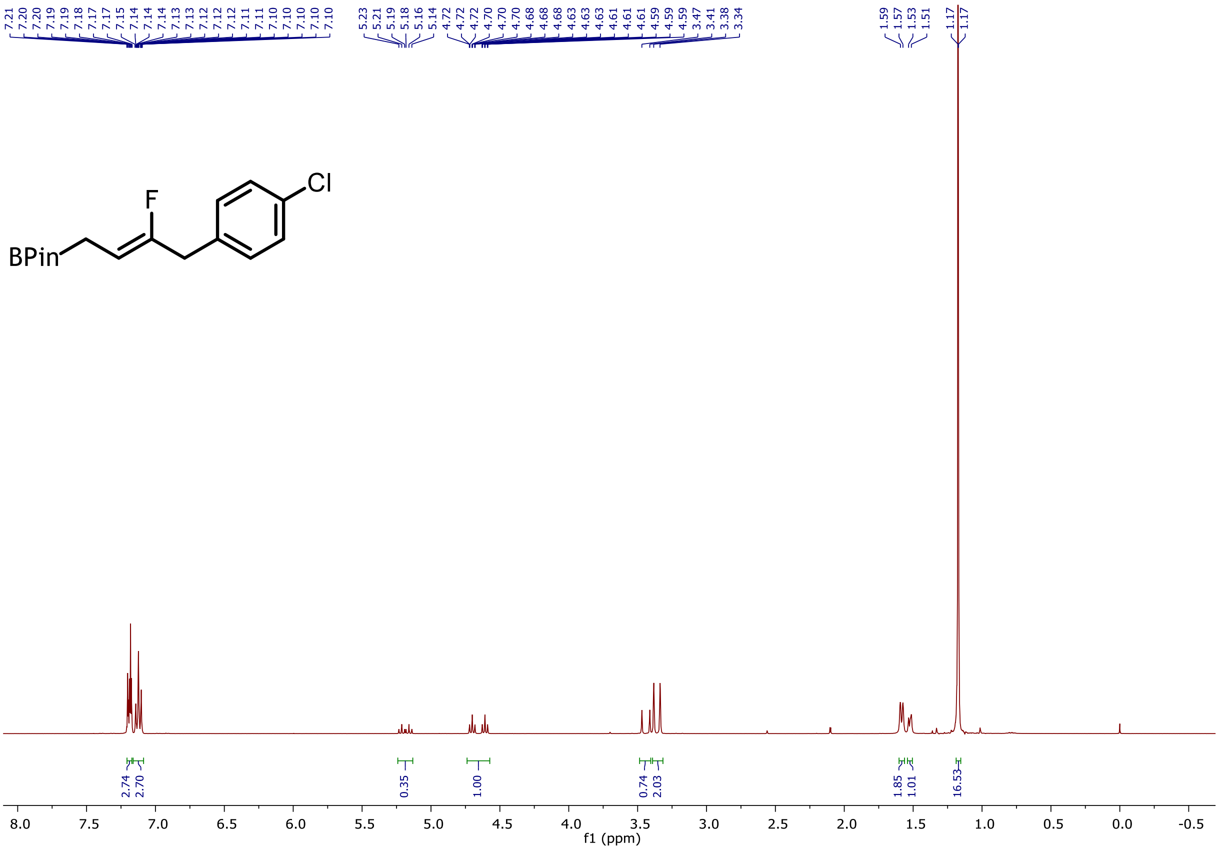

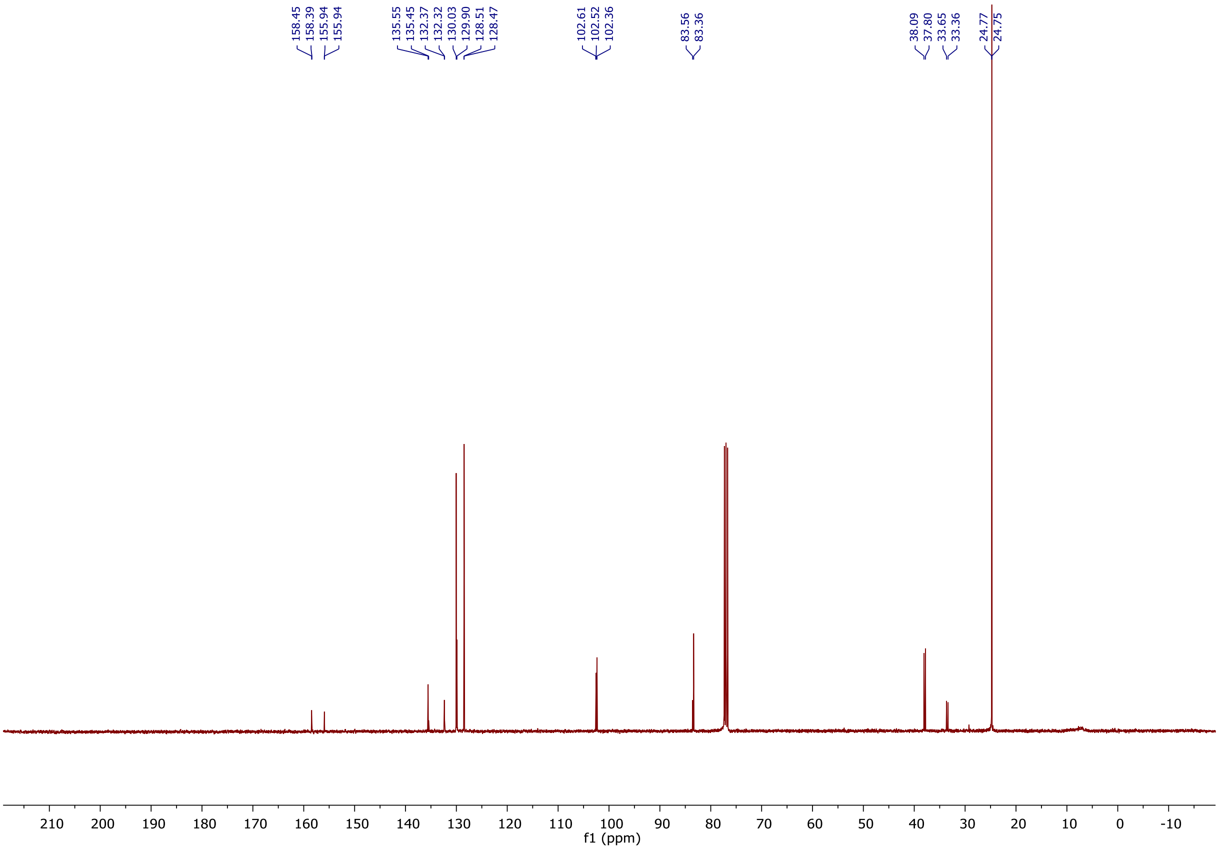

(*Z*)-4-(2-Fluoro-4-(4,4,5,5-tetramethyl-1,3,2-dioxaborolan-2-yl)but-2-en-1-yl) benzonitrile (2q)

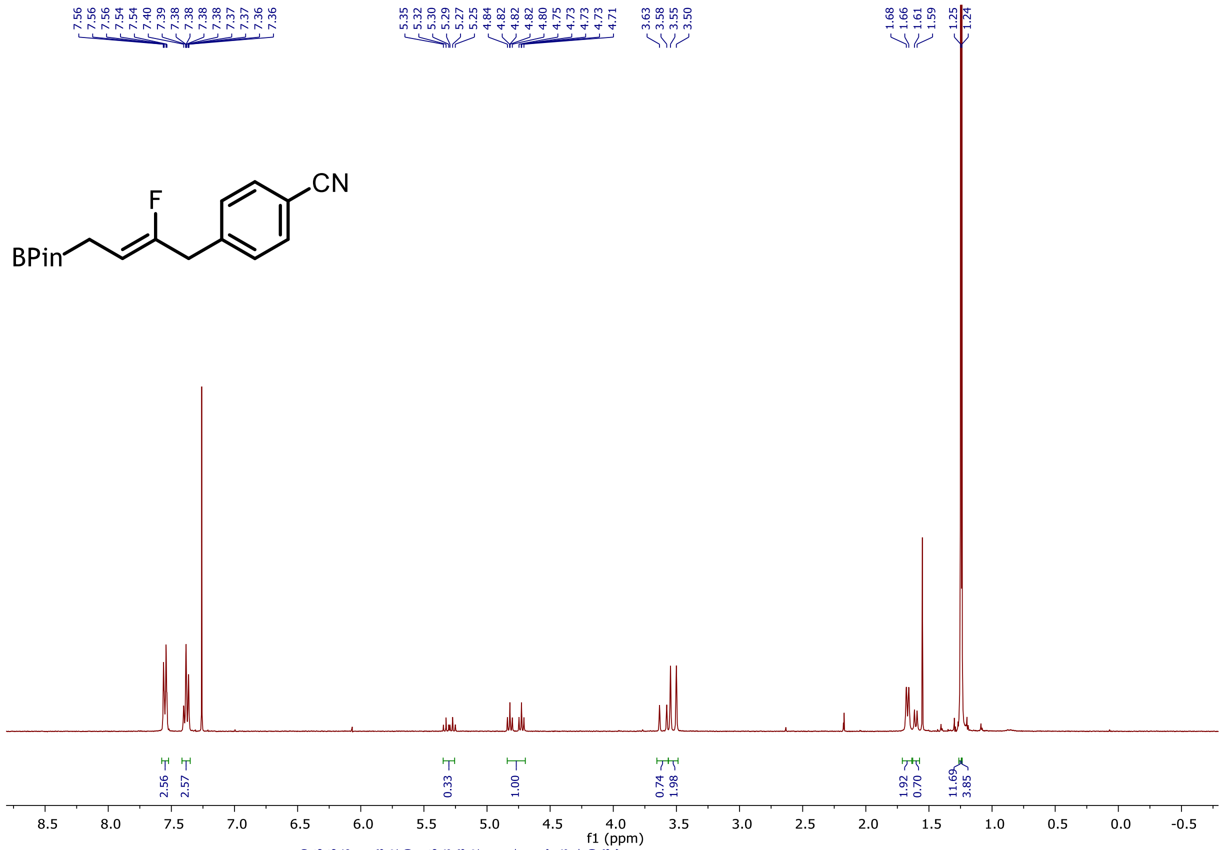

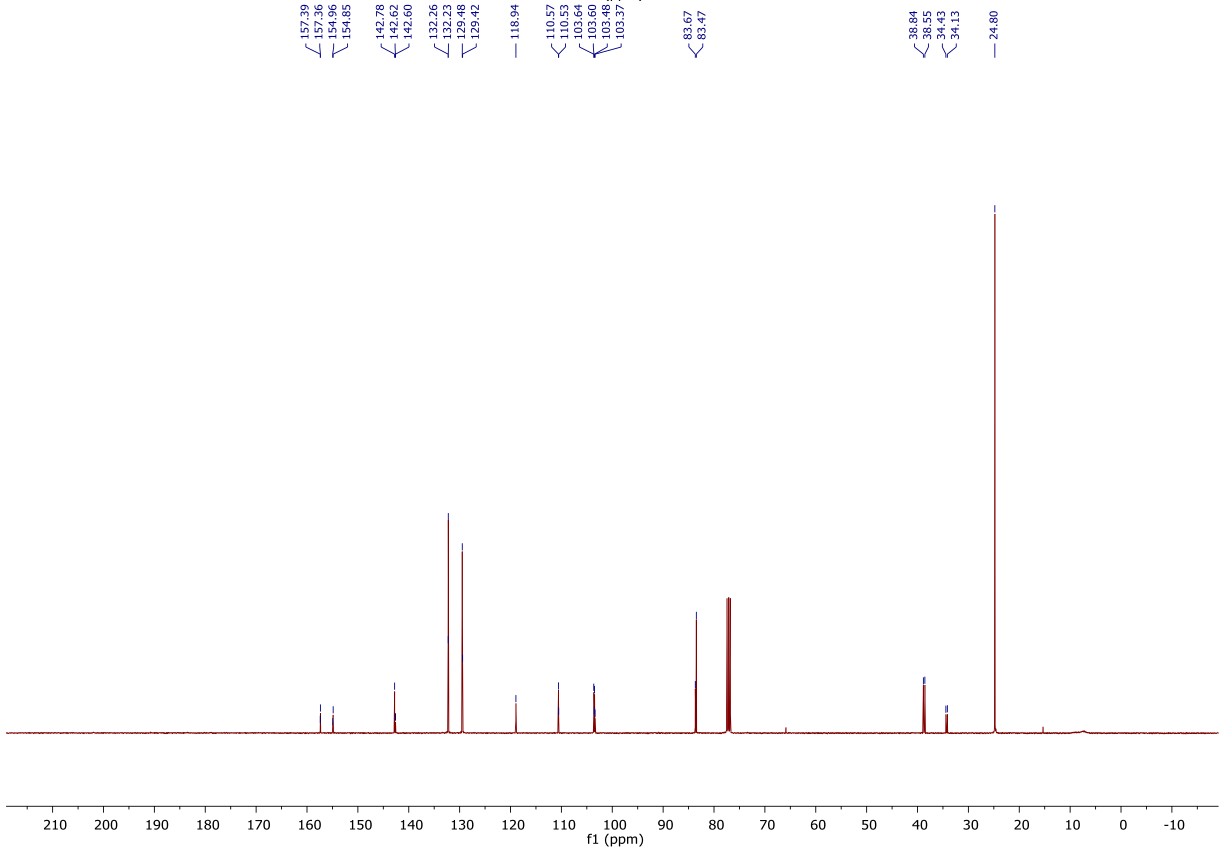

(*Z*)-2-(3-Fluoro-4-(4-(trifluoromethyl)phenyl)but-2-en-1-yl)-4,4,5,5-tetramethyl-1,3,2-dioxaborolane (2r)

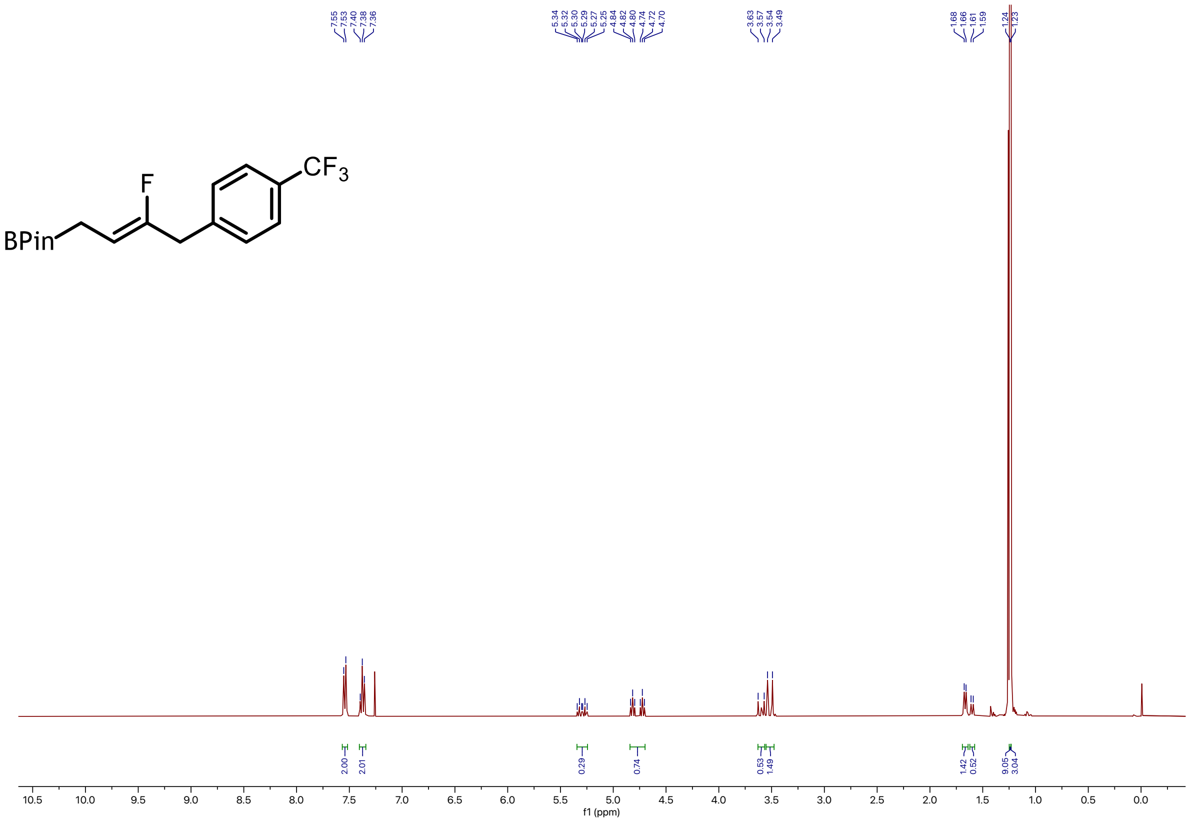

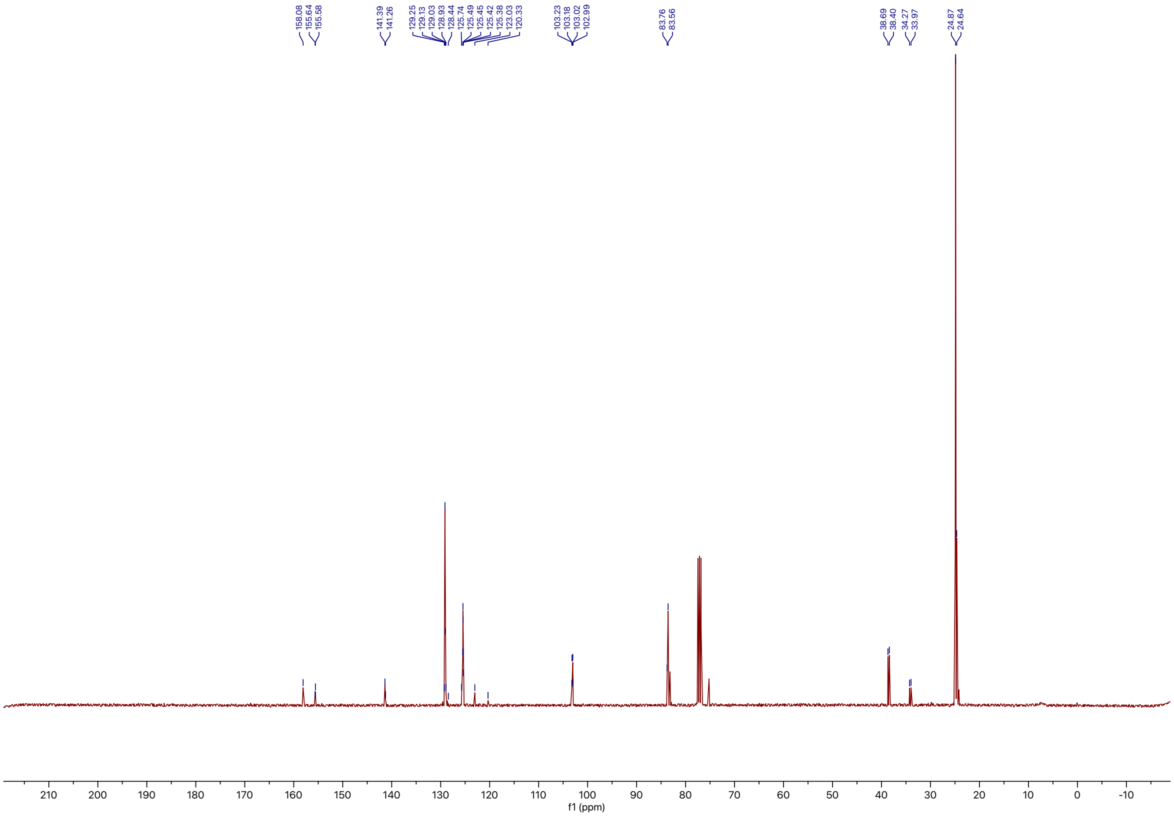

(*Z*)-2-(3-Fluoro-4-(4-methoxyphenyl)but-2-en-1-yl)-4,4,5,5-tetramethyl-1,3,2-dioxa-borolane (2s)

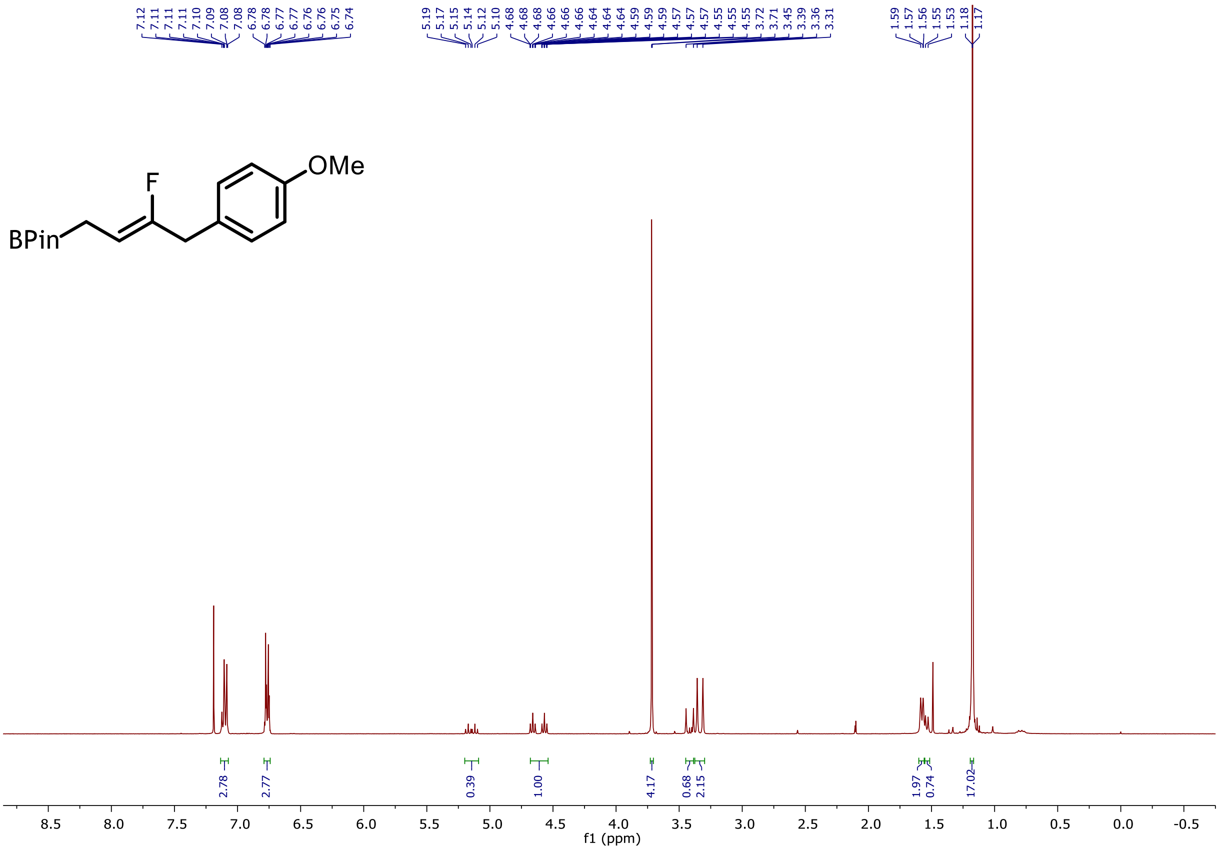

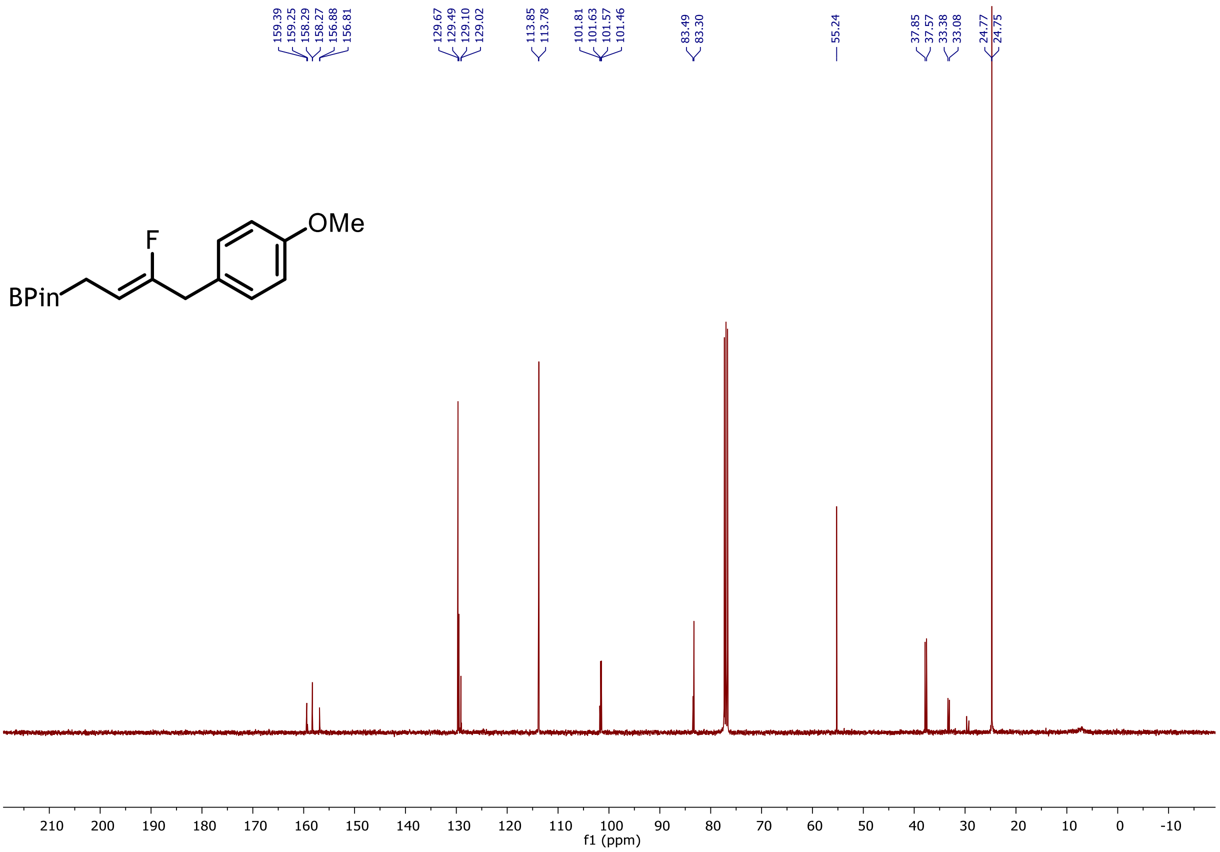

(*Z*)-2-(3-Fluoro-4-(3-methoxyphenyl)but-2-en-1-yl)-4,4,5,5-tetramethyl-1,3,2-dioxa-borolane (2t)

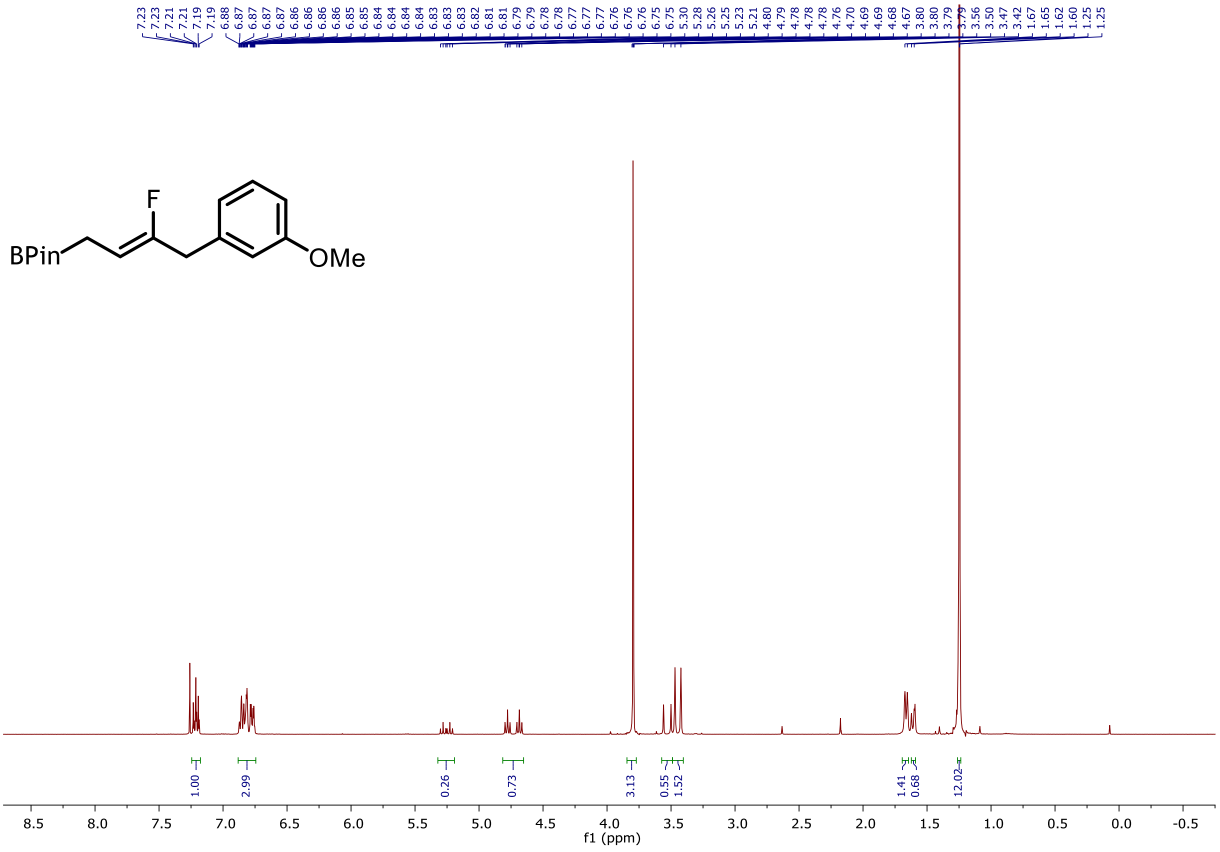

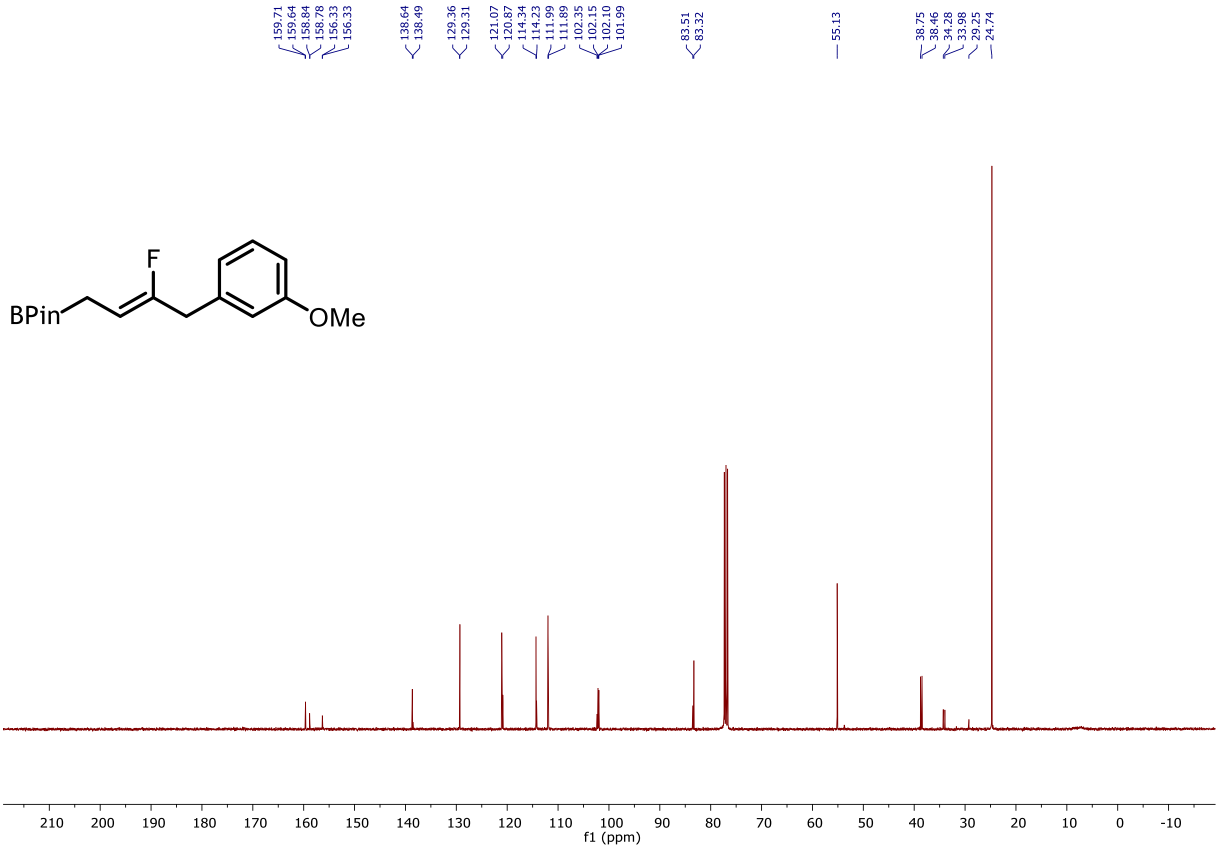

(*Z*)-2-(3-Fluoro-4-(2-methoxyphenyl)but-2-en-1-yl)-4,4,5,5-tetramethyl-1,3,2-dioxa-borolane (2u)

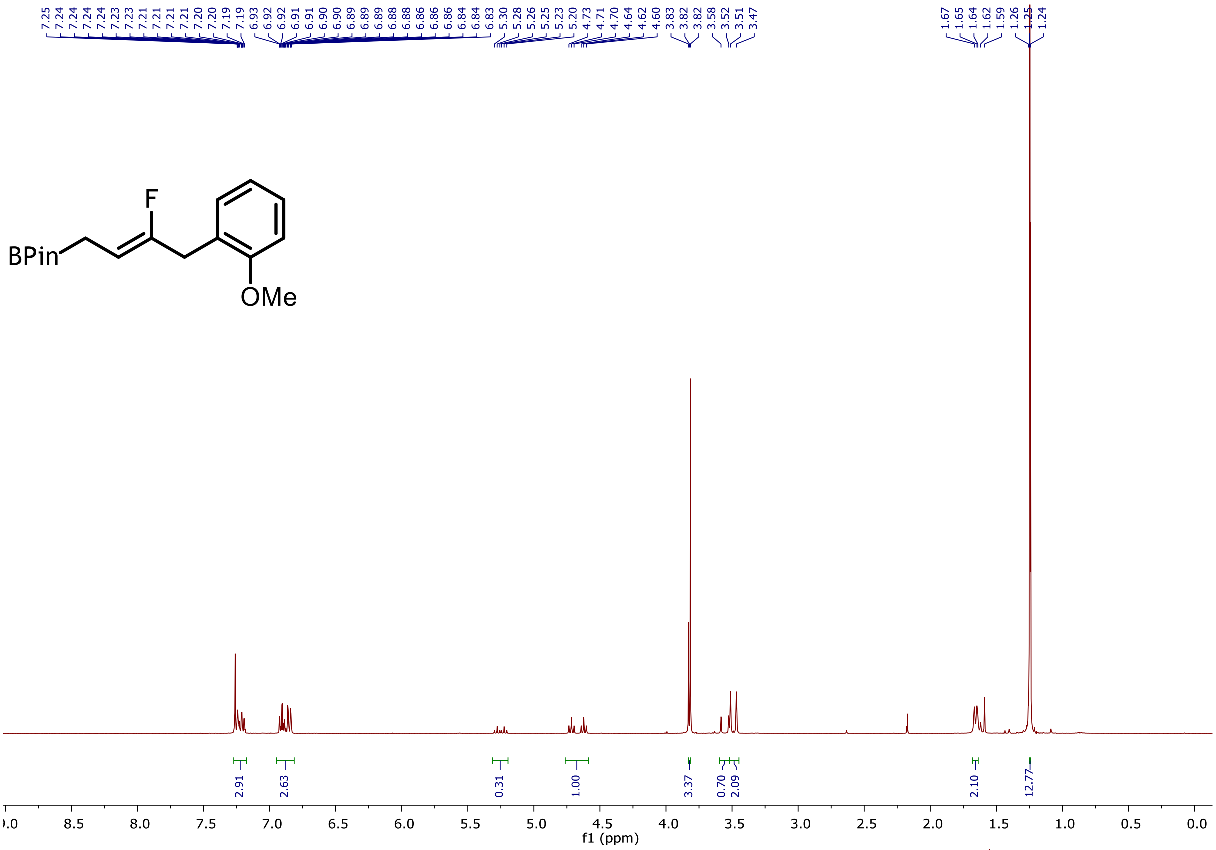

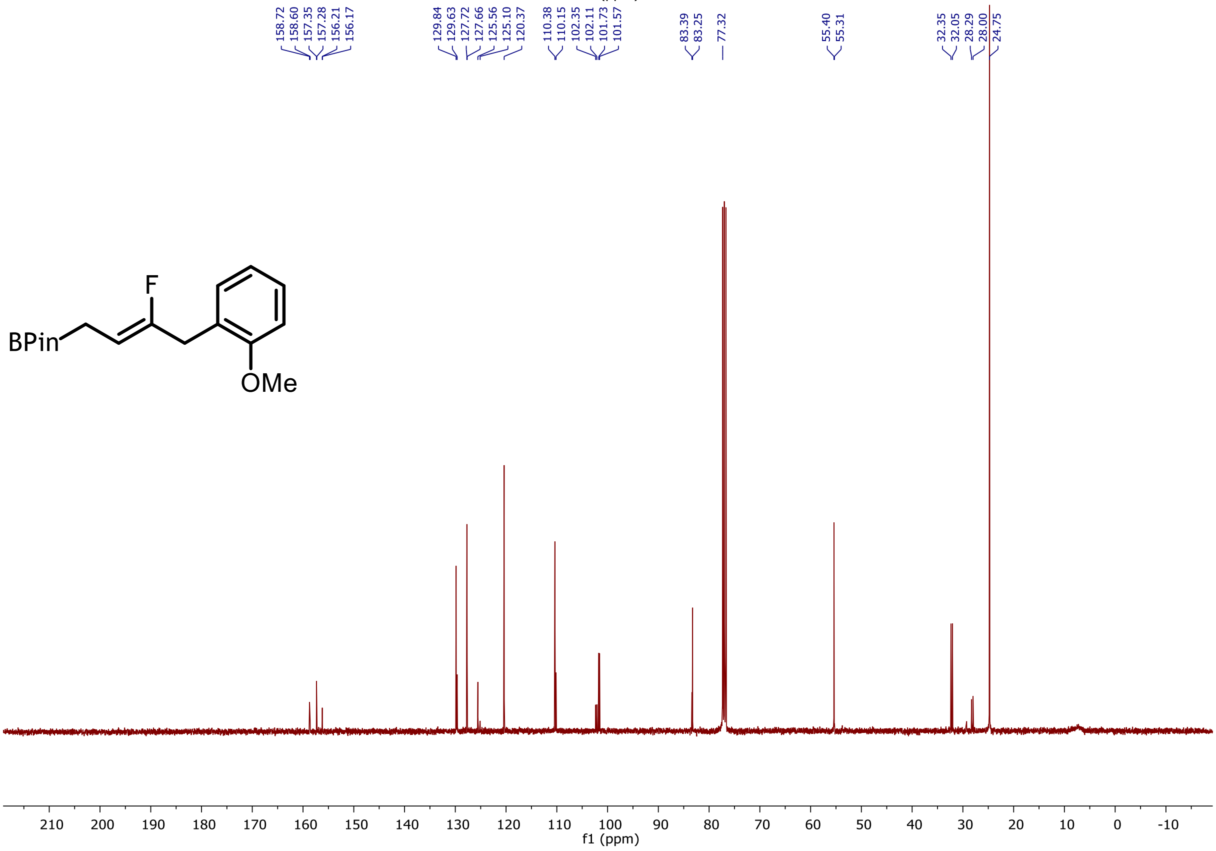

(*Z*)-2-(3-Fluoro-4-(furan-2-yl)but-2-en-1-yl)-4,4,5,5-tetramethyl-1,3,2-dioxaborolane (2v)

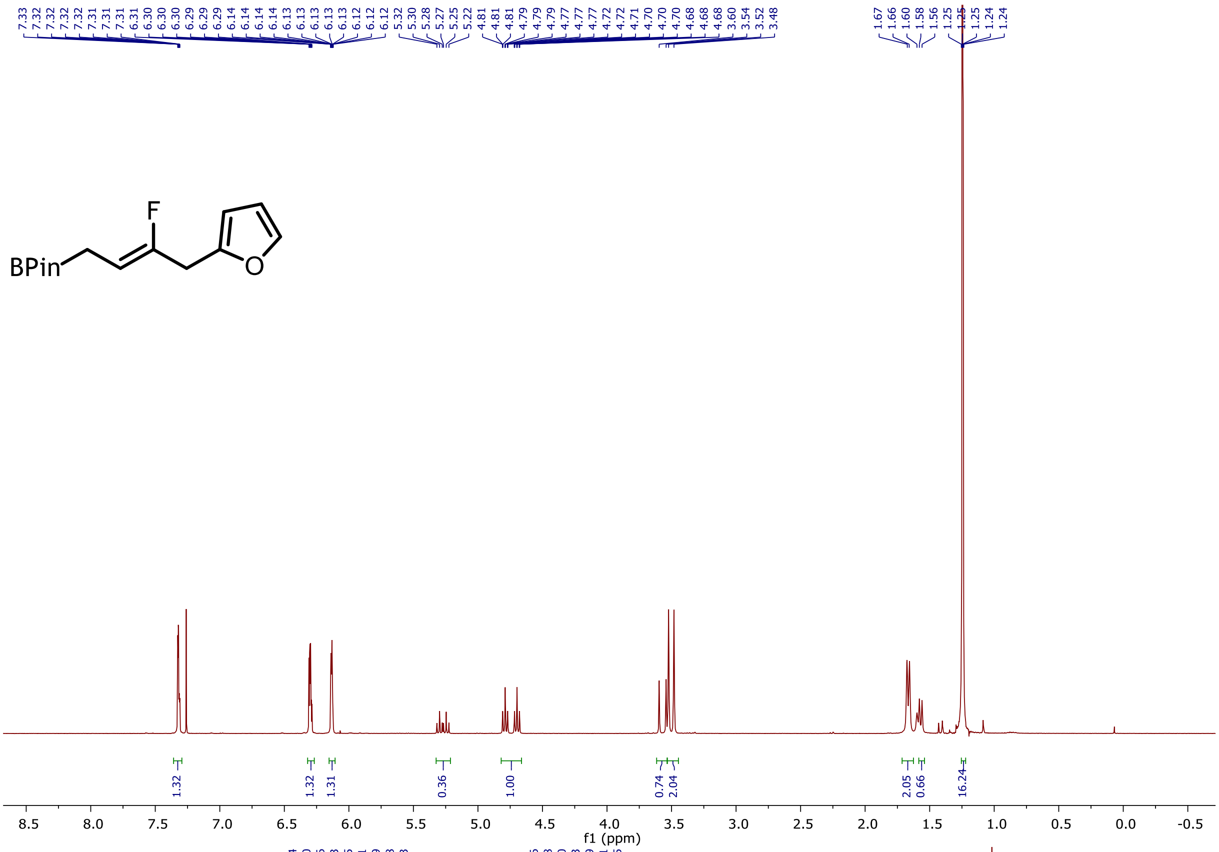

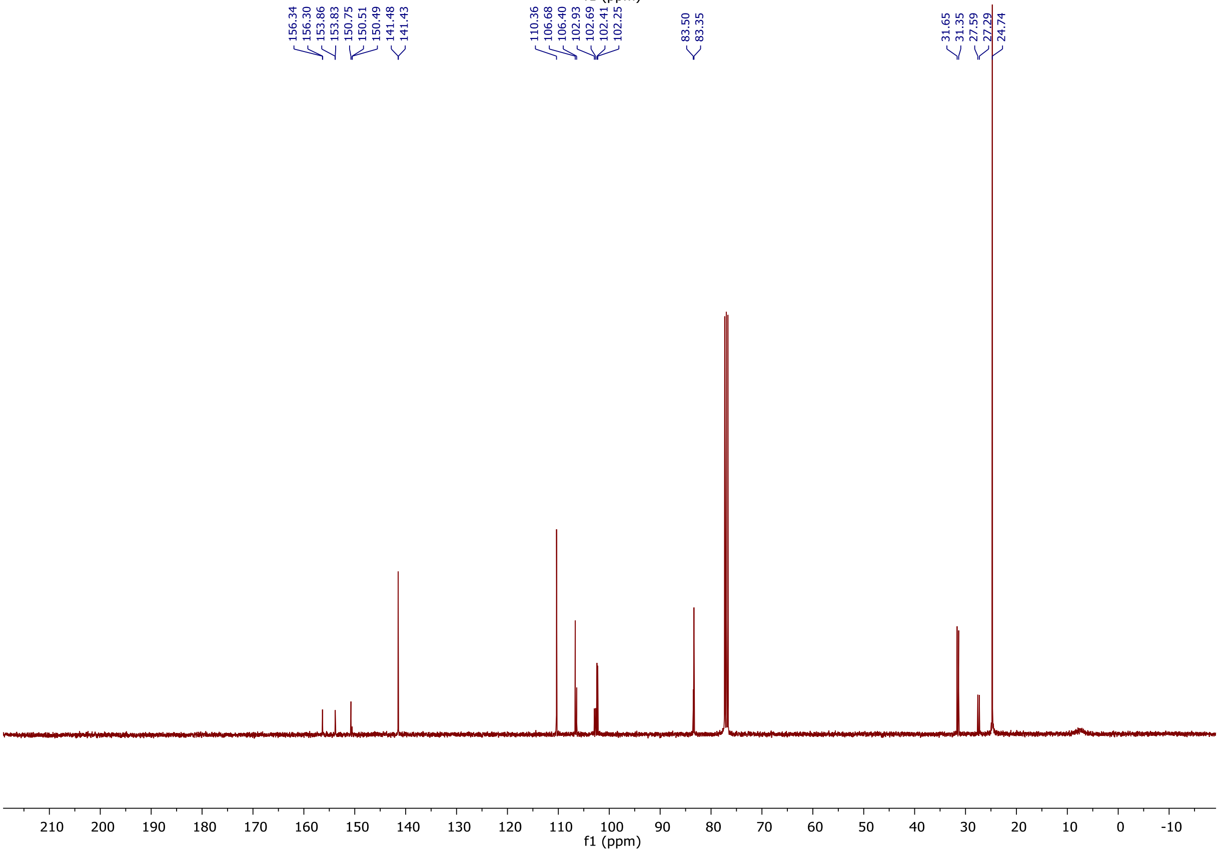

(*E*)-2-(3-Fluoro-2-phenylbut-2-en-1-yl)-4,4,5,5-tetramethyl-1,3,2-dioxaborolane (2w)

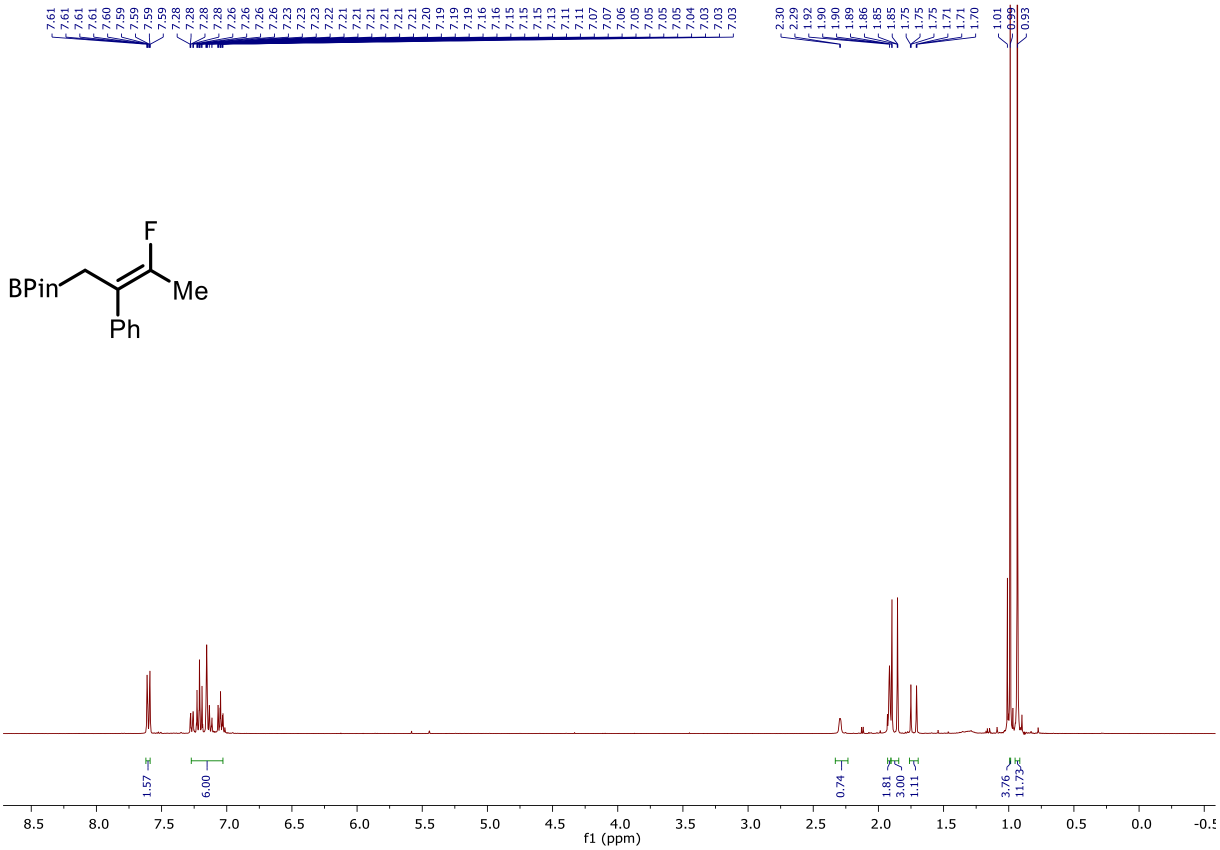

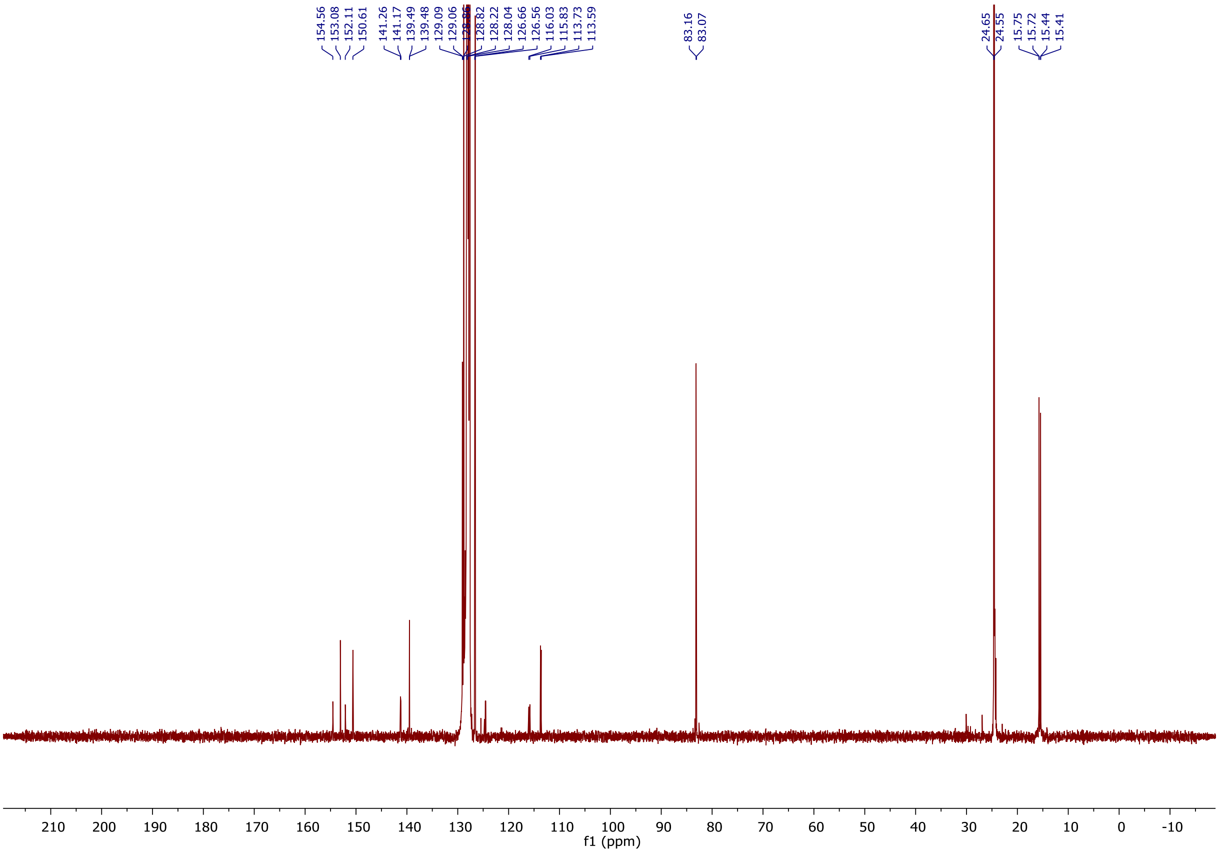

(*Z*)-2-(3-Fluoro-4-(naphthalen-1-yl)but-2-en-1-yl)-4,4,5,5-tetramethyl-1,3,2-dioxa-borolane (2x)

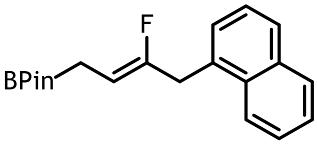

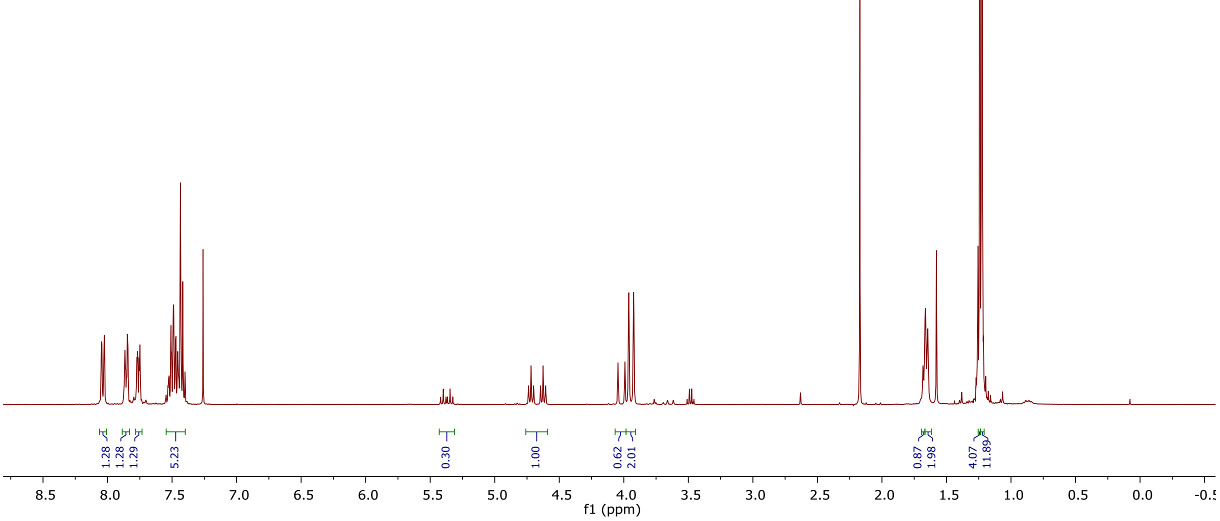

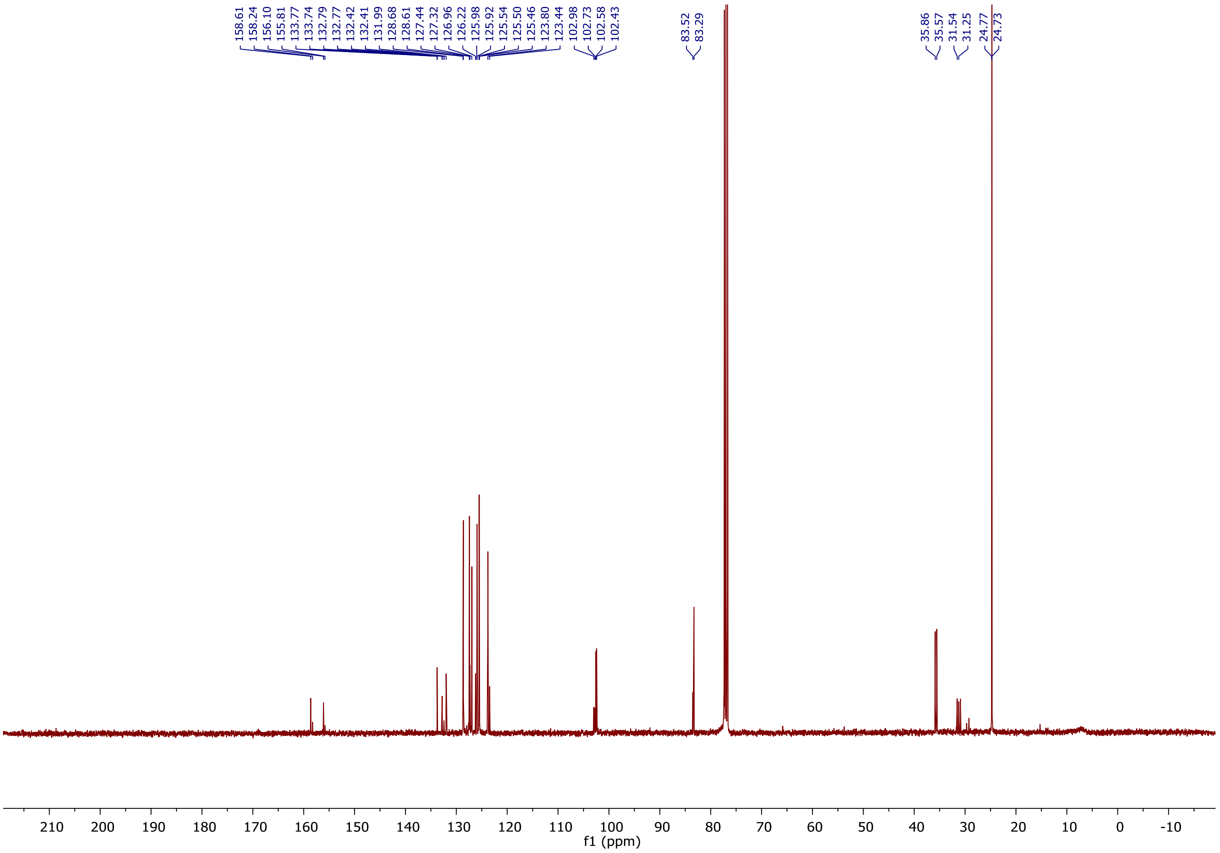

Dimethyl (*E*)-4-nitro-4-(prop-1-en-1-yl)heptanedioate (3a)

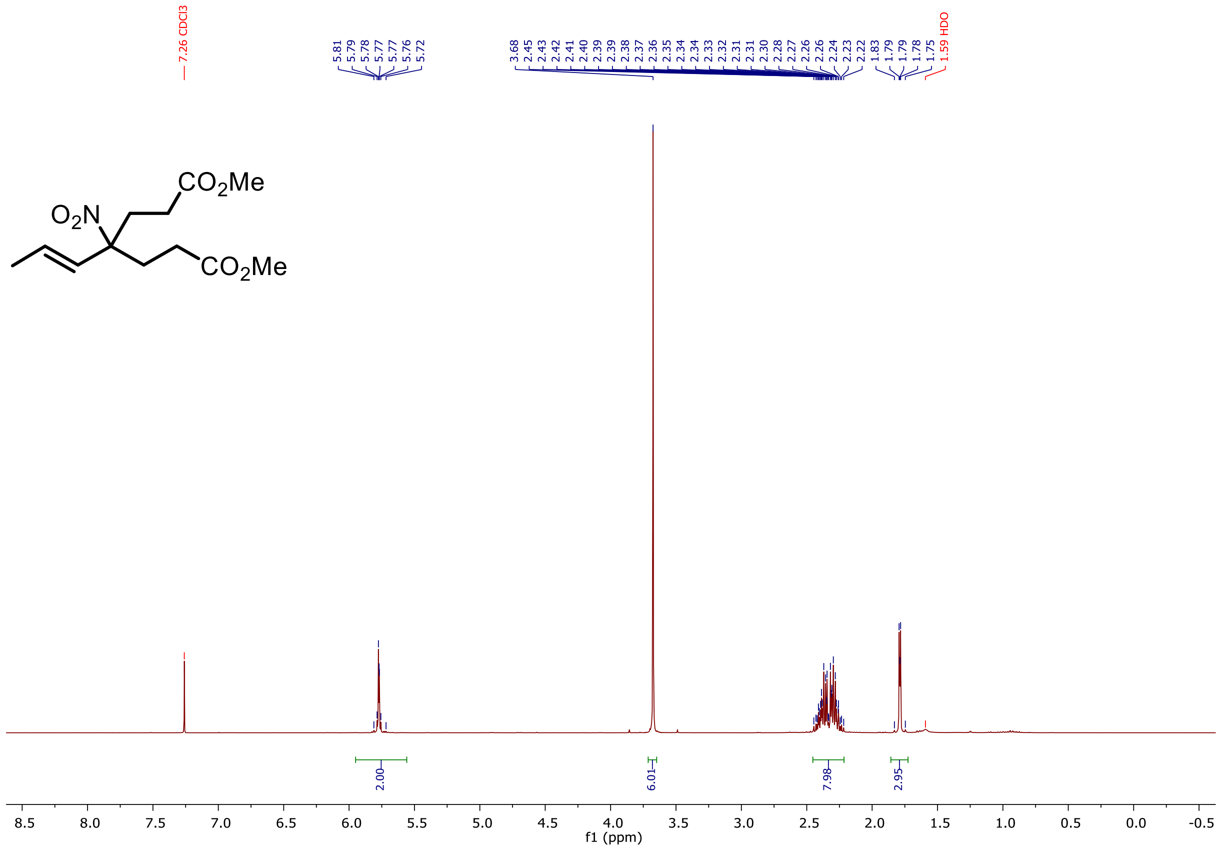

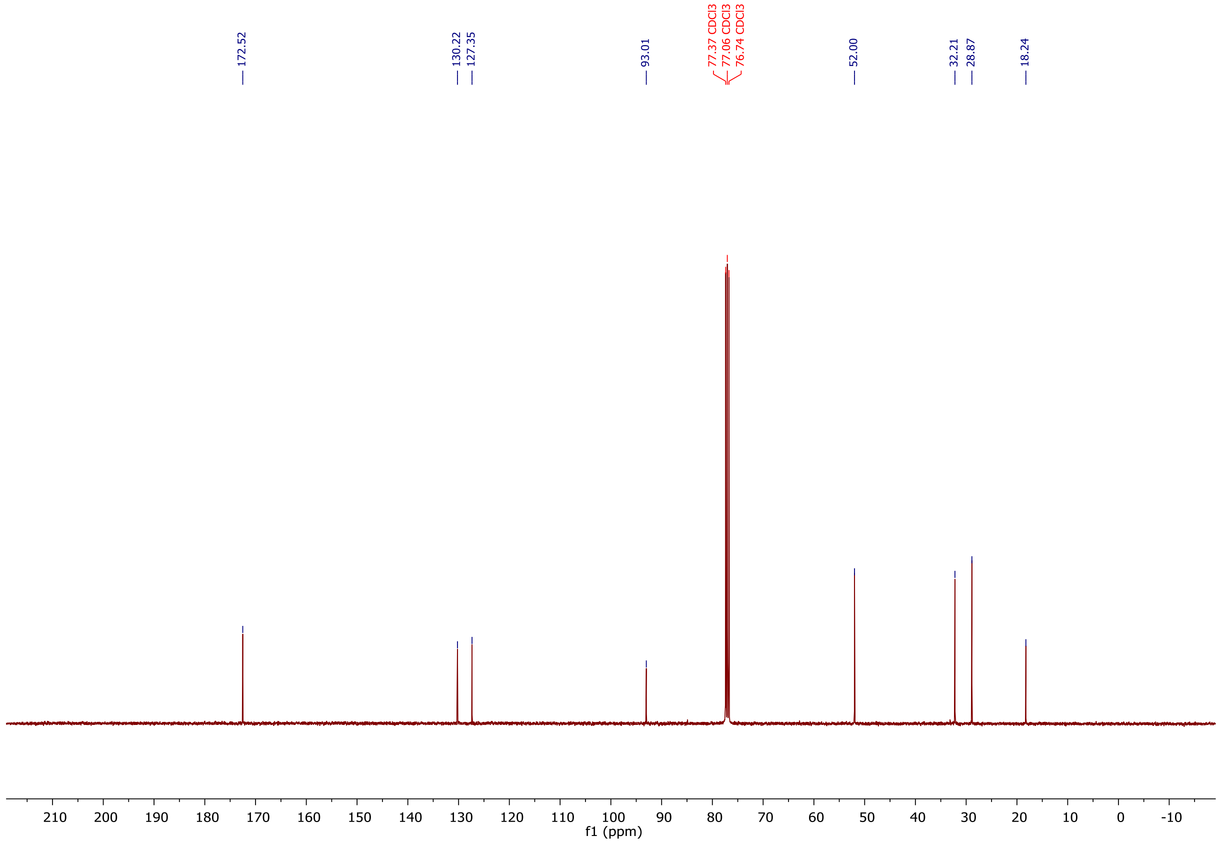

Dimethyl 4-(2-(4,4,5,5-tetramethyl-1,3,2-dioxaborolan-2-yl)propylidene)heptanedioate (4a)

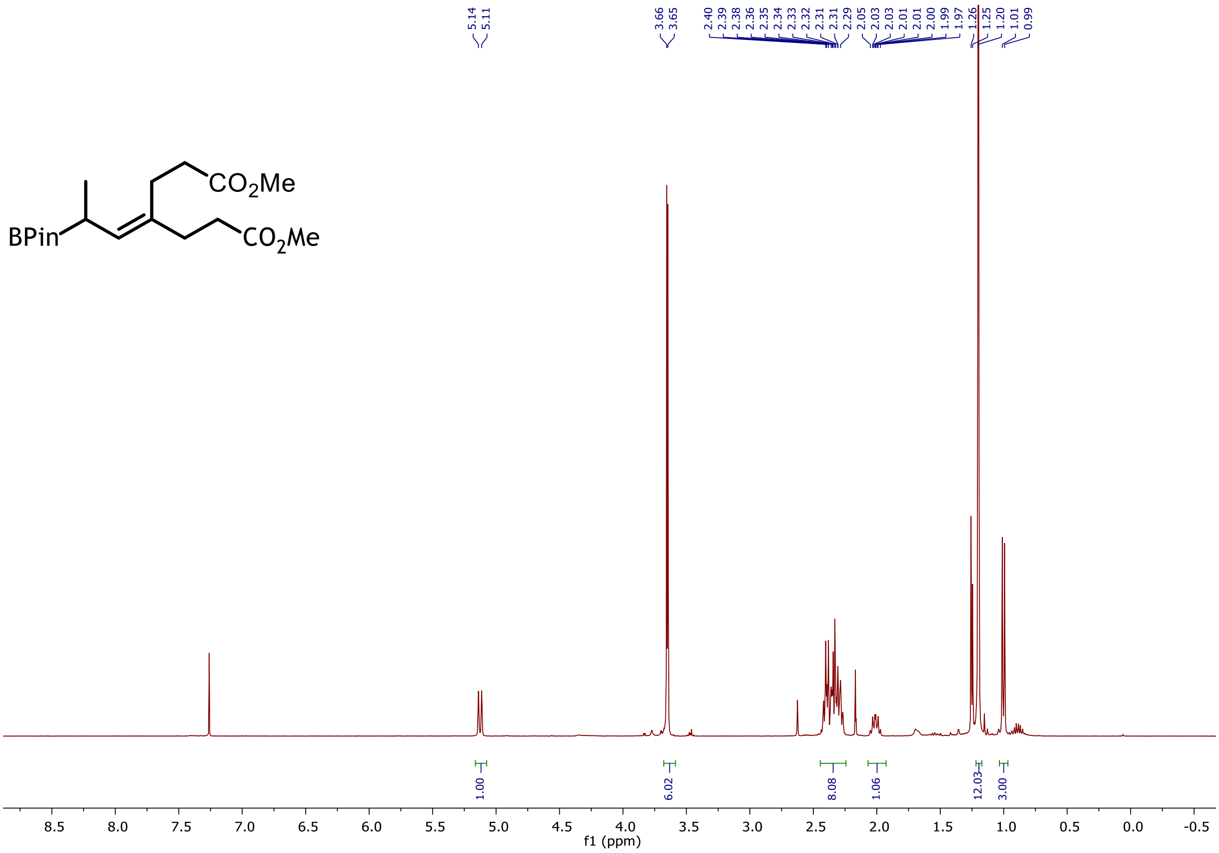

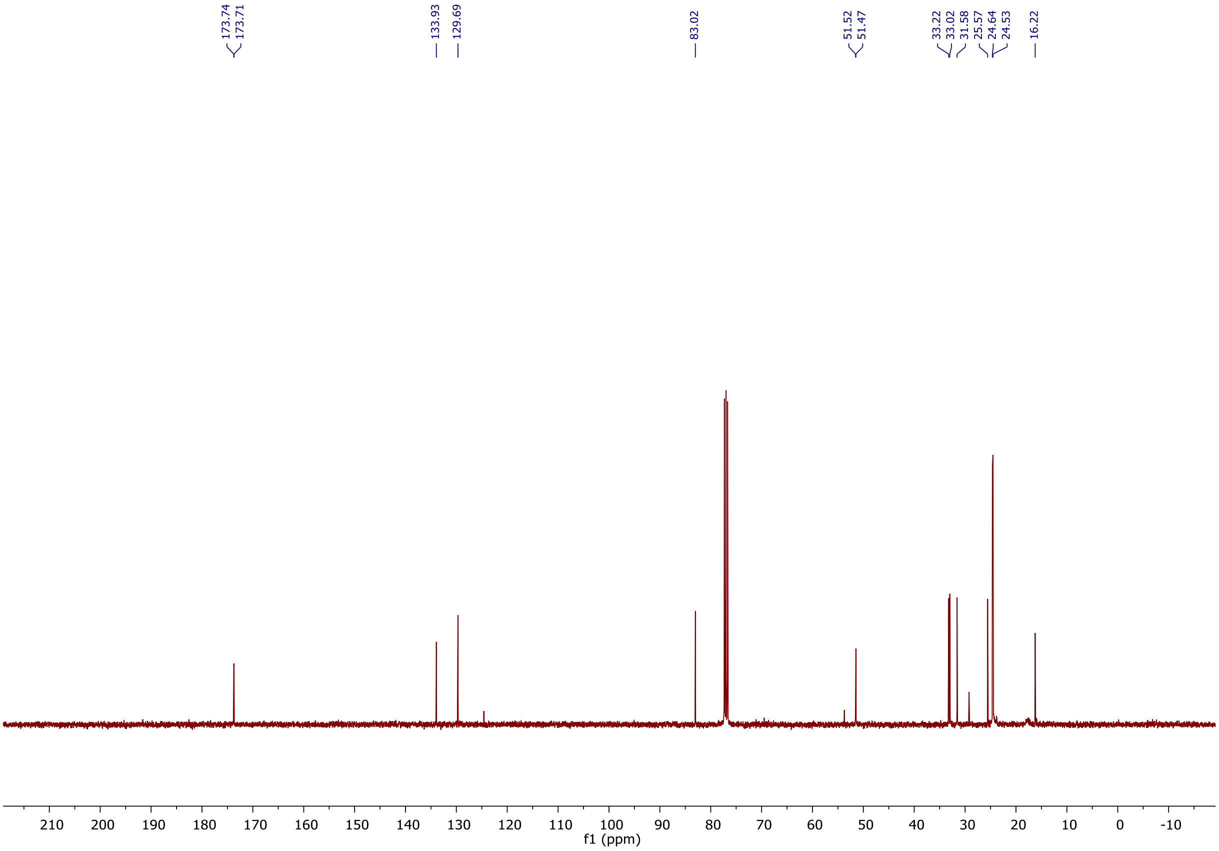

Methyl 3-(6-oxo-3-vinyltetrahydro-2*H*-pyran-3-yl)propanoate (5)

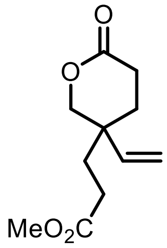

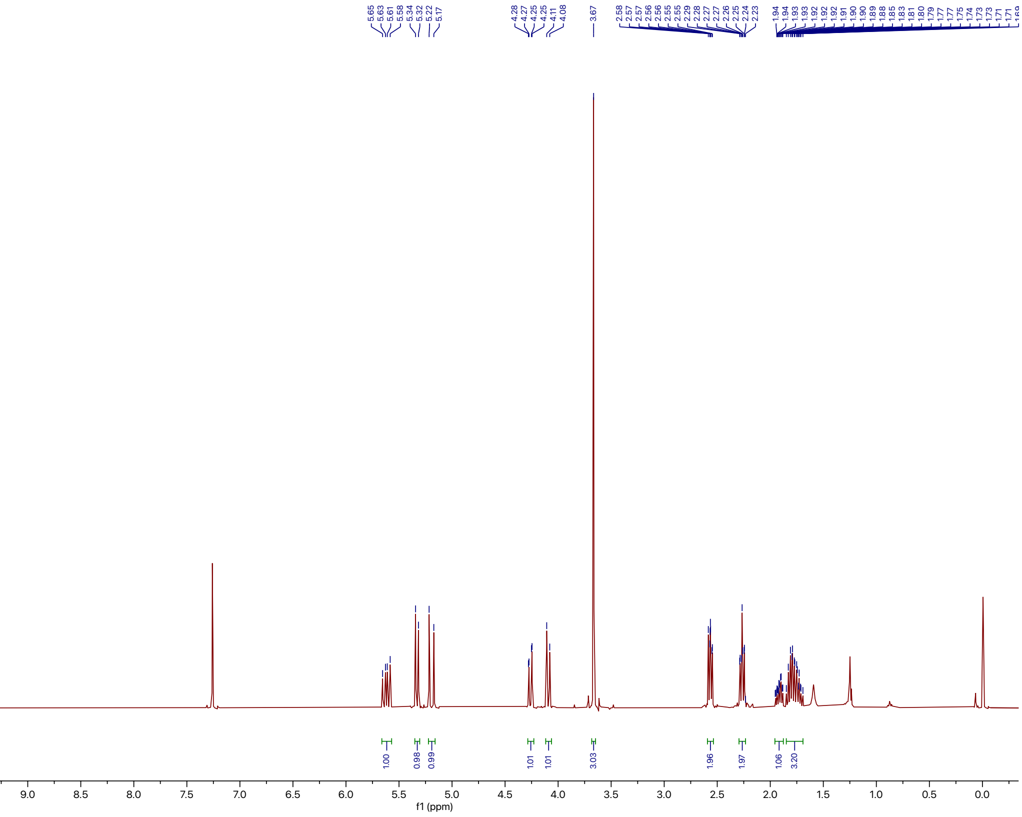

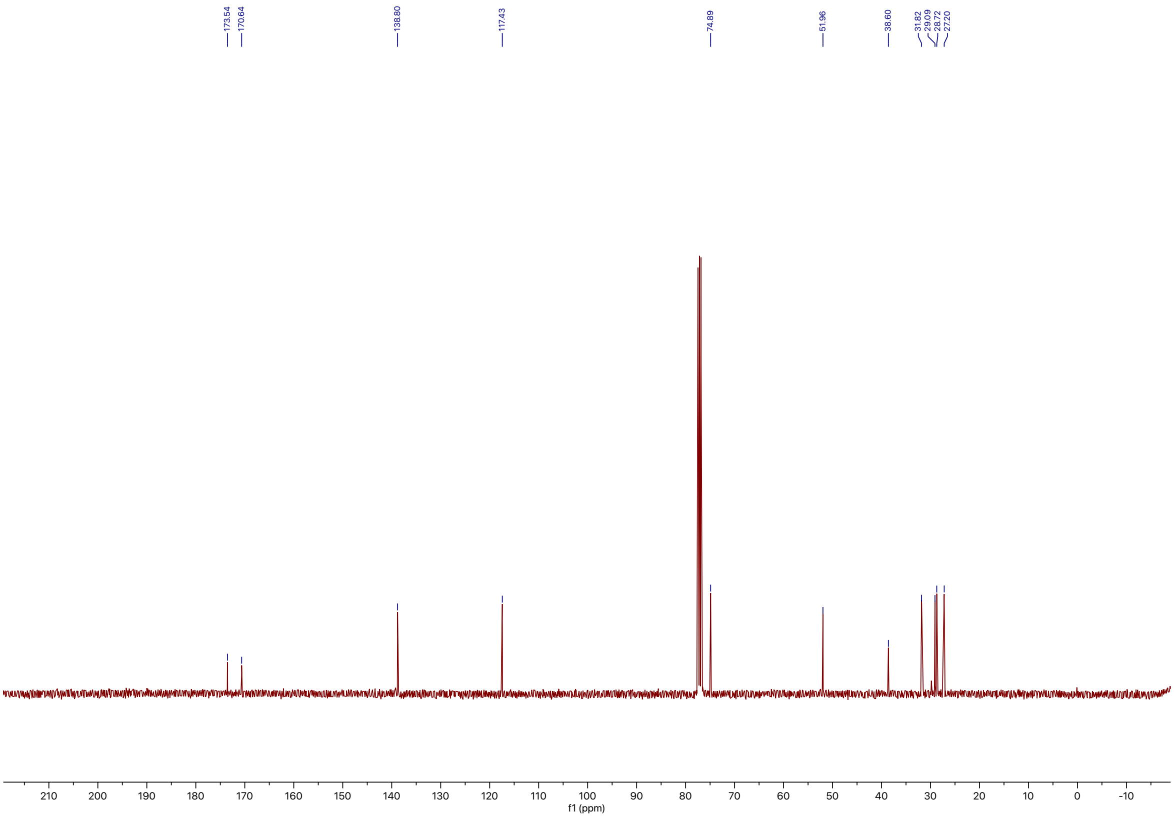

Methyl 3-(2-(4-chlorophenyl)-6-oxo-3-vinyltetrahydro-2*H*-pyran-3-yl)propanoate (*cis*-6)

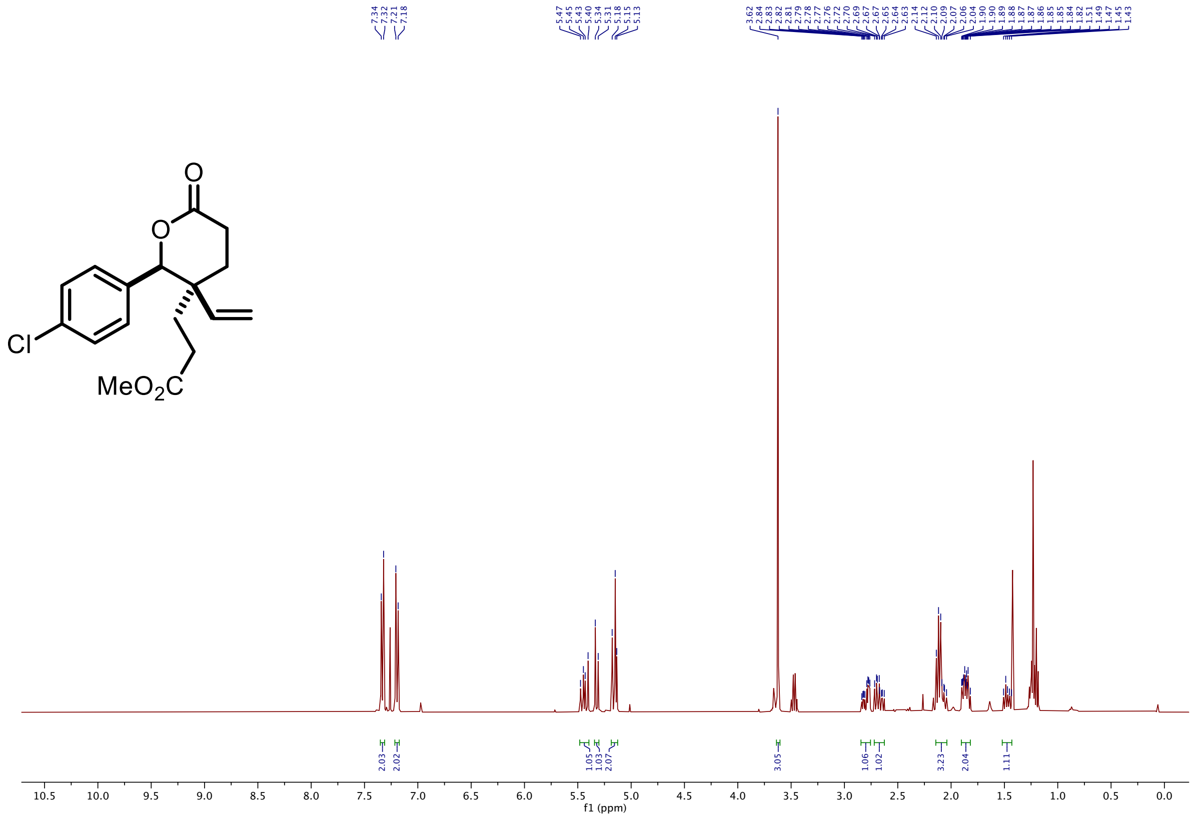

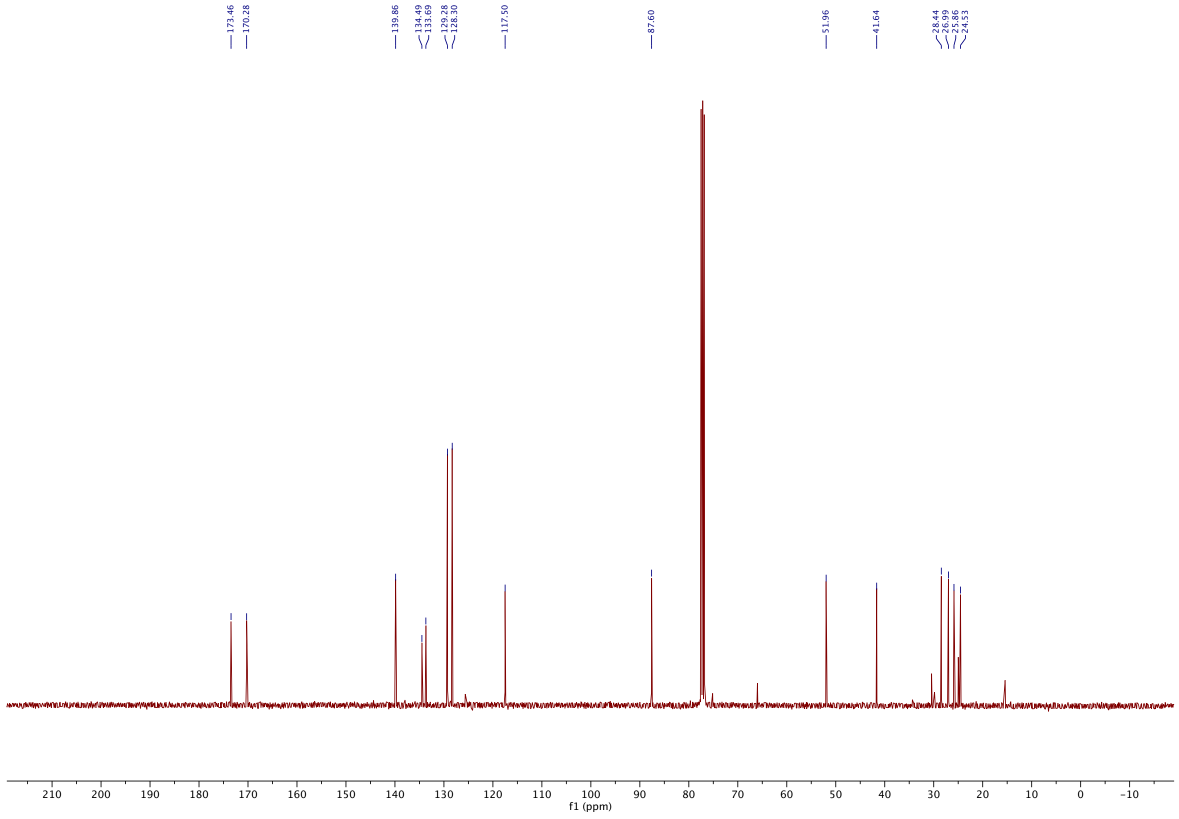

Methyl 3-(2-(4-chlorophenyl)-6-oxo-3-vinyltetrahydro-2*H*-pyran-3-yl)propanoate (*trans*-6)

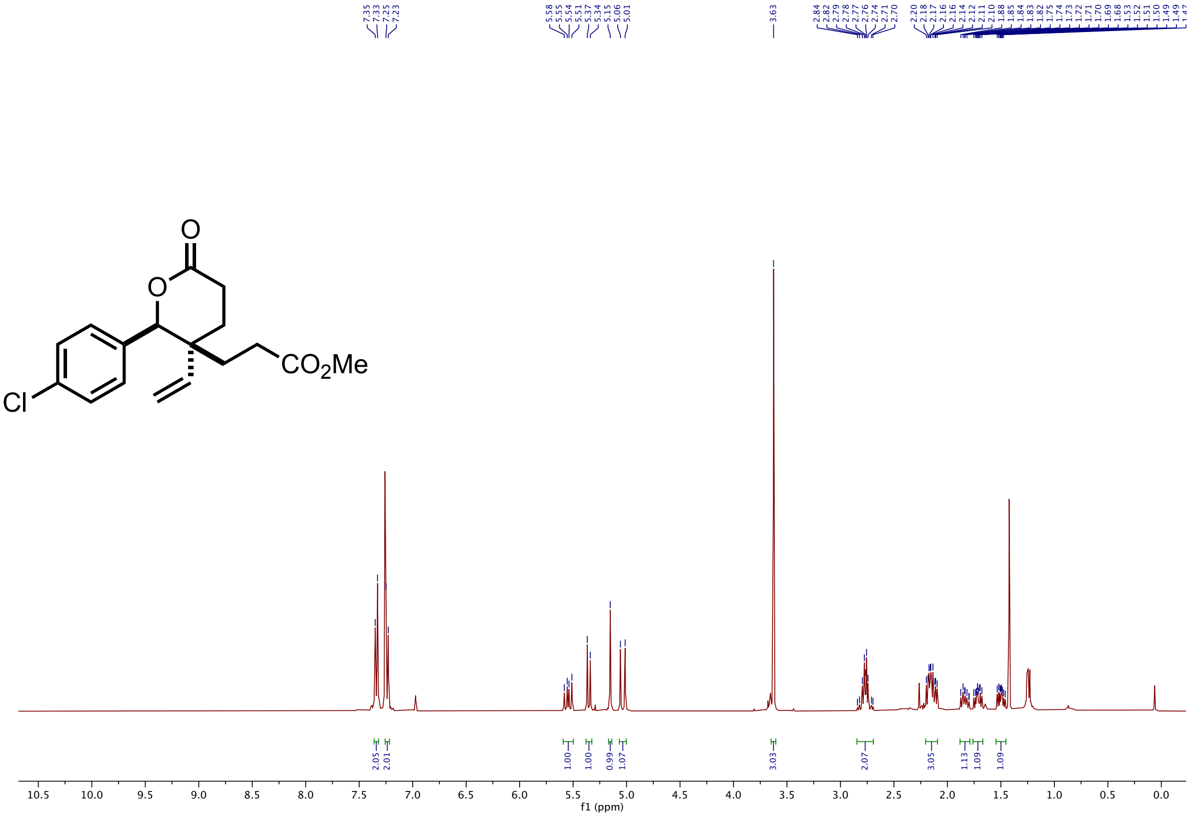

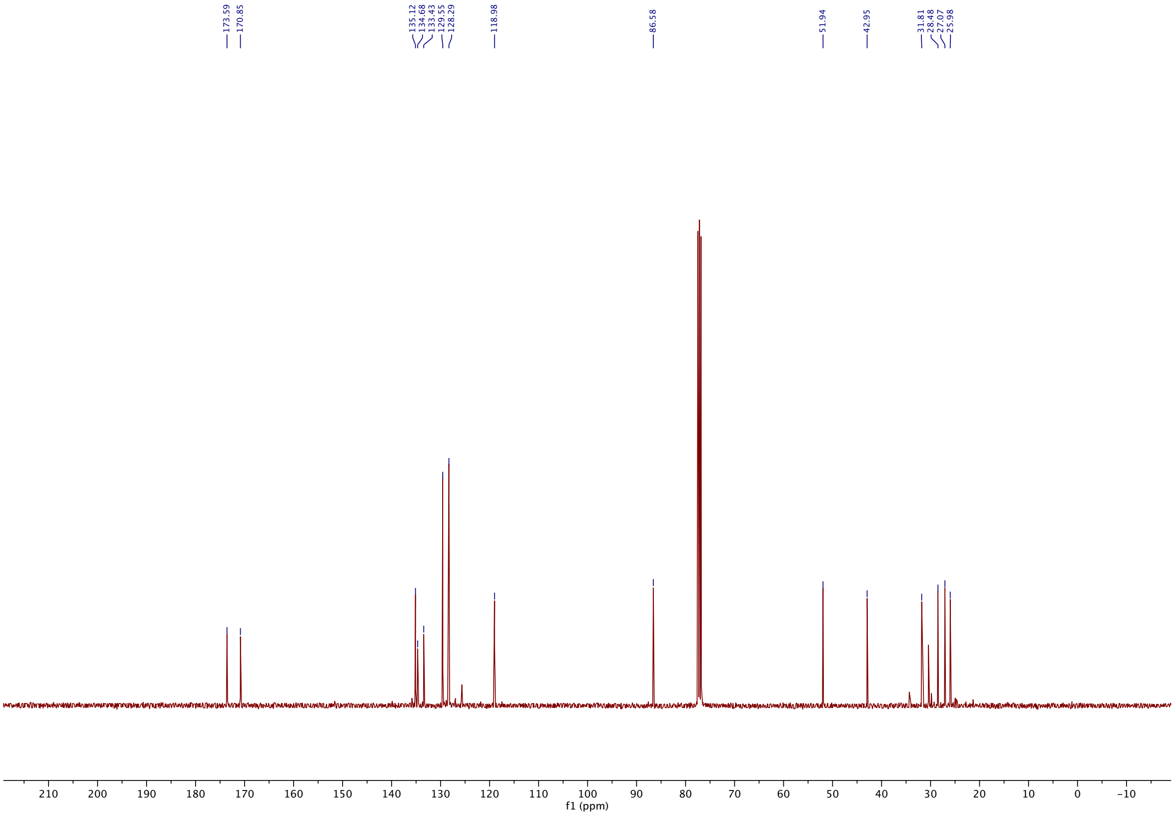

1-(4-Chlorophenyl)-2-fluoro-2-(3-methoxybenzyl)but-3-en-1-ol (7) ***Major isomer***

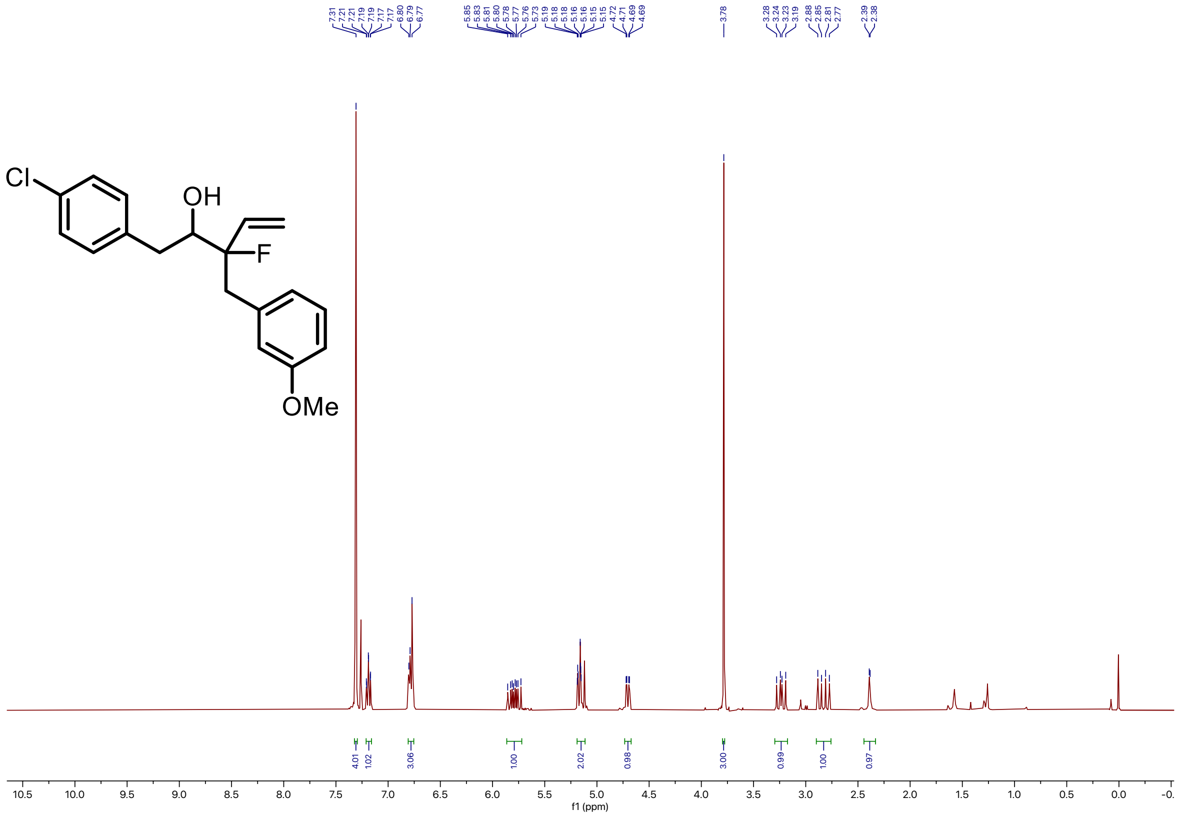

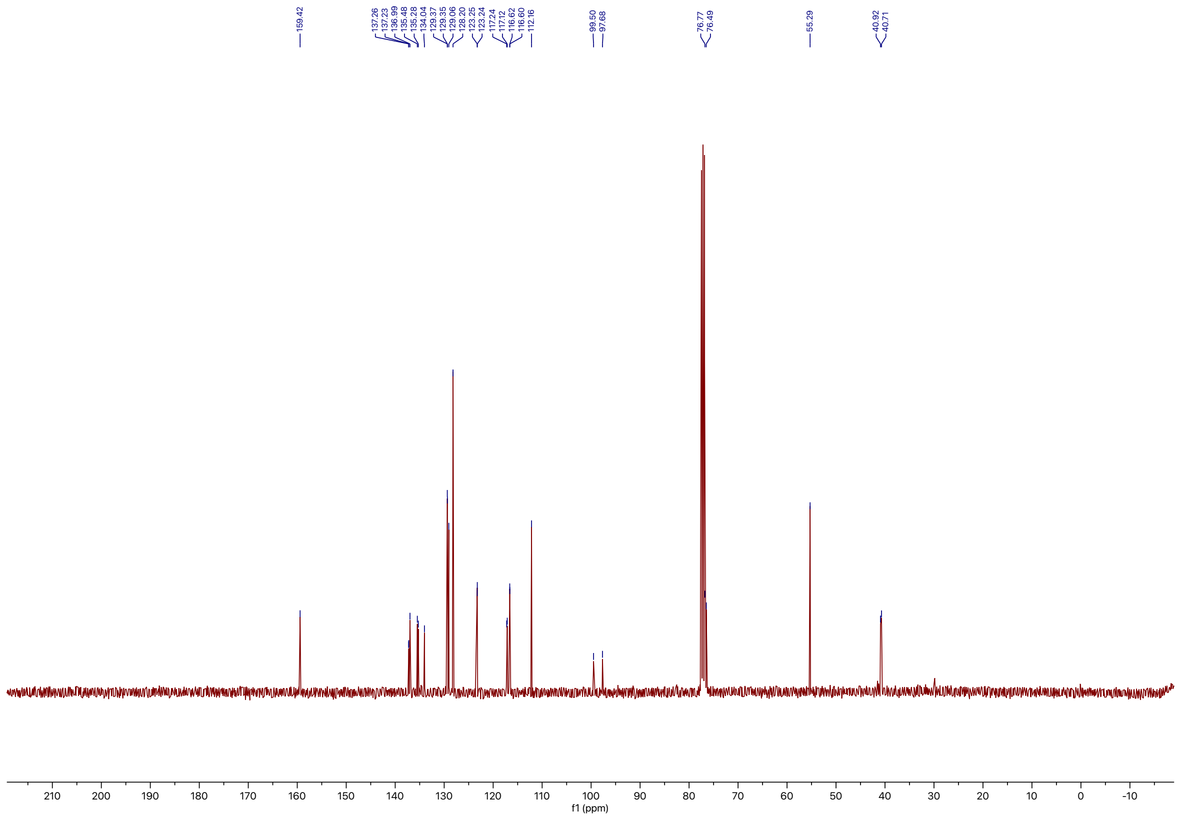

1-(4-Chlorophenyl)-2-fluoro-2-(3-methoxybenzyl)but-3-en-1-ol (7) ***minor isomer + Major isomer***

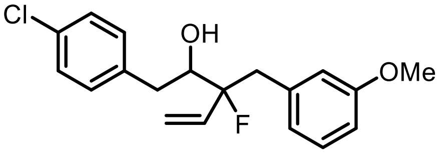

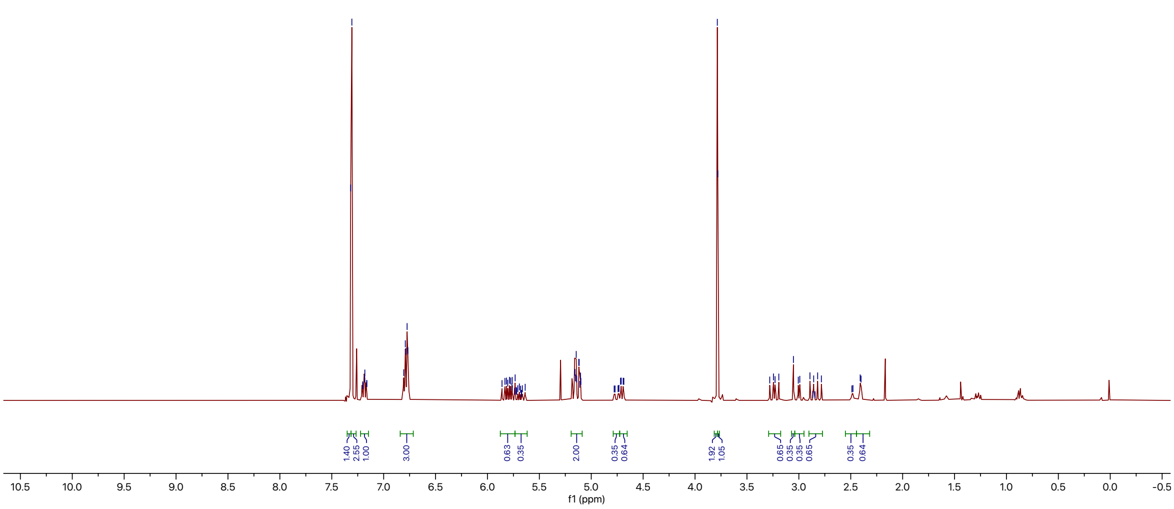

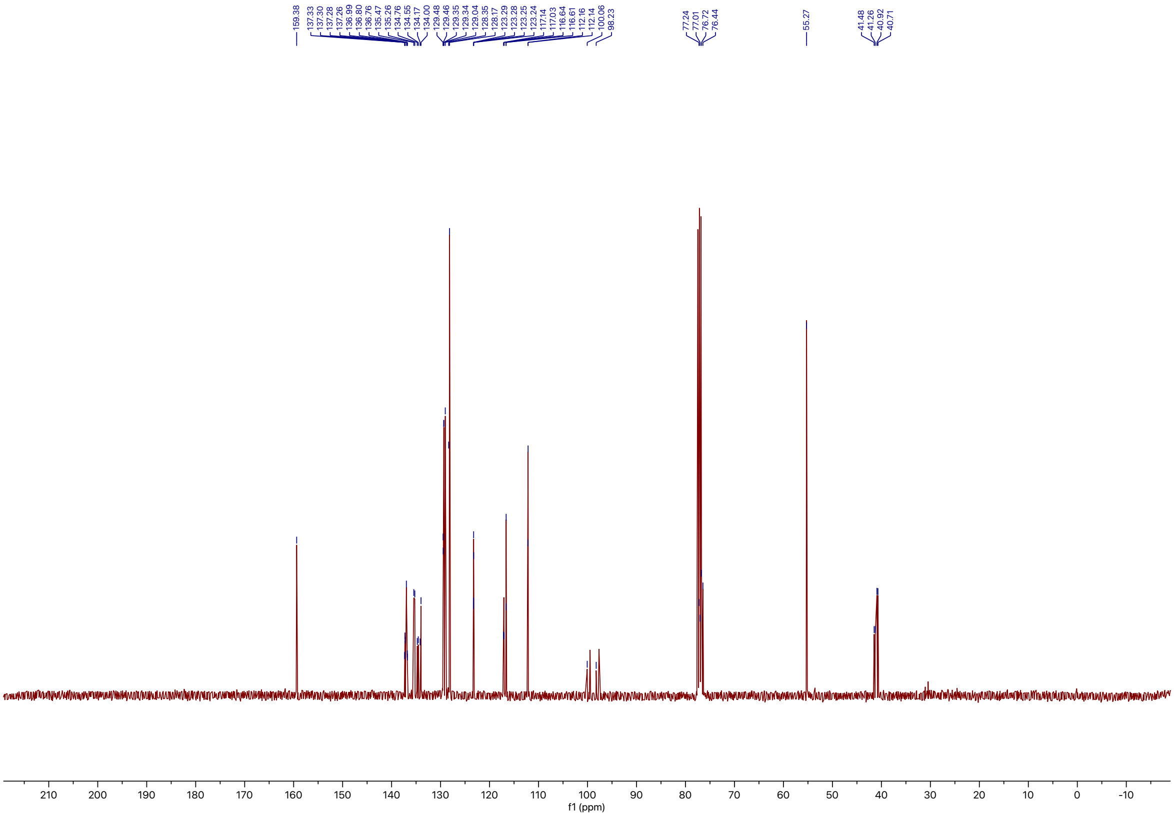

1-Methoxy-4-(1-phenylbuta-1,3-dien-2-yl) benzene (8)

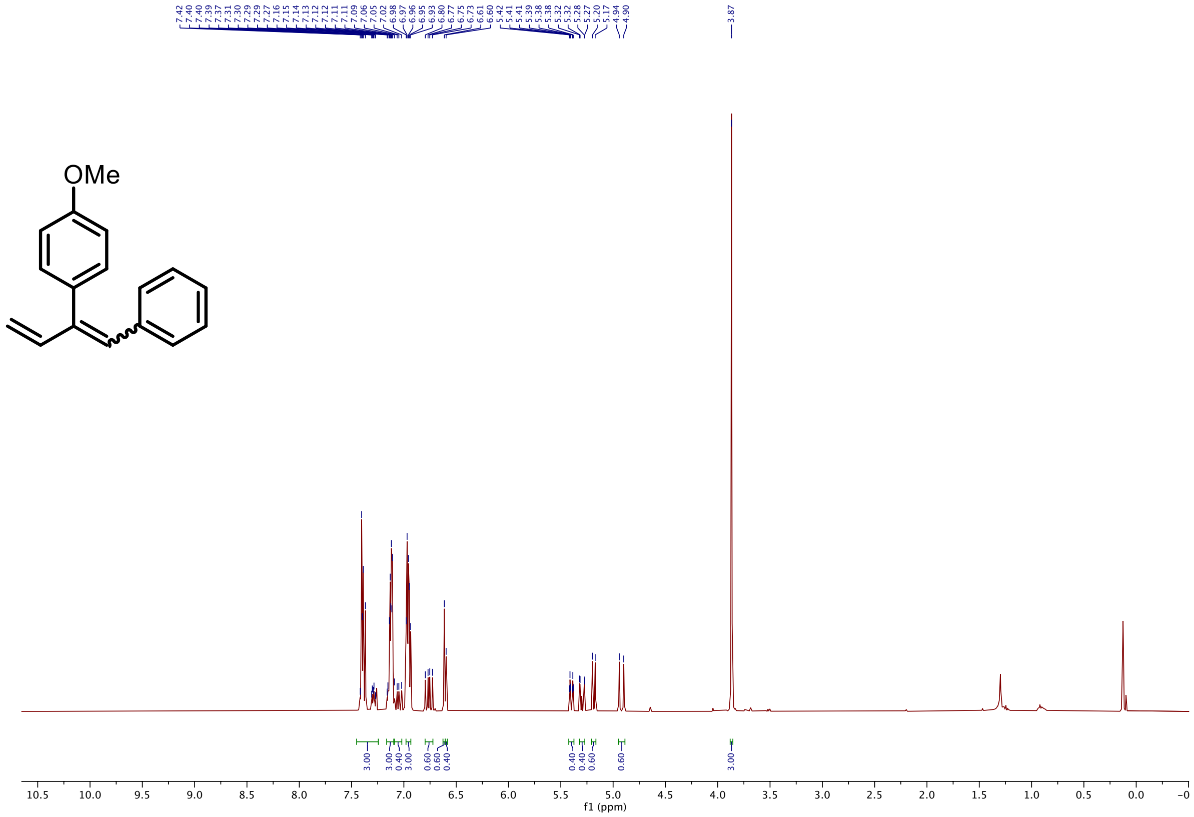

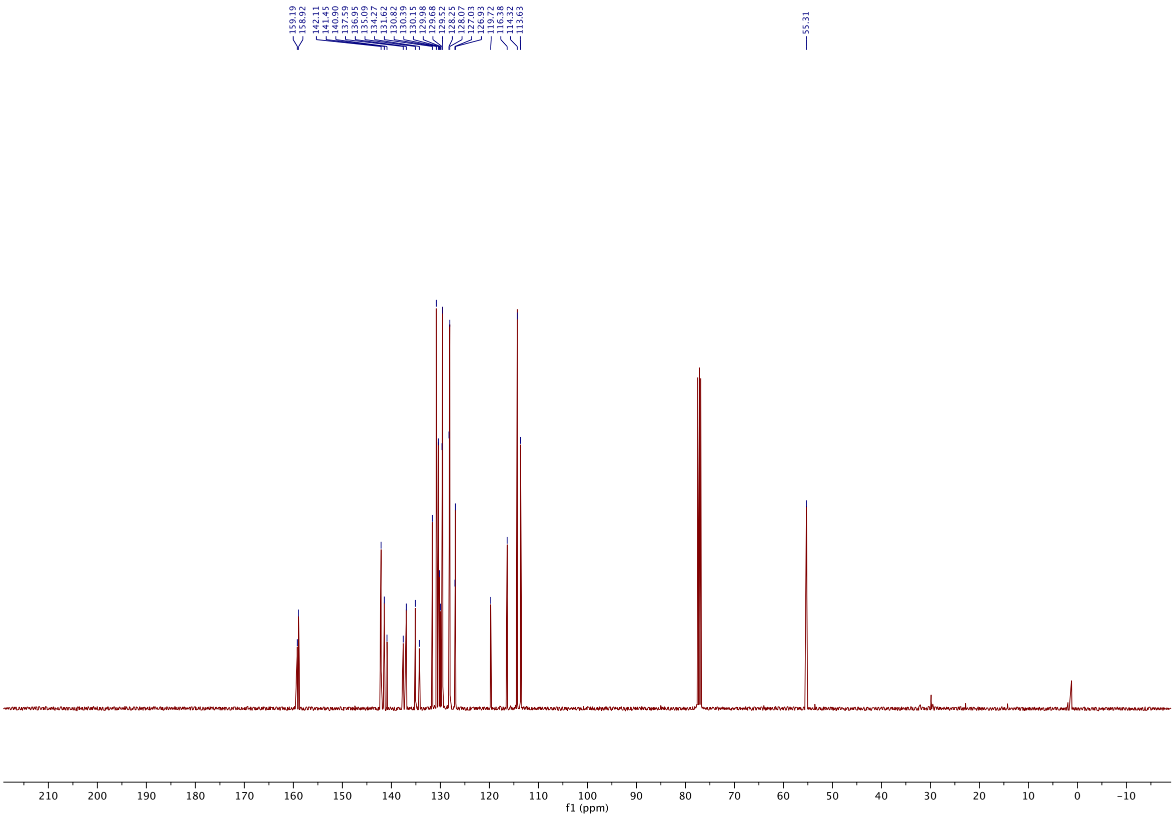

Dimethyl 4-(1-(4-chlorophenyl)-2-hydroxyethylidene)heptanedioate (9)

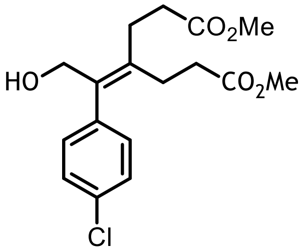

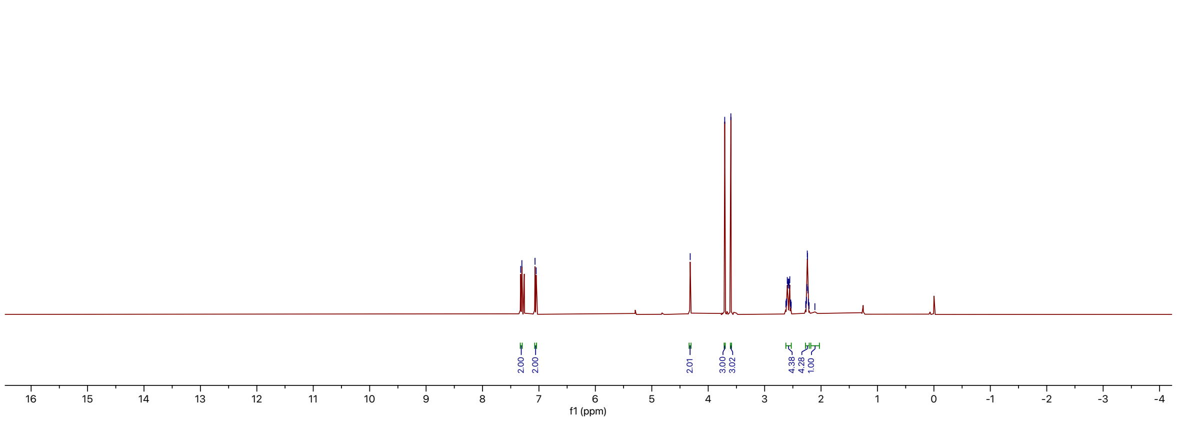

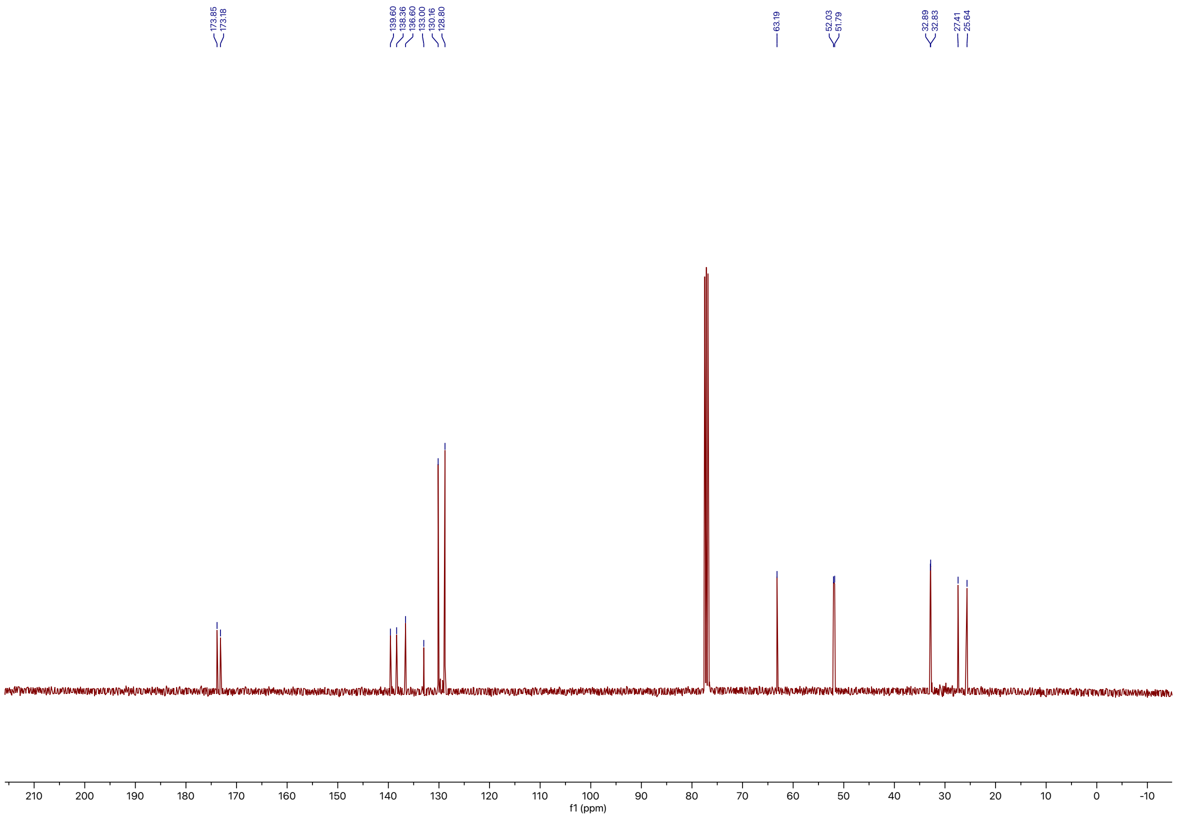

Methyl 3-((2*R*,3*R*)-2-(4-chlorophenyl)-3-(1-(4-chlorophenyl)vinyl)-6-oxotetrahydro-2*H*-pyran-3-yl)propanoate (*cis*-10)

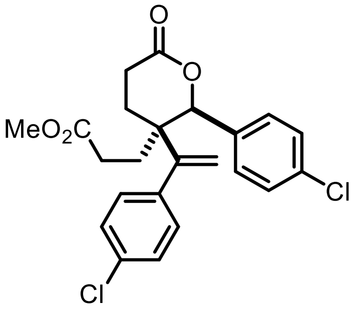

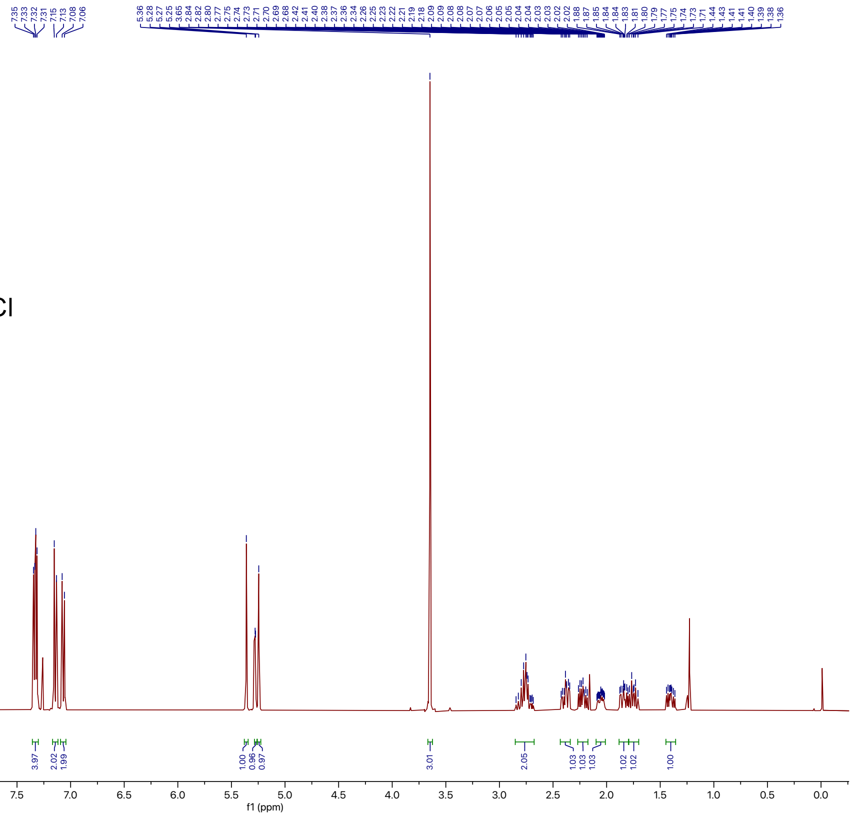

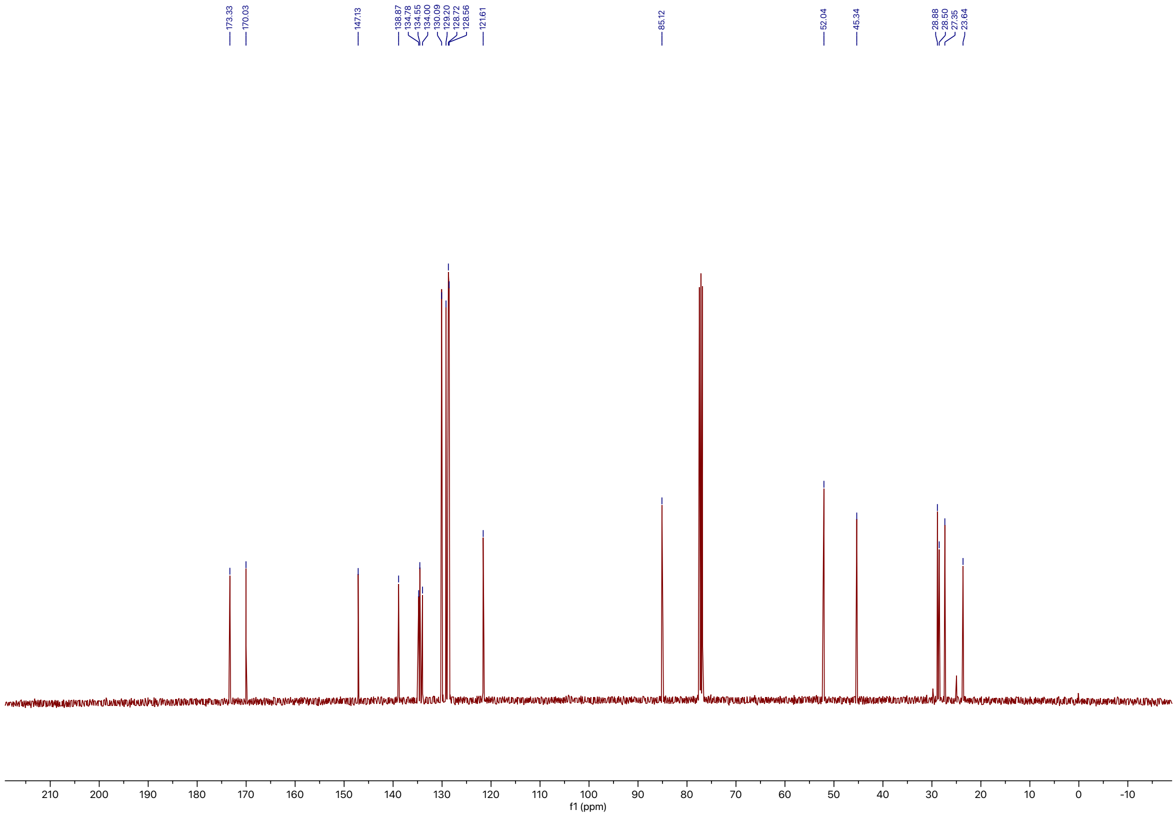

NOESY NMR of compound *cis*-10

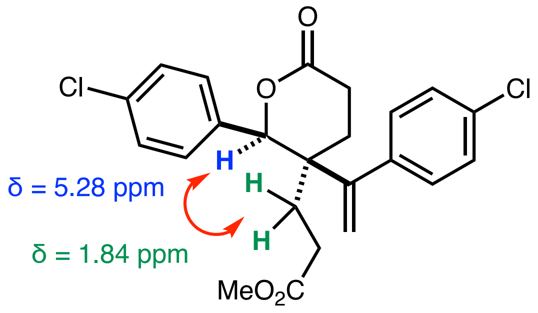

NOESY spectrum
(400 MHz, $CDCl_3$)

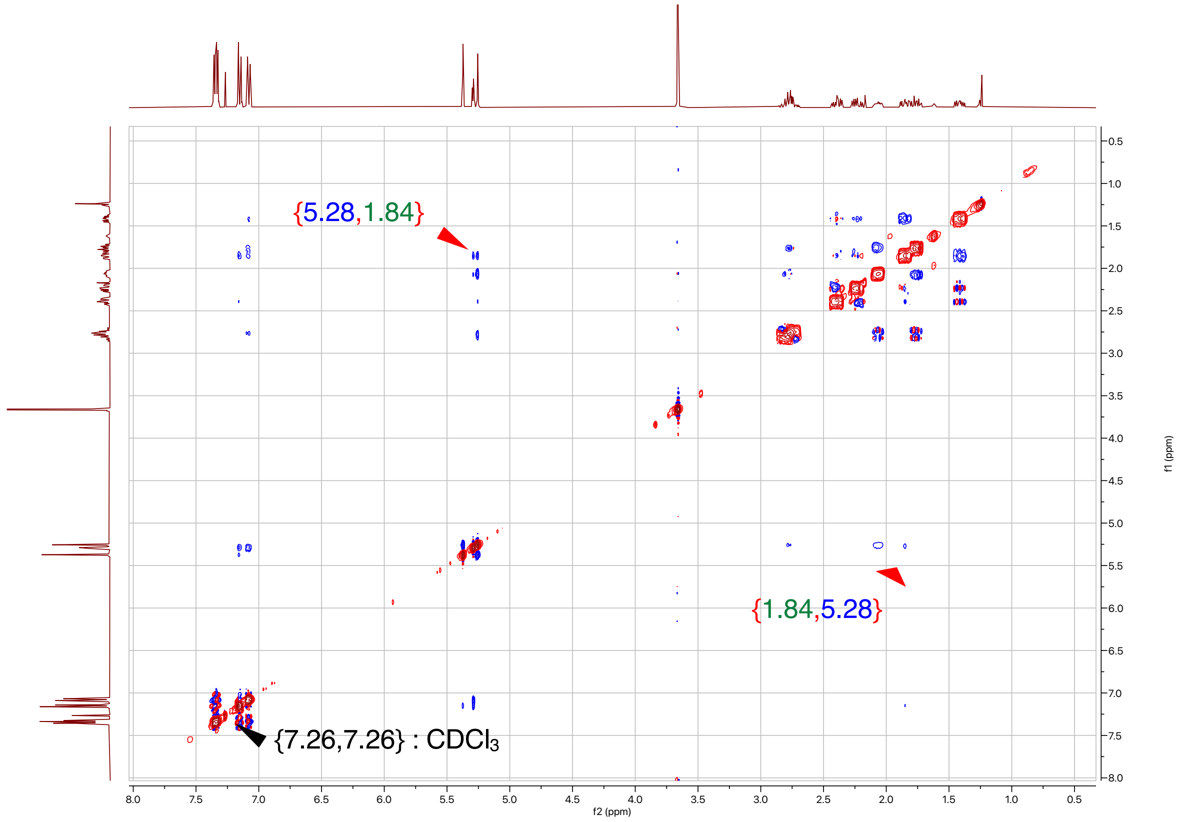

Methyl 3-(2-(1-(4-chlorophenyl)vinyl)-5-oxotetrahydrofuran-2-yl)propanoate (11)

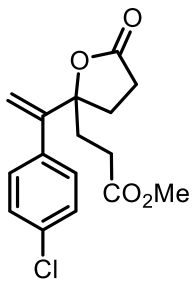

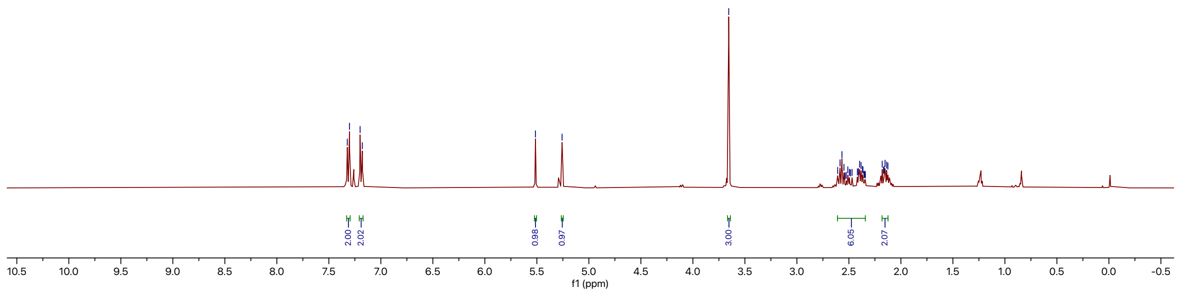

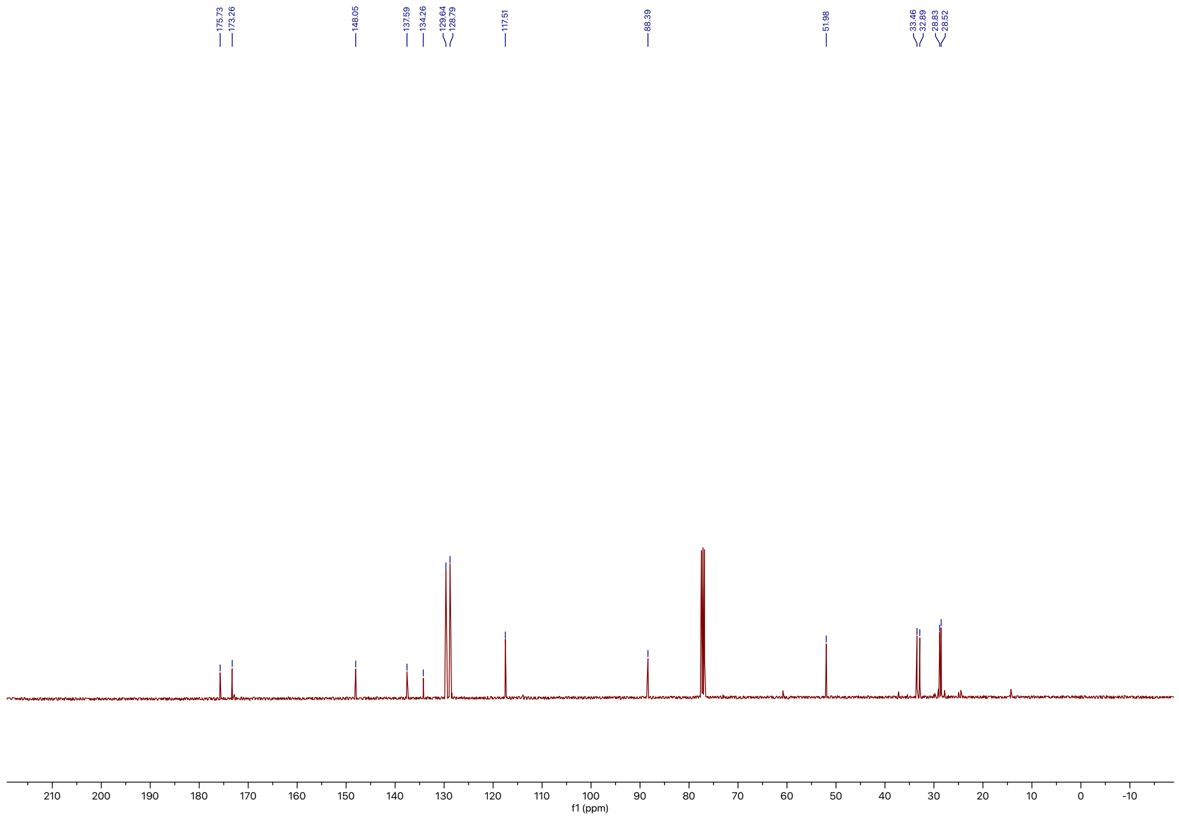

4,4'-[2-(Phenylsulfonyl)-2-vinylpropane-1,3-diyl]bis(methylbenzene) (12a)

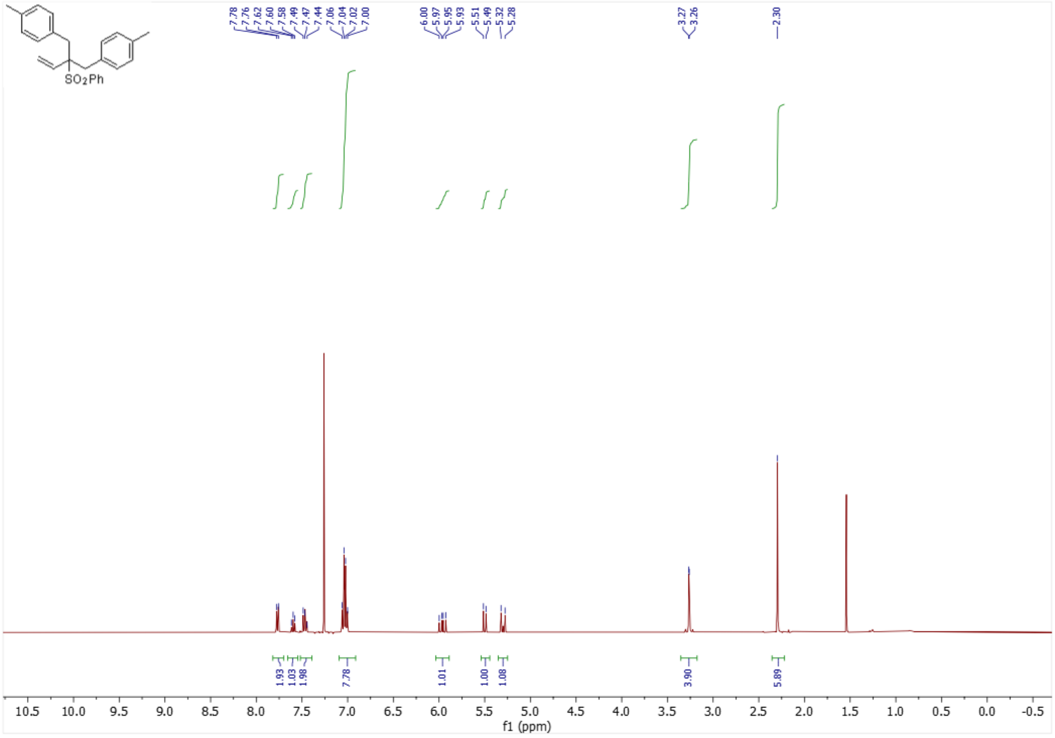

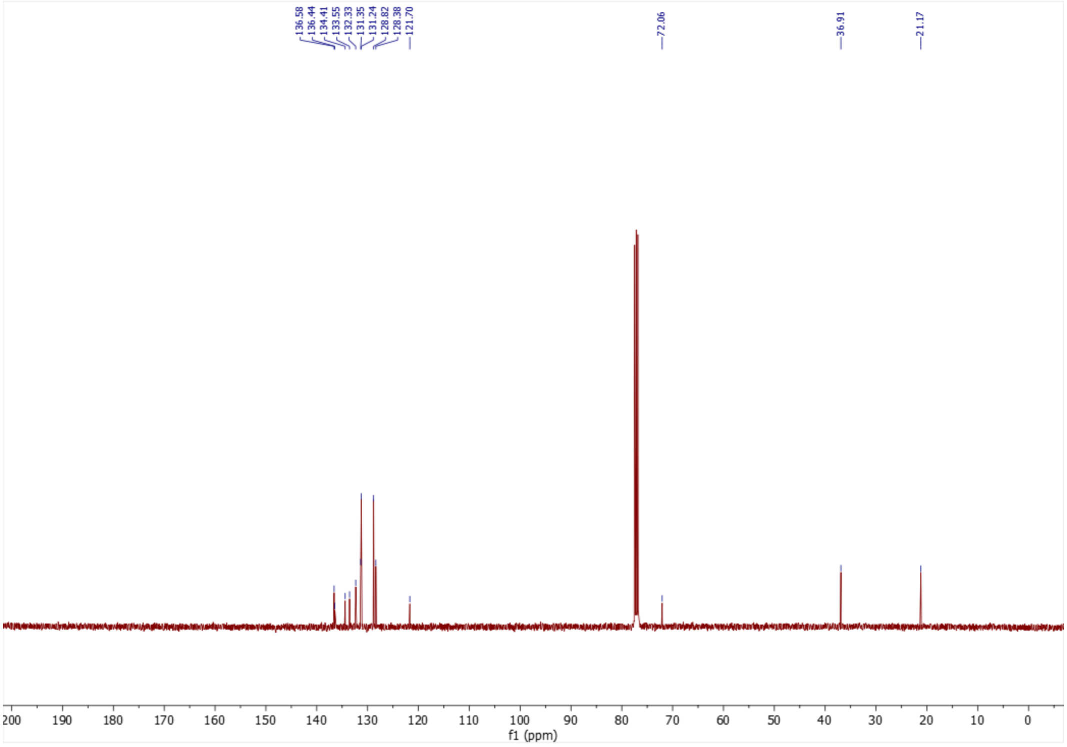

5,5'-[2-(Phenylsulfonyl)-2-vinylpropane-1,3-diyl]bis(1,3-dimethoxybenzene) (12b)

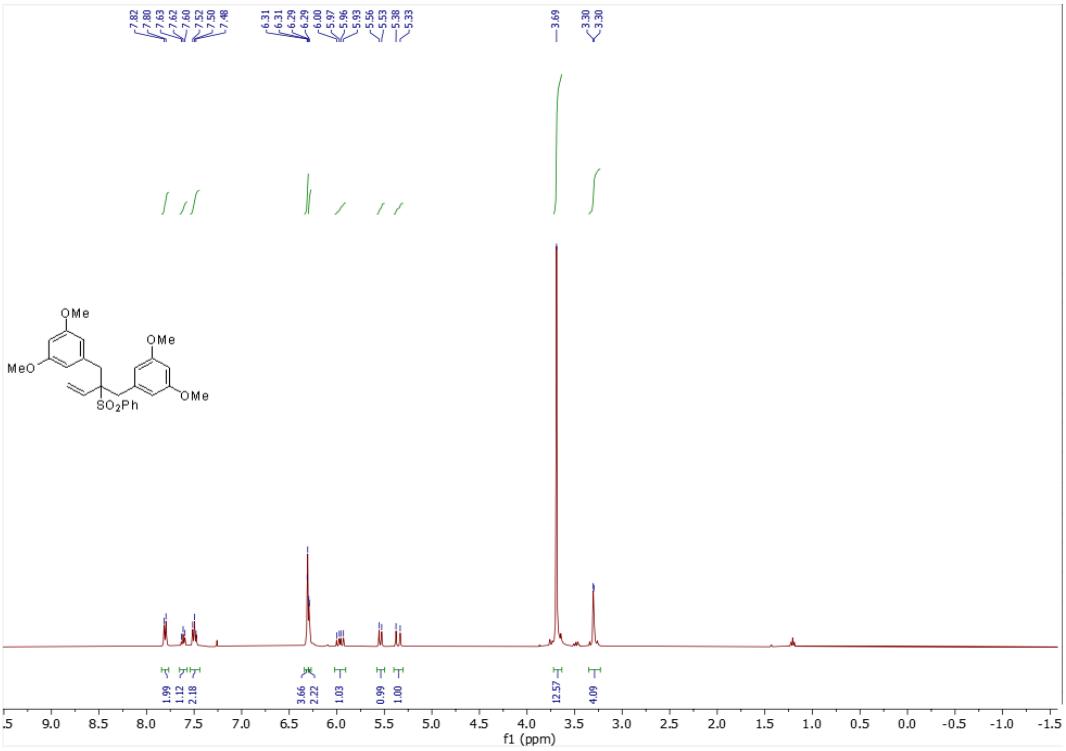

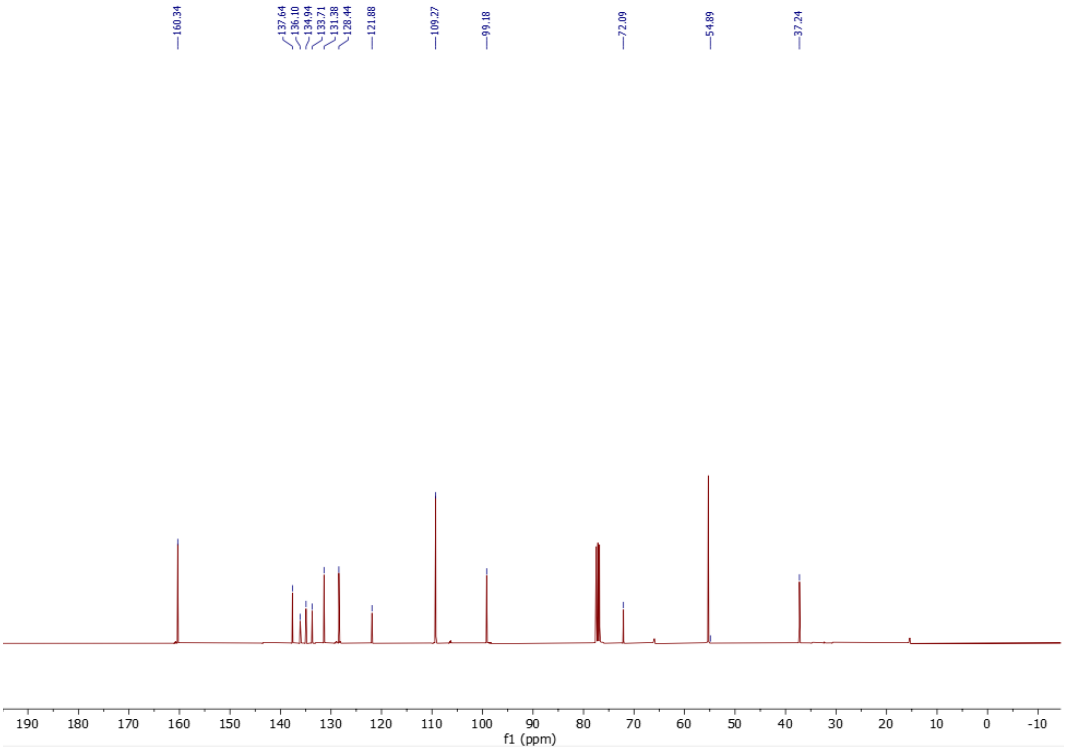

[(4-Vinylhepta-1,6-dien-4-yl]sulfonyl)benzene (12c)

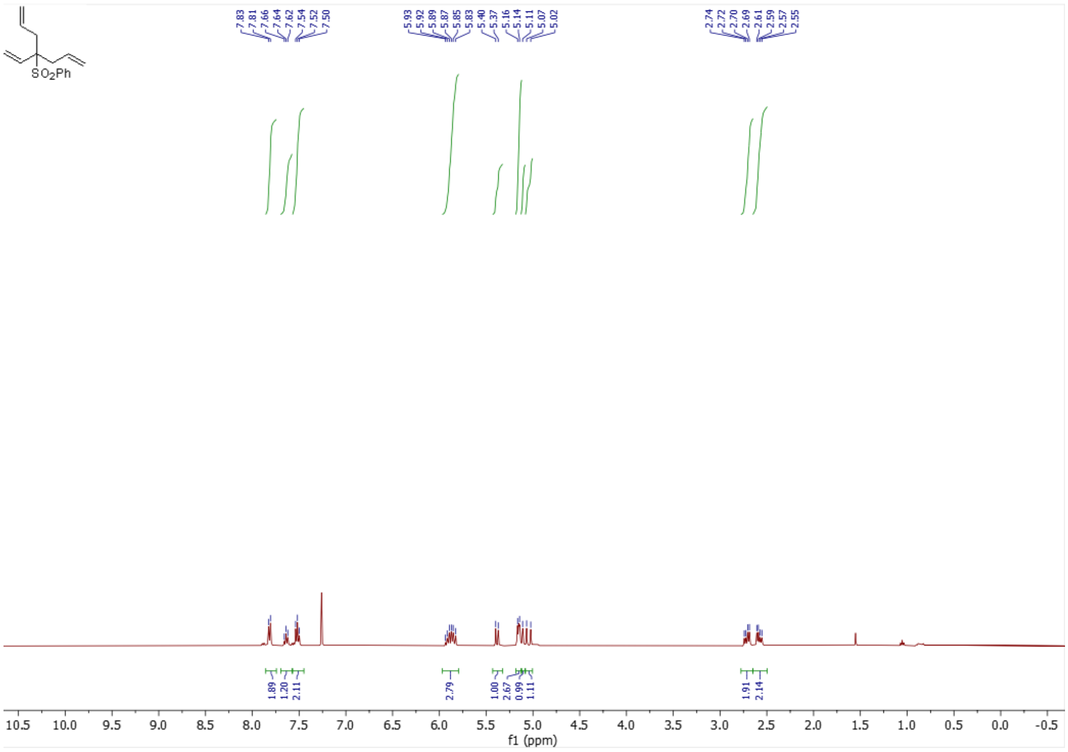

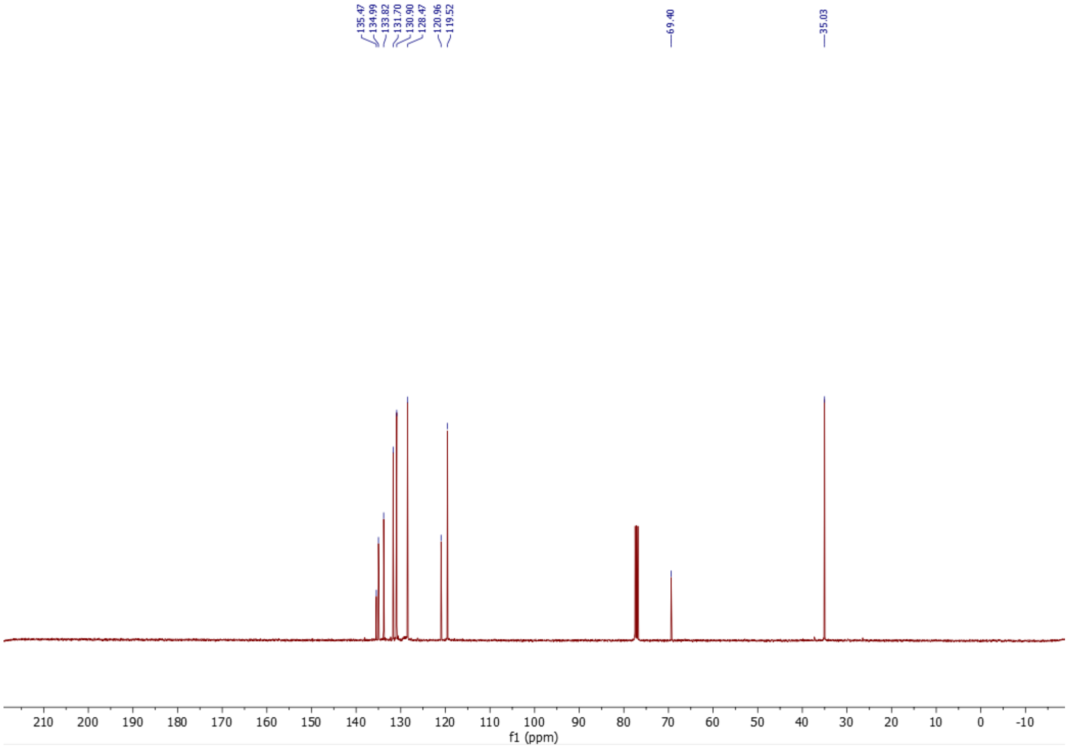

[(1*E*,6*E*)-4-(Phenylsulfonyl)-4-vinylhepta-1,6-diene-1,7-diyl]dibenzene (12d)

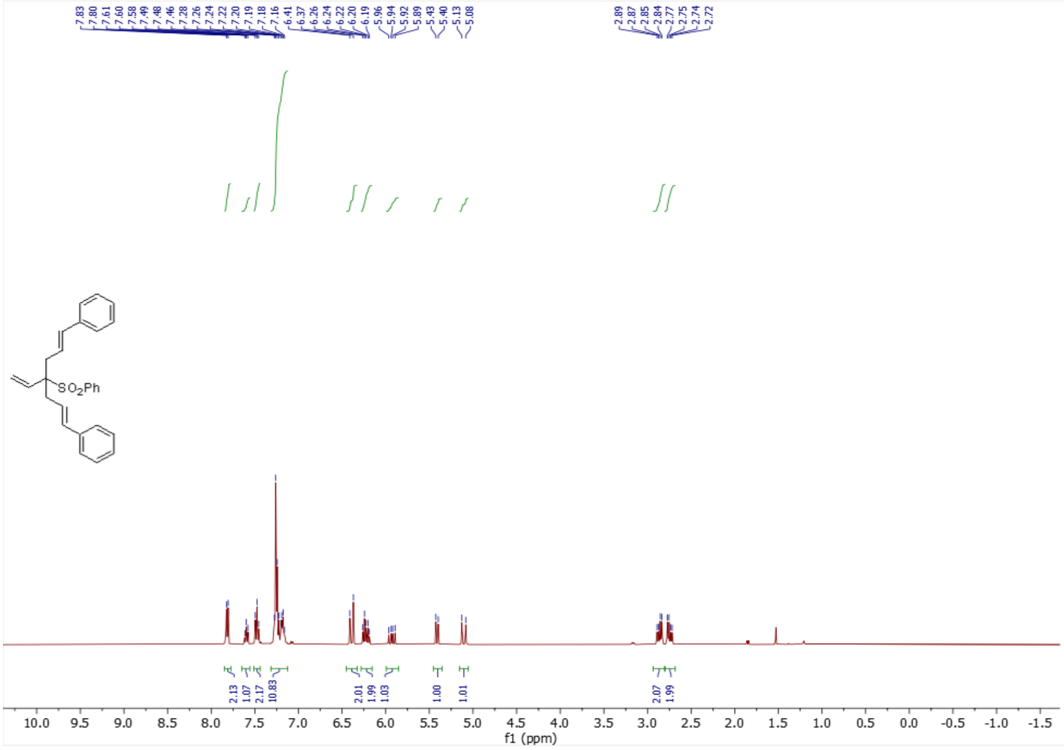

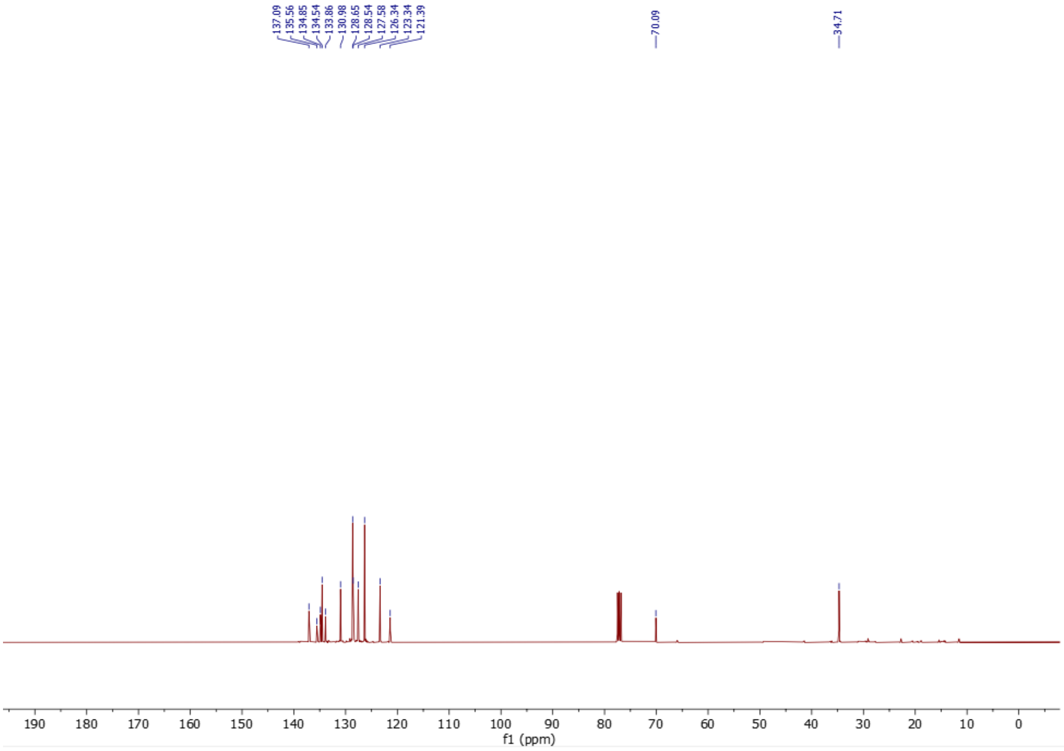

[(2,8-Dimethyl-5-vinylnona-2,7-dien-5-yl)sulfonyl]benzene (12e)

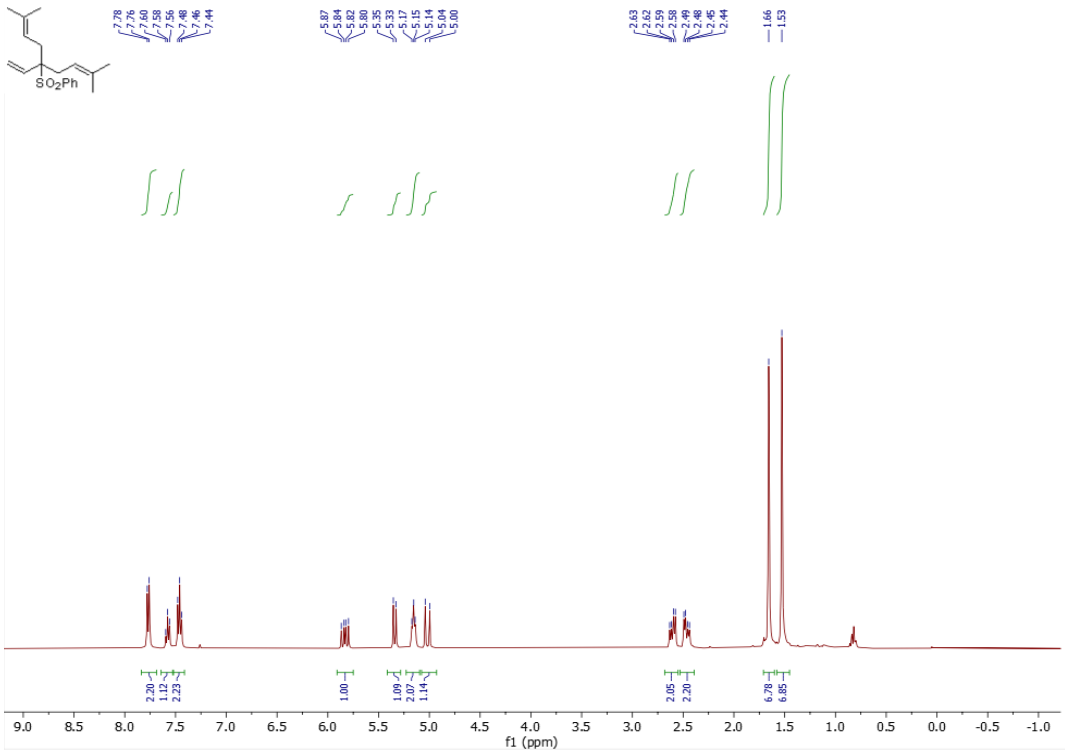

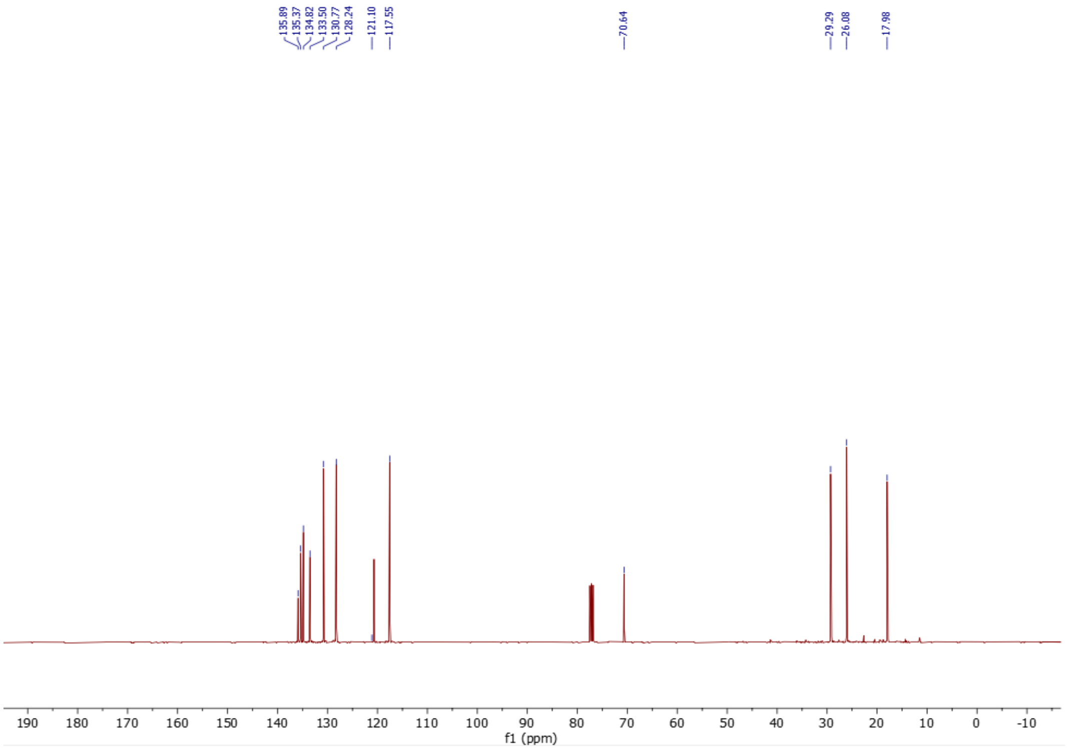

1-Methyl-4-[2-(phenylsulfonyl)but-3-en-1-yl]benzene (12f)

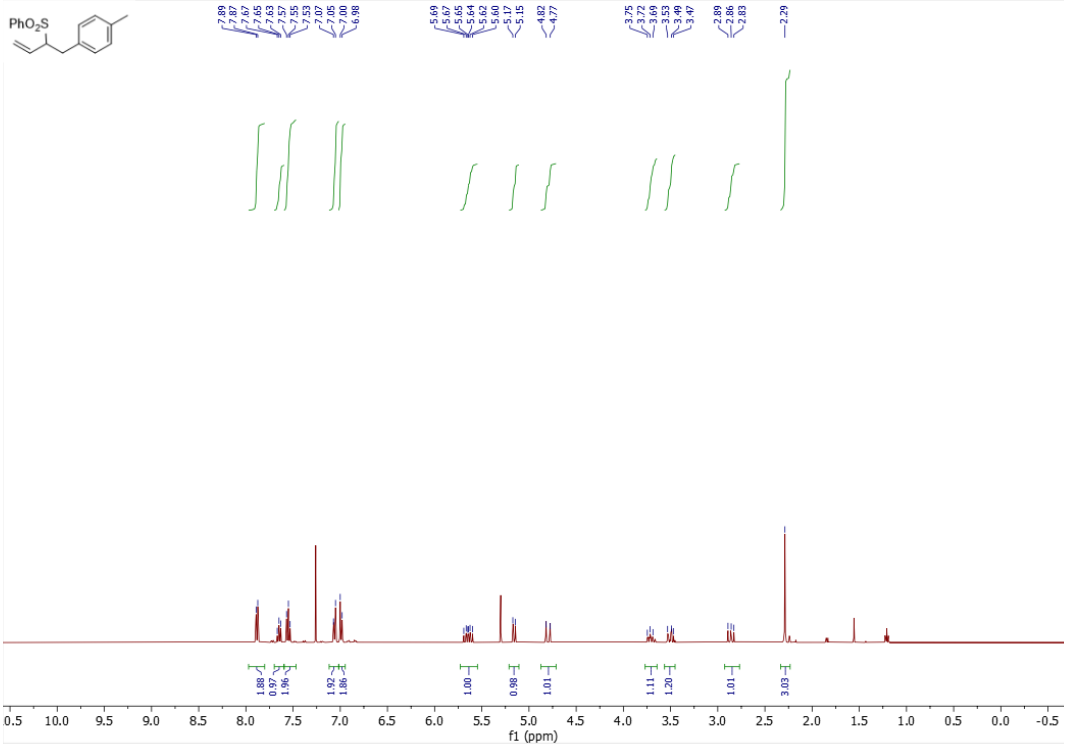

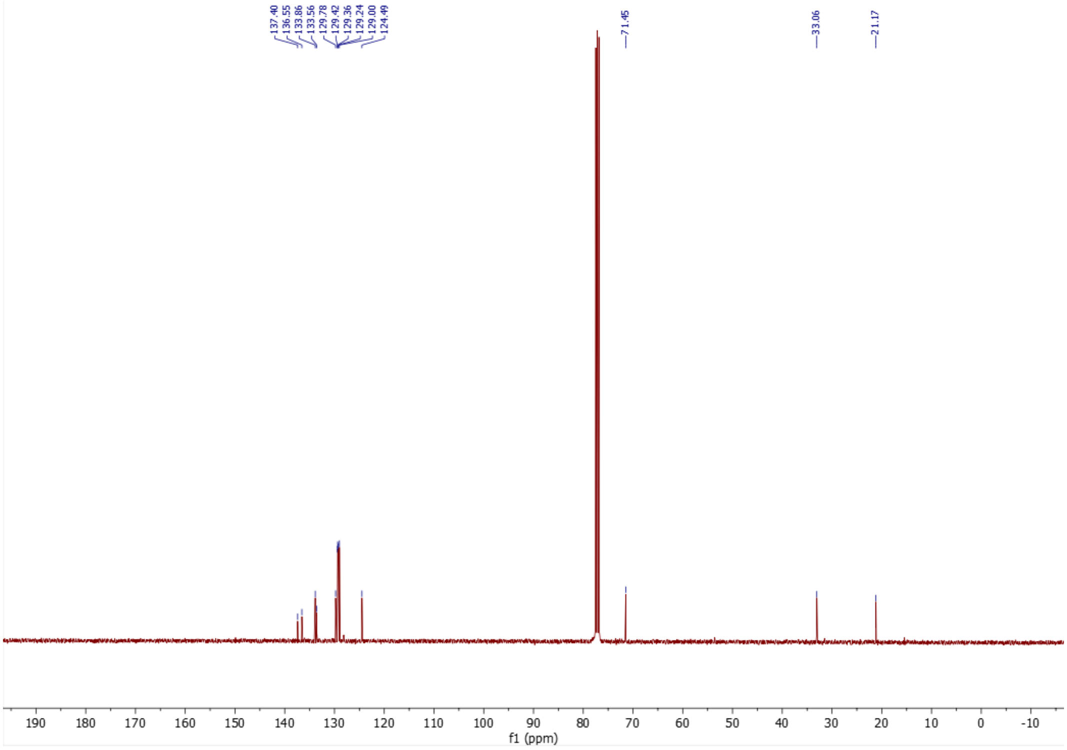

1,3-Dimethoxy-5-[2-(phenylsulfonyl)but-3-en-1-yl]benzene (12g)

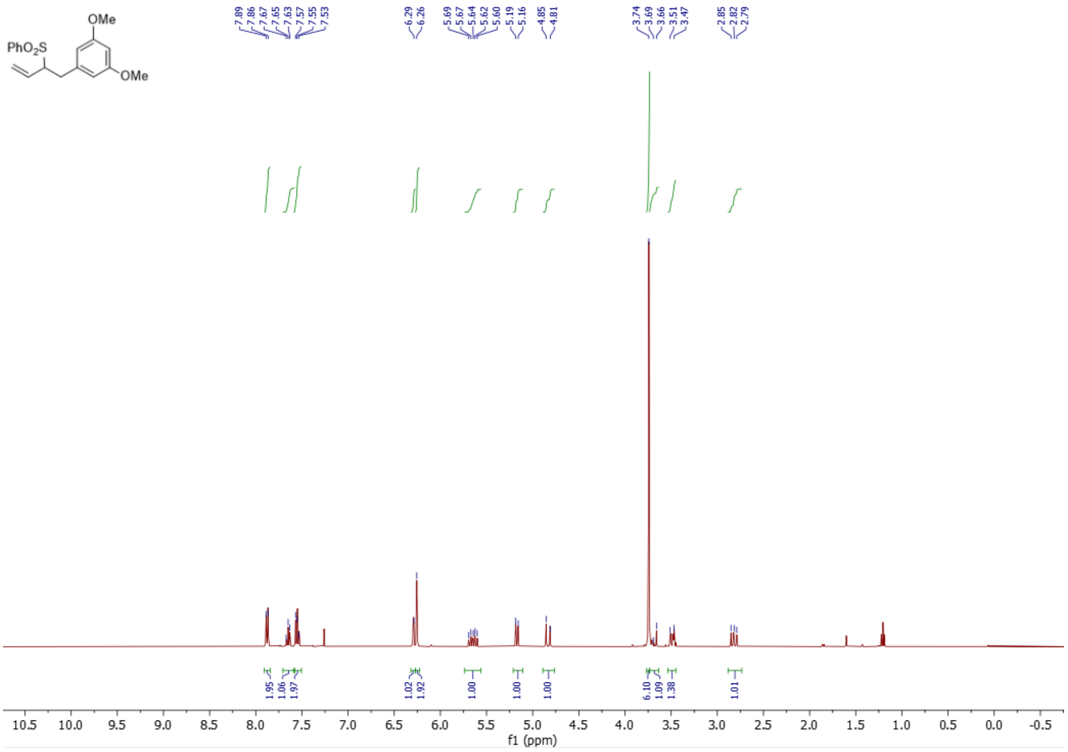

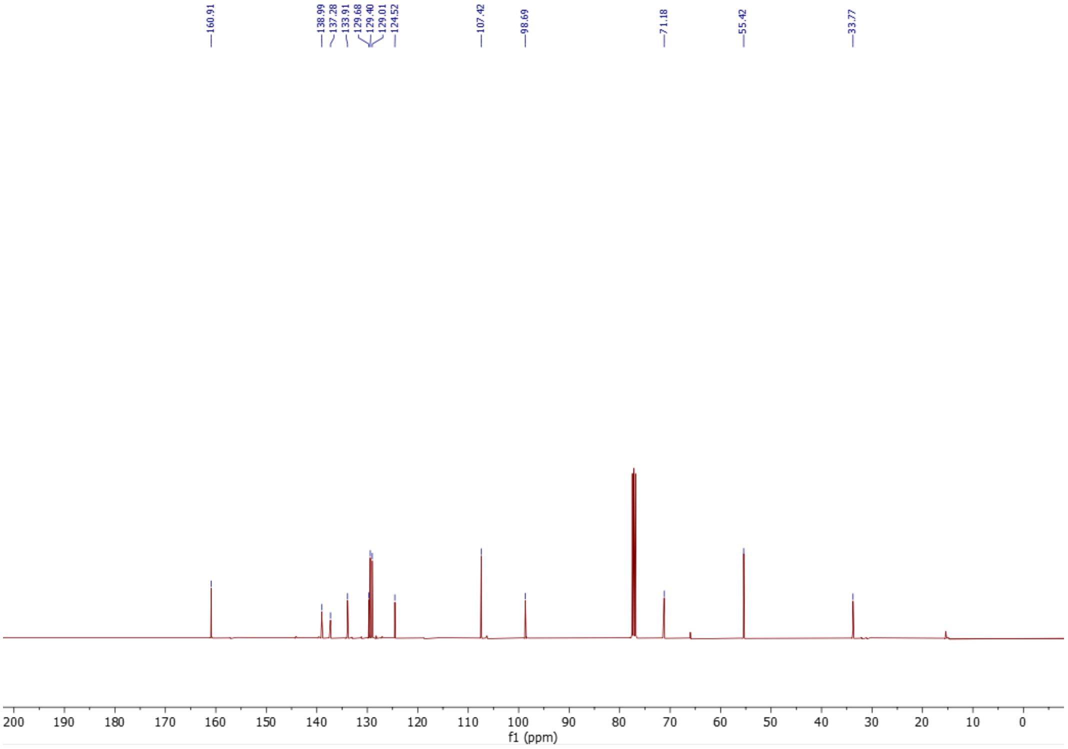

1-Bromo-2-[2-(phenylsulfonyl)but-3-en-1-yl]benzene (12h)

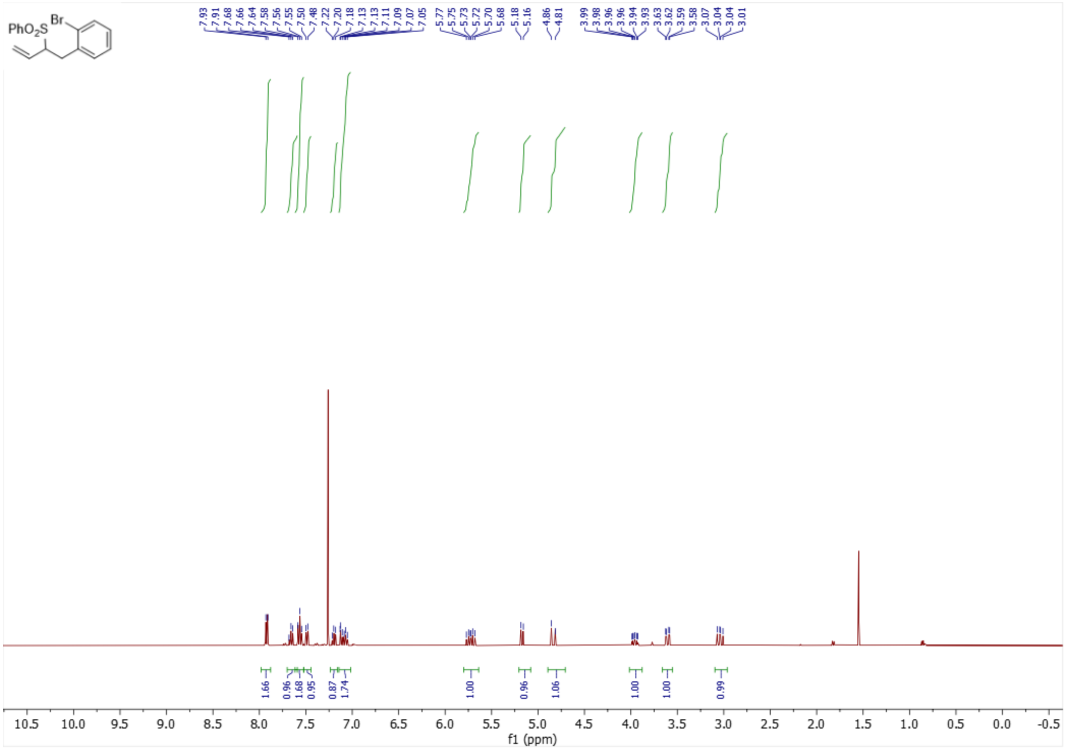

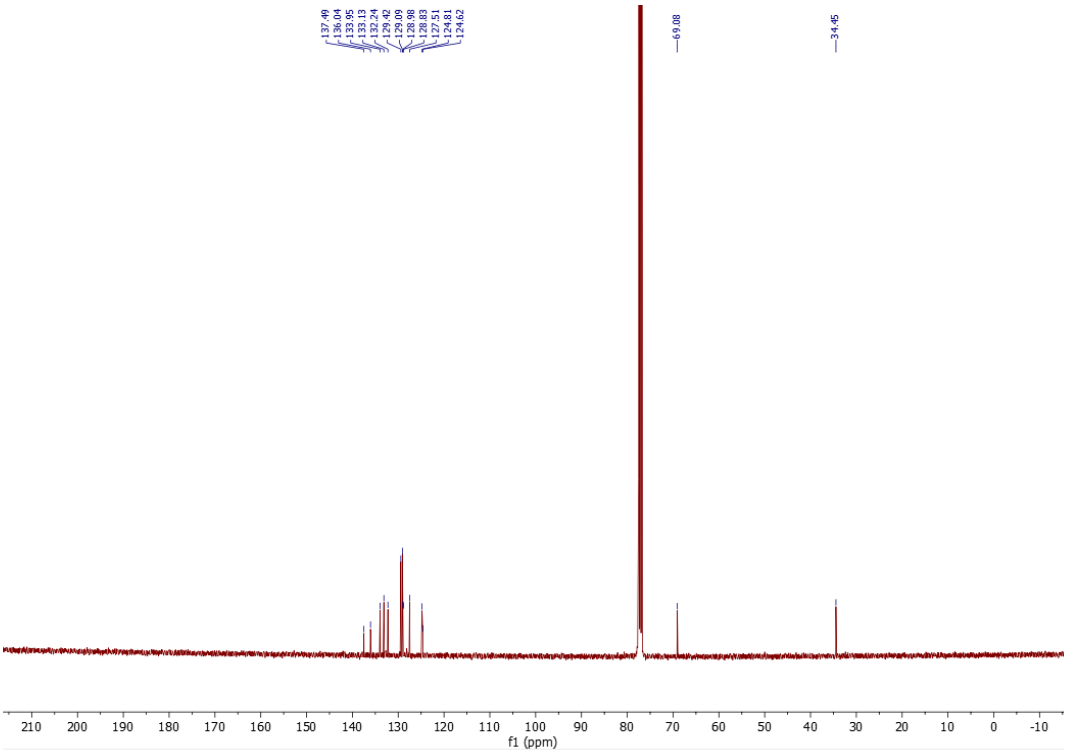

1-iodo-2-(2-(phenylsulfonyl)but-3-en-1-yl)benzene

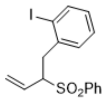

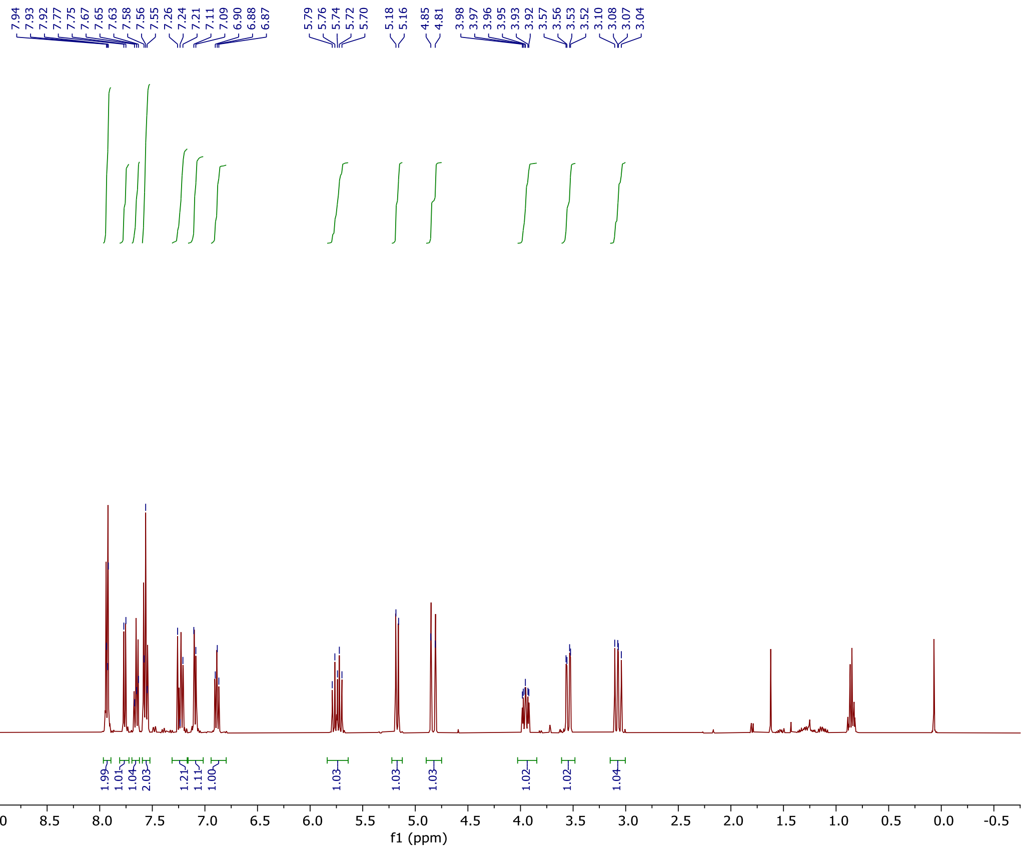

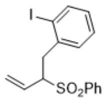

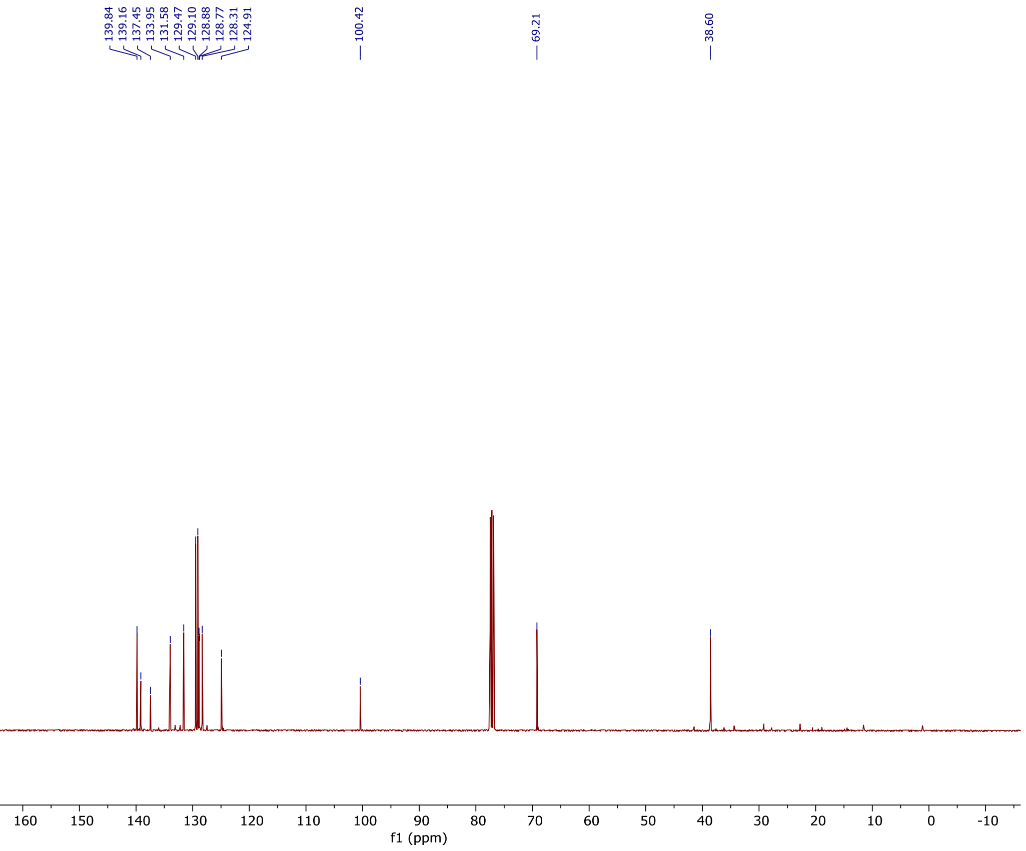

(*E*)-(Hepta-1,5-dien-3-ylsulfonyl)benzene (12j)

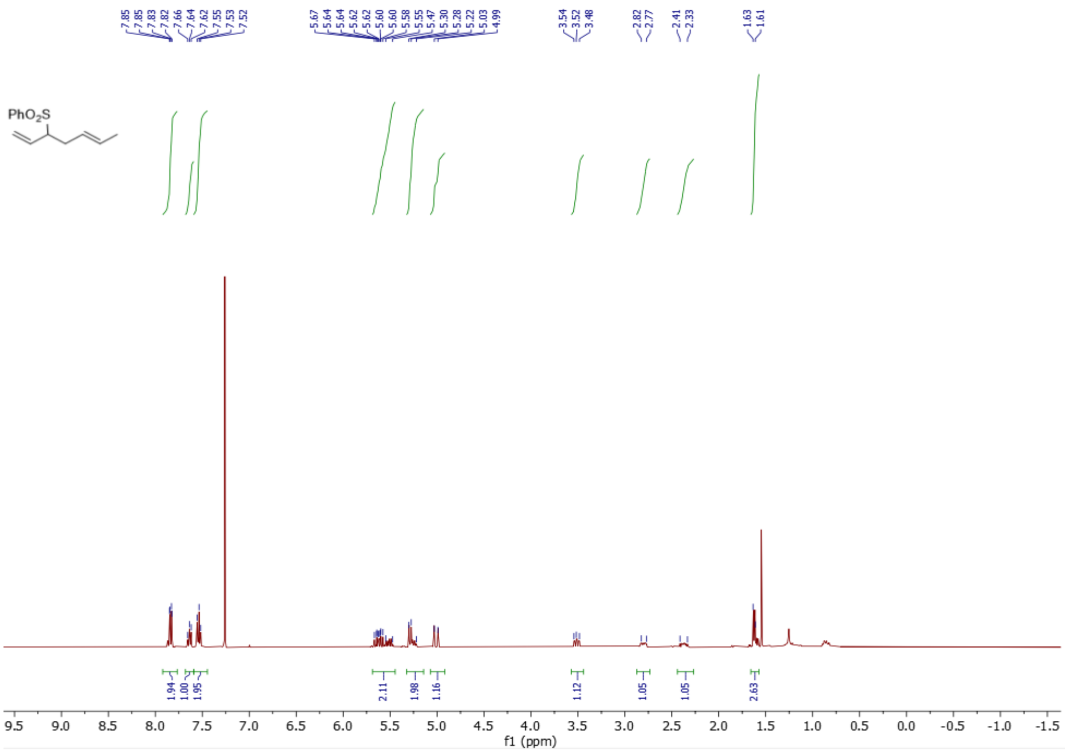

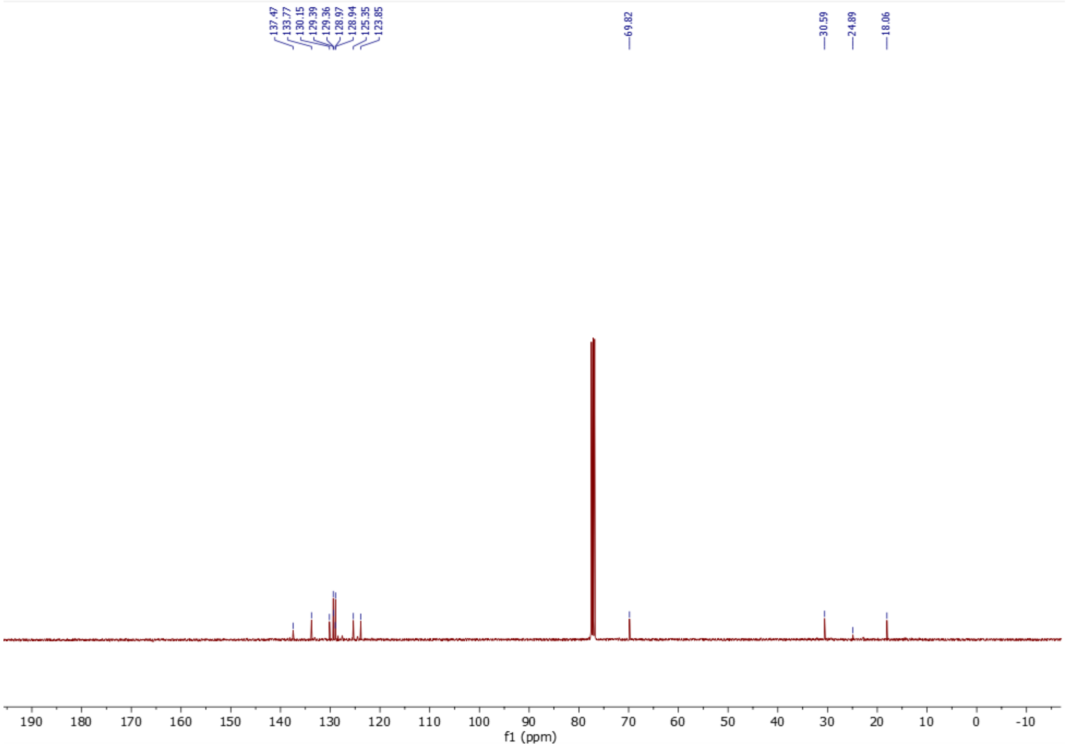

[(6-Methylhepta-1,5-dien-3-yl)sulfonyl]benzene (12k)

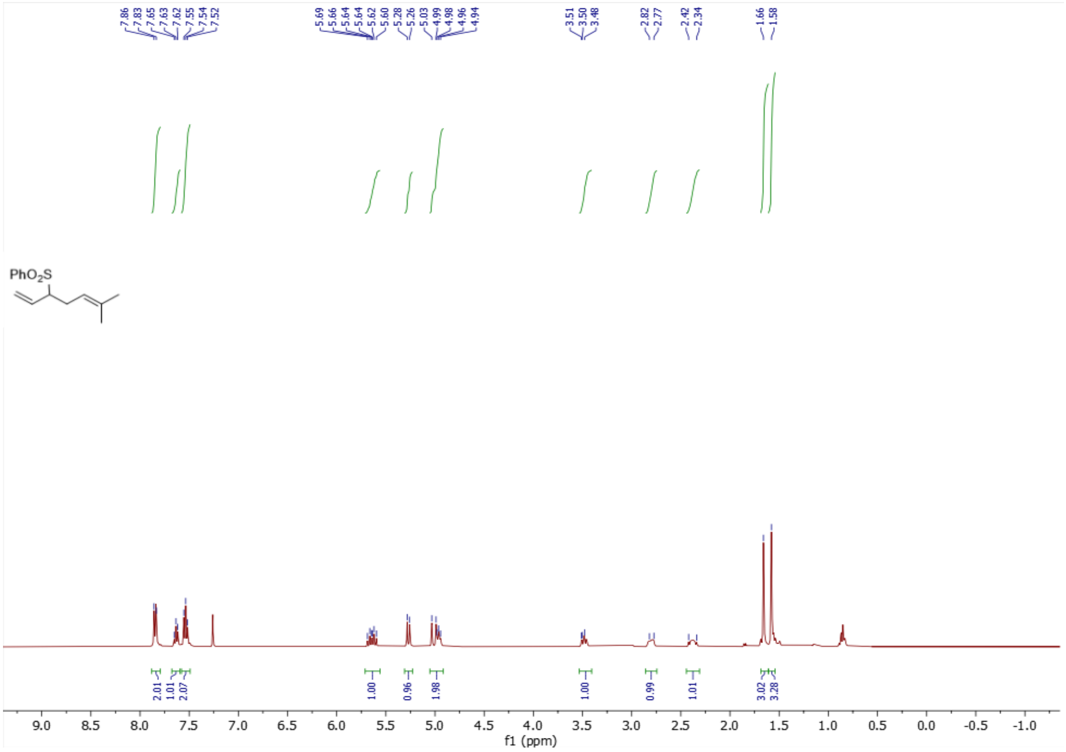

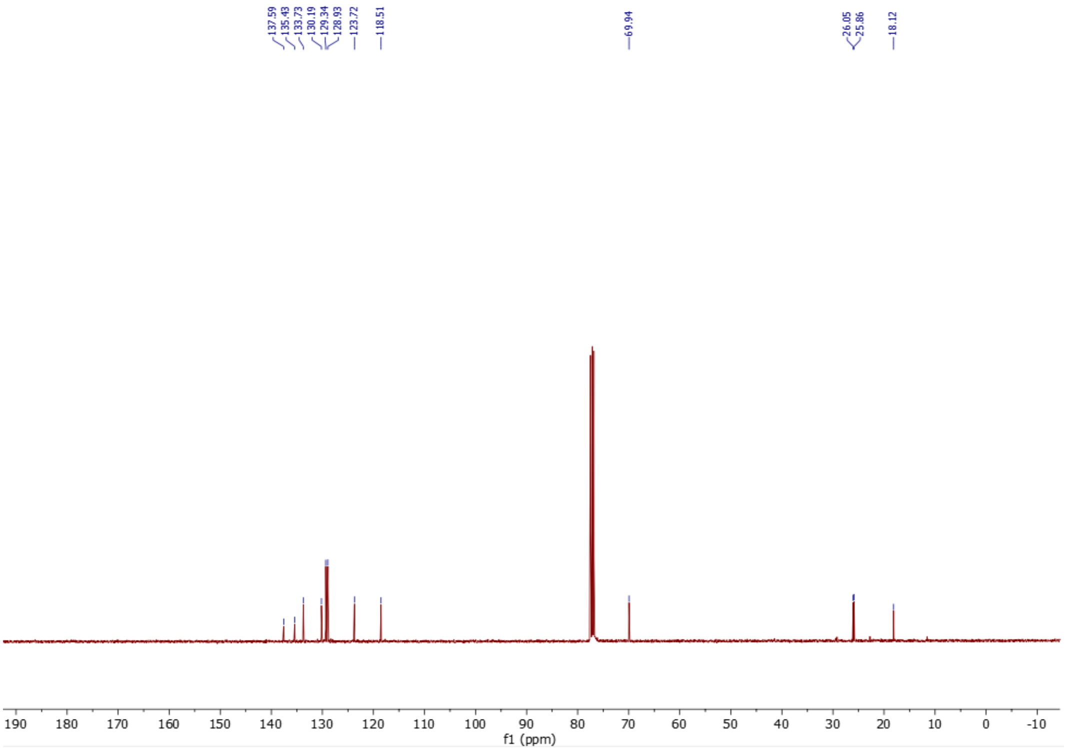

1-(1-(phenylsulfonyl)allyl)cyclopentan-1-ol

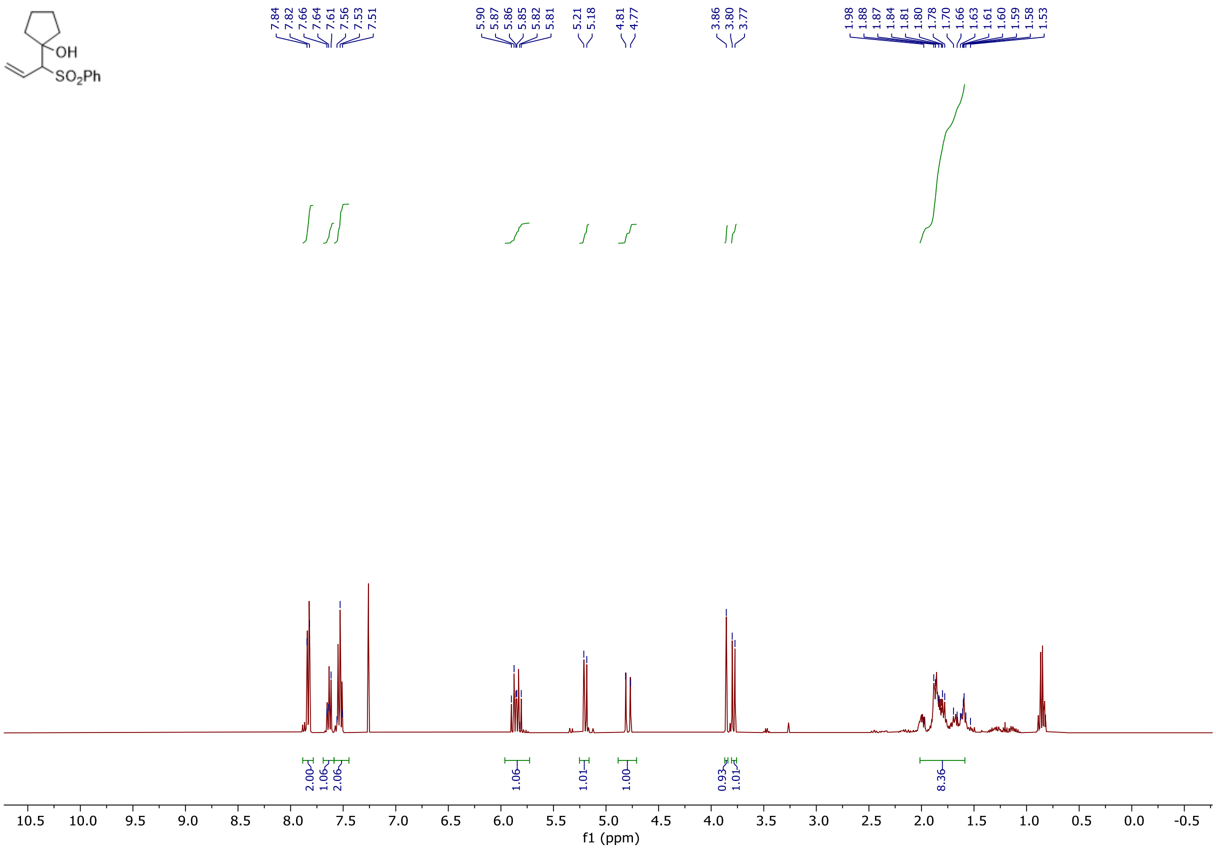

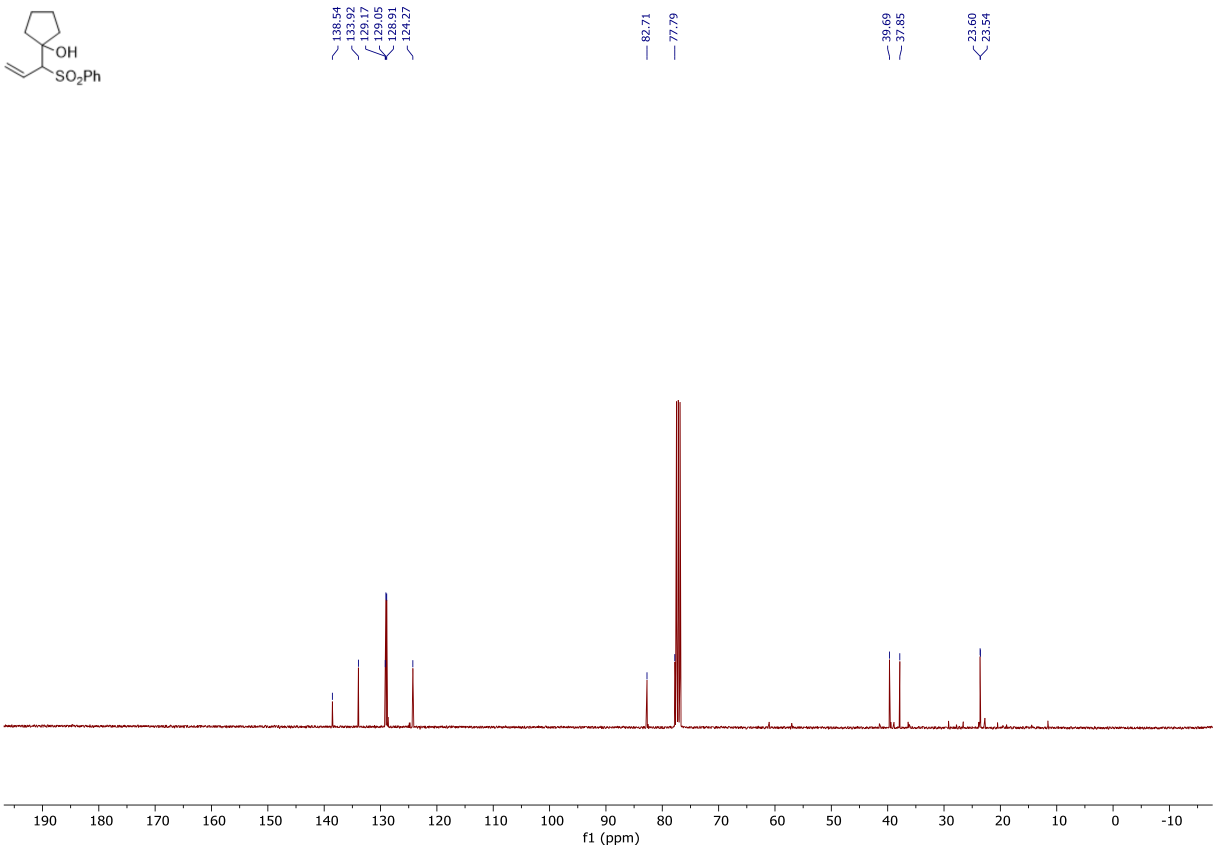

tert-butyldimethyl((1-(1-(phenylsulfonyl)allyl)cyclopentyl)oxy)silane

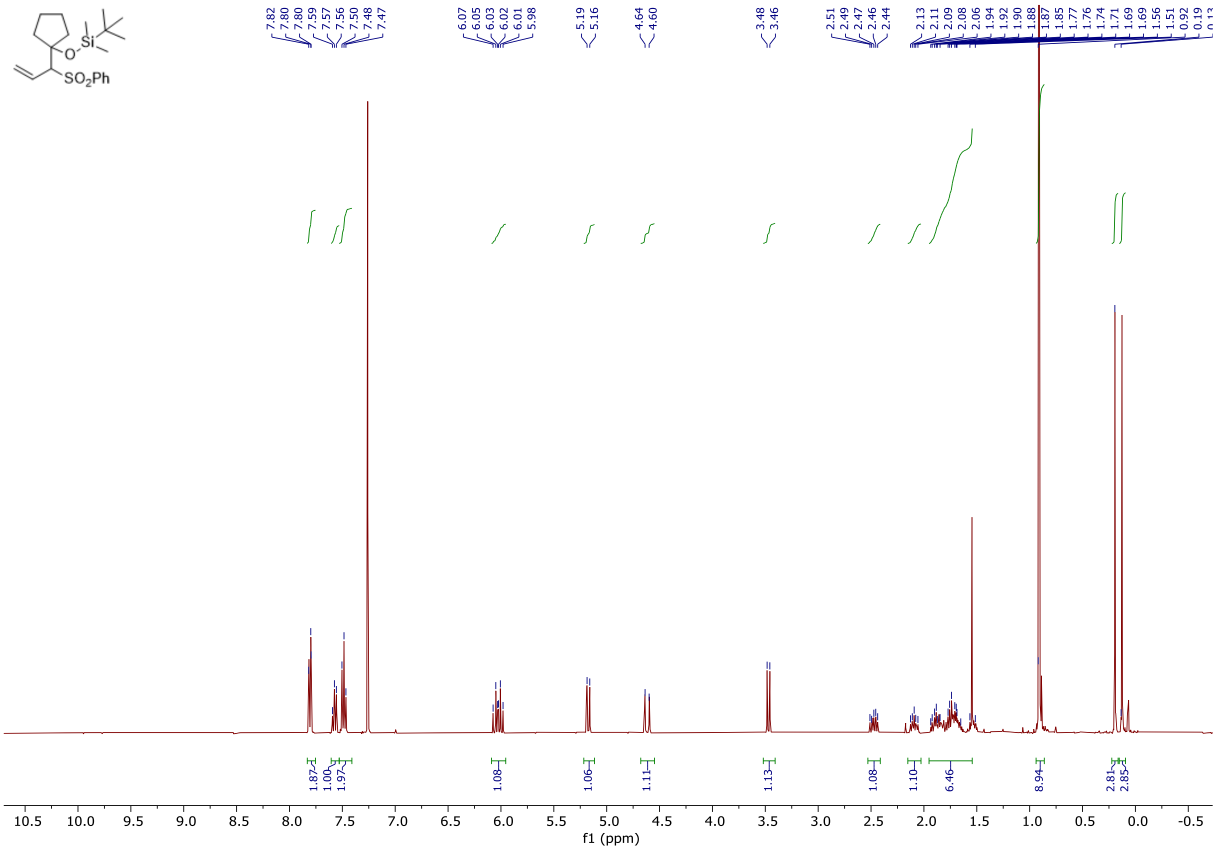

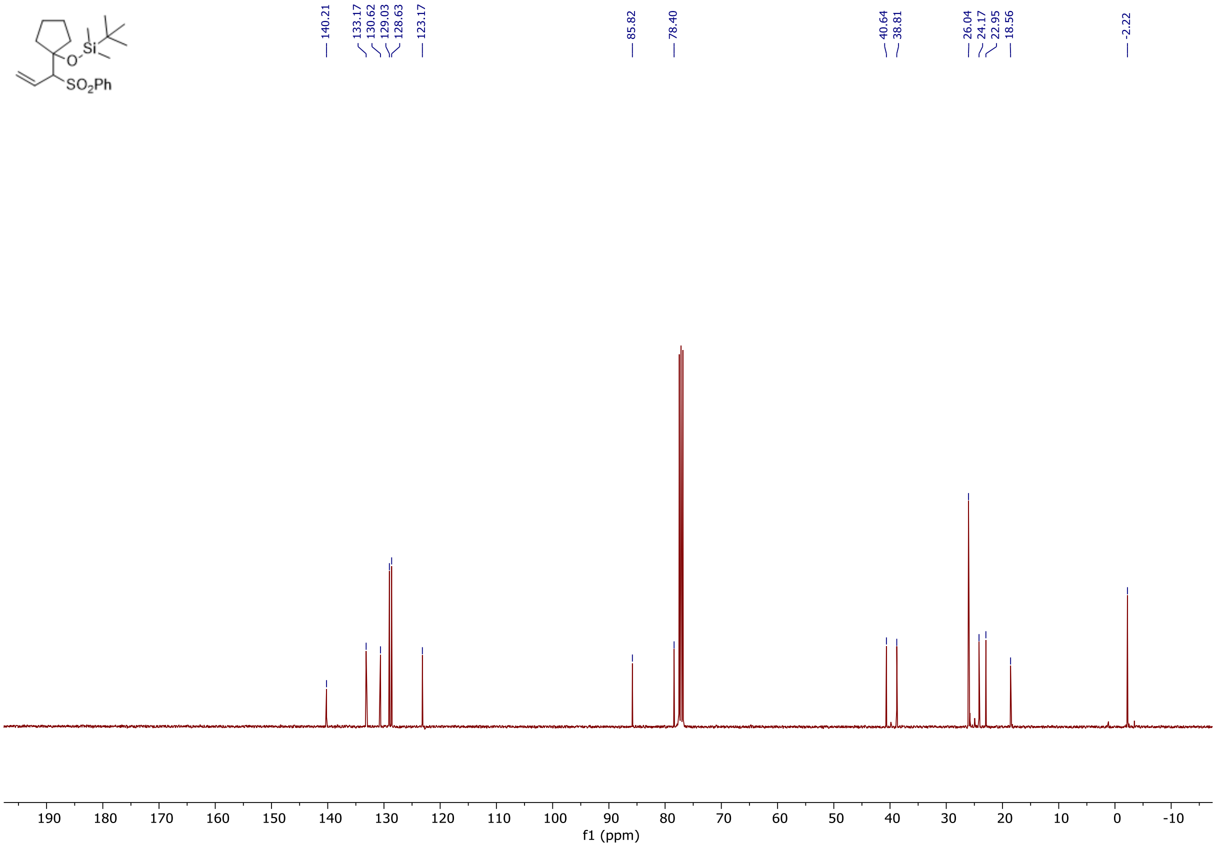

4,4,5,5-Tetramethyl-2-[3-(4-methylbenzyl)-4-(*p*-tolyl)but-2-en-1-yl]-1,3,2-dioxaborolane (13a)

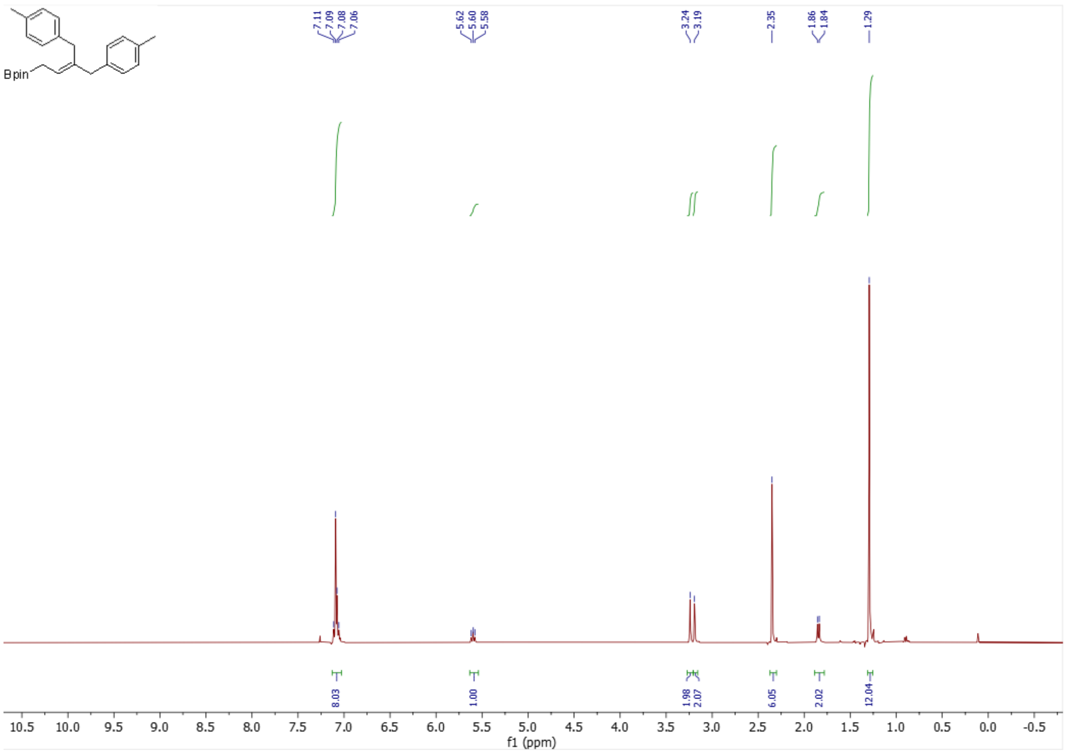

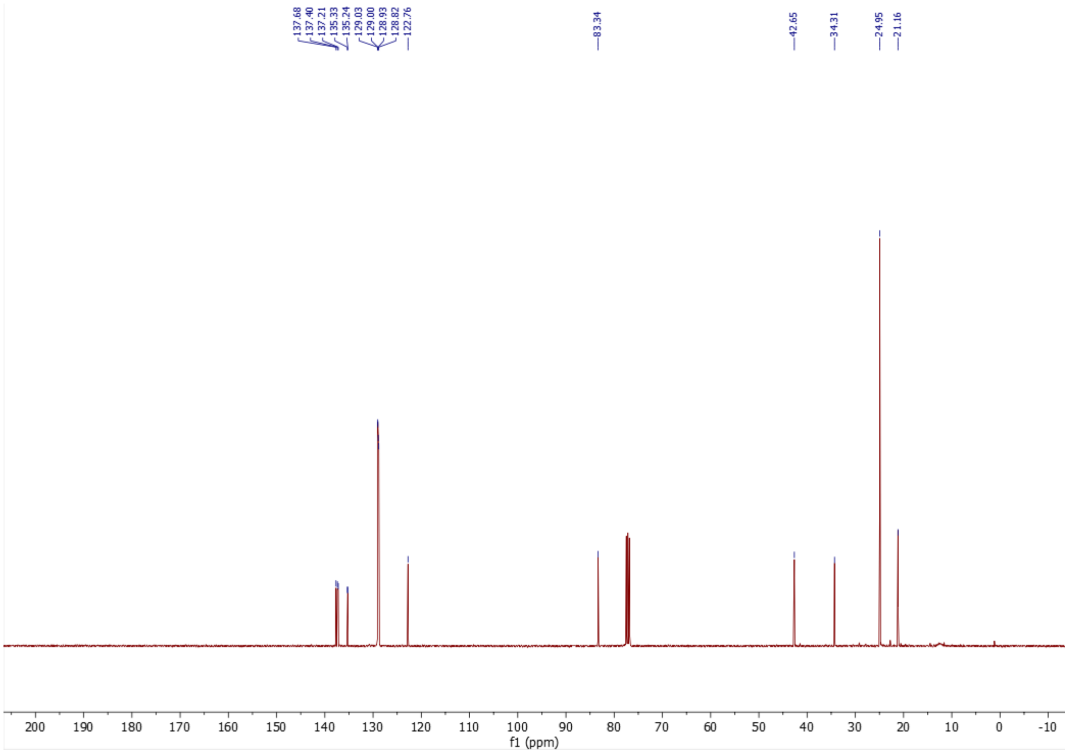

2-[3-(3,5-Dimethoxybenzyl)-4-(3,5-dimethoxyphenyl)but-2-en-1-yl)-4,4,5,5-tetramethyl-1,3,2-dioxaborolane (13b)

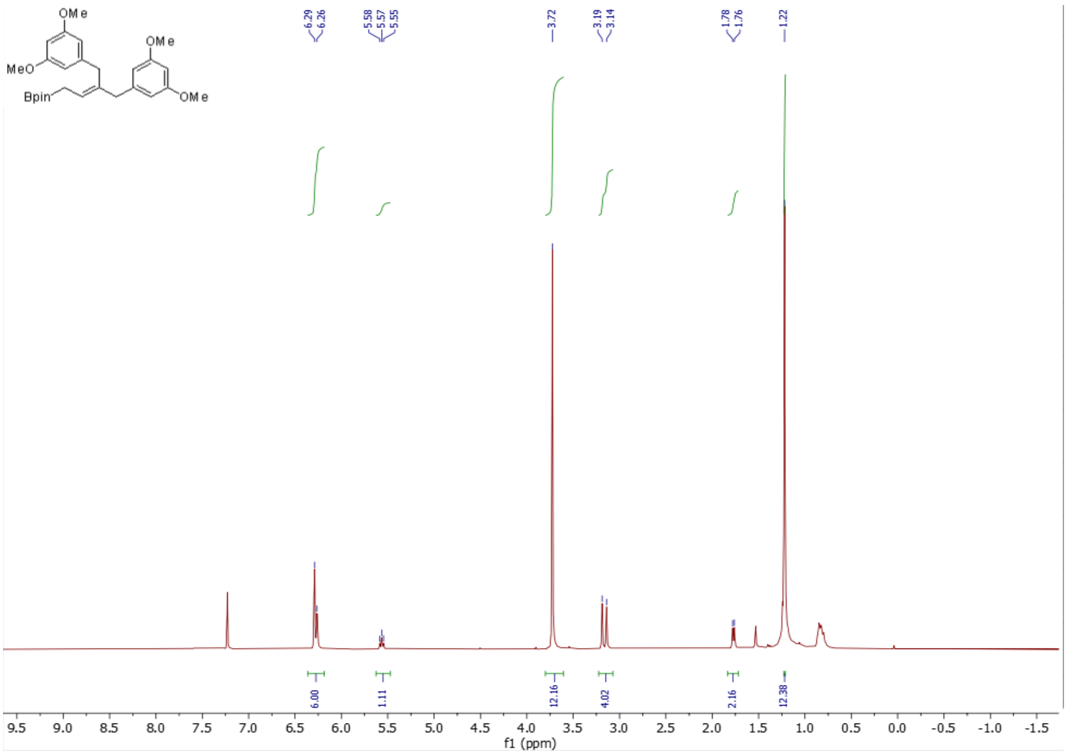

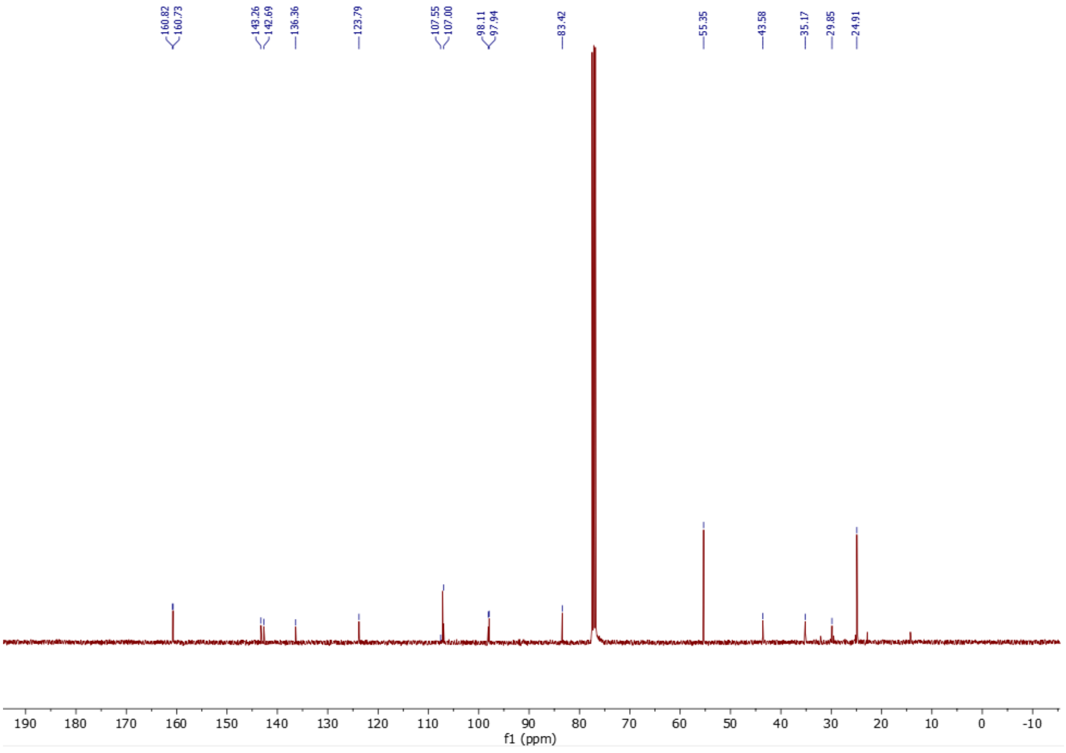

2-(3-Allylhexa-2,5-dien-1-yl)-4,4,5,5-tetramethyl-1,3,2-dioxaborolane (13c)

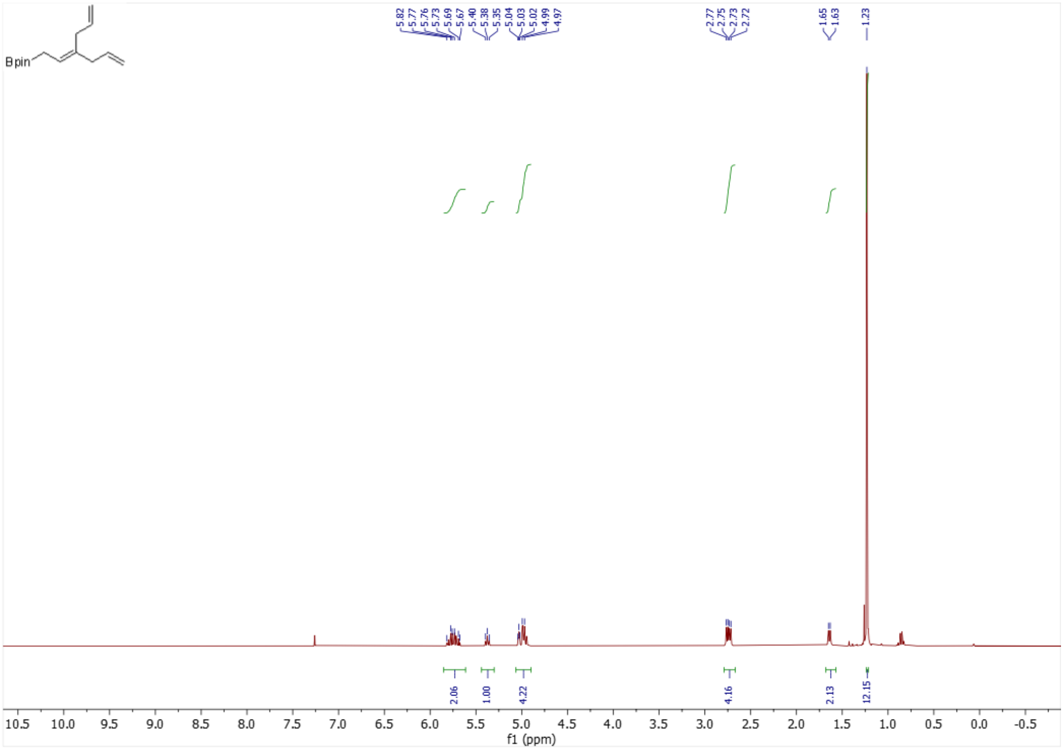

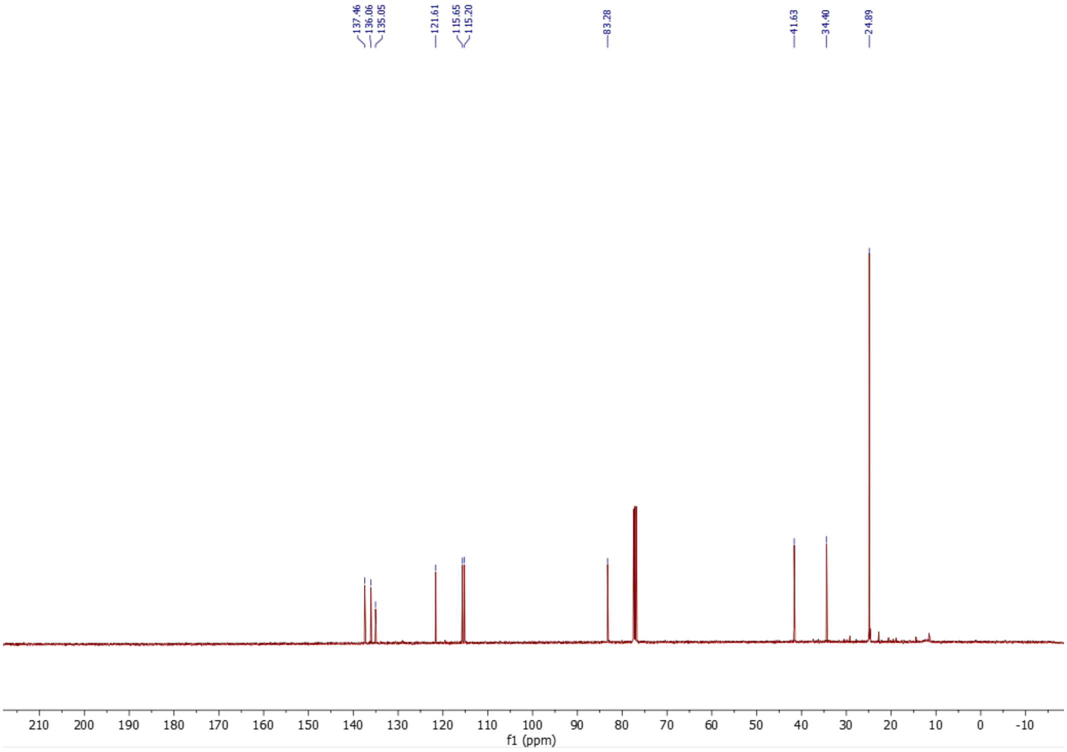

2-[(*E*)-3-Cinnamyl-6-phenylhexa-2,5-dien-1-yl]-4,4,5,5-tetramethyl-1,3,2-dioxaborolane (13d)

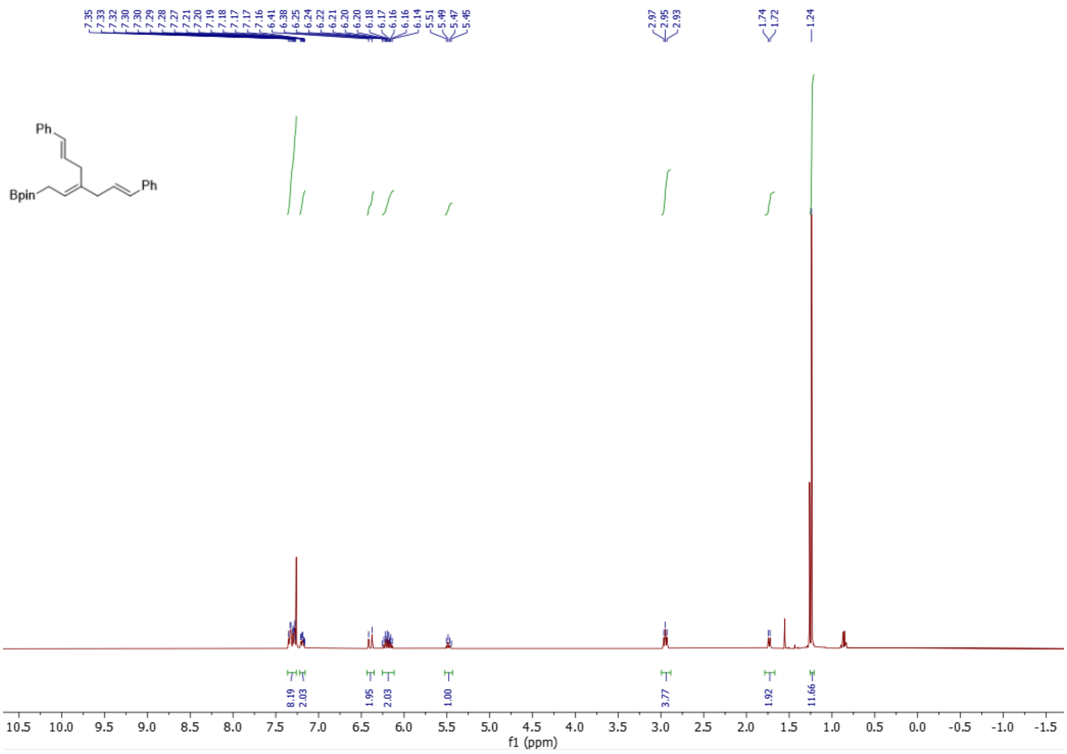

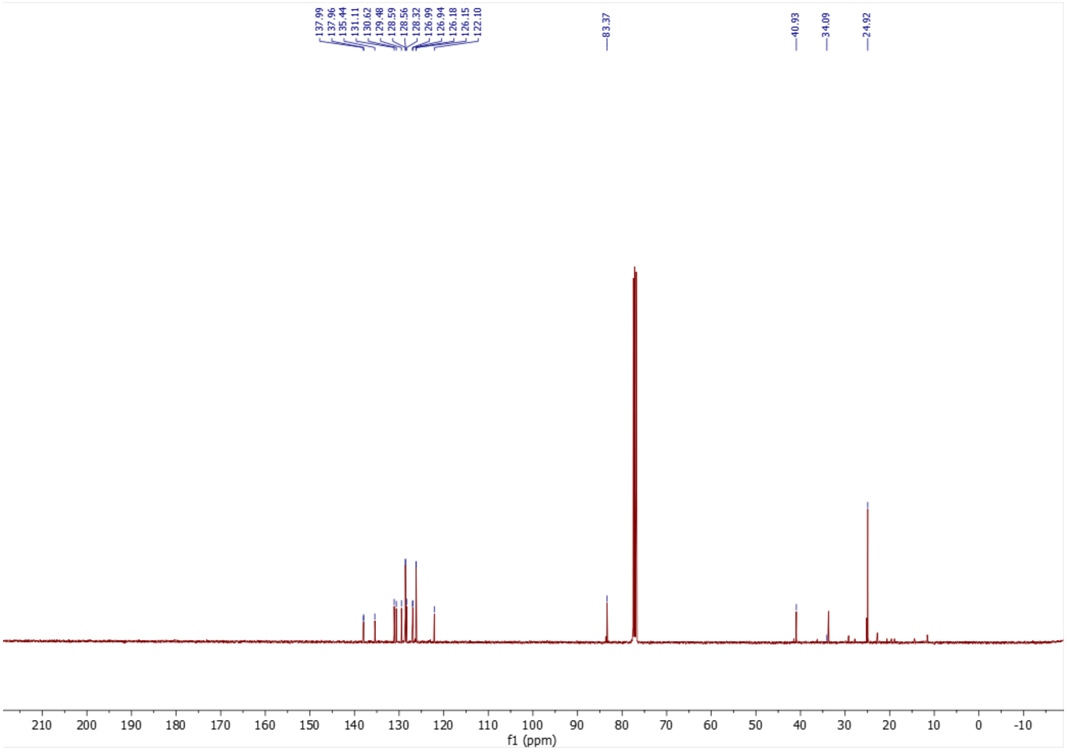

4,4,5,5-Tetramethyl-2-[6-methyl-3-(3-methylbut-2-en-1-yl)hepta-2,5-dien-1-yl]-1,3,2-dioxaborolane (13e)

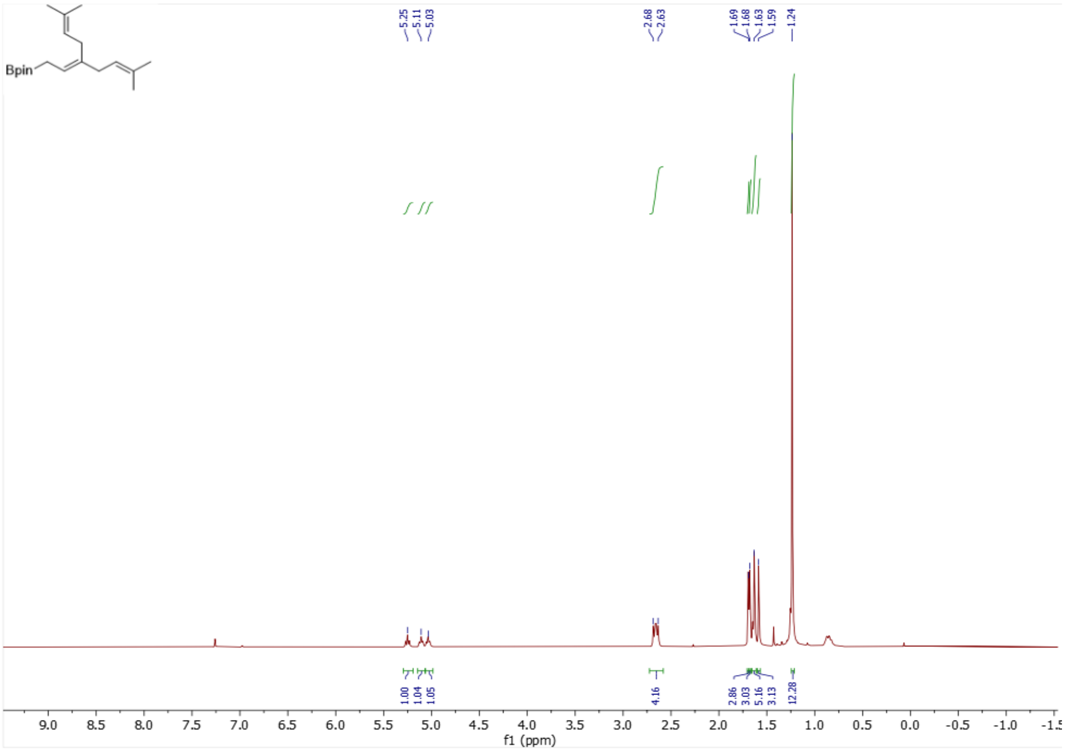

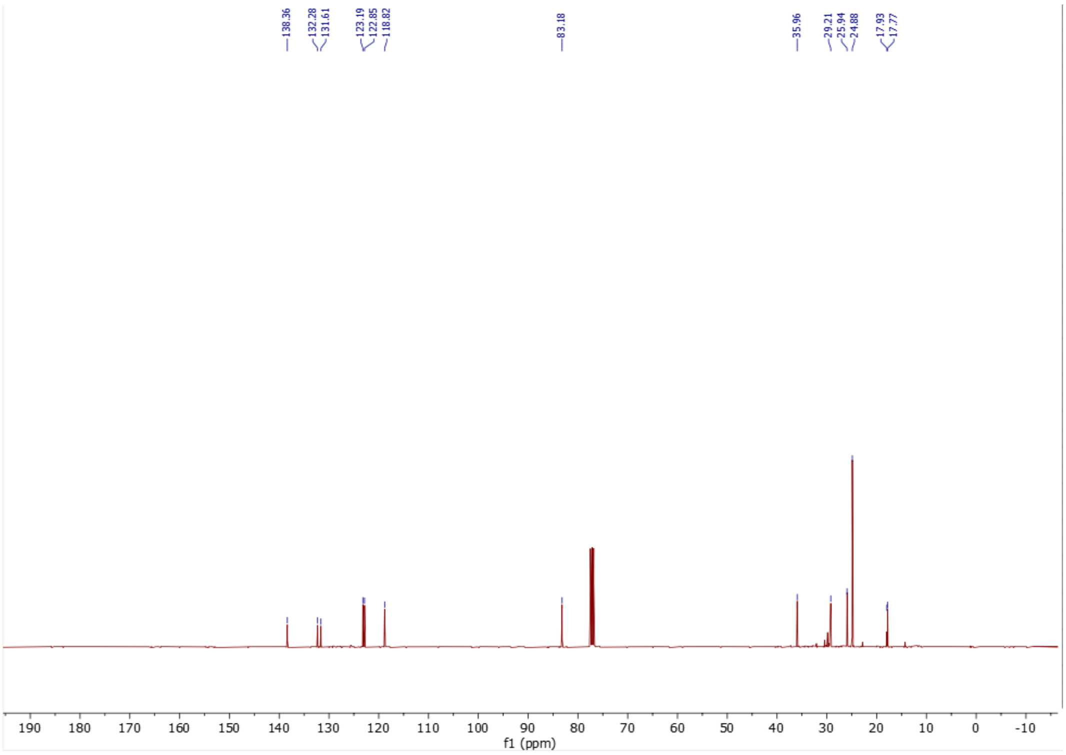

(*E*)-4,4,5,5-Tetramethyl-2-[4-(*p*-tolyl)but-2-en-1-yl]-1,3,2-dioxaborolane (13f)

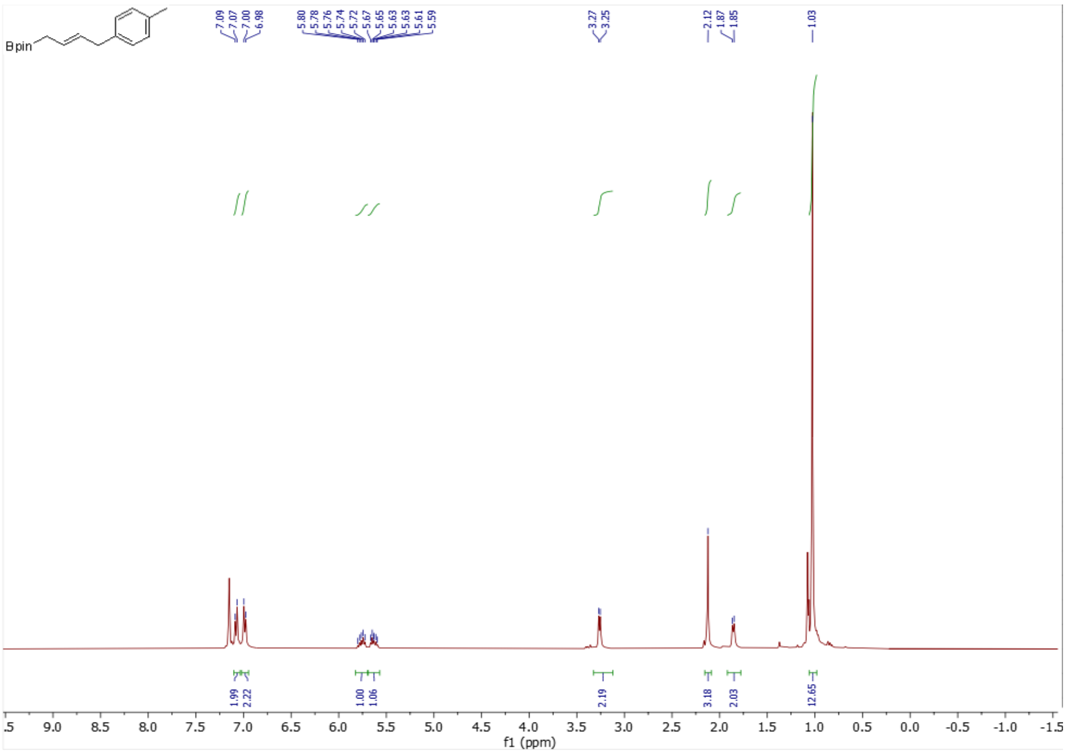

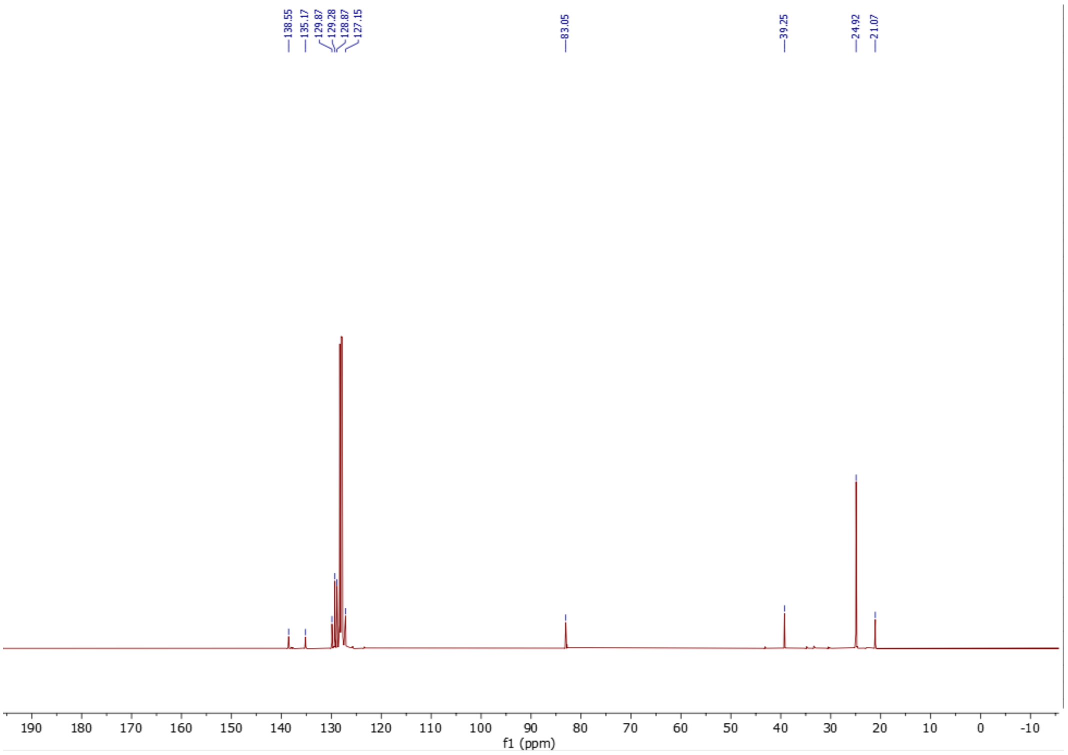

(*E*)-2-[4-(3,5-Dimethoxyphenyl)but-2-en-1-yl]-4,4,5,5-tetramethyl-1,3,2-dioxaborolane (13g)

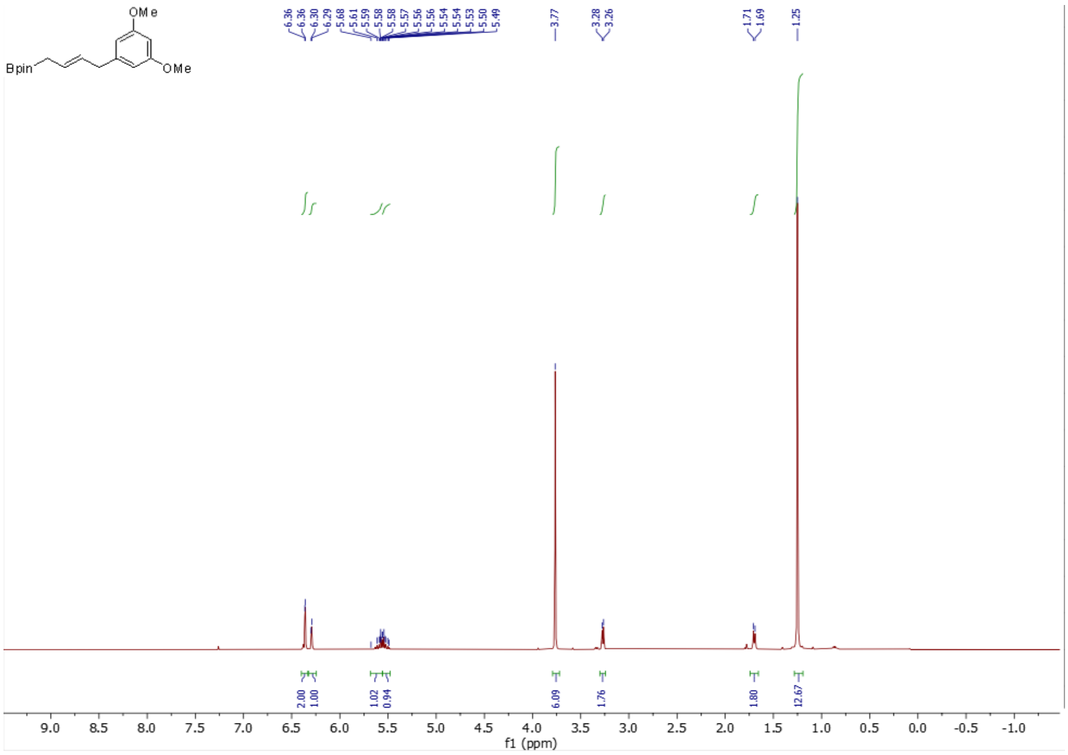

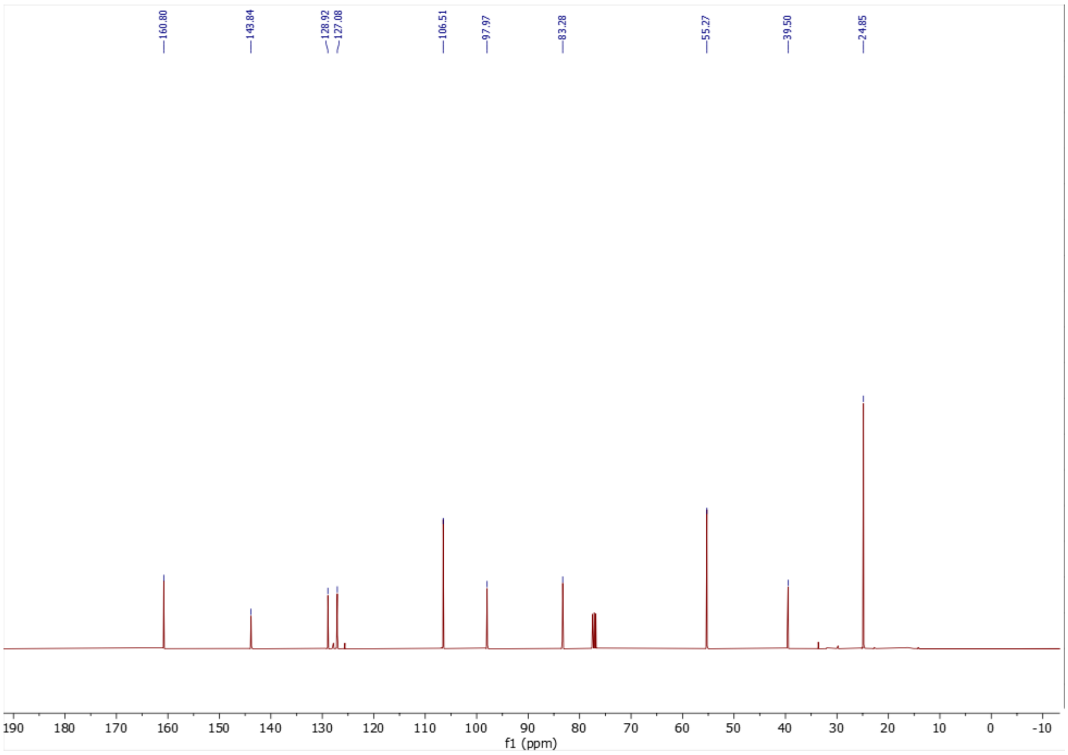

(*E*)-2-[4-(2-Bromophenyl)but-2-en-1-yl]-4,4,5,5-tetramethyl-1,3,2-dioxaborolane (13h)

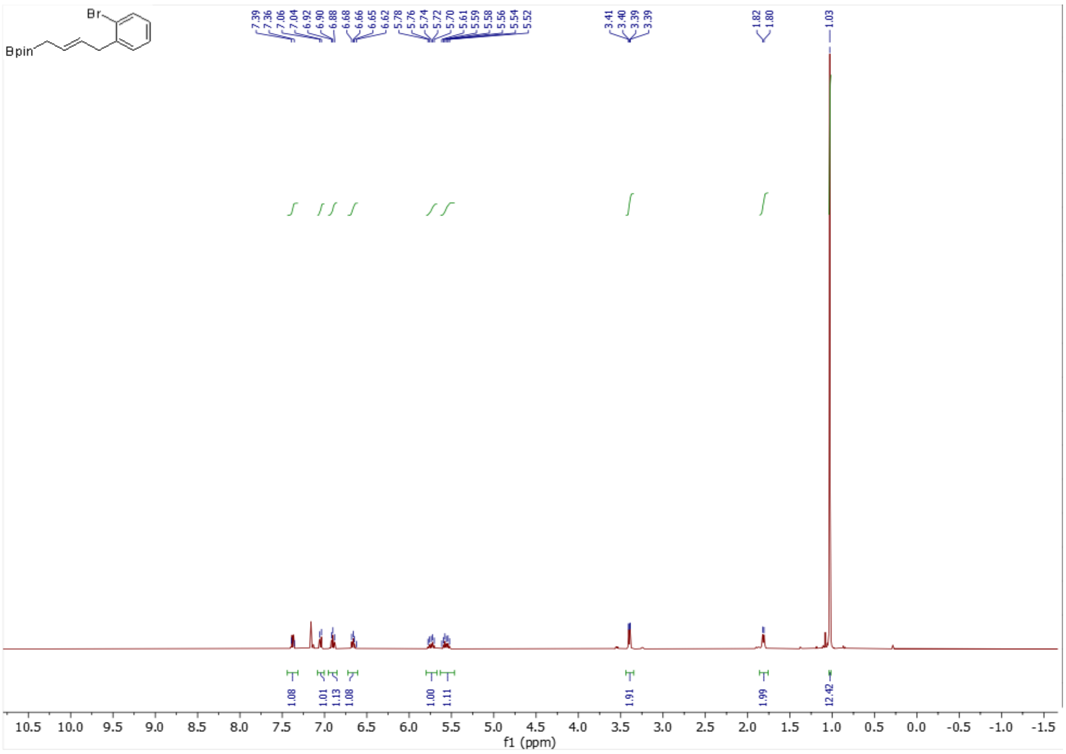

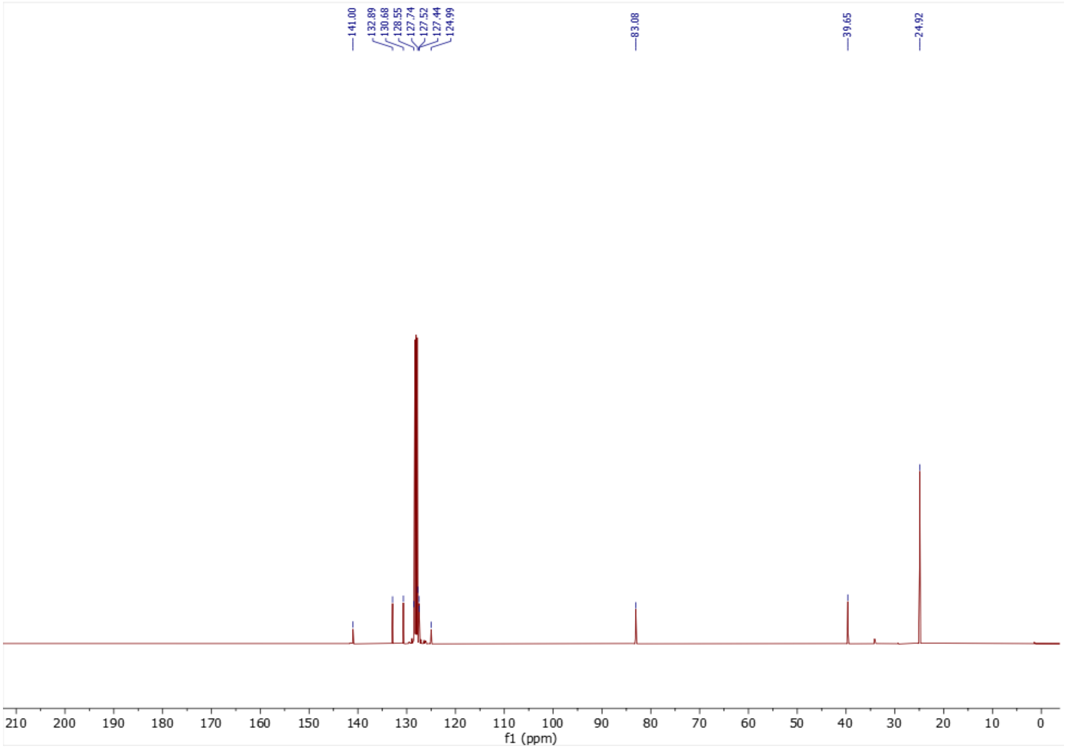

(E)-2-(4-(2-iodophenyl)but-2-en-1-yl)-4,4,5,5-tetramethyl-1,3,2-dioxaborolane

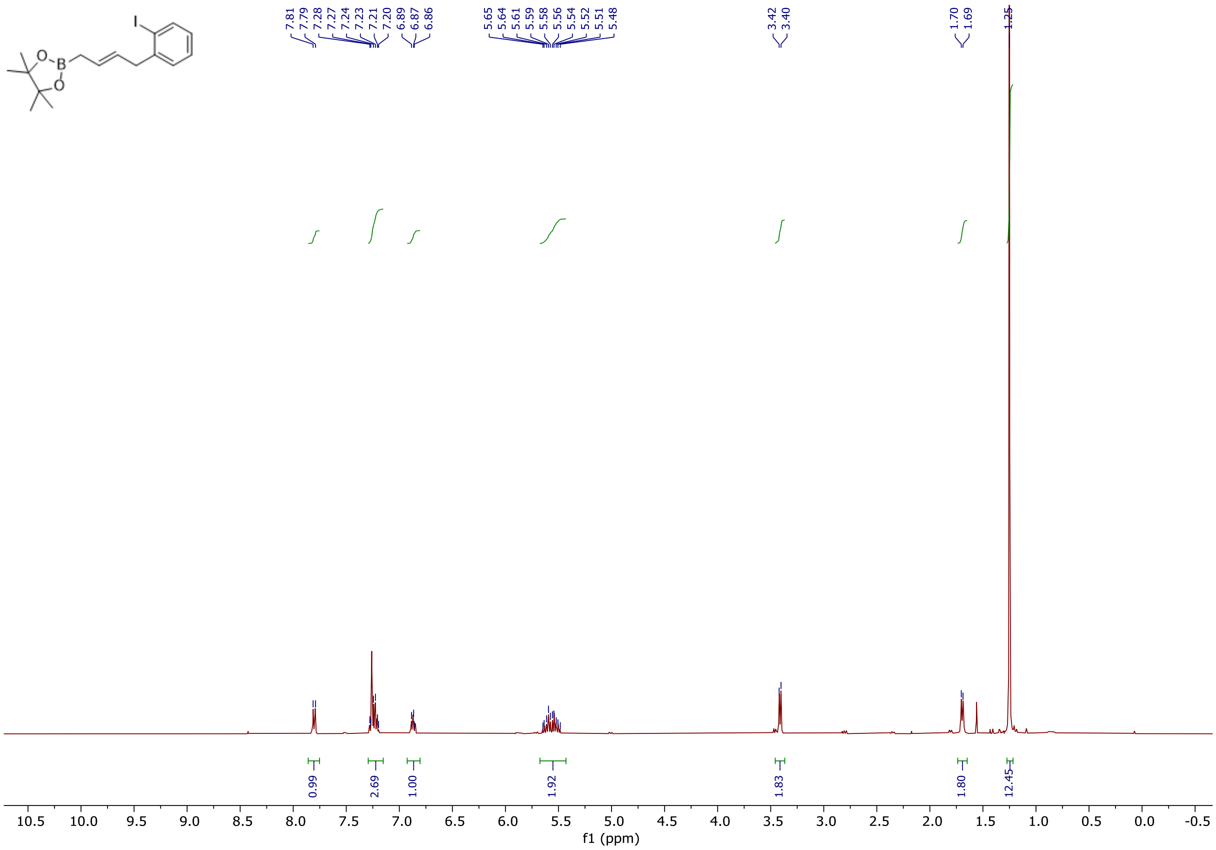

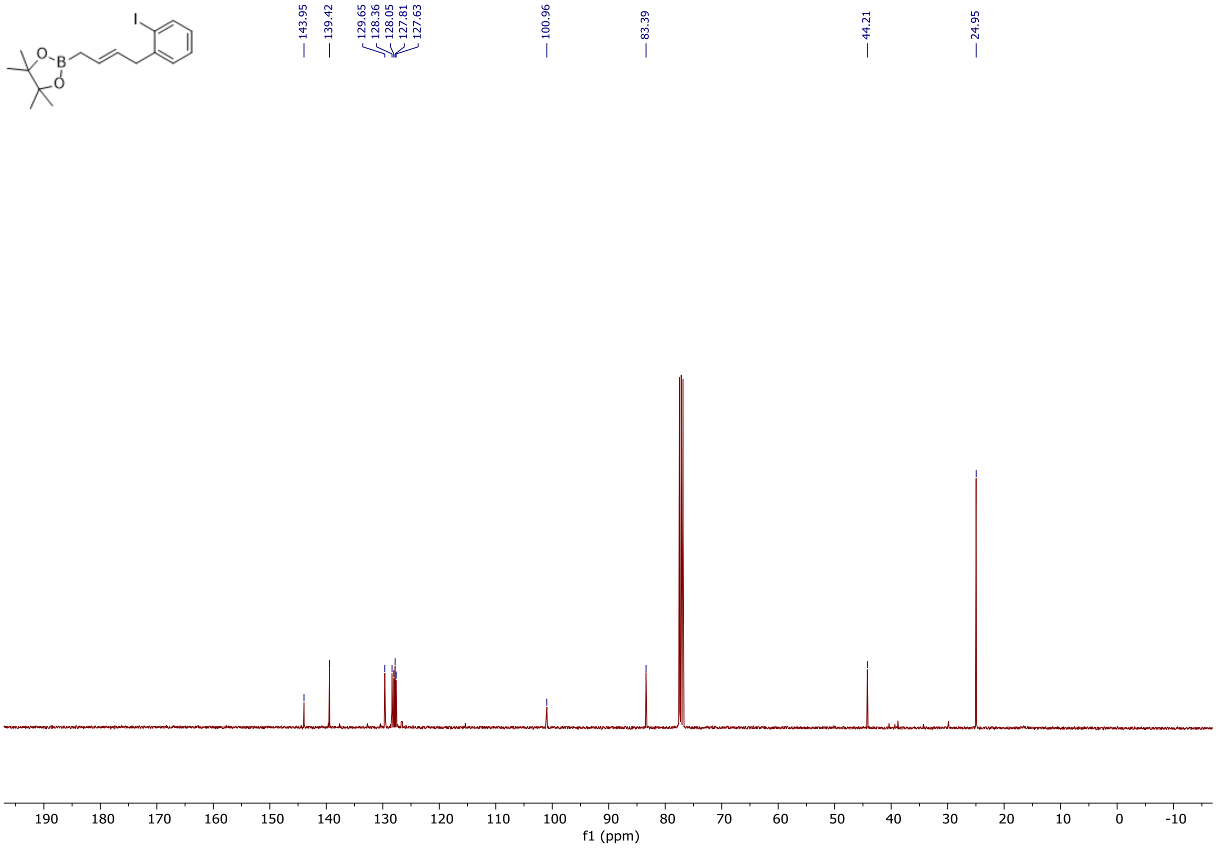

2-[(2*E*,5*E*)-Hepta-2,5-dien-1-yl]-4,4,5,5-tetramethyl-1,3,2-dioxaborolane (13j)

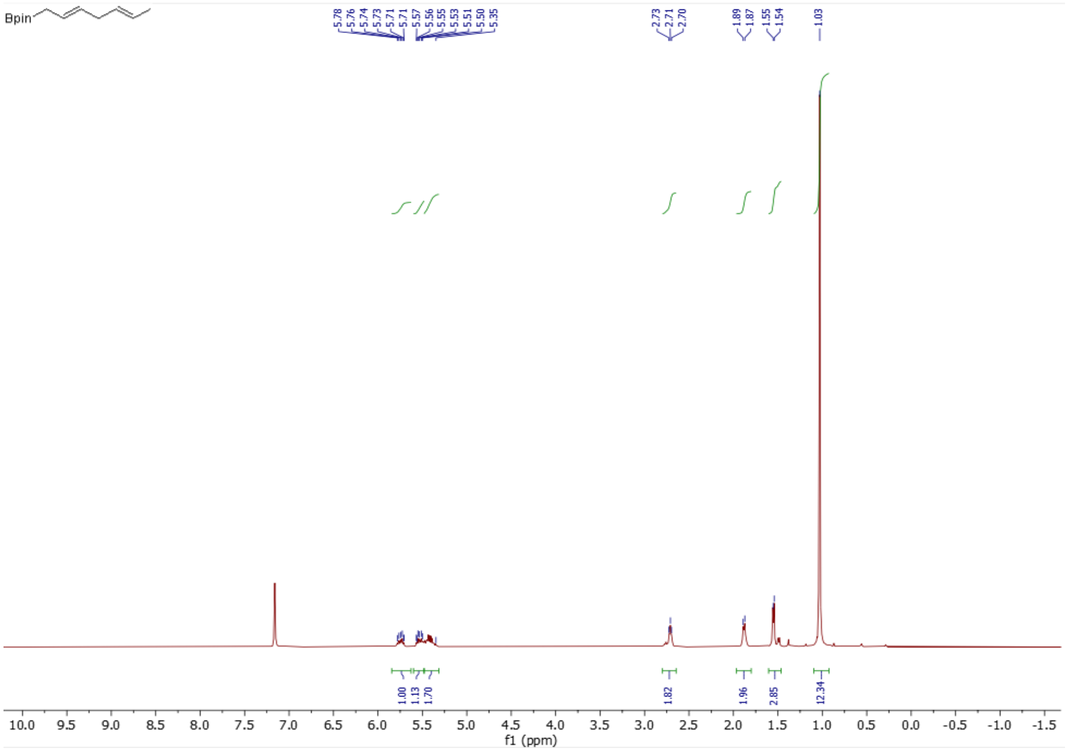

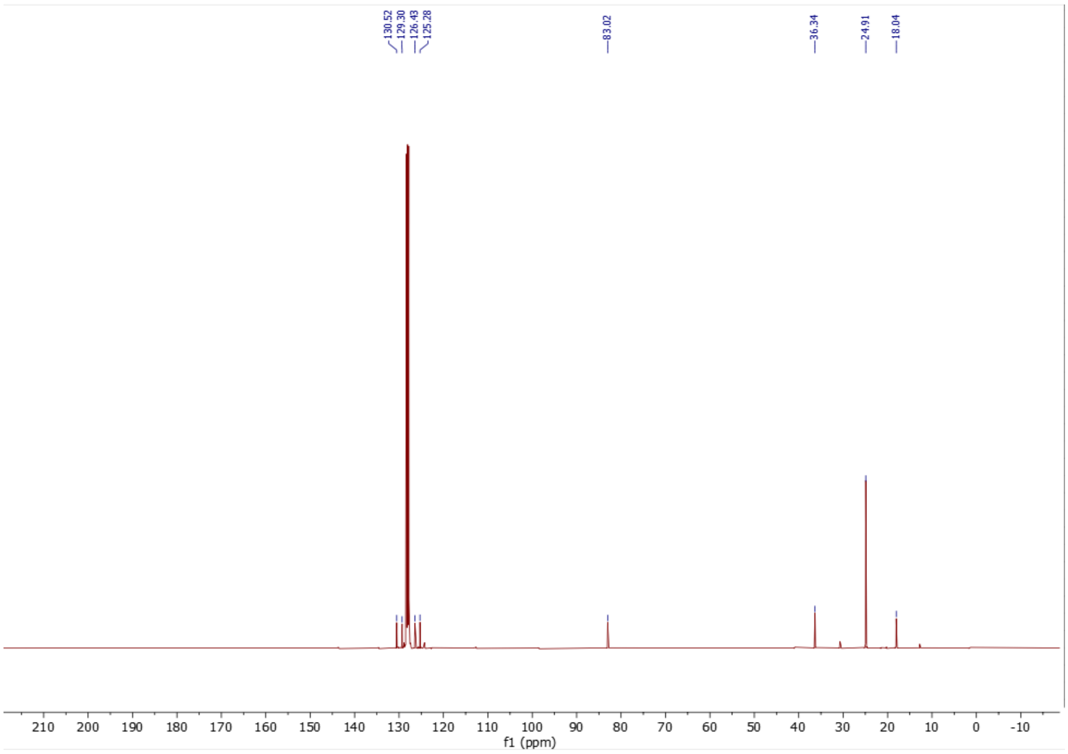

(*E*)-4,4,5,5-Tetramethyl-2-(6-methylhepta-2,5-dien-1-yl)-1,3,2-dioxaborolane (13k)

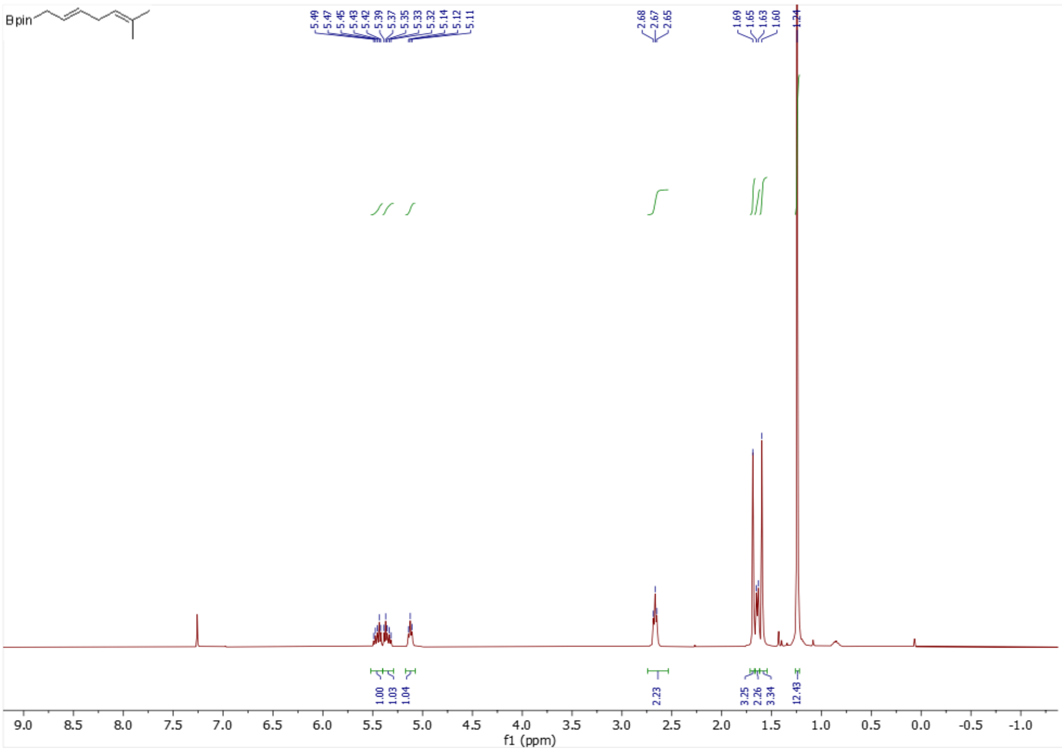

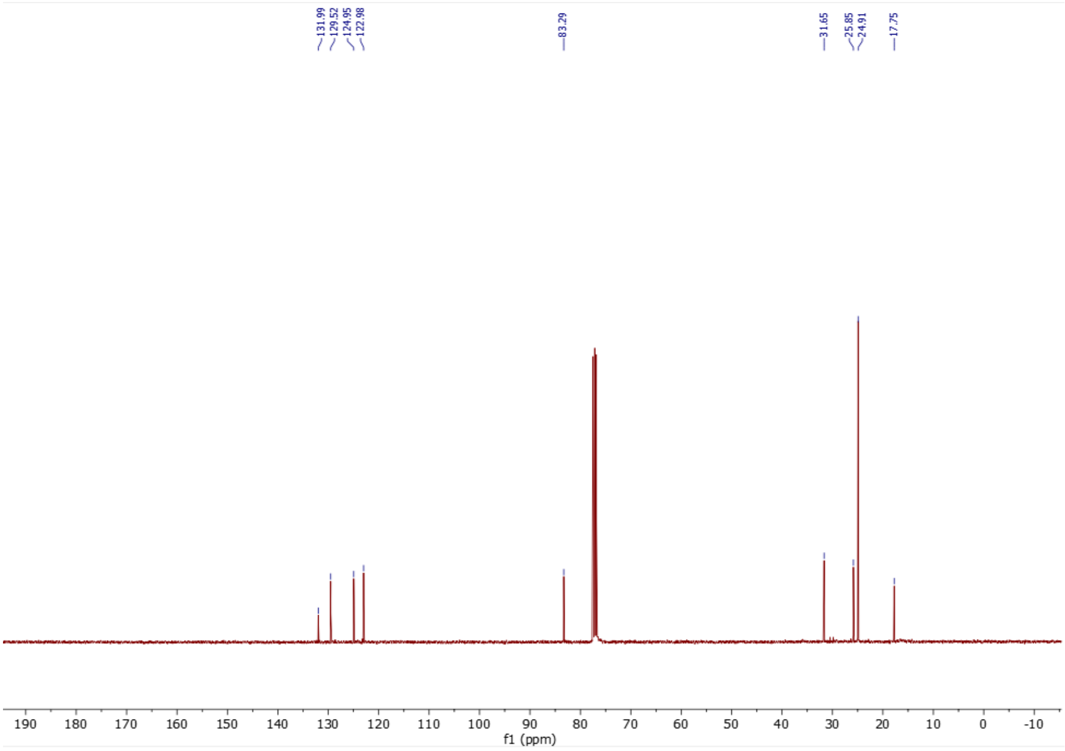

(E)-tert-butyldimethyl((1-(3-(4,4,5,5-tetramethyl-1,3,2-dioxaborolan-2-yl)prop-1-en-1-yl)cyclopentyl)oxy)silane

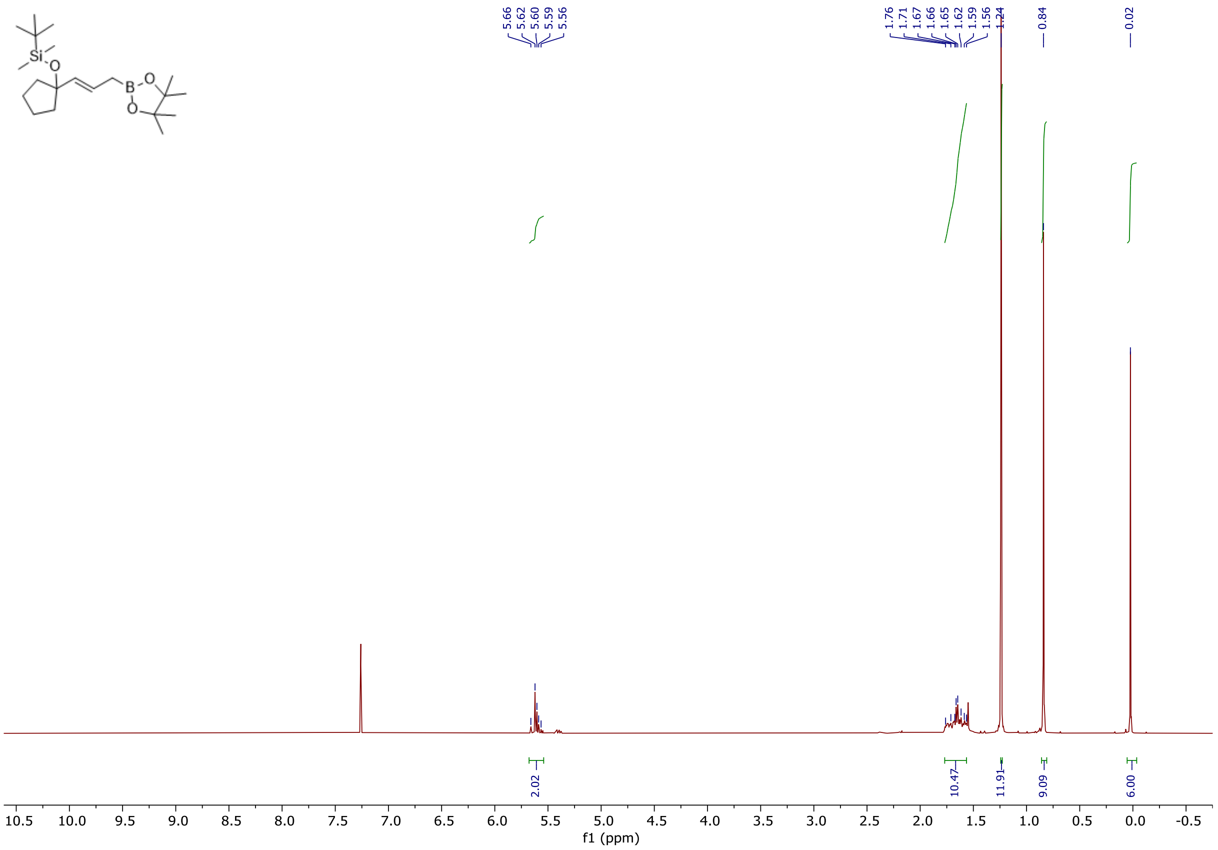

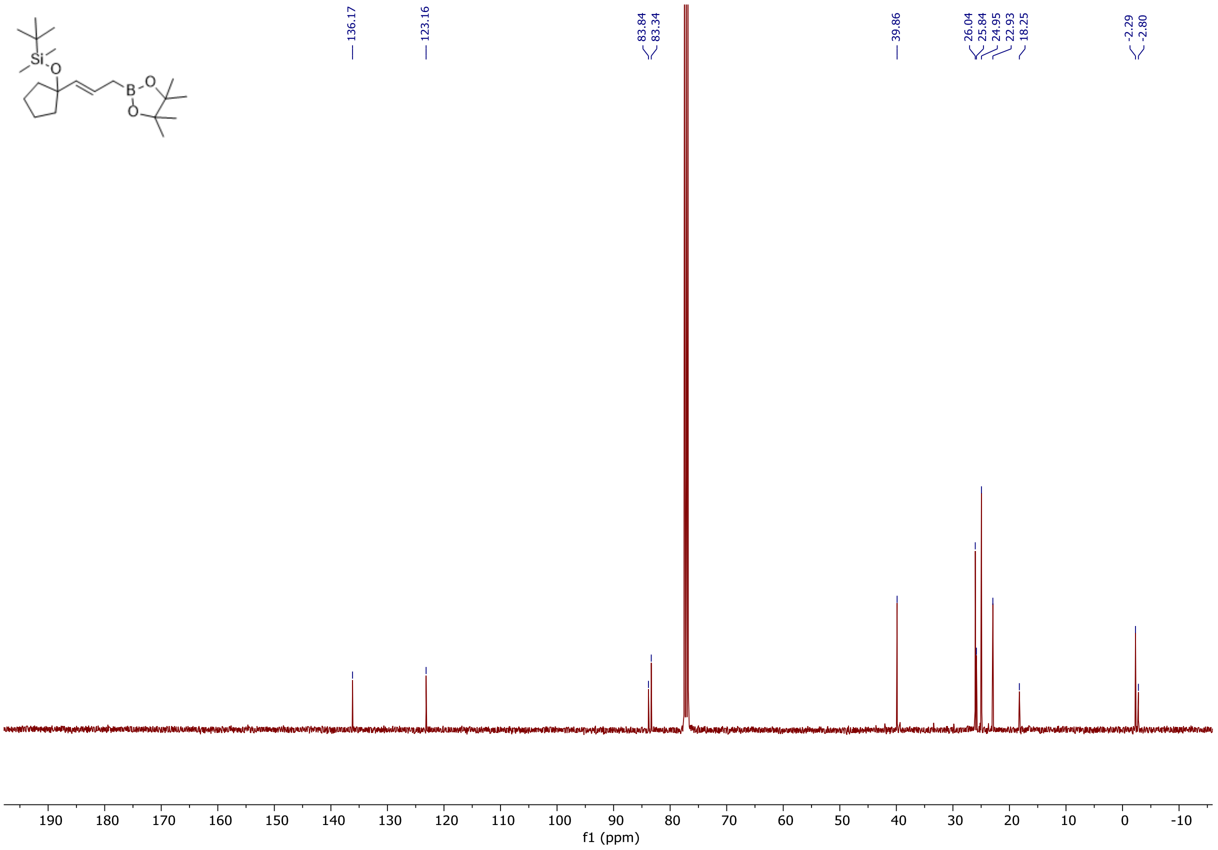

4. X-Ray crystallography of lithium(tetrahydrate) bis(pinacolato)borate

Suitable single crystals were grown from an MeOH/CH_2Cl_2 solution left for slow evaporation, selected and mounted on a STOE STADIVARI diffractometer. The crystals were kept at 150K during data collection.

Using Olex2,[2] the structure was solved with the SHELXT[3] structure solution program using Intrinsic Phasing and refined with the SHELXL[4] refinement package using Least Squares minimisation. Pictures of the compound structure were obtained using the MERCURY software. During the refinement steps, all atoms, except hydrogen atoms, were refined anisotropically. The positions of the hydrogen atoms were determined geometrically.

Lithium(tetrahydrate) bis(pinacolato)borate

The crystal structure of the compound was deposited on the Cambridge Crystallographic Data Centre under CCDC deposition number 2240976.

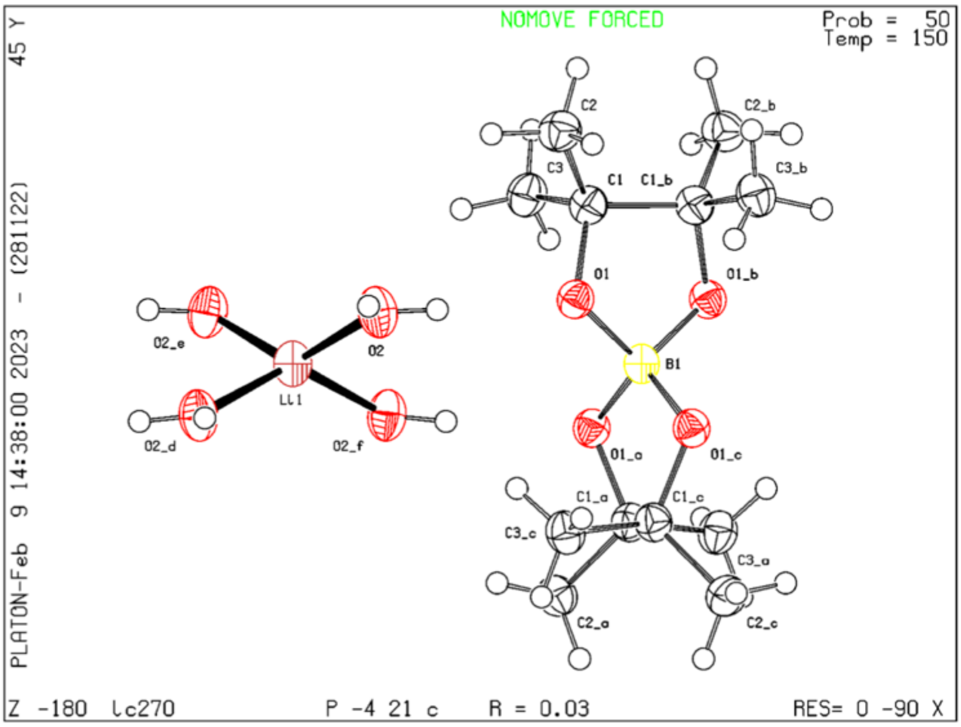

---

[2] O. V. Dolomanov, L. J. Bourhis, R. J. Gildea, J. A. K. Howard, H. Puschmann, *J. Appl. Cryst.*, **42**, 339–341 (2009).

[3] G. M. Sheldrick, *Acta Cryst.*, *A71*, 3–8 (2015).

[4] G. M. Sheldrick, *Acta Cryst.*, *C71*, 3–8 (2015).

Table S1. Crystal data and structure refinement for the compound.

Identification code	lc270
Empirical formula	$C_{12}H_{32}BLiO_8$
Formula weight	322.12
Temperature/K	150
Crystal system	tetragonal
Space group	$P\text{-}42_1c$
a/Å	6.9928(2)
b/Å	6.9928(2)
c/Å	17.6788(7)
α/°	90
β/°	90
γ/°	90
Volume/Å^3	864.48(6)
Z	2
ρ_{calc}g/cm^3	1.238
μ/mm^{-1}	0.099
F(000)	352.0
Crystal size/mm^3	0.42 x 0.32 x 0.15
Radiation	Mo Kα (λ = 0.71073)
2Θ range for data collection/°	4.608 to 64.55
Index ranges	-6 ≤ h ≤ 10, -10 ≤ k ≤ 6, -25 ≤ l ≤ 26
Reflections collected	11038
Independent reflections	11038 [R_{int} = 0.0144, R_{sigma} = 0.0077]
Data/restraints/parameters	11038/1/56
Goodness-of-fit on F^2	1.119
Final R indexes [I>=2σ (I)]	R_1 = 0.0339, wR_2 = 0.0952
Final R indexes [all data]	R_1 = 0.0350, wR_2 = 0.0960
Largest diff. peak/hole / e Å^{-3}	0.19/-0.23
Flack parameter	1.2(11)

Table S2. Fractional Atomic Coordinates (×10^4) and Equivalent Isotropic Displacement Parameters (Å^2×10^3) for the compound. U_{eq} is defined as 1/3 of the trace of the orthogonalised U_{IJ} tensor.

Atom	*x*	*y*	*z*	U(eq)
O1	1663.2(10)	9768.0(11)	5516.1(4)	28.92(19)
O2	5133.0(14)	7567.6(13)	5413.6(5)	39.6(2)
C003	932.4(14)	9408.0(15)	6269.8(6)	30.0(2)
C004	2385.9(16)	10043(2)	6861.8(6)	36.0(2)
C005	550.4(17)	7267.1(16)	6351.5(7)	35.0(2)
B006	0	10000	5000	27.9(4)
Li00	5000	5000	5000	34.7(7)

Table S3. Anisotropic Displacement Parameters ($Å^2×10^3$) for the compound. The Anisotropic displacement factor exponent takes the form: $-2\pi^2[h^2a^{*2}U_{11}+2hka^*b^*U_{12}+…]$.

Atom	U_{11}	U_{22}	U_{33}	U_{23}	U_{13}	U_{12}
O1	25.4(3)	30.3(4)	31.0(3)	0.9(3)	-0.2(2)	0.0(3)
O2	32.4(4)	30.8(4)	55.5(5)	-5.2(3)	1.1(4)	-0.7(3)
C003	28.1(4)	30.4(4)	31.4(4)	1.0(3)	0.0(3)	-1.0(3)
C004	31.5(4)	41.8(5)	34.8(4)	-0.3(4)	-3.3(4)	-1.9(5)
C005	36.0(5)	29.5(5)	39.5(5)	4.3(4)	-0.2(4)	-0.7(4)
B006	25.6(5)	25.6(5)	32.7(8)	0	0	0
Li00	30.2(10)	30.2(10)	43.7(18)	0	0	0

Table S4. Bond Lengths for the compound.

Atom	Atom	Length/Å
O1	C003	1.4491(13)
O1	B006	1.4871(7)
O2	Li00	1.9409
C003	C003[1]	1.5446(19)
C003	C004	1.5249(14)
C003	C005	1.5276(15)

[1] -X,2-Y,+Z

Table S5. Bond Angles for the compound.

Atom	Atom	Atom	Angle/˚	Atom	Atom	Atom	Angle/˚
**C003**	O1	B006	107.90(6)	O1[3]	B006	O1[1]	112.12(3)
**O1**	C003	C003[1]	101.81(6)	O1[2]	B006	O1[1]	112.12(3)
**O1**	C003	C004	110.20(9)	O1	B006	O1[3]	112.11(3)
**O1**	C003	C005	108.59(8)	O2	Li00	O2[4]	98.2
**C004**	C003	C003[1]	113.99(9)	O2[5]	Li00	O2[6]	98.2
**C004**	C003	C005	109.70(9)	O2	Li00	O2[5]	135.74(6)
**C005**	C003	C003[1]	112.19(11)	O2[4]	Li00	O2[6]	135.74(6)
**O1**	B006	O1[2]	112.11(3)	O2[4]	Li00	O2[5]	98.2
**O1**	B006	O1[1]	104.30(6)	O2	Li00	O2[6]	98.2
**O1[2]**	B006	O1[3]	104.30(6)				

[1] -X,2-Y,+Z; [2] -1+Y,1-X,1-Z; [3] 1-Y,1+X,1-Z; [4] 1-Y,+X,1-Z; [5] 1-X,1-Y,+Z; [6] +Y,1-X,1-Z

Table S6. Torsion Angles for the compound.

A	B	C	D	Angle/˚
C003	O1	B006	O1[1]	13.41(5)
C003	O1	B006	O1[2]	134.95(5)
C003	O1	B006	O1[3]	-108.12(5)
B006	O1	C003	C003[1]	-32.96(11)
B006	O1	C003	C004	-154.26(8)
B006	O1	C003	C005	85.56(9)

[1] -X,2-Y,+Z; [2] 1-Y,1+X,1-Z; [3] -1+Y,1-X,1-Z

Table S7. Hydrogen Atom Coordinates (Å×10^4) and Isotropic Displacement Parameters (Å^2×10^3) for the compound.

Atom	*x*	*y*	*z*	U(eq)
H2A	4191.01	8334.35	5432.73	59
H2B	6111.02	8279.16	5462.41	59
H00A	2765.31	11338.19	6762.39	54
H00B	3486.79	9224.69	6839.9	54
H00C	1820.53	9965.77	7355.67	54
H00D	143.48	6996.53	6858.97	52
H00E	1700.55	6568.7	6243.86	52
H00F	-432.89	6890.61	6003.05	52

5. DFT calculations

5.1. Computational details for the DFT study of the borylation reactions

To study the energetics of the borylation reactions, the energy of key structures on the potential energy surface was evaluated by means of density functional theory (DFT) with the ωB97-XD functional. This choice was based on previous studies performed by the group of Ito.[1,2] Geometry optimisations were performed using the 6-31G(d) basis set and additional single point corrections were computed using the 6-311+G(2d,2p) basis set.[3,4,5] The molecular system was embedded in a dielectric cavity mimicking methanol as solvent (polarizable continuum model, PCM).[6] All the calculations were performed with the Gaussian 16 software.[7]

The transition states (TS) or the reactions were initially located by scanning the PES along specific internal coordinates. TS geometries were then optimised and finally validated by standard normal mode calculations. For each TS, the single mode corresponding to an imaginary frequency was used to define an intrinsic reaction coordinate (IRC) and proceed downhill towards the reactants and products of the elementary step.[8] Finally, the end points of all IRCs were optimised to get the structure of all minima on the PES. Each stationary point was again confirmed by standard normal mode calculations. Graphical representations were made with the VMD software.[9]

5.2. Optimised geometries for the borylation of 3-methyl-3-nitrobutene

As mentioned in the main text, our first investigation of the reaction mechanism of the borylation reaction (see also Figure 3) was initiated from 3-methyl-3-nitrobutene and CuXantphos(Bpin) as reactive species. Figure S1 shows the corresponding optimised structures, as well as the resulting products of

---

[1] H. Iwamoto, Y. Ozawa, Y. Takenouchi, T. Imamoto, H. Ito, *J. Am. Chem. Soc.*, **143**, 6413–6422 (2021).

[2] Chai, J.-D.; Head-Gordon, M., *Phys. Chem. Chem. Phys.*, **10**, 6615–6620 (2008).

[3] R. Ditchfield, W. J. Hehre, J. A. Pople, *J. Chem. Phys.*, **54**, 724–728 (1971).

[4] W. J. Hehre, R. Ditchfield, J. A. Pople, *J. Chem. Phys.*, **56**, 2257–2261 (1972).

[5] P. C. Hariharan, J. A. Pople, *Theor. Chem. Acc.*, **28**, 213–222 (1973).

[6] J. Tomasi, B. Mennucci, R. Cammi, *Chem. Rev.*, **105**, 2999–3094 (2005).

[7] Gaussian 16, Revision C.01, M. J. Frisch, G. W. Trucks, H. B. Schlegel, G. E. Scuseria, M. A. Robb, J. R. Cheeseman, G. Scalmani, V. Barone, G. A. Petersson, H. Nakatsuji, X. Li, M. Caricato, A. V. Marenich, J. Bloino, B. G. Janesko, R. Gomperts, B. Mennucci, H. P. Hratchian, J. V. Ortiz, A. F. Izmaylov, J. L. Sonnenberg, D. Williams-Young, F. Ding, F. Lipparini, F. Egidi, J. Goings, B. Peng, A. Petrone, T. Henderson, D. Ranasinghe, V. G. Zakrzewski, J. Gao, N. Rega, G. Zheng, W. Liang, M. Hada, M. Ehara, K. Toyota, R. Fukuda, J. Hasegawa, M. Ishida, T. Nakajima, Y. Honda, O. Kitao, H. Nakai, T. Vreven, K. Throssell, J. A. Montgomery Jr., J. E. Peralta, F. Ogliaro, M. J. Bearpark, J. J. Heyd, E. N. Brothers, K. N. Kudin, V. N. Staroverov, T. A. Keith, R. Kobayashi, J. Normand, K. Raghavachari, A. P. Rendell, J. C. Burant, S. S. Iyengar, J. Tomasi, M. Cossi, J. M. Millam, M. Klene, C. Adamo, R. Cammi, J. W. Ochterski, R. L. Martin, K. Morokuma, O. Farkas, J. B. Foresman, D. J. Fox, Gaussian, Inc., Wallingford CT (2016).

[8] K. Fukui, *Acc. Chem. Res.*, **14**, 363–368 (1981).

[9] W. D. A. Humphrey, J. Schulten, *Mol. Graphics*, **14**, 33–38 (1996).

the reaction, as obtained from our DFT calculations. It is worth stressing that [Cu]-Bpin is initially obtained *in situ* from a reaction between [Cu]-OMe and B_2Pin_2, which is the starting point in Figure 3.

For the sake of clarity and in order to put the emphasis on chemistry, we provide simplified representations of the stationary points of *syn*/*anti* cupro-borylation and β-nitrite elimination in a separate figure, namely Figure S2.

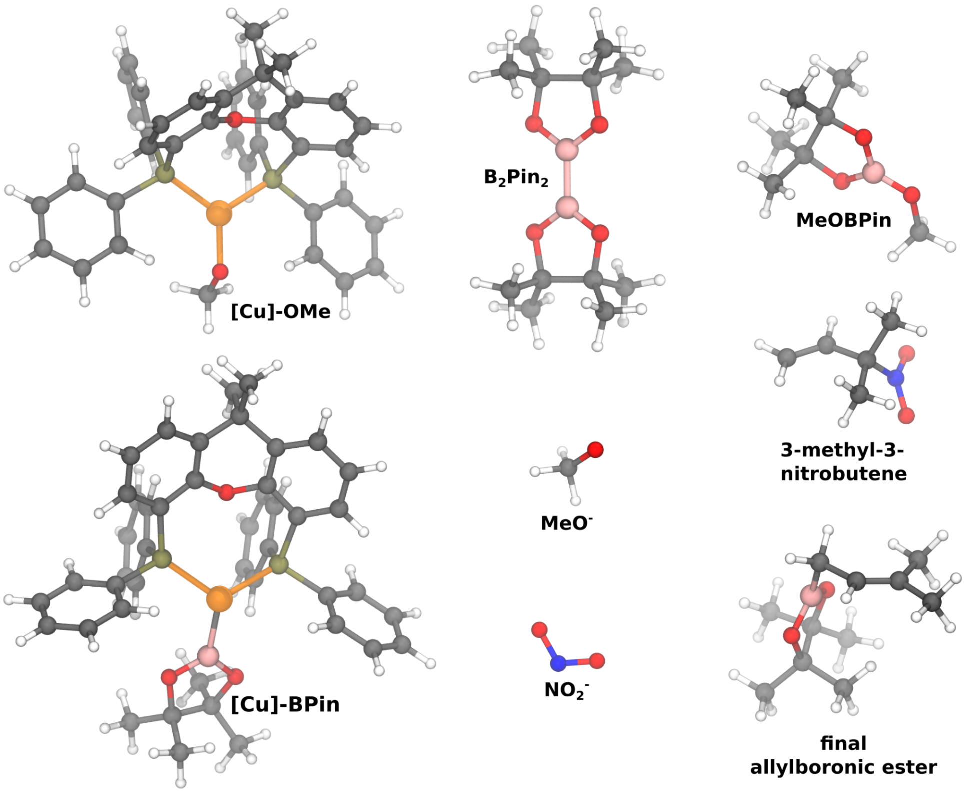

Figure S1: All-atom representation of our optimised molecular structures of the reactant and products at infinite separation shown in Figure 3. All atoms are represented explicitly. The molecular structures of all the intermediate chemical steps, namely *syn*/*anti* cupro-borylation and β-nitrite elimination, are shown with simplified representations in Figure S2.

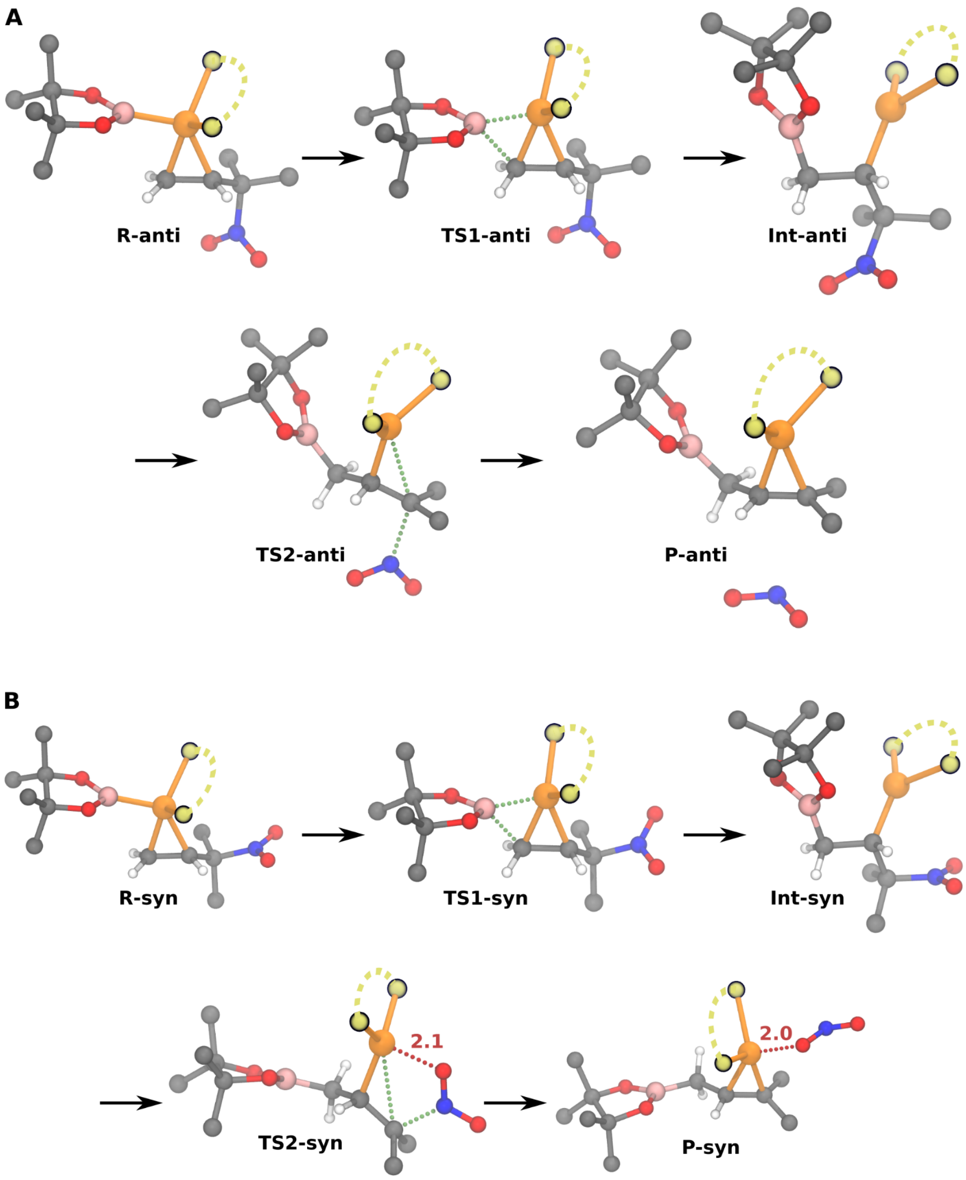

Figure S2. Optimised molecular structures for the cupro-borylation and β-nitrite elimination steps shown in Figure 3. **(A)** *anti* pathway. **(B)** *syn* pathway. For the sake of clarity, only vinylic and allylic hydrogen atoms are shown, and the Xantphos moiety is represented by a tan dashed arc. Forming or breaking bonds are represented by green dotted lines. Key Cu-O interactions in TS2-*syn* and P-*syn* are highlighted by red dotted lines and the corresponding distances are given in Angstrom. Additional (full) molecular structures of the corresponding reactants and products (end points in Figure 3) are shown in Figure S1.

5.3. NCIPlot analysis: rationalisation of the difference in energy between Int-*anti* and Int-*syn* in the borylation reaction

To have a better insight into the difference in energy between **Int-*anti*** and **Int-*syn***, we studied the non-covalent interactions (NCI) within these two molecular systems by means of a so-called NCIPlot analysis.[10,11] This analysis resorts on the electron density and its derivatives extracted from a DFT calculation. It allows to visualise NCIs in 3D space using a colour scale, as shown in Figure S3. More specifically, we used the NCIPlot software together with the keyword “INTERMOLECULAR”, in order to only represent NCIs between the [Cu] complex and the substrate. In both **Int-*syn*** and **Int-*anti***, much of the NCIs are van de Waals interactions between the substrate and the aryl groups of the Xantphos ligand, as exhibited by the yellow/green surfaces separating the two moieties (see the caption of Figure S3 for a detailed definition of the colour scale). However, in the case of **Int-*syn*** an additional red lenticule appears between the Cu centre and the oxygen of the nitro group. This highlights a strong repulsive interaction, which we interpret as being the key factor explaining the high free energy difference between **Int-*syn*** and **Int-*anti***, the latter being the most stable (see Figure 3).

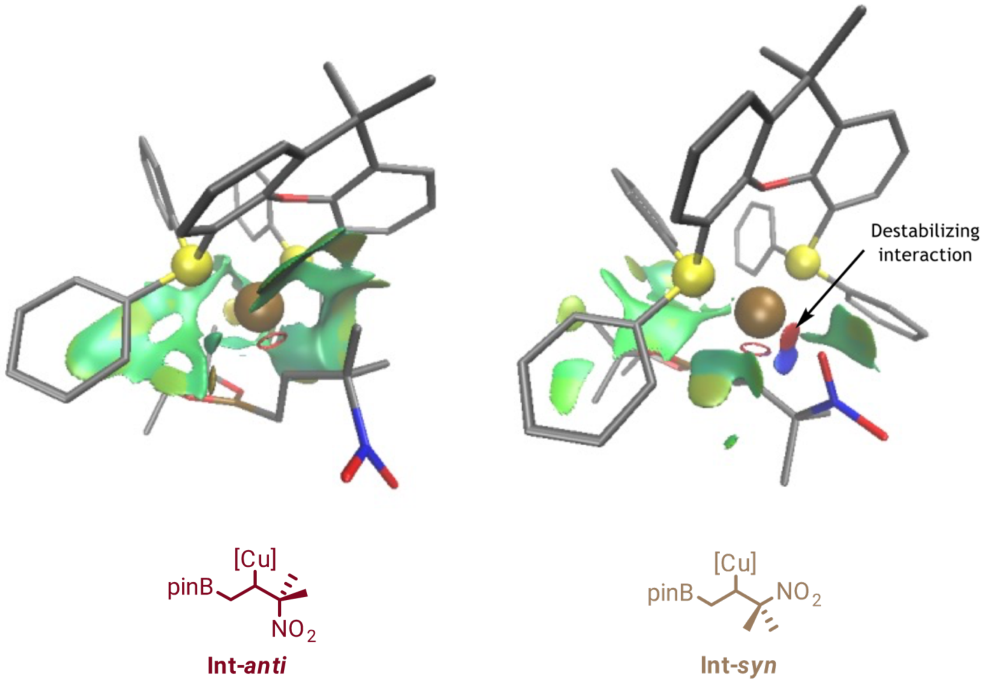

Figure S3. NCIPlots of **Int-*anti*** (left) and **Int-*syn*** (right). Blue coloured surfaces stand for stabilising interaction, red surfaces for strong repulsive interactions and green for weak van der Waals interactions (blue green = weak stabilising interaction, yellow green = weak destabilising interaction)

---

[10] E. R. Johnson, S. Keinan, P. Mori-Sanchez, J. Contreras-Garcia, A. J. Cohen, W. Yang, *J. Am. Chem. Soc.*, **132**, 6498–6506 (2010).

[11] J. Contreras-Garcia, E. R. Johnson, S. Keinan, R. Chaudret, J.-P. Piquemal, D. N. Beratan, W. Yang., *J. Chem. Theory Comput.*, **7**, 625–632 (2011).

5.4. Optimised geometries and additional free energy profiles for the borylation of 3-fluoro-3-nitrobutene

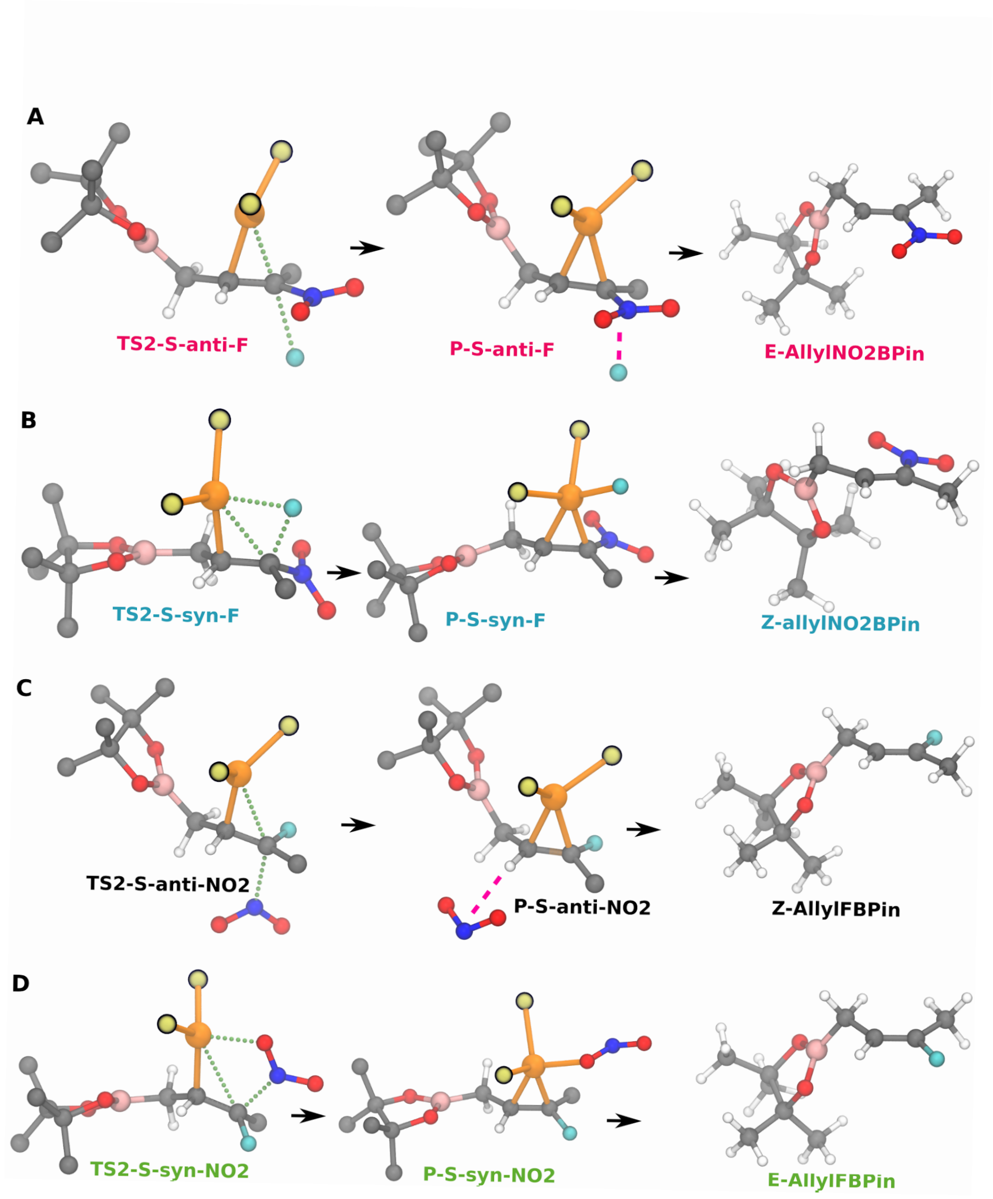

Figure S4: Optimised molecular structures for the β-elimination of the fluorine or nitro groups starting from a S configuration, as shown in Figure 5. **(A)** *anti*-Elimination of the F^- ion. **(B)** *syn*-Elimination of the F^- ion. **(C)** *anti*-Elimination of the NO_2^- group. **(D)** *syn*-Elimination of the NO_2^- group. For all transition states and the resulting adducts, only vinylic and allylic hydrogen atoms are shown, and the Xantphos moiety is represented by a tan dashed arc. Forming or breaking bonds are represented by green dotted lines. Ion pairs are indicated by magenta dashed lines. All-atom representations are used for the final products.

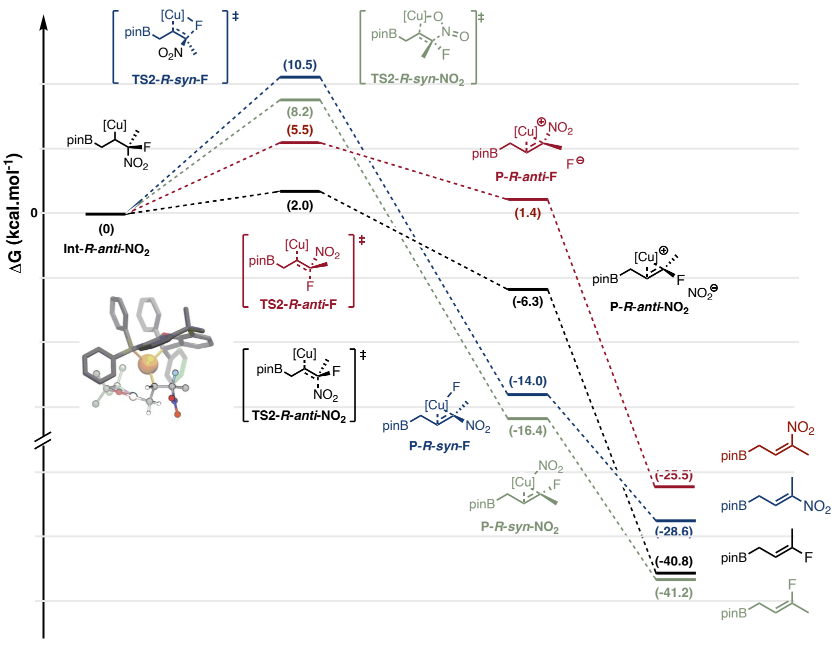

Figure S5: Comparison of the β-elimination pathways with the fluorine or nitro leaving groups, starting from the *R* intermediate. The 3D molecular structures of all TSs, adducts and final products are shown in Figure S6.

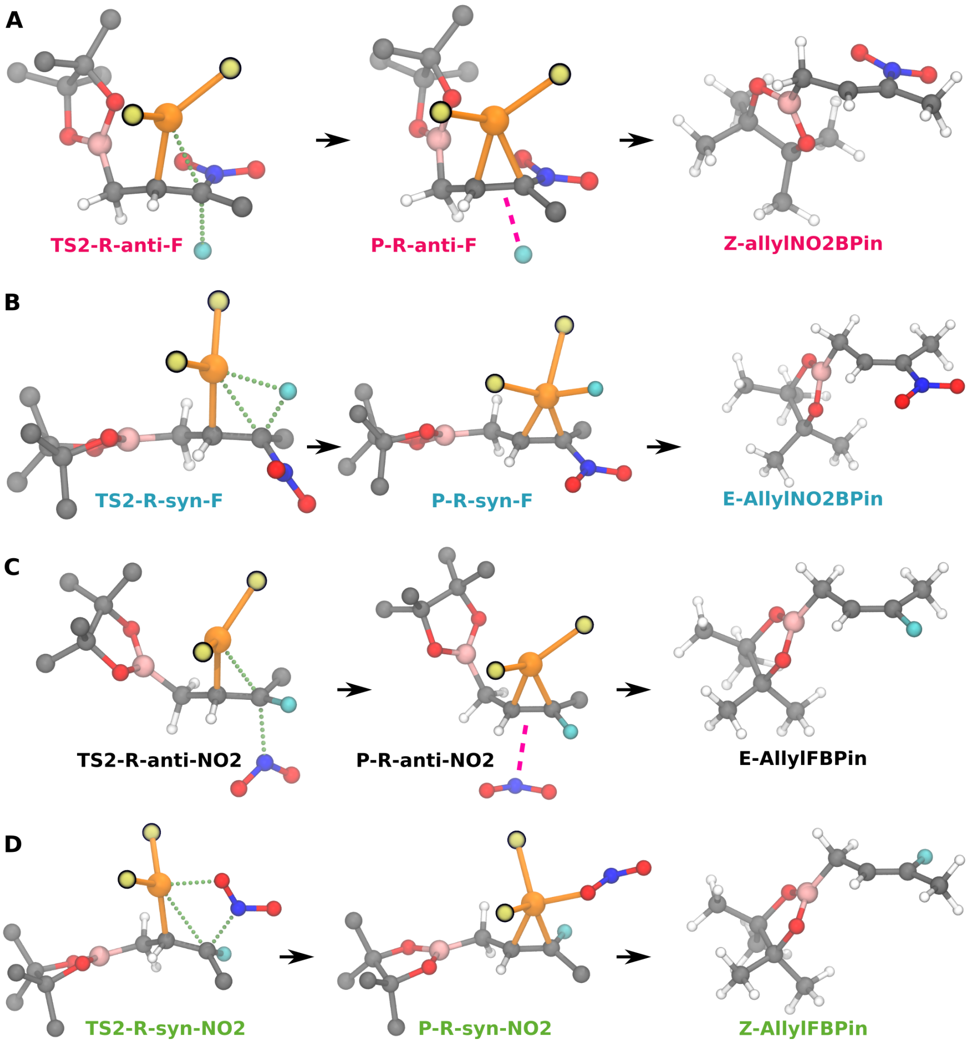

Figure S6. Optimised molecular structures for the β-elimination of the fluorine or nitro groups starting from a *R* configuration, as shown in Figure S5. **(A)** *anti*-Elimination of the F^- ion. **(B)** *syn*-Elimination of the F^- ion. **(C)** *anti*-elimination of the NO_2^- group. **(D)** *syn*-Elimination of the NO_2^- group. For all transition states and the resulting adducts, only vinylic and allylic hydrogen atoms are shown, and the Xantphos moiety is represented by a tan dashed arc. Forming or breaking bonds are represented by green dotted lines. Ion pairs are indicated by magenta dashed lines. All-atom representations are used for the final products.

5.5. Mechanism study for the scandium-catalysed lactonization leading to the formation of compound 10, and their corresponding 3D structures

The M062X functional[12] was employed together with the 6-31G(d,p) basis set to model the two different lactonization pathways, PCM was employed to model the solvent (toluene) in this reaction. Therefore, two different pathways were obtained associated with the formation of the lactone bearing the two aryl moieties in *cis* (red curve) or *trans* (blue curve) configuration with respect to the six-membered ring (Figure S7).

The first step of the lactonization reaction consists in the addition of the alcohol moiety on the carbonyl compound. This process can be considered barrierless with activation barriers below 0.5 kcal.mol^{-1} for both pathways. The scandium catalyst plays a dual role in this step: the electrophilicity of the carbonyl compound is enhanced by its coordination to the Sc, while the triflate anions coordinated to the metal center act as a proton shuttle, facilitating the addition of the alcohol and its deprotonation. The addition of the alcohol led to the formation of two intermediates, **Int-*cis*** and **Int-*trans***. A slight difference of 3.0 kcal.mol^{-1} in favor of **Int-*trans*** isomer is observed. The elimination of the methoxy-group in these two structures allows the formation of the corresponding lactone **P-*trans*** and **P-*cis***, which both are absolute minima in energy for the transformation, indicating that this process is irreversible. This elimination process is assisted in both case by the trifluoromethane sulfonic acid, which acts as a proton transfer. However, in the case of **Int-*trans*** the activation barrier associated with the departure of the methoxy group is 1.6 kcal.mol^{-1} higher than for **Int-*cis*** compound. Hence, this difference in energy might be the origin of the diastereoselectivity of the lactonization reaction for compound **10**.

[12] Y. Zhao, D. G. Truhlar, *Theor. Chem. Acc.*, **120**, 215-241 (2008).

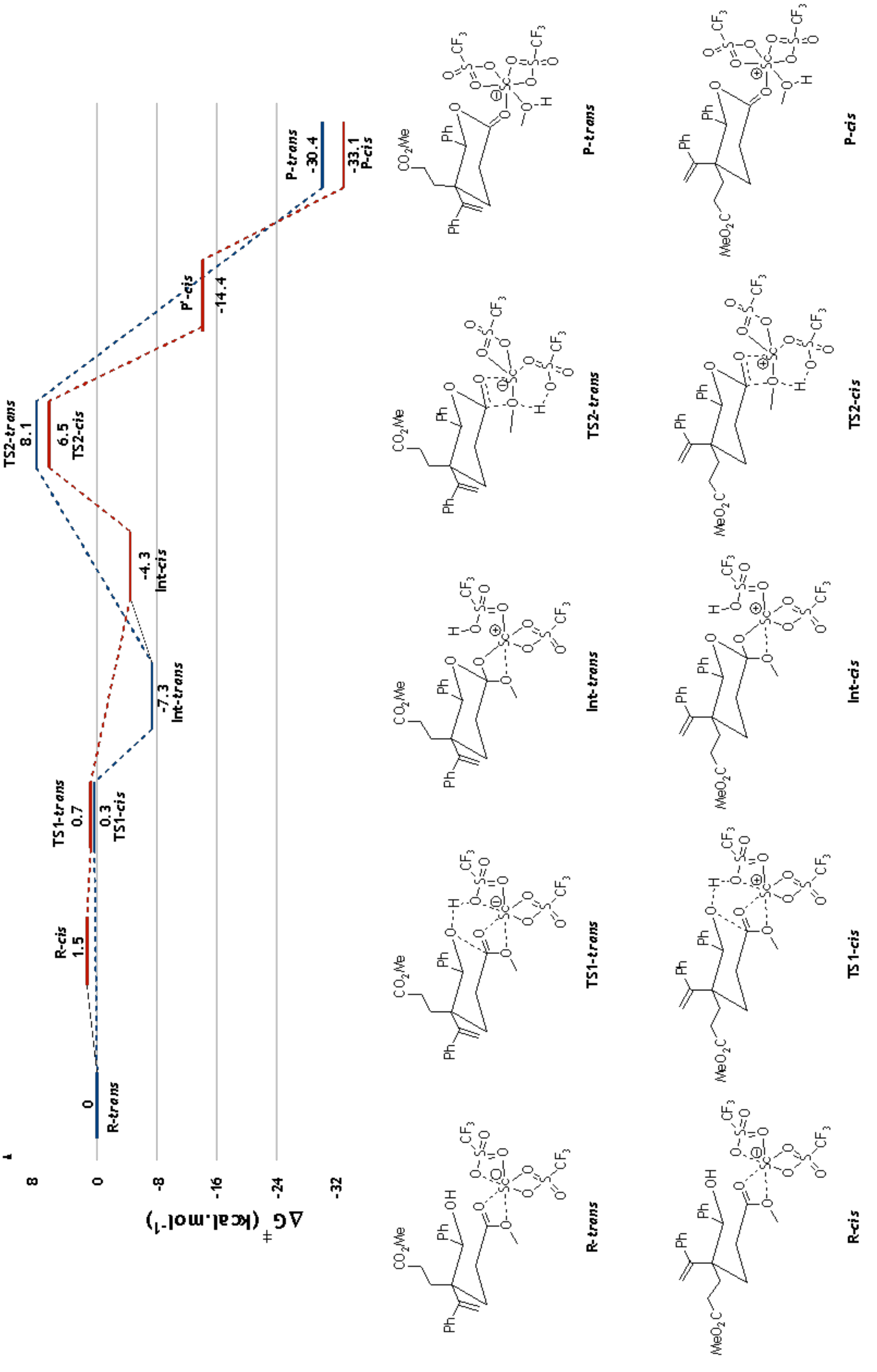

Figure S7. Free energy profiles associated with the Sc-catalysed formation of *cis* and *trans* lactones and ChemDraw structures of the corresponding stationary points in the free energy profile.

6. Additional experiments to improve the stereoselectivity

To increase the stereoselectivity in the case of unsymmetrical allylic nitroalkanes, we tested several conditions. The results are summarized in the table below.

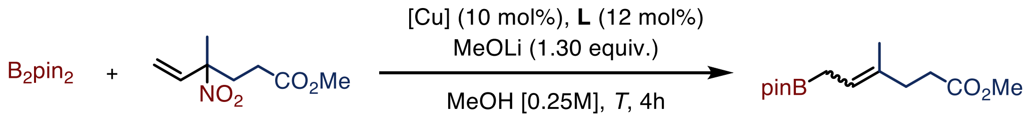

(1.30 equiv.) **1b** **2b**

Entry	[Cu] (10 mol%)	L (12 mol%)	Temp (°C)	Conv.[a] (%)	Yield[a] (%)	*E/Z* ratio[a]
1	CuCN	-	25	91	81	76:24
2	CuCN	-	0	88	73	76:24
3	CuCN	-	–20	0	-	-
4	CuIPrCl	-	25	55	20	70:30
5	CuI	dppm	25	73	50	74:26
6	CuI	dppe	25	90	74	74:26
7	CuI	dppb	25	62	40	69:31
8	CuI	dppf	25	53	41	66:34
9	CuI	XantPhos	25	54	40	75:25
10	CuI	PPh_3	25	78	64	68:32
11	CuI	PCy_3 HBF_4	25	100	90	60:40

[a] Determined by ^1H NMR on the crude reaction mixture using 1,3,5-trimethoxybenzene as internal standard.
